# Outlining the Grb2 interactome data and its interacting partners in HEK293 cells in absence and presence of epidermal growth factor

**DOI:** 10.1016/j.dib.2019.104082

**Published:** 2019-05-30

**Authors:** Shweta Duggal, Mukul Kumar Midha, Ajay Kumar, Kanury V.S. Rao

**Affiliations:** a International Centre for Genetic Engineering and Biotechnology (ICGEB), Aruna Asif Ali Marg, New Delhi 110067, India; b Drug Discovery Research Center (DDRC), Translational Health Science and Technology Institute (THSTI), NCR Biotech Science Cluster, 3rd Milestone, Faridabad-Gurgaon Expressway, Faridabad 121001, Haryana, India

**Keywords:** Grb2, EGF, Interactome, SILAC, AP-MS

## Abstract

Growth factor receptor-bound protein 2 (Grb2) is an adaptor protein involved in the signal transduction pathways. This dataset enlists proteins which interact with Grb2 in the presence and absence of a mitogenic stimulus. Grb2 expressing HEK293 cells were cultured in light and heavy labeled SILAC media. Normal lysine and arginine were incorporated as light labels while 8 and 10 Da heavier labels of respective isotopes were used for heavy labeling. While light labeled cells were used to enrich basal Grb2 interactome, the heavy labeled cells were stimulated in presence of epidermal growth factor (EGF) to investigate the altered Grb2 interactome dynamics. Equal number of EGF stimulated and non-stimulated cells was pooled, lysed and subjected to affinity purification coupled to mass spectrometry (AP-MS). The variety of Grb2 protein partners changed as a consequence of EGF stimulation. Additionally, SILAC labeling helped in quantitative estimation of altered association of a few interactors with the bait protein. Data are available via PRIDE repository with the dataset identifier PXD012957 (https://www.ebi.ac.uk/pride/archive/projects/PXD012957).

**Table:** Specifications table

Subject area	*Biology*
More specific subject area	*Proteomics*
Type of data	*Mass Spectrometry raw files, figure*
How data was acquired	*Mass Spectrometry, AB Sciex 5600 Triple TOF*
Data format	*Raw and analysed*
Experimental factors	*Stable Grb2 expressing HEK293 cell line with and without EGF stimulation*
Experimental features	*SILAC labeling, LysC - Trypsin - Chymotrypsin Digestion, peptide separation through nano-flow liquid chromatography on a nanoflex system coupled to a triple TOF 5600 mass spectrometer (AB Sciex)*
Data source location	*Delhi-NCR, India*
Data accessibility	*Data is within this article and available in* the ProteomeXchange Consortium via the PRIDE partner repository with the dataset identifier PXD012957 *(*https://www.ebi.ac.uk/pride/archive/projects/PXD012957).
Related research article	*Bisson N, James A, Ivosev G, Tate SA, Bonner R, Taylor L, Pawson T. Selected reaction monitoring mass spectrometry reveals the dynamics of signaling through the GRB2 adaptor. Nat Biotechnol. 2011 29:653-658.**[2]*

**Table:** 

**Value of the data** • The delineated dataset unveils new interacting partners of Grb2 protein apart from some of the earlier reported interactors. • The enriched interactome of Grb2, in presence and absence of EGF, will be helpful to better understand the Grb2 signalling under the influence of mitogenic (EGF) stimulation. • Incorporation of four independent biological replicates ensures high confidence dataset for Grb2 interactome. • Amino acid labeling in cell culture coupled to mass spectrometry is a well-accepted technique to understand the dynamics of protein-protein interactions and can be used in a context dependent manner in further studies.

## 1 Data

**Fig. 1:**
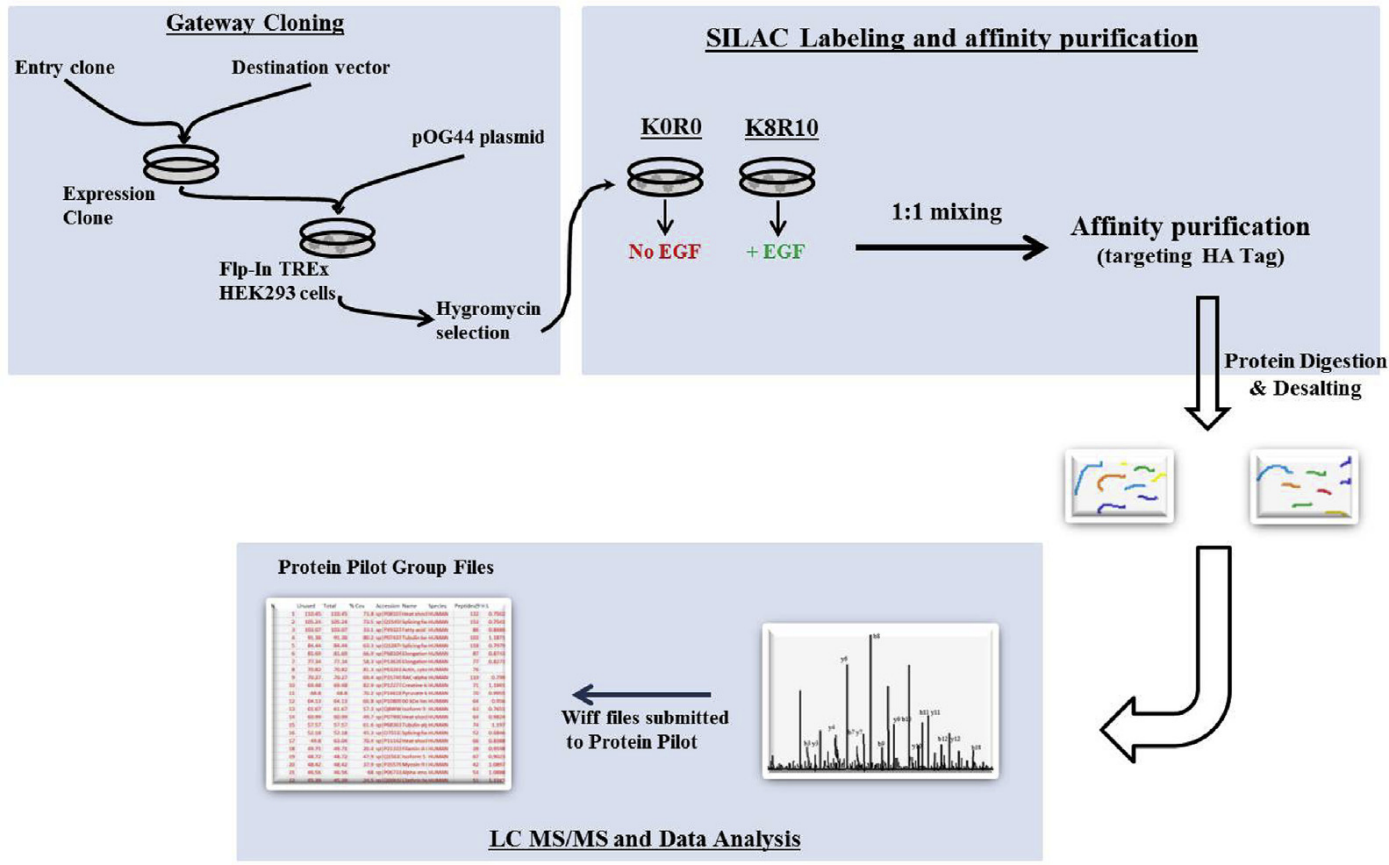
Schematic overview showing the experimental workflow.

Grb2 expressing stable HEK293 cell line was generated and subjected to SILAC labeling to examine dynamic changes in Grb2 interactome in presence and absence of EGF stimulus. A gateway expression vector for HA tagged Grb2 gene was used to produce the recombinant cell line. HA tag was used to affinity purify Grb2 protein along with its interacting partners. SILAC (Stable Isotope Labeling in Cell Culture) labeling enabled the quantitation of changing association dynamics of interacting partners of Grb2 protein in presence and absence of EGF. Four independent biological replicates of stimulated as well as non-stimulated cells were processed and analyzed. Affinity purified protein samples were triple digested with trypsin, LysC and chymotrypsin. The peptides were then acquired on AB Sciex 5600 triple TOF mass spectrometer and the results were obtained as 8 RAW file pairs (wiff and corresponding wiff. scan files). Analysis of the wiff files using protein pilot software version 5.0 resulted in 16 protein pilot group files (4 group files per biological replicate). A representative snapshot of the experiment workflow is as given in Fig. 1. Data is publicly available via ProteomeXchange with identifier ProteomeXchange: PXD012957 (https://www.ebi.ac.uk/pride/archive/projects/PXD012957).

## 2 Experimental design, materials, and methods

### 2.1 Stable cell line generation

In order to generate a tetracycline (Tet) inducible Grb2 expression vector, pENTR221 entry vector containing Grb2 ORF (GE Dharmacon) was used for LR recombination reaction, together with modified destination vector (pcDNA/FRT/TO); pcDNA/FRT/TO was a kind gift from Dr. Matthias Gstaiger (Institute of Molecular Systems Biology, ETH Zurich, Zurich, Switzerland). The modified destination vector included a HA tag for selective enrichment of Grb2 interacting partners.

Flp-In T-REx HEK293 cells (from Invitrogen), stably expressing the Tet-repressor, were cultured in DMEM medium containing 10% tetracycline screened FBS and supplemented with 100 μg/ml zeocin and 15 μg/ml blasticidin. Grb2 expression vector and pOG44 vector were co-transfected into Flp-In T-REx HEK293 cells using Xtremegene 9 transfection reagent, as instructed by the manufacturer (Roche).

Hygromycin selection (75 μg/ml), to produce stable cell line, was initiated 48 hours after transfection and continued over 2 weeks with media refreshed twice a week. Tet dosage, required to induce Grb2 expression, was optimized by monitoring Grb2 expression by western blotting after adding Tet to culture media over a range of concentrations (0.1 μg/ml to 5 μg/ml). Stable expression of Grb2 gene was obtained at 1 μg/ml Tet concentration. Anti-HA antibody (#SC-7392; mouse monoclonal, dilution 1:3000, from Santa Cruz) as well as anti-Grb2 antibody (ab32037; rabbit monoclonal, dilution 1:5000; Abcam) was used to validate Grb2 expression by Western blot method.

### 2.2 SILAC (Stable Isotope Labeling in Cell Culture) labeling

Stable HEK293 cell line expressing Grb2 protein was cultured in SILAC medium containing “lighter” (K0R0) or “heavier” (K8R10) isotopes of lysine and arginine for over 5 cell doublings to allow complete label incorporation [1]. Tet was supplemented to the culture medium at 70% confluency. After 24 hours of Tet induction, K8R10 labeled cells were stimulated with 100ng/ml EGF for 20 minutes [2]. Light labeled cells were supplemented with Tet at the same time as K8R10 labeled cells but were not stimulated with EGF. Both K0R0 and K8R10 labeled cells were trypsinized simultaneously. Cells were counted by trypan blue staining method and pelleted separately following centrifugation at 1500 rpm for 10 minutes. Multiple pellets for stimulated as well as non-stimulated cells were generated for K0R0 and K8R10 labeled cells and stored at −80 °C, until used.

### 2.3 HA tagged affinity purification

Approximately 45 million stimulated and non-stimulated Grb2 expressing cells were pooled and lysed for 1 hour on ice in IP buffer (150mM NaCl; 50mM Tris-HCl pH7.5; 1% NP-40; 1X protease inhibitor cocktail and 1mM PMSF) [3]. Cell lysates were centrifuged at 10,000 rpm for 15 minutes at 4 °C to clear-off the residual debris. Grb2 interactome was selectively enriched by targeting the HA tag as instructed in kit manual (Invitrogen #26180). Briefly, 50 μl of HA agarose beads were pre-washed in TBS buffer and mixed with clear cell lysate. The mixing was mediated at 4 °C on a rotary shaker for 2 hours. Subsequently, the beads were washed thrice with ten bead volumes of TBS-T buffer. Grb2 protein along with its interacting partners were eluted thrice in 50 μl HA peptide solution (250 μg/ml) per elution step. Similar pull-down experiments were repeated for four independent biological replicates. Eluates from each replicate set were pooled, lyophilized and saved at −80 °C, until further processing.

### 2.4 Protein digestion and sample preparation for LC-MS/MS

Each biological replicate was processed independently. Lyophilized eluates were re-suspended in 40 μl of 100mM ammonium bicarbonate, vortexed well and supplemented with 2 μl of 2% SDS (denaturant buffer). Protein samples were reduced in presence of 4 μl of 50mM TCEP [Tris-(2-carboxyethyl) phosphine] at 60 °C for 1 hour. Thereafter, the reduced cysteine residues were blocked in 2 μl of 200mM MMTS (methyl methanethiosulfonate) by incubating at room temperature for 10 minutes. Protein digestion was initiated in a 37 °C water bath by adding 5 μl of 0.1 μg/μl endo-proteinase LysC. After 4 hours of LysC treatment, 0.5 μl of trypsin (1 μg/μl) and 0.5 μl of chymotrypsin (1 μg/μl) was supplemented to the sample and incubation was continued for another 12–16 hours at 37 °C. Protein digestion was terminated by adding 2.5 μl formic acid to a final concentration of 5%.

Acidified samples were lyophilized and later subjected to desalting using monospin C-18 columns (Waters). Pre-conditioned C-18 columns were equilibrated with 3% acetonitrile (ACN) in 0.1% formic acid (FA). The lyophilized peptide samples were re-suspended in 3% ACN in 0.1% FA and allowed to bind to C-18 columns for 10 minutes. To ensure complete binding, samples were passed twice through the column. After 10 stringent washes with 3% ACN in 0.1% FA, the digested peptides were first eluted once in 40% ACN followed by two elutions in 60% ACN. The three eluates were finally pooled and lyophilized.

To remove any residual salt, the eluted peptides were re-suspended in 500 μl of 5mM ammonium formate (pH 2.5) in 30% ACN and gently vortexed to mix. The cation exchange cartridge was fixed and conditioned before loading the sample. Following sample loading, 1ml of 5mM ammonium formate was used thrice to wash the cartridge. The peptides were re-eluted twice with 400 μl of 500mM ammonium formate (pH 2.5) in 30% ACN, pooled and lyophilized.

### 2.5 LC-MS/MS analysis

All samples were analysed by nano-flow liquid chromatography (LC) on a nanoflex system (Eksigent Technologies, AB SCIEX) coupled to a triple TOF 5600 Mass Spectrometer (AB SCIEX; Concord, Canada). Each biological replicate was injected twice as two technical replicates; LC solvents included mobile phase A (2% ACN in 0.1% FA) and mobile phase B (98% ACN in 0.1% FA). For optimal sample delivery reproducibility, the auto-sampler was operated in full injection mode overfilling the 1 μl loop with 3 μl sample. Peptides were first run through trap column for 30 minutes and then eluted out from analytical column (chromolith column-particle size 5 μm, length 15cm, 75 μm ID) with a flow rate of 350nl/minute. Auto-calibration was done using 25fmol of beta-galactosidase after every two runs. Peptides were injected into mass spectrometer by using 10μm SilicaTip electrospray PicoTip emitter and the eluted peptides were monitored by following ion source parameters - IHT = 130°, ISVF = 1.9kv, GS1 = 23, curtain gas = 25. MS data was acquired in information-dependent acquisition (IDA) mode using Analyst TF 1.6 software. Mass spectra and tandem mass spectra were recorded in positive-ion and “high-sensitivity” mode; LC-MS/MS analysis was performed using TOF-MS survey scan from 350 to 1250 m/z. Accumulation time of scan was 500 ms, followed by fragmentation of 30 most abundant ion peaks. Rolling collision energy was automatically controlled by IDA rolling collision energy parameter script. Selection criteria for parent ion to be fragmented included intensity - where ions had to be greater than 150cps, mass tolerance of 50mDa, with a charge state of +2 to +5. The ions, once fragmented, were excluded from further fragmentation for 12 seconds.

### 2.6 Database search

For each independent biological replicate two wiff and two wiff. scan files were generated that represented the technical replicates. The technical replicate files for each replicate sample were pooled before searching against uniprot_humanswissprot database (release February 2019) using protein pilot version 5.0 (revision no. 4769). Trypsin and LysC digested files were acquired together while Chymotrypsin digested sample files were acquired separately. Protein pilot search was based on the paragon algorithm, a part of default statistics of the software. Settings used for paragon searches were as follows - (a) species as Homo sapiens, (b) LysC + Trypsin and Chymotrypsin as enzyme categories for different runs, (c) maximum missed cleavages = 2, (d) fixed modifications as SILAC label - K8R10; cysteine alkylation by methyl methanethiosulphonate (MMTS), (e) variable modifications as oxidation at methionine, which is a default option in protein pilot, (f) identification and SILAC (Lys+8, Arg+10) as sample types, and (g) “Search Effort” parameter “Thorough ID”, which gives us a broad search of various protein modifications, (h) mass tolerance for precursor and fragment ions were 0.05 and 0.1 Da, respectively. The parameters used for identification and quantification of differentially expressed proteins were following - (a) auto bias correction for heavy to light ratio. (b) the threshold of 1% accepted Global False discovery rate from fit (G-FDR-fit) proteins; (c) minimum protein confidence threshold cut-off of 95%; (d) at least one peptide with 95% confidence for the relative expression. Consequent lists of proteins identified in the two investigated conditions are depicted as Supplementary Table 1 (non-stimulated) and table 2 (EGF stimulated).

For each biological replicate, four protein pilot files representing the 2 SILAC labels (light and heavy) with two enzyme categories used for digestion (LysC + Trypsin and Chymotrypsin) were created individually; lighter label was represented as K0R0 (without EGF stimulation) and heavier label as K8R10 (with EGF stimulation). After processing and analyzing the two technical replicates for four independent biological replicates for each condition, we observed 40–50% reproducibility at protein levels amongst the analysed replicates.

The mass spectrometry proteomics data has been deposited to the ProteomeXchange Consortium via the PRIDE [4] partner repository with the dataset identifier PXD012957.
