# Supplementary material for: Outlining the Grb2 interactome data and its interacting partners in HEK293 cells in absence and presence of epidermal growth factor
Source: Data Brief. 2019 May 30;25:104082. doi: 10.1016/j.dib.2019.104082 (PMC6582235; doi:10.1016/j.dib.2019.104082)
Supplement: Supplementary file 1 [file mmc1.pdf]

Accession #	Gene ID	Name	Functions
**Q9H9L3**	I20L2	Interferon-stimulated 20 kDa exonuclease-like 2 OS=Homo sapiens OX=9606 GN=ISG20L2 PE=1 SV=1	3'-> 5'-exoribonuclease involved in ribosome biogenesis in the processing of the 12S pre-rRNA. Displays a strong specificity for a 3'-end containing a free hydroxyl group
**Q8N3C0**	ASCC3	Activating signal cointegrator 1 complex subunit 3 OS=Homo sapiens OX=9606 GN=ASCC3 PE=1 SV=3	3'-5' DNA helicase involved in repair of alkylated DNA. Promotes DNA unwinding to generate single-stranded substrate needed for ALKBH3, enabling ALKBH3 to process alkylated N3- methylcytosine (3mC) within double-stranded regions. Enhances NF-kappa-B, SRF and AP1 transactivation
**P38935**	SMBP2	DNA-binding protein SMUBP-2 OS=Homo sapiens OX=9606 GN=IGHMBP2 PE=1 SV=3	5' to 3' helicase that unwinds RNA and DNA duplices in an ATP-dependent reaction. Acts as a transcription regulator. Required for the transcriptional activation of the flounder liver- type antifreeze protein gene. Exhibits strong binding specificity to the enhancer element B of the flounder antifreeze protein gene intron. Binds to the insulin II gene RIPE3B enhancer region. May be involved in translation (By similarity). DNA-binding protein specific to 5'-phosphorylated single-stranded guanine-rich sequence related to the immunoglobulin mu chain switch regi [...] (993 aa)
**Q96SE0**	ABHD1	Protein ABHD1 OS=Homo sapiens OX=9606 GN=ABHD1 PE=1 SV=2	This gene is a member of the AB hydrolase superfamily and encodes a protein with an alpha/beta hydrolase fold. This domain is common to a number of hydrolytic enzymes of widely differing phylogenetic origins and catalytic functions.
**Q9UKV3**	ACINU	Apoptotic chromatin condensation inducer in the nucleus OS=Homo sapiens OX=9606 GN=ACIN1 PE=1 SV=2	ACIN1 confers RNA-binding to the complex. The ASAP complex can inhibit RNA processing during in vitro splicing reactions. The ASAP complex promotes apoptosis and is disassembled after induction of apoptosis. Involved in the splicing modulation of BCL2L1/Bcl-X (and probably other apoptotic genes); specifically inhibits formation of proapoptotic isoforms such as Bcl-X(S); the activity is different from the established EJC assembly and function. Induces apoptotic chromatin condensation after activation by CASP3. Regulates cyclin A1, but not cyclin A2, expression in leukemia cells.

**Q05682**	CALD1	Caldesmon OS=Homo sapiens OX=9606 GN=CALD1 PE=1 SV=3	Actin- and myosin-binding protein implicated in the regulation of actomyosin interactions in smooth muscle and nonmuscle cells (could act as a bridge between myosin and actin filaments). Stimulates actin binding of tropomyosin which increases the stabilization of actin filament structure. In muscle tissues, inhibits the actomyosin ATPase by binding to F-actin. This inhibition is attenuated by calcium-calmodulin and is potentiated by tropomyosin. Interacts with actin, myosin, two molecules of tropomyosin and with calmodulin. Also play an essential role during cellular mitosis [...] (793 aa)
**P60981**	DEST	Destrin OS=Homo sapiens OX=9606 GN=DSTN PE=1 SV=3	Actin-depolymerizing protein. Severs actin filaments (F- actin) and binds to actin monomers (G-actin). Acts in a pH- independent manner (165 aa)
**P63261**	ACTG	Actin, cytoplasmic 2 OS=Homo sapiens OX=9606 GN=ACTG1 PE=1 SV=1	Actins are highly conserved proteins that are involved in various types of cell motility and are ubiquitously expressed in all eukaryotic cells
**P60709**	ACTB	Actin, cytoplasmic 1 OS=Homo sapiens OX=9606 GN=ACTB PE=1 SV=1	Actins are highly conserved proteins that are involved in various types of cell motility and are ubiquitously expressed in all eukaryotic cells (375 aa)
**Q9BUJ2**	HNRL1	Heterogeneous nuclear ribonucleoprotein U-like protein 1 OS=Homo sapiens OX=9606 GN=HNRNPUL1 PE=1 SV=2	Acts as a basic transcriptional regulator. Represses basic transcription driven by several virus and cellular promoters. When associated with BRD7, activates transcription of glucocorticoid-responsive promoter in the absence of ligand- stimulation. Plays also a role in mRNA processing and transport. Binds avidly to poly(G) and poly(C) RNA homopolymers in vitro (856 aa)
**Q9BXP5**	SRRT	Serrate RNA effector molecule homolog OS=Homo sapiens OX=9606 GN=SRRT PE=1 SV=1	Acts as a mediator between the cap-binding complex (CBC) and the primary microRNAs (miRNAs) processing machinery during cell proliferation. Contributes to the stability and delivery of capped primary miRNA transcripts to the primary miRNA processing complex containing DGCR8 and DROSHA, thereby playing a role in RNA-mediated gene silencing (RNAi) by miRNAs. Binds capped RNAs (m7GpppG-capped RNA); however interaction is probably mediated via its interaction with NCBP1/CBP80 component of the CBC complex. Involved in cell cycle progression at S phase. [...] (876 aa)

**Q9BPZ3**	PAIP2	Polyadenylate-binding protein-interacting protein 2 OS=Homo sapiens OX=9606 GN=PAIP2 PE=1 SV=1	Acts as a repressor in the regulation of translation initiation of poly(A)-containing mRNAs. Its inhibitory activity on translation is mediated via its action on PABPC1. Displaces the interaction of PABPC1 with poly(A) RNA and competes with PAIP1 for binding to PABPC1. Its association with PABPC1 results in disruption of the cytoplasmic poly(A) RNP structure organization (127 aa)
**Q9P2E9**	RRBP1	Ribosome-binding protein 1 OS=Homo sapiens OX=9606 GN=RRBP1 PE=1 SV=5	Acts as a ribosome receptor and mediates interaction between the ribosome and the endoplasmic reticulum membrane (977 aa)
**O14979**	HNRDL	Heterogeneous nuclear ribonucleoprotein D-like OS=Homo sapiens OX=9606 GN=HNRNPDL PE=1 SV=3	Acts as a transcriptional regulator. Promotes transcription repression. Promotes transcription activation in differentiated myotubes (By similarity). Binds to double- and single-stranded DNA sequences. Binds to the transcription suppressor CATR sequence of the COX5B promoter (By similarity). Binds with high affinity to RNA molecules that contain AU-rich elements (AREs) found within the 3'-UTR of many proto-oncogenes and cytokine mRNAs. Binds both to nuclear and cytoplasmic poly(A) mRNAs. Binds to poly(G) and poly(A), but not to poly(U) or [...] (420 aa)
**Q8N5F7**	NKAP	NF-kappa-B-activating protein OS=Homo sapiens OX=9606 GN=NKAP PE=1 SV=1	Acts as a transcriptional repressor. Plays a role as a transcriptional corepressor of the Notch-mediated signaling required for T-cell development. Also involved in the TNF and IL-1 induced NF-kappa-B activation. Associates with chromatin at the Notch-regulated SKP2 promoter
**Q92499**	DDX1	ATP-dependent RNA helicase DDX1 OS=Homo sapiens OX=9606 GN=DDX1 PE=1 SV=2	Acts as an ATP-dependent RNA helicase, able to unwind both RNA-RNA and RNA-DNA duplexes. Possesses 5' single-stranded RNA overhang nuclease activity. Possesses ATPase activity on various RNA, but not DNA polynucleotides. May play a role in RNA clearance at DNA double-strand breaks (DSBs), thereby facilitating the template-guided repair of transcriptionally active regions of the genome. Together with RELA, acts as a coactivator to enhance NF-kappa-B-mediated transcriptional activation. Acts as a positive transcriptional regulator of cyclin CCND2 expressi [...] (740 aa)

**P36406**	TRI23	E3 ubiquitin-protein ligase TRIM23 OS=Homo sapiens OX=9606 GN=TRIM23 PE=1 SV=1	Acts as an E3 ubiquitin-protein ligase. In the presence of the human cytomegalovirus (HCMV) protein UL144, participates in 'Lys-63'-linked auto-ubiquitination of TRAF6 resulting in the virally controlled activation of NF-kappa-B at early time of infection. The C-terminus can act as an allosteric activator of the cholera toxin catalytic subunit
**O14654**	IRS4	Insulin receptor substrate 4 OS=Homo sapiens OX=9606 GN=IRS4 PE=1 SV=1	Acts as an interface between multiple growth factor receptors possessing tyrosine kinase activity, such as insulin receptor, IGF1R and FGFR1, and a complex network of intracellular signaling molecules containing SH2 domains. Involved in the IGF1R mitogenic signaling pathway. Promotes the AKT1 signaling pathway and BAD phosphorylation during insulin stimulation without activation of RPS6KB1 or the inhibition of apoptosis. Interaction with GRB2 enhances insulin-stimulated mitogen-activated protein kinase activity. May be involved in nonreceptor tyrosine kina [...] (1257 aa)
**Q99615**	DNJC7	DnaJ homolog subfamily C member 7 OS=Homo sapiens OX=9606 GN=DNAJC7 PE=1 SV=2	Acts as co-chaperone regulating the molecular chaperones HSP70 and HSP90 in folding of steroid receptors, such as the glucocorticoid receptor and the progesterone receptor. Proposed to act as a recycling chaperone by facilitating the return of chaperone substrates to early stages of chaperoning if further folding is required. In vitro, induces ATP-independent dissociation of HSP90 but not of HSP70 from the chaperone- substrate complexes. Recruits NR1I3 to the cytoplasm (By similarity)
**P25205**	MCM3	DNA replication licensing factor MCM3 OS=Homo sapiens OX=9606 GN=MCM3 PE=1 SV=3	Acts as component of the MCM2-7 complex (MCM complex) which is the putative replicative helicase essential for 'once per cell cycle' DNA replication initiation and elongation in eukaryotic cells. The active ATPase sites in the MCM2-7 ring are formed through the interaction surfaces of two neighboring subunits such that a critical structure of a conserved arginine finger motif is provided in trans relative to the ATP-binding site of the Walker A box of the adjacent subunit. The six ATPase active sites, however, are likely to contribute differential [...] (853 aa)
**Q99733**	NP1L4	Nucleosome assembly protein 1-like 4 OS=Homo sapiens OX=9606 GN=NAP1L4 PE=1 SV=1	Acts as histone chaperone in nucleosome assembly

**Q13409**	DC1I2	Cytoplasmic dynein 1 intermediate chain 2 OS=Homo sapiens OX=9606 GN=DYNC1I2 PE=1 SV=3	Acts as one of several non-catalytic accessory components of the cytoplasmic dynein 1 complex that are thought to be involved in linking dynein to cargos and to adapter proteins that regulate dynein function. Cytoplasmic dynein 1 acts as a motor for the intracellular retrograde motility of vesicles and organelles along microtubules. The intermediate chains mediate the binding of dynein to dynactin via its 150 kDa component (p150- glued) DCNT1. Involved in membrane-transport, such as Golgi apparatus, late endosomes and lysosomes (638 aa)
**Q9NP97**	DLRB1	Dynein light chain roadblock-type 1 OS=Homo sapiens OX=9606 GN=DYNLRB1 PE=1 SV=3	Acts as one of several non-catalytic accessory components of the cytoplasmic dynein 1 complex that are thought to be involved in linking dynein to cargos and to adapter proteins that regulate dynein function. Cytoplasmic dynein 1 acts as a motor for the intracellular retrograde motility of vesicles and organelles along microtubules
**Q9ULJ6**	ZMIZ1	Zinc finger MIZ domain-containing protein 1 OS=Homo sapiens OX=9606 GN=ZMIZ1 PE=1 SV=3	Acts as transcriptional coactivator. Increases ligand-dependent transcriptional activity of AR and promotes AR sumoylation. The stimulation of AR activity is dependent upon sumoylation. Involved in transcriptional activation of a subset of NOTCH1 target genes including MYC. Involved in thymocyte and T cell development (By similarity)
**Q99543**	DNJC2	DnaJ homolog subfamily C member 2 OS=Homo sapiens OX=9606 GN=DNAJC2 PE=1 SV=4	Acts both as a chaperone in the cytosol and as a chromatin regulator in the nucleus. When cytosolic, acts as a molecular chaperone- component of the ribosome-associated complex (RAC), a complex involved in folding or maintaining nascent polypeptides in a folding-competent state. In the RAC complex, stimulates the ATPase activity of the ribosome-associated pool of Hsp70-type chaperones HSPA14 that bind to the nascent polypeptide chain. When nuclear, mediates the switching from polycomb- repressed genes to an active state- specifically recruited at hist [...] (621 aa)
**Q06124**	PTN11	Tyrosine-protein phosphatase non-receptor type 11 OS=Homo sapiens OX=9606 GN=PTPN11 PE=1 SV=2	Acts downstream of various receptor and cytoplasmic protein tyrosine kinases to participate in the signal transduction from the cell surface to the nucleus. Positively regulates MAPK signal transduction pathway. Dephosphorylates GAB1, ARHGAP35 and EGFR. Dephosphorylates ROCK2 at ’Tyr-722’ resulting in stimulatation of its RhoA binding activity. Dephosphorylates CDC73

**O60610**	DIAP1	Protein diaphanous homolog 1 OS=Homo sapiens OX=9606 GN=DIAPH1 PE=1 SV=2	Acts in a Rho-dependent manner to recruit PFY1 to the membrane. Required for the assembly of F-actin structures, such as actin cables and stress fibers. Nucleates actin filaments. Binds to the barbed end of the actin filament and slows down actin polymerization and depolymerization. Required for cytokinesis, and transcriptional activation of the serum response factor. DFR proteins couple Rho and Src tyrosine kinase during signaling and the regulation of actin dynamics. Functions as a scaffold protein for MAPRE1 and APC to stabilize microtubules and promote [...] (1272 aa)
**P27348**	1433T	14-3-3 protein theta OS=Homo sapiens OX=9606 GN=YWHAQ PE=1 SV=1	Adapter protein implicated in the regulation of a large spectrum of both general and specialized signaling pathways. Binds to a large number of partners, usually by recognition of a phosphoserine or phosphothreonine motif. Binding generally results in the modulation of the activity of the binding partner. Negatively regulates the kinase activity of PDPK1
**P62258**	1433E	14-3-3 protein epsilon OS=Homo sapiens OX=9606 GN=YWHAE PE=1 SV=1	Adapter protein implicated in the regulation of a large spectrum of both general and specialized signaling pathways. Binds to a large number of partners, usually by recognition of a phosphoserine or phosphothreonine motif. Binding generally results in the modulation of the activity of the binding partner (By similarity). Positively regulates phosphorylated protein HSF1 nuclear export to the cytoplasm
**P63104**	1433Z	14-3-3 protein zeta/delta OS=Homo sapiens OX=9606 GN=YWHAZ PE=1 SV=1	Adapter protein implicated in the regulation of a large spectrum of both general and specialized signaling pathways. Binds to a large number of partners, usually by recognition of a phosphoserine or phosphothreonine motif. Binding generally results in the modulation of the activity of the binding partner
**Q8WXE9**	STON2	Stonin-2 OS=Homo sapiens OX=9606 GN=STON2 PE=1 SV=1	Adapter protein involved in endocytic machinery. Involved in the synaptic vesicle recycling. May facilitate clathrin-coated vesicle uncoating

**P22681**	CBL	E3 ubiquitin-protein ligase CBL OS=Homo sapiens OX=9606 GN=CBL PE=1 SV=2	Adapter protein that functions as a negative regulator of many signaling pathways that are triggered by activation of cell surface receptors. Acts as an E3 ubiquitin-protein ligase, which accepts ubiquitin from specific E2 ubiquitin-conjugating enzymes, and then transfers it to substrates promoting their degradation by the proteasome. Recognizes activated receptor tyrosine kinases, including KIT, FLT1, FGFR1, FGFR2, PDGFRA, PDGFRB, EGFR, CSF1R, EPHA8 and KDR and terminates signaling. Recognizes membrane-bound HCK, SRC and other kinases of the SRC family [...] (906 aa)
**P62993**	GRB2	Growth factor receptor-bound protein 2 OS=Homo sapiens OX=9606 GN=GRB2 PE=1 SV=1	Adapter protein that provides a critical link between cell surface growth factor receptors and the Ras signaling pathway
**P30419**	NMT1	Glycylpeptide N-tetradecanoyltransferase 1 OS=Homo sapiens OX=9606 GN=NMT1 PE=1 SV=2	Adds a myristoyl group to the N-terminal glycine residue of certain cellular and viral proteins (496 aa)
**Q15942**	ZYX	Zyxin OS=Homo sapiens OX=9606 GN=ZYX PE=1 SV=1	Adhesion plaque protein. Binds alpha-actinin and the CRP protein. Important for targeting TES and ENA/VASP family members to focal adhesions and for the formation of actin-rich structures. May be a component of a signal transduction pathway that mediates adhesion-stimulated changes in gene expression (By similarity) (572 aa)
**P07996**	TSP1	Thrombospondin-1 OS=Homo sapiens OX=9606 GN=THBS1 PE=1 SV=2	Adhesive glycoprotein that mediates cell-to-cell and cell-to-matrix interactions. Binds heparin. May play a role in dentinogenesis and/or maintenance of dentin and dental pulp (By similarity). Ligand for CD36 mediating antiangiogenic properties. Plays a role in ER stress response, via its interaction with the activating transcription factor 6 alpha (ATF6) which produces adaptive ER stress response factors (By similarity) (1170 aa)
**P20930**	FILA	Filaggrin OS=Homo sapiens OX=9606 GN=FLG PE=1 SV=3	Aggregates keratin intermediate filaments and promotes disulfide-bond formation among the intermediate filaments during terminal differentiation of mammalian epidermis
**P13995**	MTDC	Bifunctional methylenetetrahydrofolate dehydrogenase/cyclohydrolase, mitochondrial OS=Homo sapiens OX=9606 GN=MTHFD2 PE=1 SV=2	Although its dehydrogenase activity is NAD-specific, it can also utilize NADP at a reduced efficiency
**Q9Y285**	SYFA	Phenylalanine--tRNA ligase alpha subunit OS=Homo sapiens OX=9606 GN=FARSA PE=1 SV=3	Aminoacyl tRNA synthetases, Class II (508 aa); Diseases associated with FARSA include Myiasis and Pediculus Humanus Capitis Infestation. Among its related pathways are tRNA Aminoacylation and Gene Expression.

**Q9NSD9**	SYFB	Phenylalanine--tRNA ligase beta subunit OS=Homo sapiens OX=9606 GN=FARSB PE=1 SV=3	Aminoacyl tRNA synthetases, Class II (589 aa); Diseases associated with FARSB include Neurodevelopmental Disorder With Brain, Liver, And Lung Abnormalities and Liver Cirrhosis. Among its related pathways are tRNA Aminoacylation and Amino Acid metabolism.
**Q9BZZ5**	API5	Apoptosis inhibitor 5 OS=Homo sapiens OX=9606 GN=API5 PE=1 SV=3	Antiapoptotic factor that may have a role in protein assembly. Negatively regulates ACIN1. By binding to ACIN1, it suppresses ACIN1 cleavage from CASP3 and ACIN1-mediated DNA fragmentation. Also known to efficiently suppress E2F1-induced apoptosis. Its depletion enhances the cytotoxic action of the chemotherapeutic drugs
**Q9ULC4**	MCTS1	Malignant T-cell-amplified sequence 1 OS=Homo sapiens OX=9606 GN=MCTS1 PE=1 SV=1	Anti-oncogene that plays a role in cell cycle regulation; decreases cell doubling time and anchorage-dependent growth; shortens the duration of G1 transit time and G1/S transition. When constitutively expressed, increases CDK4 and CDK6 kinases activity and CCND1/cyclin D1 protein level, as well as G1 cyclin/CDK complex formation. Involved in translation initiation; promotes recruitment of aminoacetyled initiator tRNA to P site of 40S ribosomes. Can promote release of deacylated tRNA and mRNA from recycled 40S subunits following ABCE1-mediated diss [...] (182 aa)
**Q7Z2W4**	ZCCHV	Zinc finger CCCH-type antiviral protein 1 OS=Homo sapiens OX=9606 GN=ZC3HAV1 PE=1 SV=3	Antiviral protein which inhibits the replication of viruses by recruiting the cellular RNA degradation machineries to degrade the viral mRNAs. Binds to a ZAP-responsive element (ZRE) present in the target viral mRNA, recruits cellular poly(A)- specific ribonuclease PARN to remove the poly(A) tail, and the 3’- 5’ exoribonuclease complex exosome to degrade the RNA body from the 3’-end. It also recruits the decapping complex DCP1-DCP2 through RNA helicase p72 (DDX17) to remove the cap structure of the viral mRNA to initiate its degradation from t [...] (902 aa)
**Q12905**	ILF2	Interleukin enhancer-binding factor 2 OS=Homo sapiens OX=9606 GN=ILF2 PE=1 SV=2	Appears to function predominantly as a heterodimeric complex with ILF3. This complex may regulate transcription of the IL2 gene during T-cell activation. It can also promote the formation of stable DNA-dependent protein kinase holoenzyme complexes on DNA. Essential for the efficient reshuttling of ILF3 (isoform 1 and isoform 2) into the nucleus (390 aa)
**Q00341**	VIGLN	Vigilin OS=Homo sapiens OX=9606 GN=HDLBP PE=1 SV=2	Appears to play a role in cell sterol metabolism. It may function to protect cells from over-accumulation of cholesterol (1268 aa)

**P78344**	IF4G2	Eukaryotic translation initiation factor 4 gamma 2 OS=Homo sapiens OX=9606 GN=EIF4G2 PE=1 SV=1	Appears to play a role in the switch from cap-dependent to IRES-mediated translation during mitosis, apoptosis and viral infection. Cleaved by some caspases and viral proteases (907 aa)
**Q6ZWH5**	NEK10	Serine/threonine-protein kinase Nek10 OS=Homo sapiens OX=9606 GN=NEK10 PE=2 SV=3	Armadillo-like helical domain containing (712 aa); Gene Ontology (GO) annotations related to this gene include transferase activity, transferring phosphorus-containing groups and protein tyrosine kinase activity.
**P41091**	IF2G	Eukaryotic translation initiation factor 2 subunit 3 OS=Homo sapiens OX=9606 GN=EIF2S3 PE=1 SV=3	As a subunit of eukaryotic initiation factor 2 (eIF2), involved in the early steps of protein synthesis. In the presence of GTP, eIF2 forms a ternary complex with initiator tRNA Met-tRNAi and then recruits the 40S ribosomal complex, a step that determines the rate of protein translation. This step is followed by mRNA binding to form the 43S pre-initiation complex. Junction of the 60S ribosomal subunit to form the 80S initiation complex is preceded by hydrolysis of the GTP bound to eIF2 and release of an eIF2-GDP binary complex. In o [...] (472 aa)
**Q92841**	DDX17	Probable ATP-dependent RNA helicase DDX17 OS=Homo sapiens OX=9606 GN=DDX17 PE=1 SV=2	As an RNA helicase, unwinds RNA and alters RNA structures through ATP binding and hydrolysis. Involved in multiple cellular processes, including pre-mRNA splicing, alternative splicing, ribosomal RNA processing and miRNA processing, as well as transcription regulation. Regulates the alternative splicing of exons exhibiting specific features. For instance, promotes the inclusion of AC-rich alternative exons in CD44 transcripts. This function requires the RNA helicase activity. Affects NFAT5 and histone macro-H2A.1/H2AFY alternative splicing in [...] (731 aa)
**Q13492**	PICAL	Phosphatidylinositol-binding clathrin assembly protein OS=Homo sapiens OX=9606 GN=PICALM PE=1 SV=2	Assembly protein recruiting clathrin and adapter protein complex 2 (AP2) to cell membranes at sites of coated-pit formation and clathrin-vesicle assembly. May be required to determine the amount of membrane to be recycled, possibly by regulating the size of the clathrin cage. Involved in AP2-dependent clathrin-mediated endocytosis at the neuromuscular junction

**P40429**	RL13A	60S ribosomal protein L13a OS=Homo sapiens OX=9606 GN=RPL13A PE=1 SV=2	Associated with ribosomes but is not required for canonical ribosome function and has extra-ribosomal functions. Component of the GAIT (gamma interferon-activated inhibitor of translation) complex which mediates interferon-gamma-induced transcript-selective translation inhibition in inflammation processes. Upon interferon-gamma activation and subsequent phosphorylation dissociates from the ribosome and assembles into the GAIT complex which binds to stem loop-containing GAIT elements in the 3'-UTR of diverse inflammatory mRNAs (such as ceruplasmin) and suppre [...] (203 aa)
**P52815**	RM12	39S ribosomal protein L12, mitochondrial OS=Homo sapiens OX=9606 GN=MRPL12 PE=1 SV=2	Associates with mitochondrial RNA polymerase to activate transcription
**Q7Z478**	DHX29	ATP-dependent RNA helicase DHX29 OS=Homo sapiens OX=9606 GN=DHX29 PE=1 SV=2	ATP-binding RNA helicase involved in translation initiation. Part of the 43S pre-initiation complex that is required for efficient initiation on mRNAs of higher eukaryotes with structured 5'-UTRs by promoting efficient NTPase-dependent 48S complex formation. Specifically binds to the 40S ribosome near the mRNA entrance. Does not possess a processive helicase activity
**O14646**	CHD1	Chromodomain-helicase-DNA-binding protein 1 OS=Homo sapiens OX=9606 GN=CHD1 PE=1 SV=2	ATP-dependent chromatin-remodeling factor which functions as substrate recognition component of the transcription regulatory histone acetylation (HAT) complex SAGA. Regulates polymerase II transcription. Also required for efficient transcription by RNA polymerase I, and more specifically the polymerase I transcription termination step. Regulates negatively DNA replication. Not only involved in transcription-related chromatin-remodeling, but also required to maintain a specific chromatin configuration across the genome. Is also associated wit [...] (1710 aa)
**Q9GZR7**	DDX24	ATP-dependent RNA helicase DDX24 OS=Homo sapiens OX=9606 GN=DDX24 PE=1 SV=1	ATP-dependent RNA helicase; Gene Ontology (GO) annotations related to this gene include nucleic acid binding and ATP-dependent RNA helicase activity.
**Q9UMR2**	DD19B	ATP-dependent RNA helicase DDX19B OS=Homo sapiens OX=9606 GN=DDX19B PE=1 SV=1	ATP-dependent RNA helicase involved in mRNA export from the nucleus. Rather than unwinding RNA duplexes, DDX19B functions as a remodeler of ribonucleoprotein particles, whereby proteins bound to nuclear mRNA are dissociated and replaced by cytoplasmic mRNA binding proteins

**Q86XP3**	DDX42	ATP-dependent RNA helicase DDX42 OS=Homo sapiens OX=9606 GN=DDX42 PE=1 SV=1	ATP-dependent RNA helicase. Binds to partially double- stranded RNAs (dsRNAs) in order to unwind RNA secondary structures. Unwinding is promoted in the presence of single-strand binding proteins. Mediates also RNA duplex formation thereby displacing the single-strand RNA binding protein. ATP and ADP modulate its activity- ATP binding and hydrolysis by DDX42 triggers RNA strand separation, whereas the ADP-bound form of the protein triggers annealing of complementary RNA strands. Involved in the survival of cells by interacting with TP53BP2 and thereby c [...] (938 aa)
**P38919**	IF4A3	Eukaryotic initiation factor 4A-III OS=Homo sapiens OX=9606 GN=EIF4A3 PE=1 SV=4	ATP-dependent RNA helicase. Core component of the splicing-dependent multiprotein exon junction complex (EJC) deposited at splice junctions on mRNAs. The EJC is a dynamic structure consisting of core proteins and several peripheral nuclear and cytoplasmic associated factors that join the complex only transiently either during EJC assembly or during subsequent mRNA metabolism. The EJC marks the position of the exon-exon junction in the mature mRNA for the gene expression machinery and the core components remain bound to spliced mRNAs throughout all s [...] (411 aa)
**O43776**	SYNC	Asparagine--tRNA ligase, cytoplasmic OS=Homo sapiens OX=9606 GN=NARS PE=1 SV=1	Atypical type III intermediate filament (IF) protein that may play a supportive role in the efficient coupling of mechanical stress between the myofibril and fiber exterior. May facilitate lateral force transmission during skeletal muscle contraction. Does not form homofilaments nor heterofilaments with other IF proteins (482 aa)
**Q9UIG0**	BAZ1B	Tyrosine-protein kinase BAZ1B OS=Homo sapiens OX=9606 GN=BAZ1B PE=1 SV=2	Atypical tyrosine-protein kinase that plays a central role in chromatin remodeling and acts as a transcription regulator. Involved in DNA damage response by phosphorylating 'Tyr-142' of histone H2AX (H2AXY142ph). H2AXY142ph plays a central role in DNA repair and acts as a mark that distinguishes between apoptotic and repair responses to genotoxic stress. Essential component of the WICH complex, a chromatin remodeling complex that mobilizes nucleosomes and reconfigures irregular chromatin to a regular nucleosomal array structure. The WICH complex regulates [...] (1483 aa)

**Q5VZE5**	NAA35	N-alpha-acetyltransferase 35, NatC auxiliary subunit OS=Homo sapiens OX=9606 GN=NAA35 PE=1 SV=1	Auxillary component of the N-terminal acetyltransferase C (NatC) complex which catalyzes acetylation of N-terminal methionine residues. Involved in regulation of apoptosis and proliferation of smooth muscle cells
**Q9BXJ9**	NAA15	N-alpha-acetyltransferase 15, NatA auxiliary subunit OS=Homo sapiens OX=9606 GN=NAA15 PE=1 SV=1	Auxillary subunit of the N-terminal acetyltransferase A (NatA) complex which displays alpha (N-terminal) acetyltransferase activity. The NAT activity may be important for vascular, hematopoietic and neuronal growth and development. Required to control retinal neovascularization in adult ocular endothelial cells. In complex with XRCC6 and XRCC5 (Ku80), up-regulates transcription from the osteocalcin promoter
**Q86WR0**	CCD25	Coiled-coil domain-containing protein 25 OS=Homo sapiens OX=9606 GN=CCDC25 PE=1 SV=2	Belongs to the CCDC25 family (208 aa); Diseases associated with CCDC25 include Phocomelia and Sc Phocomelia Syndrome.
**Q4VC31**	CCD58	Coiled-coil domain-containing protein 58 OS=Homo sapiens OX=9606 GN=CCDC58 PE=1 SV=1	Belongs to the CCDC58 family (144 aa); Diseases associated with CCDC58 include Ehrlichiosis.
**P84098**	RL19	60S ribosomal protein L19 OS=Homo sapiens OX=9606 GN=RPL19 PE=1 SV=1	Belongs to the eukaryotic ribosomal protein eL19 family (196 aa); Among its related pathways are Viral mRNA Translation and Influenza Viral RNA Transcription and Replication.
**P35268**	RL22	60S ribosomal protein L22 OS=Homo sapiens OX=9606 GN=RPL22 PE=1 SV=2	Belongs to the eukaryotic ribosomal protein eL22 family (128 aa); Among its related pathways are Viral mRNA Translation and Influenza Viral RNA Transcription and Replication.
**P83731**	RL24	60S ribosomal protein L24 OS=Homo sapiens OX=9606 GN=RPL24 PE=1 SV=1	Belongs to the eukaryotic ribosomal protein eL24 family (157 aa); Diseases associated with RPL24 include Bone Resorption Disease and Bone Remodeling Disease. Among its related pathways are Viral mRNA Translation and Influenza Viral RNA Transcription and Replication.
**P62888**	RL30	60S ribosomal protein L30 OS=Homo sapiens OX=9606 GN=RPL30 PE=1 SV=2	Belongs to the eukaryotic ribosomal protein eL30 family (115 aa); Among its related pathways are Viral mRNA Translation and Influenza Viral RNA Transcription and Replication.
**P62910**	RL32	60S ribosomal protein L32 OS=Homo sapiens OX=9606 GN=RPL32 PE=1 SV=2	Belongs to the eukaryotic ribosomal protein eL32 family (135 aa); Diseases associated with RPL32 include Melanoma, Cutaneous Malignant 1. Among its related pathways are Viral mRNA Translation and Influenza Viral RNA Transcription and Replication.

**P63173**	RL38	60S ribosomal protein L38 OS=Homo sapiens OX=9606 GN=RPL38 PE=1 SV=2	Belongs to the eukaryotic ribosomal protein eL38 family (70 aa); Diseases associated with RPL38 include Aromatic L-Amino Acid Decarboxylase Deficiency. Among its related pathways are Viral mRNA Translation and Influenza Viral RNA Transcription and Replication.
**P63220**	RS21	40S ribosomal protein S21 OS=Homo sapiens OX=9606 GN=RPS21 PE=1 SV=1	Belongs to the eukaryotic ribosomal protein eS21 family (83 aa); Among its related pathways are Activation of the mRNA upon binding of the cap-binding complex and eIFs, and subsequent binding to 43S and Viral mRNA Translation.
**Q71UM5**	RS27L	40S ribosomal protein S27-like OS=Homo sapiens OX=9606 GN=RPS27L PE=1 SV=3	Belongs to the eukaryotic ribosomal protein eS27 family (84 aa); Diseases associated with RPS27L include Shwachman-Diamond Syndrome 1. Among its related pathways are Activation of the mRNA upon binding of the cap-binding complex and eIFs, and subsequent binding to 43S and Viral mRNA Translation.
**P62857**	RS28	40S ribosomal protein S28 OS=Homo sapiens OX=9606 GN=RPS28 PE=1 SV=1	Belongs to the eukaryotic ribosomal protein eS28 family (69 aa); Diseases associated with RPS28 include Diamond-Blackfan Anemia 15 With Mandibulofacial Dysostosis and Diamond-Blackfan Anemia. Among its related pathways are Activation of the mRNA upon binding of the cap-binding complex and eIFs, and subsequent binding to 43S and Viral mRNA Translation.
**P62701**	RS4X	40S ribosomal protein S4, X isoform OS=Homo sapiens OX=9606 GN=RPS4X PE=1 SV=2	Belongs to the eukaryotic ribosomal protein eS4 family (263 aa); Diseases associated with RPS4X include Turner Syndrome. Among its related pathways are Activation of the mRNA upon binding of the cap-binding complex and eIFs, and subsequent binding to 43S and Viral mRNA Translation.
**P62241**	RS8	40S ribosomal protein S8 OS=Homo sapiens OX=9606 GN=RPS8 PE=1 SV=2	Belongs to the eukaryotic ribosomal protein eS8 family (208 aa); Among its related pathways are Activation of the mRNA upon binding of the cap-binding complex and eIFs, and subsequent binding to 43S and Viral mRNA Translation.
**Q8N9T8**	KRI1	Protein KRI1 homolog OS=Homo sapiens OX=9606 GN=KRI1 PE=1 SV=3	This gene overlaps with the gene for cysteine endopeptidase AUT-like 4 in a head-to-tail orientation.
**Q3KQU3**	MA7D1	MAP7 domain-containing protein 1 OS=Homo sapiens OX=9606 GN=MAP7D1 PE=1 SV=1	MAP7D1 (MAP7 Domain Containing 1) is a Protein Coding gene. Gene Ontology (GO) annotations related to this gene include structural molecule activity. An important paralog of this gene is MAP7.

**Q9NQ50**	RM40	39S ribosomal protein L40, mitochondrial OS=Homo sapiens OX=9606 GN=MRPL40 PE=1 SV=1	Mammalian mitochondrial ribosomal proteins are encoded by nuclear genes and help in protein synthesis within the mitochondrion. Mitochondrial ribosomes (mitoribosomes) consist of a small 28S subunit and a large 39S subunit. They have an estimated 75% protein to rRNA composition compared to prokaryotic ribosomes, where this ratio is reversed. Another difference between mammalian mitoribosomes and prokaryotic ribosomes is that the latter contain a 5S rRNA. Among different species, the proteins comprising the mitoribosome differ greatly in sequence, and sometimes in biochemical properties, which prevents easy recognition by sequence homology. This gene encodes a 39S subunit protein. Deletions in this gene may contribute to the etiology of velo-cardio-facial syndrome and DiGeorge syndrome.
**Q96K17**	BT3L4	Transcription factor BTF3 homolog 4 OS=Homo sapiens OX=9606 GN=BTF3L4 PE=1 SV=1	BTF3L4 (Basic Transcription Factor 3 Like 4) is a Protein Coding gene.
**Q6GPI1**	CTRB2	Chymotrypsinogen B2 OS=Homo sapiens OX=9606 GN=CTRB2 PE=2 SV=2	Belongs to the peptidase S1 family (263 aa); Among its related pathways are Metabolism of water-soluble vitamins and cofactors and Cobalamin (Cbl, vitamin B12) transport and metabolism.
**P12273**	PIP	Prolactin-inducible protein OS=Homo sapiens OX=9606 GN=PIP PE=1 SV=1	Belongs to the PIP family (146 aa); Diseases associated with PIP include Microglandular Adenosis and Breast Cyst. Among its related pathways are PEDF Induced Signaling and Transport of glucose and other sugars, bile salts and organic acids, metal ions and amine compounds.
**Q8NDZ6**	T161B	Transmembrane protein 161B OS=Homo sapiens OX=9606 GN=TMEM161B PE=2 SV=1	Belongs to the TMEM161 family (502 aa); TMEM161B (Transmembrane Protein 161B) is a Protein Coding gene. Gene Ontology (GO) annotations related to this gene include nucleic acid binding. An important paralog of this gene is TMEM161A.
**P46776**	RL27A	60S ribosomal protein L27a OS=Homo sapiens OX=9606 GN=RPL27A PE=1 SV=2	Belongs to the universal ribosomal protein uL15 family (148 aa); Among its related pathways are Viral mRNA Translation and Influenza Viral RNA Transcription and Replication.
**P62280**	RS11	40S ribosomal protein S11 OS=Homo sapiens OX=9606 GN=RPS11 PE=1 SV=3	Belongs to the universal ribosomal protein uS17 family (158 aa); Among its related pathways are Activation of the mRNA upon binding of the cap-binding complex and eIFs, and subsequent binding to 43S and Viral mRNA Translation.

**P46782**	RS5	40S ribosomal protein S5 OS=Homo sapiens OX=9606 GN=RPS5 PE=1 SV=4	Belongs to the universal ribosomal protein uS7 family (204 aa); Diseases associated with RPS5 include Retinitis Pigmentosa 14 and Chromosome 2P16.1-P15 Deletion Syndrome. Among its related pathways are Activation of the mRNA upon binding of the cap-binding complex and eIFs, and subsequent binding to 43S and Viral mRNA Translation.
**P62244**	RS15A	40S ribosomal protein S15a OS=Homo sapiens OX=9606 GN=RPS15A PE=1 SV=2	Belongs to the universal ribosomal protein uS8 family (130 aa); Diseases associated with RPS15A include Diamond-Blackfan Anemia. Among its related pathways are Activation of the mRNA upon binding of the cap-binding complex and eIFs, and subsequent binding to 43S and Viral mRNA Translation.
**O60942**	MCE1	mRNA-capping enzyme OS=Homo sapiens OX=9606 GN=RNGTT PE=1 SV=1	Bifunctional mRNA-capping enzyme exhibiting RNA 5'-triphosphatase activity in the N-terminal part and mRNA guanylyltransferase activity in the C-terminal part. Catalyzes the first two steps of cap formation-by removing the gamma-phosphate from the 5'-triphosphate end of nascent mRNA to yield a diphosphate end, and by transferring the gmp moiety of GTP to the 5'-diphosphate terminus (597 aa)
**Q9BTD8**	RBM42	RNA-binding protein 42 OS=Homo sapiens OX=9606 GN=RBM42 PE=1 SV=1	Binds (via the RRM domain) to the 3'-untranslated region (UTR) of CDKN1A mRNA
**P35637**	FUS	RNA-binding protein FUS OS=Homo sapiens OX=9606 GN=FUS PE=1 SV=1	Binds both single-stranded and double-stranded DNA and promotes ATP-independent annealing of complementary single- stranded DNAs and D-loop formation in superhelical double-stranded DNA. May play a role in maintenance of genomic integrity
**P82979**	SARNP	SAP domain-containing ribonucleoprotein OS=Homo sapiens OX=9606 GN=SARNP PE=1 SV=3	Binds both single-stranded and double-stranded DNA with higher affinity for the single-stranded form. Specifically binds to scaffold/matrix attachment region DNA. Also binds single- stranded RNA. Enhances RNA unwinding activity of DDX39A. May participate in important transcriptional or translational control of cell growth, metabolism and carcinogenesis. Component of the TREX complex which is thought to couple mRNA transcription, processing and nuclear export, and specifically associates with spliced mRNA and not with unspliced pre-mRNA. TREX is [...] (210 aa)
**P30050**	RL12	60S ribosomal protein L12 OS=Homo sapiens OX=9606 GN=RPL12 PE=1 SV=1	Binds directly to 26S ribosomal RNA (165 aa); Among its related pathways are Viral mRNA Translation and Influenza Viral RNA Transcription and Replication.

**O95793**	STAU1	Double-stranded RNA-binding protein Staufen homolog 1 OS=Homo sapiens OX=9606 GN=STAU1 PE=1 SV=2	Binds double-stranded RNA (regardless of the sequence) and tubulin. May play a role in specific positioning of mRNAs at given sites in the cell by cross-linking cytoskeletal and RNA components, and in stimulating their translation at the site
**P07910**	HNRPC	Heterogeneous nuclear ribonucleoproteins C1/C2 OS=Homo sapiens OX=9606 GN=HNRNPC PE=1 SV=4	Binds pre-mRNA and nucleates the assembly of 40S hnRNP particles. Interacts with poly-U tracts in the 3'-UTR or 5'-UTR of mRNA and modulates the stability and the level of translation of bound mRNA molecules. Single HNRNPC tetramers bind 230-240 nucleotides. Trimers of HNRNPC tetramers bind 700 nucleotides. May play a role in the early steps of spliceosome assembly and pre-mRNA splicing. N6-methyladenosine (m6A) has been shown to alter the local structure in mRNAs and long non-coding RNAs (lncRNAs) via a mechanism named 'm(6)A-switch', f [...] (306 aa)
**Q99729**	ROAA	Heterogeneous nuclear ribonucleoprotein A/B OS=Homo sapiens OX=9606 GN=HNRNPAB PE=1 SV=2	Binds single-stranded RNA. Has a high affinity for G-rich and U-rich regions of hnRNA. Also binds to APOB mRNA transcripts around the RNA editing site
**Q9Y4Z0**	LSM4	U6 snRNA-associated Sm-like protein LSm4 OS=Homo sapiens OX=9606 GN=LSM4 PE=1 SV=1	Binds specifically to the 3'-terminal U-tract of U6 snRNA
**Q9Y333**	LSM2	U6 snRNA-associated Sm-like protein LSm2 OS=Homo sapiens OX=9606 GN=LSM2 PE=1 SV=1	Binds specifically to the 3'-terminal U-tract of U6 snRNA. May be involved in pre-mRNA splicing
**Q9H361**	PABP3	Polyadenylate-binding protein 3 OS=Homo sapiens OX=9606 GN=PABPC3 PE=1 SV=2	Binds the poly(A) tail of mRNA. May be involved in cytoplasmic regulatory processes of mRNA metabolism. Binds poly(A) with a slightly lower affinity as compared to PABPC1
**Q13310**	PABP4	Polyadenylate-binding protein 4 OS=Homo sapiens OX=9606 GN=PABPC4 PE=1 SV=1	Binds the poly(A) tail of mRNA. May be involved in cytoplasmic regulatory processes of mRNA metabolism. Can probably bind to cytoplasmic RNA sequences other than poly(A) in vivo (By similarity)
**Q15554**	TERF2	Telomeric repeat-binding factor 2 OS=Homo sapiens OX=9606 GN=TERF2 PE=1 SV=3	Binds the telomeric double-stranded 5'-TTAGGG-3' repeat and plays a central role in telomere maintenance and protection against end-to-end fusion of chromosomes. In addition to its telomeric DNA-binding role, required to recruit a number of factors and enzymes required for telomere protection, including the shelterin complex, TERF2IP/RAP1 and DCLRE1B/Apollo. Component of the shelterin complex (telosome) that is involved in the regulation of telomere length and protection. Shelterin associates with arrays of double-stranded 5'-TTAGGG-3' repeats added b [...] (542 aa)

**P06753**	TPM3	Tropomyosin alpha-3 chain OS=Homo sapiens OX=9606 GN=TPM3 PE=1 SV=2	Binds to actin filaments in muscle and non-muscle cells. Plays a central role, in association with the troponin complex, in the calcium dependent regulation of vertebrate striated muscle contraction. Smooth muscle contraction is regulated by interaction with caldesmon. In non-muscle cells is implicated in stabilizing cytoskeleton actin filaments
**Q9UHB6**	LIMA1	LIM domain and actin-binding protein 1 OS=Homo sapiens OX=9606 GN=LIMA1 PE=1 SV=1	Binds to actin monomers and filaments. Increases the number and size of actin stress fibers and inhibits membrane ruffling. Inhibits actin filament depolymerization. Bundles actin filaments, delays filament nucleation and reduces formation of branched filaments
**Q7Z7A4**	PXK	PX domain-containing protein kinase-like protein OS=Homo sapiens OX=9606 GN=PXK PE=1 SV=1	Binds to and modulates brain Na,K-ATPase subunits ATP1B1 and ATP1B3 and may thereby participate in the regulation of electrical excitability and synaptic transmission. May not display kinase activity (578 aa)
**Q9Y4D1**	DAAM1	Disheveled-associated activator of morphogenesis 1 OS=Homo sapiens OX=9606 GN=DAAM1 PE=1 SV=2	Binds to disheveled (Dvl) and Rho, and mediates Wnt-induced Dvl-Rho complex formation. May play a role as a scaffolding protein to recruit Rho-GDP and Rho-GEF, thereby enhancing Rho-GTP formation. Can direct nucleation and elongation of new actin filaments. Involved in building functional cilia. Involved in the organization of the subapical actin network in multiciliated epithelial cells (By similarity)
**P23528**	COF1	Cofilin-1 OS=Homo sapiens OX=9606 GN=CFL1 PE=1 SV=3	Binds to F-actin and exhibits pH-sensitive F-actin depolymerizing activity. Regulates actin cytoskeleton dynamics. Important for normal progress through mitosis and normal cytokinesis. Plays a role in the regulation of cell morphology and cytoskeletal organization. Required for the up-regulation of atypical chemokine receptor ACKR2 from endosomal compartment to cell membrane, increasing its efficiency in chemokine uptake and degradation. Required for neural tube morphogenesis and neural crest cell migration (By similarity) (166 aa)

**P56537**	IF6	Eukaryotic translation initiation factor 6 OS=Homo sapiens OX=9606 GN=EIF6 PE=1 SV=1	Binds to the 60S ribosomal subunit and prevents its association with the 40S ribosomal subunit to form the 80S initiation complex in the cytoplasm. Behaves as a stimulatory translation initiation factor downstream insulin/growth factors. Is also involved in ribosome biogenesis. Associates with pre-60S subunits in the nucleus and is involved in its nuclear export. Cytoplasmic release of TIF6 from 60S subunits and nuclear relocalization is promoted by a RACK1 (RACK1)-dependent protein kinase C activity. In tissues responsive to insulin, control [...] (245 aa)
**Q92945**	FUBP2	Far upstream element-binding protein 2 OS=Homo sapiens OX=9606 GN=KHSRP PE=1 SV=4	Binds to the dendritic targeting element and may play a role in mRNA trafficking (By similarity). Part of a ternary complex that binds to the downstream control sequence (DCS) of the pre-mRNA. Mediates exon inclusion in transcripts that are subject to tissue-specific alternative splicing. May interact with single-stranded DNA from the far-upstream element (FUSE). May activate gene expression. Also involved in degradation of inherently unstable mRNAs that contain AU-rich elements (AREs) in their 3’- UTR, possibly by recruiting degradation machine [...] (711 aa)
**P16989**	YBOX3	Y-box-binding protein 3 OS=Homo sapiens OX=9606 GN=YBX3 PE=1 SV=4	Binds to the GM-CSF promoter. Seems to act as a repressor. Binds also to full-length mRNA and to short RNA sequences containing the consensus site 5’-UCCAUCA-3’. May have a role in translation repression (By similarity)
**P05204**	HMGN2	Non-histone chromosomal protein HMG-17 OS=Homo sapiens OX=9606 GN=HMGN2 PE=1 SV=3	Binds to the inner side of the nucleosomal DNA thus altering the interaction between the DNA and the histone octamer. May be involved in the process which maintains transcribable genes in a unique chromatin conformation (By similarity)
**P05114**	HMGN1	Non-histone chromosomal protein HMG-14 OS=Homo sapiens OX=9606 GN=HMGN1 PE=1 SV=3	Binds to the inner side of the nucleosomal DNA thus altering the interaction between the DNA and the histone octamer. May be involved in the process which maintains transcribable genes in a unique chromatin conformation. Inhibits the phosphorylation of nucleosomal histones H3 and H2A by RPS6KA5/MSK1 and RPS6KA3/RSK2 (By similarity)

**Q00059**	TFAM	Transcription factor A, mitochondrial OS=Homo sapiens OX=9606 GN=TFAM PE=1 SV=1	Binds to the mitochondrial light strand promoter and functions in mitochondrial transcription regulation. Required for accurate and efficient promoter recognition by the mitochondrial RNA polymerase. Promotes transcription initiation from the HSP1 and the light strand promoter by binding immediately upstream of transcriptional start sites. Is able to unwind DNA. Bends the mitochondrial light strand promoter DNA into a U-turn shape via its HMG boxes. Required for maintenance of normal levels of mitochondrial DNA. May play a role in organizing and c [...] (246 aa)
**Q14008**	CKAP5	Cytoskeleton-associated protein 5 OS=Homo sapiens OX=9606 GN=CKAP5 PE=1 SV=3	Binds to the plus end of microtubules and regulates microtubule dynamics and microtubule organization. Acts as processive microtubule polymerase. Promotes cytoplasmic microtubule nucleation and elongation. Plays a major role in organizing spindle poles. In spindle formation protects kinetochore microtubules from depolymerization by KIF2C and has an essential role in centrosomal microtubule assembly independently of KIF2C activity. Contributes to centrosome integrity. Acts as component of the TACC3/ch-TOG/clathrin complex proposed to contribute to stab [...] (2032 aa)
**Q9P270**	SLAI2	SLAIN motif-containing protein 2 OS=Homo sapiens OX=9606 GN=SLAIN2 PE=1 SV=2	Binds to the plus end of microtubules and regulates microtubule dynamics and microtubule organization. Promotes cytoplasmic microtubule nucleation and elongation. Required for normal structure of the microtubule cytoskeleton during interphase (581 aa)
**Q14103**	HNRPD	Heterogeneous nuclear ribonucleoprotein D0 OS=Homo sapiens OX=9606 GN=HNRNPD PE=1 SV=1	Binds with high affinity to RNA molecules that contain AU-rich elements (AREs) found within the 3'-UTR of many proto- oncogenes and cytokine mRNAs. Also binds to double- and single- stranded DNA sequences in a specific manner and functions a transcription factor. Each of the RNA-binding domains specifically can bind solely to a single-stranded non-monotonous 5'-UUAG-3' sequence and also weaker to the single-stranded 5'-TTAGGG-3' telomeric DNA repeat. Binds RNA oligonucleotides with 5'-UUAGGG-3' repeats more tightly than the telomeric single-s [...] (355 aa)

**Q96RQ3**	MCCA	Methylcrotonoyl-CoA carboxylase subunit alpha, mitochondrial OS=Homo sapiens OX=9606 GN=MCCC1 PE=1 SV=3	Biotin-attachment subunit of the 3-methylcrotonyl-CoA carboxylase, an enzyme that catalyzes the conversion of 3- methylcrotonyl-CoA to 3-methylglutaconyl-CoA, a critical step for leucine and isovaleric acid catabolism (725 aa)
**O60759**	CYTIP	Cytohesin-interacting protein OS=Homo sapiens OX=9606 GN=CYTIP PE=1 SV=2	By its binding to cytohesin-1 (CYTH1), it modifies activation of ARFs by CYTH1 and its precise function may be to sequester CYTH1 in the cytoplasm
**P07355**	ANXA2	Annexin A2 OS=Homo sapiens OX=9606 GN=ANXA2 PE=1 SV=2	Calcium-regulated membrane-binding protein whose affinity for calcium is greatly enhanced by anionic phospholipids. It binds two calcium ions with high affinity. May be involved in heat-stress response. Inhibits PCSK9-enhanced LDLR degradation, probably reduces PCSK9 protein levels via a translational mechanism but also competes with LDLR for binding with PCSK9
**P06396**	GELS	Gelsolin OS=Homo sapiens OX=9606 GN=GSN PE=1 SV=1	Calcium-regulated, actin-modulating protein that binds to the plus (or barbed) ends of actin monomers or filaments, preventing monomer exchange (end-blocking or capping). It can promote the assembly of monomers into filaments (nucleation) as well as sever filaments already formed. Plays a role in ciliogenesis
**Q9HCC0**	MCCB	Methylcrotonoyl-CoA carboxylase beta chain, mitochondrial OS=Homo sapiens OX=9606 GN=MCCC2 PE=1 SV=1	Carboxyltransferase subunit of the 3-methylcrotonyl-CoA carboxylase, an enzyme that catalyzes the conversion of 3- methylcrotonyl-CoA to 3-methylglutaconyl-CoA, a critical step for leucine and isovaleric acid catabolism
**E9PAV3**	NACAM	Nascent polypeptide-associated complex subunit alpha, muscle-specific form OS=Homo sapiens OX=9606 GN=NACA PE=1 SV=1	Cardiac- and muscle-specific transcription factor. May act to regulate the expression of genes involved in the development of myotubes. Plays a critical role in ventricular cardiomyocyte expansion and regulates postnatal skeletal muscle growth and regeneration. Involved in the organized assembly of thick and thin filaments of myofibril sarcomeres (By similarity) (925 aa)
**P43155**	CACP	Carnitine O-acetyltransferase OS=Homo sapiens OX=9606 GN=CRAT PE=1 SV=5	Carnitine acetylase is specific for short chain fatty acids. Carnitine acetylase seems to affect the flux through the pyruvate dehydrogenase complex. It may be involved as well in the transport of acetyl-CoA into mitochondria
**O14561**	ACPM	Acyl carrier protein, mitochondrial OS=Homo sapiens OX=9606 GN=NDUFAB1 PE=1 SV=3	Carrier of the growing fatty acid chain in fatty acid biosynthesis (By similarity). Accessory and non-catalytic subunit of the mitochondrial membrane respiratory chain NADH dehydrogenase (Complex I), which functions in the transfer of electrons from NADH to the respiratory chain

**O75792**	RNH2A	Ribonuclease H2 subunit A OS=Homo sapiens OX=9606 GN=RNASEH2A PE=1 SV=2	Catalytic subunit of RNase HII, an endonuclease that specifically degrades the RNA of RNA-DNA hybrids. Participates in DNA replication, possibly by mediating the removal of lagging- strand Okazaki fragment RNA primers during DNA replication. Mediates the excision of single ribonucleotides from DNA-RNA duplexes
**P41227**	NAA10	N-alpha-acetyltransferase 10 OS=Homo sapiens OX=9606 GN=NAA10 PE=1 SV=1	Catalytic subunit of the N-terminal acetyltransferase A (NatA) complex which displays alpha (N-terminal) acetyltransferase activity. Acetylates amino termini that are devoid of initiator methionine. The alpha (N-terminal) acetyltransferase activity may be important for vascular, hematopoietic and neuronal growth and development. Without NAA15, displays epsilon (internal) acetyltransferase activity towards HIF1A, thereby promoting its degradation. Represses MYLK kinase activity by acetylation, and thus represses tumor cell migration. Acetylates, and stabili [...] (235 aa)
**Q9Y3I0**	RTCB	tRNA-splicing ligase RtcB homolog OS=Homo sapiens OX=9606 GN=RTCB PE=1 SV=1	Catalytic subunit of the tRNA-splicing ligase complex that acts by directly joining spliced tRNA halves to mature-sized tRNAs by incorporating the precursor-derived splice junction phosphate into the mature tRNA as a canonical 3’,5’- phosphodiester. May act as an RNA ligase with broad substrate specificity, and may function toward other RNAs (505 aa)
**P49591**	SYSC	Serine--tRNA ligase, cytoplasmic OS=Homo sapiens OX=9606 GN=SARS PE=1 SV=3	Catalyzes the attachment of serine to tRNA(Ser) in a two-step reaction- serine is first activated by ATP to form Ser- AMP and then transferred to the acceptor end of tRNA(Ser). Is probably also able to aminoacylate tRNA(Sec) with serine, to form the misacylated tRNA L-seryl-tRNA(Sec), which will be further converted into selenocysteinyl-tRNA(Sec). In the nucleus, binds to the VEGFA core promoter and prevents MYC binding and transcriptional activation by MYC. Recruits SIRT2 to the VEGFA promoter, promoting deacetylation of histone H4 at ’Lys-16’ (H4K16) [...] (514 aa)

**P07814**	SYEP	Bifunctional glutamate/proline--tRNA ligase OS=Homo sapiens OX=9606 GN=EPRS PE=1 SV=5	Catalyzes the attachment of the cognate amino acid to the corresponding tRNA in a two-step reaction- the amino acid is first activated by ATP to form a covalent intermediate with AMP and is then transferred to the acceptor end of the cognate tRNA. Component of the GAIT (gamma interferon-activated inhibitor of translation) complex which mediates interferon-gamma-induced transcript- selective translation inhibition in inflammation processes. Upon interferon-gamma activation and subsequent phosphorylation dissociates from the multisynthetase co [...] (1512 aa)
**P54577**	SYYC	Tyrosine--tRNA ligase, cytoplasmic OS=Homo sapiens OX=9606 GN=YARS PE=1 SV=4	Catalyzes the attachment of tyrosine to tRNA(Tyr) in a two-step reaction- tyrosine is first activated by ATP to form Tyr- AMP and then transferred to the acceptor end of tRNA(Tyr)
**Q04760**	LGUL	Lactoylglutathione lyase OS=Homo sapiens OX=9606 GN=GLO1 PE=1 SV=4	Catalyzes the conversion of hemimercaptal, formed from methylglyoxal and glutathione, to S-lactoylglutathione. Involved in the regulation of TNF-induced transcriptional activity of NF- kappa-B. Required for normal osteoclastogenesis (184 aa)
**P12268**	IMDH2	Inosine-5'-monophosphate dehydrogenase 2 OS=Homo sapiens OX=9606 GN=IMPDH2 PE=1 SV=2	Catalyzes the conversion of inosine 5’-phosphate (IMP) to xanthosine 5’-phosphate (XMP), the first committed and rate- limiting step in the de novo synthesis of guanine nucleotides, and therefore plays an important role in the regulation of cell growth. Could also have a single-stranded nucleic acid-binding activity and could play a role in RNA and/or DNA metabolism. It may also have a role in the development of malignancy and the growth progression of some tumors (514 aa)
**P05141**	ADT2	ADP/ATP translocase 2 OS=Homo sapiens OX=9606 GN=SLC25A5 PE=1 SV=7	Catalyzes the exchange of cytoplasmic ADP with mitochondrial ATP across the mitochondrial inner membrane. As part of the mitotic spindle-associated MMXD complex it may play a role in chromosome segregation
**P13639**	EF2	Elongation factor 2 OS=Homo sapiens OX=9606 GN=EEF2 PE=1 SV=4	Catalyzes the GTP-dependent ribosomal translocation step during translation elongation. During this step, the ribosome changes from the pre-translocational (PRE) to the post- translocational (POST) state as the newly formed A-site-bound peptidyl-tRNA and P-site-bound deacylated tRNA move to the P and E sites, respectively. Catalyzes the coordinated movement of the two tRNA molecules, the mRNA and conformational changes in the ribosome

**P55265**	DSRAD	Double-stranded RNA-specific adenosine deaminase OS=Homo sapiens OX=9606 GN=ADAR PE=1 SV=4	Catalyzes the hydrolytic deamination of adenosine to inosine in double-stranded RNA (dsRNA) referred to as A-to-I RNA editing. This may affect gene expression and function in a number of ways that include mRNA translation by changing codons and hence the amino acid sequence of proteins
**P00505**	AATM	Aspartate aminotransferase, mitochondrial OS=Homo sapiens OX=9606 GN=GOT2 PE=1 SV=3	Catalyzes the irreversible transamination of the L-tryptophan metabolite L-kynurenine to form kynurenic acid (KA). Plays a key role in amino acid metabolism. Important for metabolite exchange between mitochondria and cytosol. Facilitates cellular uptake of long-chain free fatty acids
**P22061**	PIMT	Protein-L-isoaspartate(D-aspartate) O-methyltransferase OS=Homo sapiens OX=9606 GN=PCMT1 PE=1 SV=4	Catalyzes the methyl esterification of L-isoaspartyl and D-aspartyl residues in peptides and proteins that result from spontaneous decomposition of normal L-aspartyl and L-asparaginyl residues. It plays a role in the repair and/or degradation of damaged proteins. Acts on EIF4EBP2, microtubule-associated protein 2, calreticulin, clathrin light chains a and b, Ubiquitin carboxyl-terminal hydrolase isozyme L1, phosphatidylethanolamine- binding protein 1, stathmin, beta-synuclein and alpha-synuclein
**Q13085**	ACACA	Acetyl-CoA carboxylase 1 OS=Homo sapiens OX=9606 GN=ACACA PE=1 SV=2	Catalyzes the rate-limiting reaction in the biogenesis of long-chain fatty acids. Carries out three functions- biotin carboxyl carrier protein, biotin carboxylase and carboxyltransferase (2383 aa)
**P14868**	SYDC	Aspartate--tRNA ligase, cytoplasmic OS=Homo sapiens OX=9606 GN=DARS PE=1 SV=2	Catalyzes the specific attachment of an amino acid to its cognate tRNA in a 2 step reaction- the amino acid (AA) is first activated by ATP to form AA-AMP and then transferred to the acceptor end of the tRNA
**P41252**	SYIC	Isoleucine--tRNA ligase, cytoplasmic OS=Homo sapiens OX=9606 GN=IARS PE=1 SV=2	Catalyzes the specific attachment of an amino acid to its cognate tRNA in a 2 step reaction- the amino acid (AA) is first activated by ATP to form AA-AMP and then transferred to the acceptor end of the tRNA
**P56192**	SYMC	Methionine--tRNA ligase, cytoplasmic OS=Homo sapiens OX=9606 GN=MARS PE=1 SV=2	Catalyzes the specific attachment of an amino acid to its cognate tRNA in a 2 step reaction- the amino acid (AA) is first activated by ATP to form AA-AMP and then transferred to the acceptor end of the tRNA
**Q9P2J5**	SYLC	Leucine--tRNA ligase, cytoplasmic OS=Homo sapiens OX=9606 GN=LARS PE=1 SV=2	Catalyzes the specific attachment of an amino acid to its cognate tRNA in a two step reaction- the amino acid (AA) is first activated by ATP to form AA-AMP and then transferred to the acceptor end of the tRNA. Exhibits a post-transfer editing activity to hydrolyze mischarged tRNAs

**Q9BTN0**	LRFN3	Leucine-rich repeat and fibronectin type-III domain-containing protein 3 OS=Homo sapiens OX=9606 GN=LRFN3 PE=2 SV=1	Cell adhesion molecule that mediates homophilic cell-cell adhesion in a Ca(2+)-independent manner. Promotes neurite outgrowth in hippocampal neurons (By similarity)
**P35579**	MYH9	Myosin-9 OS=Homo sapiens OX=9606 GN=MYH9 PE=1 SV=4	Cellular myosin that appears to play a role in cytokinesis, cell shape, and specialized functions such as secretion and capping. During cell spreading, plays an important role in cytoskeleton reorganization, focal contacts formation (in the margins but not the central part of spreading cells), and lamellipodial retraction
**P35580**	MYH10	Myosin-10 OS=Homo sapiens OX=9606 GN=MYH10 PE=1 SV=3	Cellular myosin that appears to play a role in cytokinesis, cell shape, and specialized functions such as secretion and capping. Involved with LARP6 in the stabilization of type I collagen mRNAs for CO1A1 and CO1A2. During cell spreading, plays an important role in cytoskeleton reorganization, focal contacts formation (in the central part but not the margins of spreading cells), and lamellipodial extension
**Q9NS69**	TOM22	Mitochondrial import receptor subunit TOM22 homolog OS=Homo sapiens OX=9606 GN=TOMM22 PE=1 SV=3	Central receptor component of the translocase of the outer membrane of mitochondria (TOM complex) responsible for the recognition and translocation of cytosolically synthesized mitochondrial preproteins. Together with the peripheral receptor TOM20 functions as the transit peptide receptor and facilitates the movement of preproteins into the translocation pore
**P38646**	GRP75	Stress-70 protein, mitochondrial OS=Homo sapiens OX=9606 GN=HSPA9 PE=1 SV=2	Chaperone protein which plays an important role in mitochondrial iron-sulfur cluster (ISC) biogenesis. Interacts with and stabilizes ISC cluster assembly proteins FXN, NFU1, NFS1 and ISCU. Regulates erythropoiesis via stabilization of ISC assembly. May play a role in the control of cell proliferation and cellular aging (By similarity)
**P54105**	ICLN	Methylosome subunit pICln OS=Homo sapiens OX=9606 GN=CLNS1A PE=1 SV=1	Chaperone that regulates the assembly of spliceosomal U1, U2, U4 and U5 small nuclear ribonucleoproteins (snRNPs), the building blocks of the spliceosome. Thereby, plays an important role in the splicing of cellular pre-mRNAs. Most spliceosomal snRNPs contain a common set of Sm proteins SNRPB, SNRPD1, SNRPD2, SNRPD3, SNRPE, SNRPF and SNRPG that assemble in a heptameric protein ring on the Sm site of the small nuclear RNA to form the core snRNP. In the cytosol, the Sm proteins SNRPD1, SNRPD2, SNRPE, SNRPF and SNRPG are trapped in an inactive 6S pICln-Sm comple [...] (237 aa)

**P10809**	CH60	60 kDa heat shock protein, mitochondrial OS=Homo sapiens OX=9606 GN=HSPD1 PE=1 SV=2	Chaperonin implicated in mitochondrial protein import and macromolecular assembly. Together with Hsp10, facilitates the correct folding of imported proteins. May also prevent misfolding and promote the refolding and proper assembly of unfolded polypeptides generated under stress conditions in the mitochondrial matrix. The functional units of these chaperonins consist of heptameric rings of the large subunit Hsp60, which function as a back-to-back double ring. In a cyclic reaction, Hsp60 ring complexes bind one unfolded substrate protein per rin [...] (573 aa)
**Q9Y657**	SPIN1	Spindlin-1 OS=Homo sapiens OX=9606 GN=SPIN1 PE=1 SV=3	Chromatin reader that specifically recognizes and binds histone H3 both trimethylated at 'Lys-4' and asymmetrically dimethylated at 'Arg-8' (H3K4me3 and H3R8me2a) and acts as an activator of Wnt signaling pathway downstream of PRMT2. In case of cancer, promotes cell cancer proliferation via activation of the Wnt signaling pathway. Overexpression induces metaphase arrest and chromosomal instability. Localizes to active rDNA loci and promotes the expression of rRNA genes. May play a role in cell-cycle regulation during the transition from gamete to embryo. Involved in oocyte [...] (262 aa)
**P51608**	MECP2	Methyl-CpG-binding protein 2 OS=Homo sapiens OX=9606 GN=MECP2 PE=1 SV=1	Chromosomal protein that binds to methylated DNA. It can bind specifically to a single methyl-CpG pair. It is not influenced by sequences flanking the methyl-CpGs. Mediates transcriptional repression through interaction with histone deacetylase and the corepressor SIN3A. Binds both 5-methylcytosine (5mC) and 5-hydroxymethylcytosine (5hmC)-containing DNA, with a preference for 5-methylcytosine (5mC) (498 aa)
**Q5SNV9**	CA167	Uncharacterized protein C1orf167 OS=Homo sapiens OX=9606 GN=C1orf167 PE=2 SV=2	Chromosome 1 open reading frame 167 (1468 aa); C1orf167 (Chromosome 1 Open Reading Frame 167) is a Protein Coding gene. Diseases associated with C1orf167 include Neural Tube Defects, Folate-Sensitive.
**Q8WUR7**	CO040	UPF0235 protein C15orf40 OS=Homo sapiens OX=9606 GN=C15orf40 PE=1 SV=2	Chromosome 15 open reading frame 40 (167 aa); C15orf40 (Chromosome 15 Open Reading Frame 40) is a Protein Coding gene.
**Q9BVC5**	ASHWN	Ashwin OS=Homo sapiens OX=9606 GN=C2orf49 PE=1 SV=1	Chromosome 2 open reading frame 49; C2orf49 (Chromosome 2 Open Reading Frame 49) is a Protein Coding gene. Among its related pathways are tRNA processing and Gene Expression.

**Q9BRJ6**	CG050	Uncharacterized protein C7orf50 OS=Homo sapiens OX=9606 GN=C7orf50 PE=1 SV=1	Chromosome 7 open reading frame 50 (194 aa); C7orf50 (Chromosome 7 Open Reading Frame 50) is a Protein Coding gene.
**Q9H7E9**	CH033	UPF0488 protein C8orf33 OS=Homo sapiens OX=9606 GN=C8orf33 PE=1 SV=1	Chromosome 8 open reading frame 33; C8orf33 (Chromosome 8 Open Reading Frame 33) is a Protein Coding gene.
**Q9UK59**	DBR1	Lariat debranching enzyme OS=Homo sapiens OX=9606 GN=DBR1 PE=1 SV=2	Cleaves the 2'-5' phosphodiester linkage at the branch point of lariat intron pre-mRNAs after splicing and converts them into linear molecules that are subsequently degraded. It thereby facilitates ribonucleotide turnover. It may also participate in retrovirus replication via an RNA lariat intermediate in cDNA synthesis
**P61604**	CH10	10 kDa heat shock protein, mitochondrial OS=Homo sapiens OX=9606 GN=HSPE1 PE=1 SV=2	Co-chaperonin implicated in mitochondrial protein import and macromolecular assembly. Together with Hsp60, facilitates the correct folding of imported proteins. May also prevent misfolding and promote the refolding and proper assembly of unfolded polypeptides generated under stress conditions in the mitochondrial matrix. The functional units of these chaperonins consist of heptameric rings of the large subunit Hsp60, which function as a back-to-back double ring. In a cyclic reaction, Hsp60 ring complexes bind one unfolded substrate protein per [...] (102 aa)
**P98179**	RBM3	RNA-binding protein 3 OS=Homo sapiens OX=9606 GN=RBM3 PE=1 SV=1	Cold-inducible mRNA binding protein that enhances global protein synthesis at both physiological and mild hypothermic temperatures. Reduces the relative abundance of microRNAs, when overexpressed. Enhances phosphorylation of translation initiation factors and active polysome formation (By similarity) (157 aa)
**Q14011**	CIRBP	Cold-inducible RNA-binding protein OS=Homo sapiens OX=9606 GN=CIRBP PE=1 SV=1	Cold-inducible mRNA binding protein that plays a protective role in the genotoxic stress response by stabilizing transcripts of genes involved in cell survival. Acts as a translational activator. Seems to play an essential role in cold- induced suppression of cell proliferation. Binds specifically to the 3'-untranslated regions (3'-UTRs) of stress-responsive transcripts RPA2 and TXN. Acts as a translational repressor (By similarity). Promotes assembly of stress granules (SGs), when overexpressed (297 aa)

**P14923**	PLAK	Junction plakoglobin OS=Homo sapiens OX=9606 GN=JUP PE=1 SV=3	Common junctional plaque protein. The membrane-associated plaques are architectural elements in an important strategic position to influence the arrangement and function of both the cytoskeleton and the cells within the tissue. The presence of plakoglobin in both the desmosomes and in the intermediate junctions suggests that it plays a central role in the structure and function of submembranous plaques. Acts as a substrate for VE-PTP and is required by it to stimulate VE- cadherin function in endothelial cells. Can replace beta-catenin in E-cadherin/catenin adhes [...] (745 aa)
**Q9HC52**	CBX8	Chromobox protein homolog 8 OS=Homo sapiens OX=9606 GN=CBX8 PE=1 SV=3	Component of a Polycomb group (PcG) multiprotein PRC1- like complex, a complex class required to maintain the transcriptionally repressive state of many genes, including Hox genes, throughout development. PcG PRC1 complex acts via chromatin remodeling and modification of histones
**Q5SSJ5**	HP1B3	Heterochromatin protein 1-binding protein 3 OS=Homo sapiens OX=9606 GN=HP1BP3 PE=1 SV=1	Component of heterochromatin that maintains heterochromatin integrity during G1/S progression and regulates the duration of G1 phase to critically influence cell proliferative capacity. Mediates chromatin condensation during hypoxia, leading to increased tumor cell viability, radio-resistance, chemo-resistance and self- renewal (553 aa)
**P45973**	CBX5	Chromobox protein homolog 5 OS=Homo sapiens OX=9606 GN=CBX5 PE=1 SV=1	Component of heterochromatin that recognizes and binds histone H3 tails methylated at ’Lys-9’ (H3K9me), leading to epigenetic repression. In contrast, it is excluded from chromatin when ’Tyr-41’ of histone H3 is phosphorylated (H3Y41ph). Can interact with lamin-B receptor (LBR). This interaction can contribute to the association of the heterochromatin with the inner nuclear membrane. Involved in the formation of functional kinetochore through interaction with MIS12 complex proteins
**P83916**	CBX1	Chromobox protein homolog 1 OS=Homo sapiens OX=9606 GN=CBX1 PE=1 SV=1	Component of heterochromatin. Recognizes and binds histone H3 tails methylated at ’Lys-9’, leading to epigenetic repression. Interaction with lamin B receptor (LBR) can contribute to the association of the heterochromatin with the inner nuclear membrane

**O43390**	HNRPR	Heterogeneous nuclear ribonucleoprotein R OS=Homo sapiens OX=9606 GN=HNRNPR PE=1 SV=1	Component of ribonucleosomes, which are complexes of at least 20 other different heterogenious nuclear ribonucleoproteins (hnRNP). hnRNP play an important role in processing of precursor mRNA in the nucleus
**O43242**	PSMD3	26S proteasome non-ATPase regulatory subunit 3 OS=Homo sapiens OX=9606 GN=PSMD3 PE=1 SV=2	Component of the 26S proteasome, a multiprotein complex involved in the ATP-dependent degradation of ubiquitinated proteins. This complex plays a key role in the maintenance of protein homeostasis by removing misfolded or damaged proteins, which could impair cellular functions, and by removing proteins whose functions are no longer required. Therefore, the proteasome participates in numerous cellular processes, including cell cycle progression, apoptosis, or DNA damage repair
**Q9NRL2**	BAZ1A	Bromodomain adjacent to zinc finger domain protein 1A OS=Homo sapiens OX=9606 GN=BAZ1A PE=1 SV=2	Component of the ACF complex, an ATP-dependent chromatin remodeling complex, that regulates spacing of nucleosomes using ATP to generate evenly spaced nucleosomes along the chromatin. The ATPase activity of the complex is regulated by the length of flanking DNA. Also involved in facilitating the DNA replication process. BAZ1A is the accessory, non-catalytic subunit of the complex which can enhance and direct the process provided by the ATPase subunit, SMARCA5, probably through targeting pericentromeric heterochromatin in late S pha [...] (1556 aa)
**O95782**	AP2A1	AP-2 complex subunit alpha-1 OS=Homo sapiens OX=9606 GN=AP2A1 PE=1 SV=3	Component of the adaptor protein complex 2 (AP-2). Adaptor protein complexes function in protein transport via transport vesicles in different membrane traffic pathways. Adaptor protein complexes are vesicle coat components and appear to be involved in cargo selection and vesicle formation. AP-2 is involved in clathrin-dependent endocytosis in which cargo proteins are incorporated into vesicles surrounded by clathrin (clathrin- coated vesicles, CCVs) which are destined for fusion with the early endosome. The clathrin lattice serves as a mechanical scaffold [...] (977 aa)

**O75175**	CNOT3	CCR4-NOT transcription complex subunit 3 OS=Homo sapiens OX=9606 GN=CNOT3 PE=1 SV=1	Component of the CCR4-NOT complex which is one of the major cellular mRNA deadenylases and is linked to various cellular processes including bulk mRNA degradation, miRNA-mediated repression, translational repression during translational initiation and general transcription regulation. Additional complex functions may be a consequence of its influence on mRNA expression. May be involved in metabolic regulation; may be involved in recruitment of the CCR4-NOT complex to deadenylation target mRNAs involved in energy metabolism. Involved in mitotic [...] (753 aa)
**Q9H0H5**	RGAP1	Rac GTPase-activating protein 1 OS=Homo sapiens OX=9606 GN=RACGAP1 PE=1 SV=1	Component of the centralspindlin complex that serves as a microtubule-dependent and Rho-mediated signaling required for the myosin contractile ring formation during the cell cycle cytokinesis. Required for proper attachment of the midbody to the cell membrane during cytokinesis. Plays key roles in controlling cell growth and differentiation of hematopoietic cells through mechanisms other than regulating Rac GTPase activity. Also involved in the regulation of growth-related processes in adipocytes and myoblasts. May be involved in regulating spermatogene [...] (632 aa)
**Q9NQS7**	INCE	Inner centromere protein OS=Homo sapiens OX=9606 GN=INCENP PE=1 SV=3	Component of the chromosomal passenger complex (CPC), a complex that acts as a key regulator of mitosis. The CPC complex has essential functions at the centromere in ensuring correct chromosome alignment and segregation and is required for chromatin-induced microtubule stabilization and spindle assembly. Acts as a scaffold regulating CPC localization and activity. The C-terminus associates with AURKB or AURKC, the N-terminus associated with BIRC5/survivin and CDCA8/borealin tethers the CPC to the inner centromere, and the microtubule binding activity within th [...] (918 aa)
**Q16630**	CPSF6	Cleavage and polyadenylation specificity factor subunit 6 OS=Homo sapiens OX=9606 GN=CPSF6 PE=1 SV=2	Component of the cleavage factor Im complex (CFIm) that plays a key role in pre-mRNA 3'-processing. Involved in association with NUDT21/CPSF5 in pre-MRNA 3'-end poly(A) site cleavage and poly(A) addition. CPSF6 binds to cleavage and polyadenylation RNA substrates and promotes RNA looping

**O94979**	SC31A	Protein transport protein Sec31A OS=Homo sapiens OX=9606 GN=SEC31A PE=1 SV=3	Component of the coat protein complex II (COPII) which promotes the formation of transport vesicles from the endoplasmic reticulum (ER). The coat has two main functions, the physical deformation of the endoplasmic reticulum membrane into vesicles and the selection of cargo molecules (By similarity)
**O00267**	SPT5H	Transcription elongation factor SPT5 OS=Homo sapiens OX=9606 GN=SUPT5H PE=1 SV=1	Component of the DRB sensitivity-inducing factor complex (DSIF complex), which regulates mRNA processing and transcription elongation by RNA polymerase II. DSIF positively regulates mRNA capping by stimulating the mRNA guanylyltransferase activity of RNGTT/CAP1A. DSIF also acts cooperatively with the negative elongation factor complex (NELF complex) to enhance transcriptional pausing at sites proximal to the promoter. Transcriptional pausing may facilitate the assembly of an elongation competent RNA polymerase II complex. DSIF and NELF promote paus [...] (1087 aa)
**Q9Y3C4**	TPRKB	EKC/KEOPS complex subunit TPRKB OS=Homo sapiens OX=9606 GN=TPRKB PE=1 SV=1	Component of the EKC/KEOPS complex that is required for the formation of a threonylcarbamoyl group on adenosine at position 37 (t(6)A37) in tRNAs that read codons beginning with adenine. The complex is probably involved in the transfer of the threonylcarbamoyl moiety of threonylcarbamoyl-AMP (TC-AMP) to the N6 group of A37. TPRKB acts as an allosteric effector that regulates the t(6)A activity of the complex. TPRKB is not required for tRNA modification (By similarity)
**Q86YZ3**	HORN	Hornerin OS=Homo sapiens OX=9606 GN=HRNR PE=1 SV=2	Component of the epidermal cornified cell envelopes; Diseases associated with HRNR include Ankyloblepharon-Ectodermal Defects-Cleft Lip/Palate. Among its related pathways are Innate Immune System.
**Q9UK41**	VPS28	Vacuolar protein sorting-associated protein 28 homolog OS=Homo sapiens OX=9606 GN=VPS28 PE=1 SV=1	Component of the ESCRT-I complex, a regulator of vesicular trafficking process (233 aa)
**O75822**	EIF3J	Eukaryotic translation initiation factor 3 subunit J OS=Homo sapiens OX=9606 GN=EIF3J PE=1 SV=2	Component of the eukaryotic translation initiation factor 3 (eIF-3) complex, which is required for several steps in the initiation of protein synthesis. The eIF-3 complex associates with the 40S ribosome and facilitates the recruitment of eIF-1, eIF-1A, eIF- 2-GTP-methionyl-tRNAi and eIF-5 to form the 43S pre-initiation complex (43S PIC). The eIF-3 complex stimulates mRNA recruitment to the 43S PIC and scanning of the mRNA for AUG recognition. The eIF-3 complex is also required for disassembly and recycling of post-termination ribos [...] (258 aa)

**Q08945**	SSRP1	FACT complex subunit SSRP1 OS=Homo sapiens OX=9606 GN=SSRP1 PE=1 SV=1	Component of the FACT complex, a general chromatin factor that acts to reorganize nucleosomes. The FACT complex is involved in multiple processes that require DNA as a template such as mRNA elongation, DNA replication and DNA repair. During transcription elongation the FACT complex acts as a histone chaperone that both destabilizes and restores nucleosomal structure. It facilitates the passage of RNA polymerase II and transcription by promoting the dissociation of one histone H2A-H2B dimer from the nucleosome, then subsequently promotes the reestablishment o [...] (709 aa)
**Q9Y5B9**	SP16H	FACT complex subunit SPT16 OS=Homo sapiens OX=9606 GN=SUPT16H PE=1 SV=1	Component of the FACT complex, a general chromatin factor that acts to reorganize nucleosomes. The FACT complex is involved in multiple processes that require DNA as a template such as mRNA elongation, DNA replication and DNA repair. During transcription elongation the FACT complex acts as a histone chaperone that both destabilizes and restores nucleosomal structure. It facilitates the passage of RNA polymerase II and transcription by promoting the dissociation of one histone H2A-H2B dimer from the nucleosome, then subsequently promotes the reestablishment o [...] (1047 aa)
**Q92613**	JADE3	Protein Jade-3 OS=Homo sapiens OX=9606 GN=JADE3 PE=1 SV=1	Component of the HBO1 complex which has a histone H4- specific acetyltransferase activity, a reduced activity toward histone H3 and is responsible for the bulk of histone H4 acetylation in vivo
**P52597**	HNRPF	Heterogeneous nuclear ribonucleoprotein F OS=Homo sapiens OX=9606 GN=HNRNPF PE=1 SV=3	Component of the heterogeneous nuclear ribonucleoprotein (hnRNP) complexes which provide the substrate for the processing events that pre-mRNAs undergo before becoming functional, translatable mRNAs in the cytoplasm. Plays a role in the regulation of alternative splicing events. Binds G-rich sequences in pre-mRNAs and keeps target RNA in an unfolded state
**P18621**	RL17	60S ribosomal protein L17 OS=Homo sapiens OX=9606 GN=RPL17 PE=1 SV=3	Component of the large ribosomal subunit; Diseases associated with RPL17 include Vesicoureteral Reflux 1. Among its related pathways are Viral mRNA Translation and Influenza Viral RNA Transcription and Replication.
**P46779**	RL28	60S ribosomal protein L28 OS=Homo sapiens OX=9606 GN=RPL28 PE=1 SV=3	Component of the large ribosomal subunit; Diseases associated with RPL28 include Albinism-Deafness Syndrome. Among its related pathways are Viral mRNA Translation and Influenza Viral RNA Transcription and Replication.

**P50914**	RL14	60S ribosomal protein L14 OS=Homo sapiens OX=9606 GN=RPL14 PE=1 SV=4	Component of the large ribosomal subunit; Among its related pathways are Viral mRNA Translation and Influenza Viral RNA Transcription and Replication.
**Q07020**	RL18	60S ribosomal protein L18 OS=Homo sapiens OX=9606 GN=RPL18 PE=1 SV=2	Component of the large ribosomal subunit; Diseases associated with RPL18 include Diamond-Blackfan Anemia. Among its related pathways are Viral mRNA Translation and Influenza Viral RNA Transcription and Replication.
**Q9Y3U8**	RL36	60S ribosomal protein L36 OS=Homo sapiens OX=9606 GN=RPL36 PE=1 SV=3	Component of the large ribosomal subunit (105 aa); Diseases associated with RPL36 include Retinitis Pigmentosa 36 and Retinitis Pigmentosa 59. Among its related pathways are Viral mRNA Translation and Influenza Viral RNA Transcription and Replication.
**P49207**	RL34	60S ribosomal protein L34 OS=Homo sapiens OX=9606 GN=RPL34 PE=1 SV=3	Component of the large ribosomal subunit (117 aa); Diseases associated with RPL34 include Baastrup's Syndrome and Supine Hypotensive Syndrome. Among its related pathways are Viral mRNA Translation and Influenza Viral RNA Transcription and Replication.
**P42766**	RL35	60S ribosomal protein L35 OS=Homo sapiens OX=9606 GN=RPL35 PE=1 SV=2	Component of the large ribosomal subunit (123 aa); Diseases associated with RPL35 include Diamond-Blackfan Anemia and Muscular Dystrophy, Congenital, Lmna-Related.
**P47914**	RL29	60S ribosomal protein L29 OS=Homo sapiens OX=9606 GN=RPL29 PE=1 SV=2	Component of the large ribosomal subunit (159 aa); Among its related pathways are Viral mRNA Translation and Influenza Viral RNA Transcription and Replication.
**P46778**	RL21	60S ribosomal protein L21 OS=Homo sapiens OX=9606 GN=RPL21 PE=1 SV=2	Component of the large ribosomal subunit (160 aa); Diseases associated with RPL21 include Hypotrichosis 12 and Hypotrichosis Simplex. Among its related pathways are Viral mRNA Translation and Influenza Viral RNA Transcription and Replication.
**P62906**	RL10A	60S ribosomal protein L10a OS=Homo sapiens OX=9606 GN=RPL10A PE=1 SV=2	Component of the large ribosomal subunit (217 aa); Among its related pathways are Viral mRNA Translation and Influenza Viral RNA Transcription and Replication.
**P62917**	RL8	60S ribosomal protein L8 OS=Homo sapiens OX=9606 GN=RPL8 PE=1 SV=2	Component of the large ribosomal subunit (257 aa); Among its related pathways are Viral mRNA Translation and Influenza Viral RNA Transcription and Replication.
**Q02878**	RL6	60S ribosomal protein L6 OS=Homo sapiens OX=9606 GN=RPL6 PE=1 SV=3	Component of the large ribosomal subunit (288 aa); Specifically binds to domain C of the Tax-responsive enhancer element in the long terminal repeat of HTLV-I (PubMed:8457378).
**P18124**	RL7	60S ribosomal protein L7 OS=Homo sapiens OX=9606 GN=RPL7 PE=1 SV=1	Component of the large ribosomal subunit. Binds to G-rich structures in 28S rRNA and in mRNAs. Plays a regulatory role in the translation apparatus

**P27635**	RL10	60S ribosomal protein L10 OS=Homo sapiens OX=9606 GN=RPL10 PE=1 SV=4	Component of the large ribosomal subunit. Plays a role in the formation of actively translating ribosomes. May play a role in the embryonic brain development (214 aa)
**P61353**	RL27	60S ribosomal protein L27 OS=Homo sapiens OX=9606 GN=RPL27 PE=1 SV=2	Component of the large ribosomal subunit. Required for proper rRNA processing and maturation of 28S and 5.8S rRNAs (136 aa)
**O95696**	BRD1	Bromodomain-containing protein 1 OS=Homo sapiens OX=9606 GN=BRD1 PE=1 SV=1	Component of the MOZ/MORF complex which has a histone H3 acetyltransferase activity
**Q92878**	RAD50	DNA repair protein RAD50 OS=Homo sapiens OX=9606 GN=RAD50 PE=1 SV=1	Component of the MRN complex, which plays a central role in double-strand break (DSB) repair, DNA recombination, maintenance of telomere integrity and meiosis. The complex possesses single-strand endonuclease activity and double-strand- specific 3’-5’ exonuclease activity, which are provided by MRE11. RAD50 may be required to bind DNA ends and hold them in close proximity. This could facilitate searches for short or long regions of sequence homology in the recombining DNA templates, and may also stimulate the activity of DNA ligases and/or restrict the nucleas [...] (1312 aa)
**Q9NV56**	MRGBP	MRG/MORF4L-binding protein OS=Homo sapiens OX=9606 GN=MRGBP PE=1 SV=1	Component of the NuA4 histone acetyltransferase (HAT) complex which is involved in transcriptional activation of select genes principally by acetylation of nucleosomal histones H4 and H2A. This modification may both alter nucleosome - DNA interactions and promote interaction of the modified histones with other proteins which positively regulate transcription. This complex may be required for the activation of transcriptional programs associated with oncogene and proto-oncogene mediated growth induction, tumor suppressor mediated growth arrest and replicative [...] (204 aa)

**Q15014**	MO4L2	Mortality factor 4-like protein 2 OS=Homo sapiens OX=9606 GN=MORF4L2 PE=1 SV=1	Component of the NuA4 histone acetyltransferase complex which is involved in transcriptional activation of select genes principally by acetylation of nucleosomal histone H4 and H2A. This modification may both alter nucleosome - DNA interactions and promote interaction of the modified histones with other proteins which positively regulate transcription. This complex may be required for the activation of transcriptional programs associated with oncogene and proto-oncogene mediated growth induction, tumor suppressor mediated growth arrest and replicative [...] (288 aa)
**Q9GZS3**	WDR61	WD repeat-containing protein 61 OS=Homo sapiens OX=9606 GN=WDR61 PE=1 SV=1	Component of the PAF1 complex (PAF1C) which has multiple functions during transcription by RNA polymerase II and is implicated in regulation of development and maintenance of embryonic stem cell pluripotency. PAF1C associates with RNA polymerase II through interaction with POLR2A CTD non- phosphorylated and ’Ser-2’- and ’Ser-5’-phosphorylated forms and is involved in transcriptional elongation, acting both indepentently and synergistically with TCEA1 and in cooperation with the DSIF complex and HTATSF1. PAF1C is required for transcription of Hox and Wnt [...] (305 aa)
**O00541**	PESC	Pescadillo homolog OS=Homo sapiens OX=9606 GN=PES1 PE=1 SV=1	Component of the PeBoW complex, which is required for maturation of 28S and 5.8S ribosomal RNAs and formation of the 60S ribosome
**Q14137**	BOP1	Ribosome biogenesis protein BOP1 OS=Homo sapiens OX=9606 GN=BOP1 PE=1 SV=2	Component of the PeBoW complex, which is required for maturation of 28S and 5.8S ribosomal RNAs and formation of the 60S ribosome
**Q04637**	IF4G1	Eukaryotic translation initiation factor 4 gamma 1 OS=Homo sapiens OX=9606 GN=EIF4G1 PE=1 SV=4	Component of the protein complex eIF4F, which is involved in the recognition of the mRNA cap, ATP-dependent unwinding of 5’-terminal secondary structure and recruitment of mRNA to the ribosome
**O43660**	PLRG1	Pleiotropic regulator 1 OS=Homo sapiens OX=9606 GN=PLRG1 PE=1 SV=1	Component of the PRP19-CDC5L complex that forms an integral part of the spliceosome and is required for activating pre-mRNA splicing (514 aa)
**Q8WYA6**	CTBL1	Beta-catenin-like protein 1 OS=Homo sapiens OX=9606 GN=CTNNBL1 PE=1 SV=1	Component of the PRP19-CDC5L complex that forms an integral part of the spliceosome and is required for activating pre-mRNA splicing. Participates in AID/AICDA-mediated Ig class switching recombination (CSR). May induce apoptosis

**Q9UKD2**	MRT4	mRNA turnover protein 4 homolog OS=Homo sapiens OX=9606 GN=MRTO4 PE=1 SV=2	Component of the ribosome assembly machinery. Nuclear paralog of the ribosomal protein P0, it binds pre-60S subunits at an early stage of assembly in the nucleolus, and is replaced by P0 in cytoplasmic pre-60S subunits and mature 80S ribosomes (239 aa)
**P46777**	RL5	60S ribosomal protein L5 OS=Homo sapiens OX=9606 GN=RPL5 PE=1 SV=3	Component of the ribosome, a large ribonucleoprotein complex responsible for the synthesis of proteins in the cell. The small ribosomal subunit (SSU) binds messenger RNAs (mRNAs) and translates the encoded message by selecting cognate aminoacyl- transfer RNA (tRNA) molecules. The large subunit (LSU) contains the ribosomal catalytic site termed the peptidyl transferase center (PTC), which catalyzes the formation of peptide bonds, thereby polymerizing the amino acids delivered by tRNAs into a polypeptide chain. The nascent polypeptides leave the ribosome through [...] (297 aa)
**P62913**	RL11	60S ribosomal protein L11 OS=Homo sapiens OX=9606 GN=RPL11 PE=1 SV=2	Component of the ribosome, a large ribonucleoprotein complex responsible for the synthesis of proteins in the cell. The small ribosomal subunit (SSU) binds messenger RNAs (mRNAs) and translates the encoded message by selecting cognate aminoacyl- transfer RNA (tRNA) molecules. The large subunit (LSU) contains the ribosomal catalytic site termed the peptidyl transferase center (PTC), which catalyzes the formation of peptide bonds, thereby polymerizing the amino acids delivered by tRNAs into a polypeptide chain. The nascent polypeptides leave the ribosome throug [...] (178 aa)
**P62266**	RS23	40S ribosomal protein S23 OS=Homo sapiens OX=9606 GN=RPS23 PE=1 SV=3	Component of the ribosome, a large ribonucleoprotein complex responsible for the synthesis of proteins in the cell. The small ribosomal subunit (SSU) binds messenger RNAs (mRNAs) and translates the encoded message by selecting cognate aminoacyl-transfer RNA (tRNA) molecules. The large subunit (LSU) contains the ribosomal catalytic site termed the peptidyl transferase center (PTC), which catalyzes the formation of peptide bonds, thereby polymerizing the amino acids delivered by tRNAs into a polypeptide chain. The nascent polypeptides leave the ribosome through [...] (143 aa)

**Q9UBL3**	ASH2L	Set1/Ash2 histone methyltransferase complex subunit ASH2 OS=Homo sapiens OX=9606 GN=ASH2L PE=1 SV=1	Component of the Set1/Ash2 histone methyltransferase (HMT) complex, a complex that specifically methylates 'Lys-4' of histone H3, but not if the neighboring 'Lys-9' residue is already methylated. As part of the MLL1/MLL complex it is involved in methylation and dimethylation at 'Lys-4' of histone H3. May function as a transcriptional regulator. May play a role in hematopoiesis
**P42677**	RS27	40S ribosomal protein S27 OS=Homo sapiens OX=9606 GN=RPS27 PE=1 SV=3	Component of the small ribosomal subunit. Required for proper rRNA processing and maturation of 18S rRNAs
**P08621**	RU17	U1 small nuclear ribonucleoprotein 70 kDa OS=Homo sapiens OX=9606 GN=SNRNP70 PE=1 SV=2	Component of the spliceosomal U1 snRNP, which is essential for recognition of the pre-mRNA 5' splice-site and the subsequent assembly of the spliceosome. SNRNP70 binds to the loop I region of U1-snRNA. The truncated isoforms cannot bind U1-snRNA
**P09234**	RU1C	U1 small nuclear ribonucleoprotein C OS=Homo sapiens OX=9606 GN=SNRPC PE=1 SV=1	Component of the spliceosomal U1 snRNP, which is essential for recognition of the pre-mRNA 5' splice-site and the subsequent assembly of the spliceosome. SNRPC/U1-C is directly involved in initial 5' splice-site recognition for both constitutive and regulated alternative splicing. The interaction with the 5' splice-site seems to precede base-pairing between the pre-mRNA and the U1 snRNA. Stimulates commitment or early (E) complex formation by stabilizing the base pairing of the 5' end of the U1 snRNA and the 5' splice-site region
**P09012**	SNRPA	U1 small nuclear ribonucleoprotein A OS=Homo sapiens OX=9606 GN=SNRPA PE=1 SV=3	Component of the spliceosomal U1 snRNP, which is essential for recognition of the pre-mRNA 5' splice-site and the subsequent assembly of the spliceosome. U1 snRNP is the first snRNP to interact with pre-mRNA. This interaction is required for the subsequent binding of U2 snRNP and the U4/U6/U5 tri-snRNP. SNRPA binds stem loop II of U1 snRNA. In a snRNP-free form (SF-A) may be involved in coupled pre-mRNA splicing and polyadenylation process. May bind preferentially to the 5'-UGCAC-3' motif on RNAs
**Q9Y5M8**	SRPRB	Signal recognition particle receptor subunit beta OS=Homo sapiens OX=9606 GN=SRPRB PE=1 SV=3	Component of the SRP (signal recognition particle) receptor. Ensures, in conjunction with the signal recognition particle, the correct targeting of the nascent secretory proteins to the endoplasmic reticulum membrane system. Has GTPase activity. May mediate the membrane association of SRPR (By similarity) (271 aa)

**Q15029**	U5S1	116 kDa U5 small nuclear ribonucleoprotein component OS=Homo sapiens OX=9606 GN=EFTUD2 PE=1 SV=1	Component of the U5 snRNP and the U4/U6-U5 tri-snRNP complex required for pre-mRNA splicing. Binds GTP
**Q02880**	TOP2B	DNA topoisomerase 2-beta OS=Homo sapiens OX=9606 GN=TOP2B PE=1 SV=3	Control of topological states of DNA by transient breakage and subsequent rejoining of DNA strands. Topoisomerase II makes double-strand breaks
**P11388**	TOP2A	DNA topoisomerase 2-alpha OS=Homo sapiens OX=9606 GN=TOP2A PE=1 SV=3	Control of topological states of DNA by transient breakage and subsequent rejoining of DNA strands. Topoisomerase II makes double-strand breaks. Essential during mitosis and meiosis for proper segregation of daughter chromosomes. May play a role in regulating the period length of ARNTL/BMAL1 transcriptional oscillation (By similarity)
**P12821**	ACE	Angiotensin-converting enzyme OS=Homo sapiens OX=9606 GN=ACE PE=1 SV=1	Converts angiotensin I to angiotensin II by release of the terminal His-Leu, this results in an increase of the vasoconstrictor activity of angiotensin. Also able to inactivate bradykinin, a potent vasodilator. Has also a glycosidase activity which releases GPI-anchored proteins from the membrane by cleaving the mannose linkage in the GPI moiety
**P58876**	H2B1D	Histone H2B type 1-D OS=Homo sapiens OX=9606 GN=HIST1H2BD PE=1 SV=2	Core component of nucleosome. Nucleosomes wrap and compact DNA into chromatin, limiting DNA accessibility to the cellular machineries which require DNA as a template. Histones thereby play a central role in transcription regulation, DNA repair, DNA replication and chromosomal stability. DNA accessibility is regulated via a complex set of post-translational modifications of histones, also called histone code, and nucleosome remodeling (126 aa)
**P62805**	H4	Histone H4 OS=Homo sapiens OX=9606 GN=HIST1H4A PE=1 SV=2	Core component of nucleosome. Nucleosomes wrap and compact DNA into chromatin, limiting DNA accessibility to the cellular machineries which require DNA as a template. Histones thereby play a central role in transcription regulation, DNA repair, DNA replication and chromosomal stability. DNA accessibility is regulated via a complex set of post-translational modifications of histones, also called histone code, and nucleosome remodeling (103 aa)

**P68431**	H31	Histone H3.1 OS=Homo sapiens OX=9606 GN=HIST1H3A PE=1 SV=2	Core component of nucleosome. Nucleosomes wrap and compact DNA into chromatin, limiting DNA accessibility to the cellular machineries which require DNA as a template. Histones thereby play a central role in transcription regulation, DNA repair, DNA replication and chromosomal stability. DNA accessibility is regulated via a complex set of post-translational modifications of histones, also called histone code, and nucleosome remodeling (136 aa)
**Q16778**	H2B2E	Histone H2B type 2-E OS=Homo sapiens OX=9606 GN=HIST2H2BE PE=1 SV=3	Core component of nucleosome. Nucleosomes wrap and compact DNA into chromatin, limiting DNA accessibility to the cellular machineries which require DNA as a template. Histones thereby play a central role in transcription regulation, DNA repair, DNA replication and chromosomal stability. DNA accessibility is regulated via a complex set of post-translational modifications of histones, also called histone code, and nucleosome remodeling (126 aa)
**Q8IUE6**	H2A2B	Histone H2A type 2-B OS=Homo sapiens OX=9606 GN=HIST2H2AB PE=1 SV=3	Core component of nucleosome. Nucleosomes wrap and compact DNA into chromatin, limiting DNA accessibility to the cellular machineries which require DNA as a template. Histones thereby play a central role in transcription regulation, DNA repair, DNA replication and chromosomal stability. DNA accessibility is regulated via a complex set of post-translational modifications of histones, also called histone code, and nucleosome remodeling (130 aa)
**Q99878**	H2A1J	Histone H2A type 1-J OS=Homo sapiens OX=9606 GN=HIST1H2AJ PE=1 SV=3	Core component of nucleosome. Nucleosomes wrap and compact DNA into chromatin, limiting DNA accessibility to the cellular machineries which require DNA as a template. Histones thereby play a central role in transcription regulation, DNA repair, DNA replication and chromosomal stability. DNA accessibility is regulated via a complex set of post-translational modifications of histones, also called histone code, and nucleosome remodeling (128 aa)

**Q99879**	H2B1M	Histone H2B type 1-M OS=Homo sapiens OX=9606 GN=HIST1H2BM PE=1 SV=3	Core component of nucleosome. Nucleosomes wrap and compact DNA into chromatin, limiting DNA accessibility to the cellular machineries which require DNA as a template. Histones thereby play a central role in transcription regulation, DNA repair, DNA replication and chromosomal stability. DNA accessibility is regulated via a complex set of post-translational modifications of histones, also called histone code, and nucleosome remodeling (126 aa)
**P62306**	RUXF	Small nuclear ribonucleoprotein F OS=Homo sapiens OX=9606 GN=SNRPF PE=1 SV=1	Core component of the spliceosomal U1, U2, U4 and U5 small nuclear ribonucleoproteins (snRNPs), the building blocks of the spliceosome. Thereby, plays an important role in the splicing of cellular pre-mRNAs. Most spliceosomal snRNPs contain a common set of Sm proteins SNRPB, SNRPD1, SNRPD2, SNRPD3, SNRPE, SNRPF and SNRPG that assemble in a heptameric protein ring on the Sm site of the small nuclear RNA to form the core snRNP. As part of the U7 snRNP it is involved in histone 3'-end processing (86 aa)
**P62308**	RUXG	Small nuclear ribonucleoprotein G OS=Homo sapiens OX=9606 GN=SNRPG PE=1 SV=1	Core component of the spliceosomal U1, U2, U4 and U5 small nuclear ribonucleoproteins (snRNPs), the building blocks of the spliceosome. Thereby, plays an important role in the splicing of cellular pre-mRNAs. Most spliceosomal snRNPs contain a common set of Sm proteins SNRPB, SNRPD1, SNRPD2, SNRPD3, SNRPE, SNRPF and SNRPG that assemble in a heptameric protein ring on the Sm site of the small nuclear RNA to form the core snRNP. Appears to function in the U7 snRNP complex that is involved in histone 3'-end processing (76 aa)
**P62314**	SMD1	Small nuclear ribonucleoprotein Sm D1 OS=Homo sapiens OX=9606 GN=SNRPD1 PE=1 SV=1	Core component of the spliceosomal U1, U2, U4 and U5 small nuclear ribonucleoproteins (snRNPs), the building blocks of the spliceosome. Thereby, plays an important role in the splicing of cellular pre-mRNAs. Most spliceosomal snRNPs contain a common set of Sm proteins SNRPB, SNRPD1, SNRPD2, SNRPD3, SNRPE, SNRPF and SNRPG that assemble in a heptameric protein ring on the Sm site of the small nuclear RNA to form the core snRNP. May act as a charged protein scaffold to promote snRNP assembly or strengthen snRNP- snRNP interactions through nonspecific [...] (119 aa)

**P62316**	SMD2	Small nuclear ribonucleoprotein Sm D2 OS=Homo sapiens OX=9606 GN=SNRPD2 PE=1 SV=1	Core component of the spliceosomal U1, U2, U4 and U5 small nuclear ribonucleoproteins (snRNPs), the building blocks of the spliceosome. Thereby, plays an important role in the splicing of cellular pre-mRNAs. Most spliceosomal snRNPs contain a common set of Sm proteins SNRPB, SNRPD1, SNRPD2, SNRPD3, SNRPE, SNRPF and SNRPG that assemble in a heptameric protein ring on the Sm site of the small nuclear RNA to form the core snRNP (118 aa)
**P62318**	SMD3	Small nuclear ribonucleoprotein Sm D3 OS=Homo sapiens OX=9606 GN=SNRPD3 PE=1 SV=1	Core component of the spliceosomal U1, U2, U4 and U5 small nuclear ribonucleoproteins (snRNPs), the building blocks of the spliceosome. Thereby, plays an important role in the splicing of cellular pre-mRNAs. Most spliceosomal snRNPs contain a common set of Sm proteins SNRPB, SNRPD1, SNRPD2, SNRPD3, SNRPE, SNRPF and SNRPG that assemble in a heptameric protein ring on the Sm site of the small nuclear RNA to form the core snRNP. As part of the U7 snRNP it is involved in histone 3'-end processing (126 aa)
**O15234**	CASC3	Protein CASC3 OS=Homo sapiens OX=9606 GN=CASC3 PE=1 SV=2	Core component of the splicing-dependent multiprotein exon junction complex (EJC) deposited at splice junctions on mRNAs. The EJC is a dynamic structure consisting of core proteins and several peripheral nuclear and cytoplasmic associated factors that join the complex only transiently either during EJC assembly or during subsequent mRNA metabolism. The EJC marks the position of the exon-exon junction in the mature mRNA for the gene expression machinery and the core components remain bound to spliced mRNAs throughout all stages of mRNA metabolism thereby influencing downs [...] (703 aa)
**Q9Y5S9**	RBM8A	RNA-binding protein 8A OS=Homo sapiens OX=9606 GN=RBM8A PE=1 SV=1	Core component of the splicing-dependent multiprotein exon junction complex (EJC) deposited at splice junctions on mRNAs. The EJC is a dynamic structure consisting of core proteins and several peripheral nuclear and cytoplasmic associated factors that join the complex only transiently either during EJC assembly or during subsequent mRNA metabolism. The EJC marks the position of the exon-exon junction in the mature mRNA for the gene expression machinery and the core components remain bound to spliced mRNAs throughout all stages of mRNA metabolism thereby influenc [...] (174 aa)

**Q09028**	RBBP4	Histone-binding protein RBBP4 OS=Homo sapiens OX=9606 GN=RBBP4 PE=1 SV=3	Core histone-binding subunit that may target chromatin assembly factors, chromatin remodeling factors and histone deacetylases to their histone substrates in a manner that is regulated by nucleosomal DNA. Component of several complexes which regulate chromatin metabolism. These include the chromatin assembly factor 1 (CAF-1) complex, which is required for chromatin assembly following DNA replication and DNA repair
**Q16576**	RBBP7	Histone-binding protein RBBP7 OS=Homo sapiens OX=9606 GN=RBBP7 PE=1 SV=1	Core histone-binding subunit that may target chromatin remodeling factors, histone acetyltransferases and histone deacetylases to their histone substrates in a manner that is regulated by nucleosomal DNA. Component of several complexes which regulate chromatin metabolism. These include the type B histone acetyltransferase (HAT) complex, which is required for chromatin assembly following DNA replication
**P53582**	MAP11	Methionine aminopeptidase 1 OS=Homo sapiens OX=9606 GN=METAP1 PE=1 SV=2	Cotranslationally removes the N-terminal methionine from nascent proteins. The N-terminal methionine is often cleaved when the second residue in the primary sequence is small and uncharged (Met-Ala-, Cys, Gly, Pro, Ser, Thr, or Val). Required for normal progression through the cell cycle
**Q13588**	GRAP	GRB2-related adapter protein OS=Homo sapiens OX=9606 GN=GRAP PE=1 SV=1	Couples signals from receptor and cytoplasmic tyrosine kinases to the Ras signaling pathway
**Q9Y295**	DRG1	Developmentally-regulated GTP-binding protein 1 OS=Homo sapiens OX=9606 GN=DRG1 PE=1 SV=1	Critical regulator of cell growth under specific conditions. Implicated in differentiation and cell cycle arrest (367 aa)
**P12081**	SYHC	Histidine--tRNA ligase, cytoplasmic OS=Homo sapiens OX=9606 GN=HARS PE=1 SV=2	Cytoplasmic histidine--tRNA ligase (Probable). Plays a role in axon guidance
**P58107**	EPIPL	Epiplakin OS=Homo sapiens OX=9606 GN=EPPK1 PE=1 SV=3	Cytoskeletal linker protein that connects to intermediate filaments and controls their reorganization in response to stress. In response to mechanical stress like wound healing, is associated with the machinery for cellular motility by slowing down keratinocyte migration and proliferation and accelerating keratin bundling in proliferating keratinocytes thus contributing to tissue architecture. However in wound healing in corneal epithelium also positively regulates cell differentiation and proliferation and negatively regulates migration thereby controlling corneal epitheliu [...] (5088 aa)

**P81605**	DCD	Dermcidin OS=Homo sapiens OX=9606 GN=DCD PE=1 SV=2	DCD-1 displays antimicrobial activity thereby limiting skin infection by potential pathogens in the first few hours after bacterial colonization. Highly effective against E.coli, E.faecalis, S.aureus and C.albicans. Optimal pH and salt concentration resemble the conditions in sweat. Also exhibits proteolytic activity, cleaving on the C-terminal side of Arg and, to a lesser extent, Lys residues (121 aa)
**Q8IY37**	DHX37	Probable ATP-dependent RNA helicase DHX37 OS=Homo sapiens OX=9606 GN=DHX37 PE=1 SV=1	DEAH-box helicase 37 (1157 aa); This gene encodes a DEAD box protein. DEAD box proteins, characterized by the conserved motif Asp-Glu-Ala-Asp (DEAD), are putative RNA helicases. They are implicated in a number of cellular processes involving alteration of RNA secondary structure such as translation initiation, nuclear and mitochondrial splicing, and ribosome and spliceosome assembly. Based on their distribution patterns, some members of this family are believed to be involved in embryogenesis, spermatogenesis, and cellular growth and division.
**Q9NYF8**	BCLF1	Bcl-2-associated transcription factor 1 OS=Homo sapiens OX=9606 GN=BCLAF1 PE=1 SV=2	Death-promoting transcriptional repressor. May be involved in cyclin-D1/CCND1 mRNA stability through the SNARP complex which associates with both the 3'end of the CCND1 gene and its mRNA (920 aa)
**Q9BQ39**	DDX50	ATP-dependent RNA helicase DDX50 OS=Homo sapiens OX=9606 GN=DDX50 PE=1 SV=1	DExD-box helicase 50; DEAD box proteins, characterized by the conserved motif Asp-Glu-Ala-Asp (DEAD), are putative RNA helicases. They are implicated in a number of cellular processes involving alteration of RNA secondary structure such as translation initiation, nuclear and mitochondrial splicing, and ribosome and spliceosome assembly. Based on their distribution patterns, some members of this DEAD box protein family are believed to be involved in embryogenesis, spermatogenesis, and cellular growth and division. This gene encodes a DEAD box enzyme that may be involved in ribosomal RNA synthesis or processing. This gene and DDX21, also called RH-II/GuA, have similar genomic structures and are in tandem orientation on chromosome 10, suggesting that the two genes arose by gene duplication in evolution. This gene has pseudogenes on chromosomes 2, 3 and 4. Alternative splicing of this gene generates multiple transcript variants, but the full length nature of all the other variants but one has not been defined.

**P35030**	TRY3	Trypsin-3 OS=Homo sapiens OX=9606 GN=PRSS3 PE=1 SV=2	Digestive protease specialized for the degradation of trypsin inhibitors. In the ileum, may be involved in defensin processing, including DEFA5
**Q96HS1**	PGAM5	Serine/threonine-protein phosphatase PGAM5, mitochondrial OS=Homo sapiens OX=9606 GN=PGAM5 PE=1 SV=2	Displays phosphatase activity for serine/threonine residues, and, dephosphorylates and activates MAP3K5 kinase. Has apparently no phosphoglycerate mutase activity. May be regulator of mitochondrial dynamics. Substrate for a KEAP1-dependent ubiquitin ligase complex. Contributes to the repression of NFE2L2- dependent gene expression. Acts as a central mediator for programmed necrosis induced by TNF, by reactive oxygen species and by calcium ionophore (289 aa)
**Q15233**	NONO	Non-POU domain-containing octamer-binding protein OS=Homo sapiens OX=9606 GN=NONO PE=1 SV=4	DNA- and RNA binding protein, involved in several nuclear processes. Binds the conventional octamer sequence in double-stranded DNA. Also binds single-stranded DNA and RNA at a site independent of the duplex site. Involved in pre-mRNA splicing, probably as a heterodimer with SFPQ. Interacts with U5 snRNA, probably by binding to a purine-rich sequence located on the 3' side of U5 snRNA stem 1b. Together with PSPC1, required for the formation of nuclear paraspeckles. The SFPQ-NONO heteromer associated with MATR3 may play a role in nuclea [...] (471 aa)
**P23246**	SFPQ	Splicing factor, proline- and glutamine-rich OS=Homo sapiens OX=9606 GN=SFPQ PE=1 SV=2	DNA- and RNA binding protein, involved in several nuclear processes. Essential pre-mRNA splicing factor required early in spliceosome formation and for splicing catalytic step II, probably as a heteromer with NONO. Binds to pre-mRNA in spliceosome C complex, and specifically binds to intronic polypyrimidine tracts. Involved in regulation of signal-induced alternative splicing. During splicing of PTPRC/CD45, a phosphorylated form is sequestered by THRAP3 from the pre-mRNA in resting T-cells

**Q00839**	HNRPU	Heterogeneous nuclear ribonucleoprotein U OS=Homo sapiens OX=9606 GN=HNRNPU PE=1 SV=6	DNA- and RNA-binding protein involved in several cellular processes such as nuclear chromatin organization, telomere-length regulation, transcription, mRNA alternative splicing and stability, Xist-mediated transcriptional silencing and mitotic cell progression. Plays a role in the regulation of interphase large-scale gene-rich chromatin organization through chromatin- associated RNAs (caRNAs) in a transcription-dependent manner, and thereby maintains genomic stability. Required for the localization of the long non-coding Xist RNA on the inacti [...] (825 aa)
**Q9UHX1**	PUF60	Poly(U)-binding-splicing factor PUF60 OS=Homo sapiens OX=9606 GN=PUF60 PE=1 SV=1	DNA- and RNA-binding protein, involved in several nuclear processes such as pre-mRNA splicing, apoptosis and transcription regulation. In association with FUBP1 regulates MYC transcription at the P2 promoter through the core-TFIIH basal transcription factor. Acts as a transcriptional repressor through the core-TFIIH basal transcription factor. Represses FUBP1-induced transcriptional activation but not basal transcription. Decreases ERCC3 helicase activity. Does not repress TFIIH-mediated transcription in xeroderma pigmentosum complementation group [...] (559 aa)
**Q86WJ1**	CHD1L	Chromodomain-helicase-DNA-binding protein 1-like OS=Homo sapiens OX=9606 GN=CHD1L PE=1 SV=3	DNA helicase which plays a role in chromatin-remodeling following DNA damage. Targeted to sites of DNA damage through interaction with poly(ADP-ribose) and functions to regulate chromatin during DNA repair. Able to catalyze nucleosome sliding in an ATP-dependent manner. Helicase activity is strongly stimulated upon poly(ADP-ribose)-binding
**Q9NUW8**	TYDP1	Tyrosyl-DNA phosphodiesterase 1 OS=Homo sapiens OX=9606 GN=TDP1 PE=1 SV=2	DNA repair enzyme that can remove a variety of covalent adducts from DNA through hydrolysis of a 3’-phosphodiester bond, giving rise to DNA with a free 3’ phosphate. Catalyzes the hydrolysis of dead-end complexes between DNA and the topoisomerase I active site tyrosine residue. Hydrolyzes 3’-phosphoglycolates on protruding 3’ ends on DNA double-strand breaks due to DNA damage by radiation and free radicals. Acts on blunt-ended double-strand DNA breaks and on single-stranded DNA. Has low 3’exonuclease activity and can remove a single nucleoside from the [...] (608 aa)

**Q99459**	CDC5L	Cell division cycle 5-like protein OS=Homo sapiens OX=9606 GN=CDC5L PE=1 SV=2	DNA-binding protein involved in cell cycle control. May act as a transcription activator. Component of the PRP19-CDC5L complex that forms an integral part of the spliceosome and is required for activating pre-mRNA splicing. The PRP19-CDC5L complex may also play a role in the response to DNA damage (DDR)
**O15514**	RPB4	DNA-directed RNA polymerase II subunit RPB4 OS=Homo sapiens OX=9606 GN=POLR2D PE=1 SV=1	DNA-dependent RNA polymerase catalyzes the transcription of DNA into RNA using the four ribonucleoside triphosphates as substrates. Component of RNA polymerase II which synthesizes mRNA precursors and many functional non-coding RNAs. Pol II is the central component of the basal RNA polymerase II transcription machinery. It is composed of mobile elements that move relative to each other. RPB4 is part of a subcomplex with RPB7 that binds to a pocket formed by RPB1, RPB2 and RPB6 at the base of the clamp element. The RBP4-RPB7 subcomplex seems [...] (142 aa)
**P36954**	RPB9	DNA-directed RNA polymerase II subunit RPB9 OS=Homo sapiens OX=9606 GN=POLR2I PE=1 SV=1	DNA-dependent RNA polymerase catalyzes the transcription of DNA into RNA using the four ribonucleoside triphosphates as substrates. Component of RNA polymerase II which synthesizes mRNA precursors and many functional non-coding RNAs. Pol II is the central component of the basal RNA polymerase II transcription machinery. It is composed of mobile elements that move relative to each other. RPB9 is part of the upper jaw surrounding the central large cleft and thought to grab the incoming DNA template (By similarity) (125 aa)
**P24928**	RPB1	DNA-directed RNA polymerase II subunit RPB1 OS=Homo sapiens OX=9606 GN=POLR2A PE=1 SV=2	DNA-dependent RNA polymerase catalyzes the transcription of DNA into RNA using the four ribonucleoside triphosphates as substrates. Largest and catalytic component of RNA polymerase II which synthesizes mRNA precursors and many functional non-coding RNAs. Forms the polymerase active center together with the second largest subunit. Pol II is the central component of the basal RNA polymerase II transcription machinery. It is composed of mobile elements that move relative to each other. RPB1 is part of the core element with the central large cl [...] (1980 aa)
**P56945**	BCAR1	Breast cancer anti-estrogen resistance protein 1 OS=Homo sapiens OX=9606 GN=BCAR1 PE=1 SV=2	Docking protein which plays a central coordinating role for tyrosine kinase-based signaling related to cell adhesion. Implicated in induction of cell migration. Overexpression confers antiestrogen resistance on breast cancer cells

**Q86UE4**	LYRIC	Protein LYRIC OS=Homo sapiens OX=9606 GN=MTDH PE=1 SV=2	Downregulates SLC1A2/EAAT2 promoter activity when expressed ectopically. Activates the nuclear factor kappa-B (NF- kappa-B) transcription factor. Promotes anchorage-independent growth of immortalized melanocytes and astrocytes which is a key component in tumor cell expansion. Promotes lung metastasis and also has an effect on bone and brain metastasis, possibly by enhancing the seeding of tumor cells to the target organ endothelium. Induces chemoresistance (582 aa)
**Q16643**	DREB	Drebrin OS=Homo sapiens OX=9606 GN=DBN1 PE=1 SV=4	Drebrins might play some role in cell migration, extension of neuronal processes and plasticity of dendrites. Required for actin polymerization at immunological synapses (IS) and for CXCR4 recruitment to IS (651 aa)
**Q8NBL1**	PGLT1	Protein O-glucosyltransferase 1 OS=Homo sapiens OX=9606 GN=POGLUT1 PE=1 SV=1	Dual specificity glycosyltransferase that catalyzes the transfer of glucose and xylose from UDP-glucose and UDP-xylose, respectively, to a serine residue found in the consensus sequence of C-X-S-X-P-C. Specifically targets extracellular EGF repeats of protein such as CRB2, F7, F9 and NOTCH2. Acts as a positive regulator of Notch signaling by mediating O-glucosylation of Notch, leading to regulate muscle development. Notch glucosylation does not affect Notch ligand binding. Required during early development to promote gastrulation- acts by mediating O- g [...] (392 aa)
**Q99496**	RING2	E3 ubiquitin-protein ligase RING2 OS=Homo sapiens OX=9606 GN=RNF2 PE=1 SV=1	E3 ubiquitin-protein ligase that mediates monoubiquitination of 'Lys-119' of histone H2A (H2AK119Ub), thereby playing a central role in histone code and gene regulation. H2AK119Ub gives a specific tag for epigenetic transcriptional repression and participates in X chromosome inactivation of female mammals. May be involved in the initiation of both imprinted and random X inactivation (By similarity). Essential component of a Polycomb group (PcG) multiprotein PRC1-like complex, a complex class required to maintain the transcriptionally repressive state [...] (336 aa)

**Q9BRZ2**	TRI56	E3 ubiquitin-protein ligase TRIM56 OS=Homo sapiens OX=9606 GN=TRIM56 PE=1 SV=3	E3 ubiquitin-protein ligase that plays a key role in innate antiviral immunity. In response to pathogen- and host-derived double-stranded DNA (dsDNA), targets TMEM173/STING to ’Lys-63’-linked ubiquitination, thereby promoting its homodimerization, a step required for the production of type I interferon IFN-beta (By similarity). Independently of its E3 ubiquitin ligase activity, positive regulator of TLR3 signaling. Potentiates extracellular double stranded RNA (dsRNA)-induced expression of IFNB1 and interferon-stimulated genes ISG15, IFIT1/ISG56, CXC [...] (755 aa)
**Q13191**	CBLB	E3 ubiquitin-protein ligase CBL-B OS=Homo sapiens OX=9606 GN=CBLB PE=1 SV=2	E3 ubiquitin-protein ligase which accepts ubiquitin from specific E2 ubiquitin-conjugating enzymes, and transfers it to substrates, generally promoting their degradation by the proteasome. Negatively regulates TCR (T-cell receptor), BCR (B- cell receptor) and FCER1 (high affinity immunoglobulin epsilon receptor) signal transduction pathways. In naive T-cells, inhibits VAV1 activation upon TCR engagement and imposes a requirement for CD28 costimulation for proliferation and IL-2 production. Also acts by promoting PIK3R1/p85 ubiquitination, which impair [...] (982 aa)
**Q12899**	TRI26	Tripartite motif-containing protein 26 OS=Homo sapiens OX=9606 GN=TRIM26 PE=1 SV=1	E3 ubiquitin-protein ligase which regulates the IFN-beta production and antiviral response downstream of various DNA- encoded pattern-recognition receptors (PRRs). Promotes nuclear IRF3 ubiquitination and proteasomal degradation. Bridges together TBK1 and NEMO during the innate response to viral infection leading to the activation of TBK1
**P19474**	RO52	E3 ubiquitin-protein ligase TRIM21 OS=Homo sapiens OX=9606 GN=TRIM21 PE=1 SV=1	E3 ubiquitin-protein ligase whose activity is dependent on E2 enzymes, UBE2D1, UBE2D2, UBE2E1 and UBE2E2. Forms a ubiquitin ligase complex in cooperation with the E2 UBE2D2 that is used not only for the ubiquitination of USP4 and IKBKB but also for its self-ubiquitination. Component of cullin-RING-based SCF (SKP1-CUL1-F-box protein) E3 ubiquitin-protein ligase complexes such as SCF(SKP2)-like complexes. A TRIM21-containing SCF(SKP2)- like complex is shown to mediate ubiquitination of CDKN1B (’Thr- 187’ phosphorylated-form), thereby promoting its degr [...] (475 aa)

**P20042**	IF2B	Eukaryotic translation initiation factor 2 subunit 2 OS=Homo sapiens OX=9606 GN=EIF2S2 PE=1 SV=2	eIF-2 functions in the early steps of protein synthesis by forming a ternary complex with GTP and initiator tRNA. This complex binds to a 40S ribosomal subunit, followed by mRNA binding to form a 43S preinitiation complex. Junction of the 60S ribosomal subunit to form the 80S initiation complex is preceded by hydrolysis of the GTP bound to eIF-2 and release of an eIF-2-GDP binary complex. In order for eIF-2 to recycle and catalyze another round of initiation, the GDP bound to eIF-2 must exchange with GTP by way of a reaction catalyz [...] (333 aa)
**P28370**	SMCA1	Probable global transcription activator SNF2L1 OS=Homo sapiens OX=9606 GN=SMARCA1 PE=1 SV=2	Energy-transducing component of NURF (nucleosome- remodeling factor) and CERF (CECR2-containing-remodeling factor) complexes. Both complexes facilitate the perturbation of chromatin structure in an ATP-dependent manner. Potentiates neurite outgrowth. May be involved in brain development by regulating En-1 and En-2 expression. May be involved in the development of luteal cells
**Q13442**	HAP28	28 kDa heat- and acid-stable phosphoprotein OS=Homo sapiens OX=9606 GN=PDAP1 PE=1 SV=1	Enhances PDGFA-stimulated cell growth in fibroblasts, but inhibits the mitogenic effect of PDGFB (181 aa)
**P02538**	K2C6A	Keratin, type II cytoskeletal 6A OS=Homo sapiens OX=9606 GN=KRT6A PE=1 SV=3	Epidermis-specific type I keratin involved in wound healing. Involved in the activation of follicular keratinocytes after wounding, while it does not play a major role in keratinocyte proliferation or migration. Participates in the regulation of epithelial migration by inhibiting the activity of SRC during wound repair
**P08779**	K1C16	Keratin, type I cytoskeletal 16 OS=Homo sapiens OX=9606 GN=KRT16 PE=1 SV=4	Epidermis-specific type I keratin that plays a key role in skin. Acts as a regulator of innate immunity in response to skin barrier breach- required for some inflammatory checkpoint for the skin barrier maintenance (473 aa)
**Q9UIF9**	BAZ2A	Bromodomain adjacent to zinc finger domain protein 2A OS=Homo sapiens OX=9606 GN=BAZ2A PE=1 SV=4	Essential component of the NoRC (nucleolar remodeling complex) complex, a complex that mediates silencing of a fraction of rDNA by recruiting histone-modifying enzymes and DNA methyltransferases, leading to heterochromatin formation and transcriptional silencing. In the complex, it plays a central role by being recruited to rDNA and by targeting chromatin modifying enzymes such as HDAC1, leading to repress RNA polymerase I transcription. Recruited to rDNA via its interaction with TTF1 and its ability to recognize and bind histone H [...] (1905 aa)

**P63208**	SKP1	S-phase kinase-associated protein 1 OS=Homo sapiens OX=9606 GN=SKP1 PE=1 SV=2	Essential component of the SCF (SKP1-CUL1-F-box protein) ubiquitin ligase complex, which mediates the ubiquitination of proteins involved in cell cycle progression, signal transduction and transcription. In the SCF complex, serves as an adapter that links the F-box protein to CUL1. The functional specificity of the SCF complex depends on the F-box protein as substrate recognition component. SCF(BTRC) and SCF(FBXW11) direct ubiquitination of CTNNB1 and participate in Wnt signaling. SCF(FBXW11) directs ubiquitination of phosphorylated NFKBIA. SCF(BTRC [...] (163 aa)
**Q9NVI7**	ATD3A	ATPase family AAA domain-containing protein 3A OS=Homo sapiens OX=9606 GN=ATAD3A PE=1 SV=2	Essential for mitochondrial network organization, mitochondrial metabolism and cell growth at organism and cellular level. May play an important role in mitochondrial protein synthesis. May also participate in mitochondrial DNA replication. May bind to mitochondrial DNA D-loops and contribute to nucleoid stability. Required for enhanced channeling of cholesterol for hormone-dependent steroidogenesis
**Q16774**	KGUA	Guanylate kinase OS=Homo sapiens OX=9606 GN=GUK1 PE=1 SV=2	Essential for recycling GMP and indirectly, cGMP (241 aa)
**P14635**	CCNB1	G2/mitotic-specific cyclin-B1 OS=Homo sapiens OX=9606 GN=CCNB1 PE=1 SV=1	Essential for the control of the cell cycle at the G2/M (mitosis) transition
**Q14690**	RRP5	Protein RRP5 homolog OS=Homo sapiens OX=9606 GN=PDCD11 PE=1 SV=3	Essential for the generation of mature 18S rRNA, specifically necessary for cleavages at sites A0, 1 and 2 of the 47S precursor. Directly interacts with U3 snoRNA
**P04843**	RPN1	Dolichyl-diphosphooligosaccharide--protein glycosyltransferase subunit 1 OS=Homo sapiens OX=9606 GN=RPN1 PE=1 SV=1	Essential subunit of the N-oligosaccharyl transferase (OST) complex which catalyzes the transfer of a high mannose oligosaccharide from a lipid-linked oligosaccharide donor to an asparagine residue within an Asn-X-Ser/Thr consensus motif in nascent polypeptide chains
**Q9GZR2**	REXO4	RNA exonuclease 4 OS=Homo sapiens OX=9606 GN=REXO4 PE=1 SV=2	Exonucleases (422 aa); REXO4 (REX4 Homolog, 3'-5' Exonuclease) is a Protein Coding gene. Gene Ontology (GO) annotations related to this gene include nucleic acid binding and DNA-binding transcription factor activity.

**Q86V81**	THOC4	THO complex subunit 4 OS=Homo sapiens OX=9606 GN=ALYREF PE=1 SV=3	Export adapter involved in nuclear export of spliced and unspliced mRNA. Binds mRNA which is thought to be transferred to the NXF1-NXT1 heterodimer for export (TAP/NFX1 pathway). Component of the TREX complex which is thought to couple mRNA transcription, processing and nuclear export, and specifically associates with spliced mRNA and not with unspliced pre-mRNA. TREX is recruited to spliced mRNAs by a transcription-independent mechanism, binds to mRNA upstream of the exon-junction complex (EJC) and is recruited in a splicing- and cap-dependent manner to a region [...] (264 aa)
**P46821**	MAP1B	Microtubule-associated protein 1B OS=Homo sapiens OX=9606 GN=MAP1B PE=1 SV=2	Facilitates tyrosination of alpha-tubulin in neuronal microtubules (By similarity). Phosphorylated MAP1B may play a role in the cytoskeletal changes that accompany neurite extension. Possibly MAP1B binds to at least two tubulin subunits in the polymer, and this bridging of subunits might be involved in nucleating microtubule polymerization and in stabilizing microtubules. Acts as a positive cofactor in DAPK1-mediated autophagic vesicle formation and membrane blebbing
**P47755**	CAZA2	F-actin-capping protein subunit alpha-2 OS=Homo sapiens OX=9606 GN=CAPZA2 PE=1 SV=3	F-actin-capping proteins bind in a Ca(2+)-independent manner to the fast growing ends of actin filaments (barbed end) thereby blocking the exchange of subunits at these ends. Unlike other capping proteins (such as gelsolin and severin), these proteins do not sever actin filaments (286 aa)

**P62861**	RS30	40S ribosomal protein S30 OS=Homo sapiens OX=9606 GN=FAU PE=1 SV=1	FAU, ubiquitin like and ribosomal protein S30 fusion (133 aa); This gene is the cellular homolog of the fox sequence in the Finkel-Biskis-Reilly murine sarcoma virus (FBR-MuSV). It encodes a fusion protein consisting of the ubiquitin-like protein fubi at the N terminus and ribosomal protein S30 at the C terminus. It has been proposed that the fusion protein is post-translationally processed to generate free fubi and free ribosomal protein S30. Fubi is a member of the ubiquitin family, and ribosomal protein S30 belongs to the S30E family of ribosomal proteins. Whereas the function of fubi is currently unknown, ribosomal protein S30 is a component of the 40S subunit of the cytoplasmic ribosome and displays antimicrobial activity. Pseudogenes derived from this gene are present in the genome. Similar to ribosomal protein S30, ribosomal proteins S27a and L40 are synthesized as fusion proteins with ubiquitin.
**Q5VST9**	OBSCN	Obscurin OS=Homo sapiens OX=9606 GN=OBSCN PE=1 SV=3	Structural component of striated muscles which plays a role in myofibrillogenesis. Probably involved in the assembly of myosin into sarcomeric A bands in striated muscle (PubMed:11448995, PubMed:16205939). Has serine/threonine protein kinase activity and phosphorylates N-cadherin CDH2 and sodium/potassium-transporting ATPase subunit ATP1B1
**Q9UHD8**	SEPT9	Septin-9 OS=Homo sapiens OX=9606 GN=SEPT9 PE=1 SV=2	Filament-forming cytoskeletal GTPase (By similarity). May play a role in cytokinesis (Potential). May play a role in the internalization of 2 intracellular microbial pathogens, Listeria monocytogenes and Shigella flexneri
**Q00688**	FKBP3	Peptidyl-prolyl cis-trans isomerase FKBP3 OS=Homo sapiens OX=9606 GN=FKBP3 PE=1 SV=1	FK506- and rapamycin-binding proteins (FKBPs) constitute a family of receptors for the two immunosuppressants which inhibit T-cell proliferation by arresting two distinct cytoplasmic signal transmission pathways. PPIases accelerate the folding of proteins
**Q96DT5**	DYH11	Dynein heavy chain 11, axonemal OS=Homo sapiens OX=9606 GN=DNAH11 PE=1 SV=4	Force generating protein of respiratory cilia. Produces force towards the minus ends of microtubules. Dynein has ATPase activity
**P54136**	SYRC	Arginine--tRNA ligase, cytoplasmic OS=Homo sapiens OX=9606 GN=RARS PE=1 SV=2	Forms part of a macromolecular complex that catalyzes the attachment of specific amino acids to cognate tRNAs during protein synthesis. Modulates the secretion of AIMP1 and may be involved in generation of the inflammatory cytokine EMAP2 from AIMP1

**Q7KZF4**	SND1	Staphylococcal nuclease domain-containing protein 1 OS=Homo sapiens OX=9606 GN=SND1 PE=1 SV=1	Functions as a bridging factor between STAT6 and the basal transcription factor. Plays a role in PIM1 regulation of MYB activity. Functions as a transcriptional coactivator for the Epstein-Barr virus nuclear antigen 2 (EBNA2)
**Q04721**	NOTC2	Neurogenic locus notch homolog protein 2 OS=Homo sapiens OX=9606 GN=NOTCH2 PE=1 SV=3	Functions as a receptor for membrane-bound ligands Jagged1, Jagged2 and Delta1 to regulate cell-fate determination. Upon ligand activation through the released notch intracellular domain (NICD) it forms a transcriptional activator complex with RBPJ/RBPSUH and activates genes of the enhancer of split locus. Affects the implementation of differentiation, proliferation and apoptotic programs (By similarity). Involved in bone remodeling and homeostasis. In collaboration with RELA/p65 enhances NFATc1 promoter activity and positively regulates RANKL- [...] (2471 aa)
**Q6P2Q9**	PRP8	Pre-mRNA-processing-splicing factor 8 OS=Homo sapiens OX=9606 GN=PRPF8 PE=1 SV=2	Functions as a scaffold that mediates the ordered assembly of spliceosomal proteins and snRNAs. Required for the assembly of the U4/U6-U5 tri-snRNP complex. Functions as scaffold that positions spliceosomal U2, U5 and U6 snRNAs at splice sites on pre-mRNA substrates, so that splicing can occur. Interacts with both the 5’ and the 3’ splice site (2335 aa)
**P52926**	HMGA2	High mobility group protein HMGI-C OS=Homo sapiens OX=9606 GN=HMGA2 PE=1 SV=1	Functions as a transcriptional regulator. Functions in cell cycle regulation through CCNA2. Plays an important role in chromosome condensation during the meiotic G2/M transition of spermatocytes. Plays a role in postnatal myogenesis, is involved in satellite cell activation (By similarity)
**Q14258**	TRI25	E3 ubiquitin/ISG15 ligase TRIM25 OS=Homo sapiens OX=9606 GN=TRIM25 PE=1 SV=2	Functions as a ubiquitin E3 ligase and as an ISG15 E3 ligase. Involved in innate immune defense against viruses by mediating ubiquitination of DDX58. Mediates ’Lys-63’-linked polyubiquitination of the DDX58 N-terminal CARD-like region which is crucial for triggering the cytosolic signal transduction that leads to the production of interferons in response to viral infection. Promotes ISGylation of 14-3-3 sigma (SFN), an adapter protein implicated in the regulation of a large spectrum signaling pathway. Mediates estrogen action in various target organs. [...] (630 aa)

**O00505**	IMA4	Importin subunit alpha-4 OS=Homo sapiens OX=9606 GN=KPNA3 PE=1 SV=2	Functions in nuclear protein import as an adapter protein for nuclear receptor KPNB1. Binds specifically and directly to substrates containing either a simple or bipartite NLS motif. Docking of the importin/substrate complex to the nuclear pore complex (NPC) is mediated by KPNB1 through binding to nucleoporin FxFG repeats and the complex is subsequently translocated through the pore by an energy requiring, Ran- dependent mechanism. At the nucleoplasmic side of the NPC, Ran binds to importin-beta and the three components separate and importin-alpha and -beta ar [...] (521 aa)
**P52292**	IMA1	Importin subunit alpha-1 OS=Homo sapiens OX=9606 GN=KPNA2 PE=1 SV=1	Functions in nuclear protein import as an adapter protein for nuclear receptor KPNB1. Binds specifically and directly to substrates containing either a simple or bipartite NLS motif. Docking of the importin/substrate complex to the nuclear pore complex (NPC) is mediated by KPNB1 through binding to nucleoporin FxFG repeats and the complex is subsequently translocated through the pore by an energy requiring, Ran- dependent mechanism. At the nucleoplasmic side of the NPC, Ran binds to importin-beta and the three components separate and importin-alpha and -beta ar [...] (529 aa)
**P05198**	IF2A	Eukaryotic translation initiation factor 2 subunit 1 OS=Homo sapiens OX=9606 GN=EIF2S1 PE=1 SV=3	Functions in the early steps of protein synthesis by forming a ternary complex with GTP and initiator tRNA. This complex binds to a 40S ribosomal subunit, followed by mRNA binding to form a 43S pre-initiation complex. Junction of the 60S ribosomal subunit to form the 80S initiation complex is preceded by hydrolysis of the GTP bound to eIF-2 and release of an eIF-2- GDP binary complex. In order for eIF-2 to recycle and catalyze another round of initiation, the GDP bound to eIF-2 must exchange with GTP by way of a reaction catalyzed b [...] (315 aa)
**P53999**	TCP4	Activated RNA polymerase II transcriptional coactivator p15 OS=Homo sapiens OX=9606 GN=SUB1 PE=1 SV=3	General coactivator that functions cooperatively with TAFs and mediates functional interactions between upstream activators and the general transcriptional machinery. May be involved in stabilizing the multiprotein transcription complex. Binds single-stranded DNA. Also binds, in vitro, non-specifically to double-stranded DNA (ds DNA) (127 aa)
**P47897**	SYQ	Glutamine--tRNA ligase OS=Homo sapiens OX=9606 GN=QARS PE=1 SV=1	Glutamine--tRNA ligase. Plays a critical role in brain development

**P14618**	KPYM	Pyruvate kinase PKM OS=Homo sapiens OX=9606 GN=PKM PE=1 SV=4	Glycolytic enzyme that catalyzes the transfer of a phosphoryl group from phosphoenolpyruvate (PEP) to ADP, generating ATP. Stimulates POU5F1-mediated transcriptional activation. Plays a general role in caspase independent cell death of tumor cells. The ratio between the highly active tetrameric form and nearly inactive dimeric form determines whether glucose carbons are channeled to biosynthetic processes or used for glycolytic ATP production. The transition between the 2 forms contributes to the control of glycolysis and is important for tumor cell proliferation a [...] (531 aa)
**P21453**	S1PR1	Sphingosine 1-phosphate receptor 1 OS=Homo sapiens OX=9606 GN=S1PR1 PE=1 SV=2	G-protein coupled receptor for the bioactive lysosphingolipid sphingosine 1-phosphate (S1P) that seems to be coupled to the G(i) subclass of heteromeric G proteins. Signaling leads to the activation of RAC1, SRC, PTK2/FAK1 and MAP kinases. Plays an important role in cell migration, probably via its role in the reorganization of the actin cytoskeleton and the formation of lamellipodia in response to stimuli that increase the activity of the sphingosine kinase SPHK1. Required for normal chemotaxis toward sphingosine 1-phosphate. Required for normal emb [...] (382 aa)
**Q96FS4**	SIPA1	Signal-induced proliferation-associated protein 1 OS=Homo sapiens OX=9606 GN=SIPA1 PE=1 SV=1	GTPase activator for the nuclear Ras-related regulatory proteins Rap1 and Rap2 in vitro, converting them to the putatively inactive GDP-bound state. Affects cell cycle progression (By similarity)
**P62826**	RAN	GTP-binding nuclear protein Ran OS=Homo sapiens OX=9606 GN=RAN PE=1 SV=3	GTPase involved in nucleocytoplasmic transport, participating both to the import and the export from the nucleus of proteins and RNAs. Switches between a cytoplasmic GDP- and a nuclear GTP-bound state by nucleotide exchange and GTP hydrolysis. Nuclear import receptors such as importin beta bind their substrates only in the absence of GTP-bound RAN and release them upon direct interaction with GTP-bound RAN while export receptors behave in the opposite way. Thereby, RAN controls cargo loading and release by transport receptors in the proper compartment a [...] (216 aa)
**Q13823**	NOG2	Nucleolar GTP-binding protein 2 OS=Homo sapiens OX=9606 GN=GNL2 PE=1 SV=1	GTPase that associates with pre-60S ribosomal subunits in the nucleolus and is required for their nuclear export and maturation (731 aa)

**Q9NP61**	ARFG3	ADP-ribosylation factor GTPase-activating protein 3 OS=Homo sapiens OX=9606 GN=ARFGAP3 PE=1 SV=1	GTPase-activating protein (GAP) for ADP ribosylation factor 1 (ARF1). Hydrolysis of ARF1-bound GTP may lead to dissociation of coatomer from Golgi-derived membranes to allow fusion with target membranes
**Q8N6H7**	ARFG2	ADP-ribosylation factor GTPase-activating protein 2 OS=Homo sapiens OX=9606 GN=ARFGAP2 PE=1 SV=1	GTPase-activating protein (GAP) for ADP ribosylation factor 1 (ARF1). Implicated in coatomer-mediated protein transport between the Golgi complex and the endoplasmic reticulum. Hydrolysis of ARF1-bound GTP may lead to dissociation of coatomer from Golgi-derived membranes to allow fusion with target membranes
**Q6ZS11**	RINL	Ras and Rab interactor-like protein OS=Homo sapiens OX=9606 GN=RINL PE=2 SV=2	Guanine nucleotide exchange factor (GEF) for RAB5A and RAB22A that activates RAB5A and RAB22A by exchanging bound GDP for free GTP. Plays a role in endocytosis via its role in activating Rab family members (By similarity)
**Q12774**	ARHG5	Rho guanine nucleotide exchange factor 5 OS=Homo sapiens OX=9606 GN=ARHGEF5 PE=1 SV=3	Guanine nucleotide exchange factor which activates Rho GTPases. Strongly activates RHOA. Also strongly activates RHOB, weakly activates RHOC and RHOG and shows no effect on RHOD, RHOV, RHOQ or RAC1 (By similarity). Involved in regulation of cell shape and actin cytoskeletal organization. Plays a role in actin organization by generating a loss of actin stress fibers and the formation of membrane ruffles and filopodia. Required for SRC-induced podosome formation (By similarity). Involved in positive regulation of immature dendritic cell migration [...] (1597 aa)
**P18754**	RCC1	Regulator of chromosome condensation OS=Homo sapiens OX=9606 GN=RCC1 PE=1 SV=1	Guanine-nucleotide releasing factor that promotes the exchange of Ran-bound GDP by GTP. Involved in the regulation of onset of chromosome condensation in the S phase. Binds both to the nucleosomes and double-stranded DNA. RCC1-Ran complex (together with other proteins) acts as a component of a signal transmission pathway that detects unreplicated DNA. Plays a key role in nucleo- cytoplasmic transport, mitosis and nuclear-envelope assembly (452 aa)

**O43684**	BUB3	Mitotic checkpoint protein BUB3 OS=Homo sapiens OX=9606 GN=BUB3 PE=1 SV=1	Has a dual function in spindle-assembly checkpoint signaling and in promoting the establishment of correct kinetochore-microtubule (K-MT) attachments. Promotes the formation of stable end-on bipolar attachments. Necessary for kinetochore localization of BUB1. Regulates chromosome segregation during oocyte meiosis. The BUB1/BUB3 complex plays a role in the inhibition of anaphase-promoting complex or cyclosome (APC/C) when spindle-assembly checkpoint is activated and inhibits the ubiquitin ligase activity of APC/C by phosphorylating its activator CDC20. T [...] (328 aa)
**Q13523**	PRP4B	Serine/threonine-protein kinase PRP4 homolog OS=Homo sapiens OX=9606 GN=PRPF4B PE=1 SV=3	Has a role in pre-mRNA splicing. Phosphorylates SF2/ASF (1007 aa)
**P07477**	TRY1	Trypsin-1 OS=Homo sapiens OX=9606 GN=PRSS1 PE=1 SV=1	Has activity against the synthetic substrates Boc-Phe-Ser-Arg-Mec, Boc-Leu-Thr-Arg-Mec, Boc-Gln-Ala-Arg-Mec and Boc-Val- Pro-Arg-Mec. The single-chain form is more active than the two- chain form against all of these substrates
**P04406**	G3P	Glyceraldehyde-3-phosphate dehydrogenase OS=Homo sapiens OX=9606 GN=GAPDH PE=1 SV=3	Has both glyceraldehyde-3-phosphate dehydrogenase and nitrosylase activities, thereby playing a role in glycolysis and nuclear functions, respectively. Participates in nuclear events including transcription, RNA transport, DNA replication and apoptosis. Nuclear functions are probably due to the nitrosylase activity that mediates cysteine S-nitrosylation of nuclear target proteins such as SIRT1, HDAC2 and PRKDC. Modulates the organization and assembly of the cytoskeleton. Facilitates the CHP1-dependent microtubule and membrane associations throu [...] (335 aa)
**Q8TDD1**	DDX54	ATP-dependent RNA helicase DDX54 OS=Homo sapiens OX=9606 GN=DDX54 PE=1 SV=2	Has RNA-dependent ATPase activity. Represses the transcriptional activity of nuclear receptors
**O60264**	SMCA5	SWI/SNF-related matrix-associated actin-dependent regulator of chromatin subfamily A member 5 OS=Homo sapiens OX=9606 GN=SMARCA5 PE=1 SV=1	Helicase that possesses intrinsic ATP-dependent nucleosome-remodeling activity. Complexes containing SMARCA5 are capable of forming ordered nucleosome arrays on chromatin; this may require intact histone H4 tails. Also required for replication of pericentric heterochromatin in S-phase specifically in conjunction with BAZ1A. Probably plays a role in repression of polI dependent transcription of the rDNA locus, through the recruitment of the SIN3/HDAC1 corepressor complex to the rDNA promoter. [...] (1052 aa)

**Q15477**	SKIV2	Helicase SKI2W OS=Homo sapiens OX=9606 GN=SKIV2L PE=1 SV=3	Helicase; Helicase; has ATPase activity. Component of the SKI complex which is thought to be involved in exosome-mediated RNA decay and associates with transcriptionally active genes in a manner dependent on PAF1 complex (PAF1C).
**P51858**	HDGF	Hepatoma-derived growth factor OS=Homo sapiens OX=9606 GN=HDGF PE=1 SV=1	Heparin-binding protein, with mitogenic activity for fibroblasts. Acts as a transcriptional repressor
**P22626**	ROA2	Heterogeneous nuclear ribonucleoproteins A2/B1 OS=Homo sapiens OX=9606 GN=HNRNPA2B1 PE=1 SV=2	Heterogeneous nuclear ribonucleoprotein (hnRNP) that associates with nascent pre-mRNAs, packaging them into hnRNP particles. The hnRNP particle arrangement on nascent hnRNA is non- random and sequence-dependent and serves to condense and stabilize the transcripts and minimize tangling and knotting. Packaging plays a role in various processes such as transcription, pre-mRNA processing, RNA nuclear export, subcellular location, mRNA translation and stability of mature mRNAs. Forms hnRNP particles with at least 20 other different hnRNP and h [...] (353 aa)
**O60506**	HNRPQ	Heterogeneous nuclear ribonucleoprotein Q OS=Homo sapiens OX=9606 GN=SYNCRIP PE=1 SV=2	Heterogenous nuclear ribonucleoprotein (hnRNP) implicated in mRNA processing mechanisms. Component of the CRD- mediated complex that promotes MYC mRNA stability. Isoform 1, isoform 2 and isoform 3 are associated in vitro with pre-mRNA, splicing intermediates and mature mRNA protein complexes. Isoform 1 binds to apoB mRNA AU-rich sequences. Isoform 1 is part of the APOB mRNA editosome complex and may modulate the postranscriptional C to U RNA-editing of the APOB mRNA through either by binding to A1CF (APOBEC1 complementation factor), to APOBEC1 [...] (623 aa)
**Q07065**	CKAP4	Cytoskeleton-associated protein 4 OS=Homo sapiens OX=9606 GN=CKAP4 PE=1 SV=2	High-affinity epithelial cell surface receptor for APF (602 aa); Mediates the anchoring of the endoplasmic reticulum to microtubules.
**Q9BQ67**	GRWD1	Glutamate-rich WD repeat-containing protein 1 OS=Homo sapiens OX=9606 GN=GRWD1 PE=1 SV=1	Histone binding-protein that regulates chromatin dynamics and minichromosome maintenance (MCM) loading at replication origins, possibly by promoting chromatin openness

**Q8NB78**	KDM1B	Lysine-specific histone demethylase 1B OS=Homo sapiens OX=9606 GN=KDM1B PE=1 SV=3	Histone demethylase that demethylates ’Lys-4’ of histone H3, a specific tag for epigenetic transcriptional activation, thereby acting as a corepressor. Required for de novo DNA methylation of a subset of imprinted genes during oogenesis. Acts by oxidizing the substrate by FAD to generate the corresponding imine that is subsequently hydrolyzed. Demethylates both mono- and di-methylated ’Lys-4’ of histone H3. Has no effect on tri- methylated ’Lys-4’, mono-, di- or tri-methylated ’Lys-9’, mono-, di- or tri-methylated ’Lys-27’, mono-, di- or tri-meth [...] (590 aa)
**P10412**	H14	Histone H1.4 OS=Homo sapiens OX=9606 GN=HIST1H1E PE=1 SV=2	Histone H1 protein binds to linker DNA between nucleosomes forming the macromolecular structure known as the chromatin fiber. Histones H1 are necessary for the condensation of nucleosome chains into higher-order structured fibers. Acts also as a regulator of individual gene transcription through chromatin remodeling, nucleosome spacing and DNA methylation (By similarity) (219 aa)
**P16402**	H13	Histone H1.3 OS=Homo sapiens OX=9606 GN=HIST1H1D PE=1 SV=2	Histone H1 protein binds to linker DNA between nucleosomes forming the macromolecular structure known as the chromatin fiber. Histones H1 are necessary for the condensation of nucleosome chains into higher-order structured fibers. Acts also as a regulator of individual gene transcription through chromatin remodeling, nucleosome spacing and DNA methylation (By similarity) (221 aa)
**P16403**	H12	Histone H1.2 OS=Homo sapiens OX=9606 GN=HIST1H1C PE=1 SV=2	Histone H1 protein binds to linker DNA between nucleosomes forming the macromolecular structure known as the chromatin fiber. Histones H1 are necessary for the condensation of nucleosome chains into higher-order structured fibers. Acts also as a regulator of individual gene transcription through chromatin remodeling, nucleosome spacing and DNA methylation (By similarity) (213 aa)
**Q9NR48**	ASH1L	Histone-lysine N-methyltransferase ASH1L OS=Homo sapiens OX=9606 GN=ASH1L PE=1 SV=2	Histone methyltransferase specifically methylating ’Lys- 36’ of histone H3 (H3K36me)

**Q96KQ7**	EHMT2	Histone-lysine N-methyltransferase EHMT2 OS=Homo sapiens OX=9606 GN=EHMT2 PE=1 SV=3	Histone methyltransferase that specifically mono- and dimethylates 'Lys-9' of histone H3 (H3K9me1 and H3K9me2, respectively) in euchromatin. H3K9me represents a specific tag for epigenetic transcriptional repression by recruiting HP1 proteins to methylated histones. Also mediates monomethylation of 'Lys-56' of histone H3 (H3K56me1) in G1 phase, leading to promote interaction between histone H3 and PCNA and regulating DNA replication. Also weakly methylates 'Lys-27' of histone H3 (H3K27me). Also required for DNA methylation, the histone methyltr [...] (1233 aa)
**Q92522**	H1X	Histone H1x OS=Homo sapiens OX=9606 GN=H1FX PE=1 SV=1	Histones H1 are necessary for the condensation of nucleosome chains into higher-order structures (213 aa)
**P07305**	H10	Histone H1.0 OS=Homo sapiens OX=9606 GN=H1F0 PE=1 SV=3	Histones H1 are necessary for the condensation of nucleosome chains into higher-order structures. The H1F0 histones are found in cells that are in terminal stages of differentiation or that have low rates of cell division (194 aa)
**P17096**	HMGA1	High mobility group protein HMG-I/HMG-Y OS=Homo sapiens OX=9606 GN=HMGA1 PE=1 SV=3	HMG-I/Y bind preferentially to the minor groove of A+T rich regions in double-stranded DNA. It is suggested that these proteins could function in nucleosome phasing and in the 3'-end processing of mRNA transcripts. They are also involved in the transcription regulation of genes containing, or in close proximity to A+T-rich regions
**P29372**	3MG	DNA-3-methyladenine glycosylase OS=Homo sapiens OX=9606 GN=MPG PE=1 SV=3	Hydrolysis of the deoxyribose N-glycosidic bond to excise 3-methyladenine, and 7-methylguanine from the damaged DNA polymer formed by alkylation lesions (298 aa)
**Q9NTK5**	OLA1	Obg-like ATPase 1 OS=Homo sapiens OX=9606 GN=OLA1 PE=1 SV=2	Hydrolyzes ATP, and can also hydrolyze GTP with lower efficiency. Has lower affinity for GTP
**P19525**	E2AK2	Interferon-induced, double-stranded RNA-activated protein kinase OS=Homo sapiens OX=9606 GN=EIF2AK2 PE=1 SV=2	IFN-induced dsRNA-dependent serine/threonine-protein kinase which plays a key role in the innate immune response to viral infection and is also involved in the regulation of signal transduction, apoptosis, cell proliferation and differentiation. Exerts its antiviral activity on a wide range of DNA and RNA viruses including hepatitis C virus (HCV), hepatitis B virus (HBV), measles virus (MV) and herpes simplex virus 1 (HHV-1). Inhibits viral replication via phosphorylation of the alpha subunit of eukaryotic initiation fac [...] (551 aa)
**Q9BRT6**	LLPH	Protein LLP homolog OS=Homo sapiens OX=9606 GN=LLPH PE=1 SV=1	In hippocampal neurons, regulates dendritic and spine growth and synaptic transmission (129 aa)

**Q12955**	ANK3	Ankyrin-3 OS=Homo sapiens OX=9606 GN=ANK3 PE=1 SV=3	In skeletal muscle, required for costamere localization of DMD and betaDAG1 (By similarity). Membrane-cytoskeleton linker. May participate in the maintenance/targeting of ion channels and cell adhesion molecules at the nodes of Ranvier and axonal initial segments. Regulates KCNA1 channel activity in function of dietary Mg(2+) levels, and thereby contributes to the regulation of renal Mg(2+) reabsorption
**Q12972**	PP1R8	Nuclear inhibitor of protein phosphatase 1 OS=Homo sapiens OX=9606 GN=PPP1R8 PE=1 SV=2	Inhibitor subunit of the major nuclear protein phosphatase-1 (PP-1). It has RNA-binding activity but does not cleave RNA and may target PP-1 to RNA-associated substrates. May also be involved in pre-mRNA splicing. Binds DNA and might act as a transcriptional repressor. Seems to be required for cell proliferation (351 aa)
**P43487**	RANG	Ran-specific GTPase-activating protein OS=Homo sapiens OX=9606 GN=RANBP1 PE=1 SV=1	Inhibits GTP exchange on Ran. Forms a Ran-GTP-RANBP1 trimeric complex. Increase GTP hydrolysis induced by the Ran GTPase activating protein RANGAP1. May act in an intracellular signaling pathway which may control the progression through the cell cycle by regulating the transport of protein and nucleic acids across the nuclear membrane (278 aa)
**P25685**	DNJB1	DnaJ homolog subfamily B member 1 OS=Homo sapiens OX=9606 GN=DNAJB1 PE=1 SV=4	Interacts with HSP70 and can stimulate its ATPase activity. Stimulates the association between HSC70 and HIP. Negatively regulates heat shock-induced HSF1 transcriptional activity during the attenuation and recovery phase period of the heat shock response. Stimulates ATP hydrolysis and the folding of unfolded proteins mediated by HSPA1A/B (in vitro) (340 aa)
**Q96P16**	RPR1A	Regulation of nuclear pre-mRNA domain-containing protein 1A OS=Homo sapiens OX=9606 GN=RPRD1A PE=1 SV=1	Interacts with phosphorylated C-terminal heptapeptide repeat domain (CTD) of the largest RNA polymerase II subunit POLR2A, and participates in dephosphorylation of the CTD by RPAP2. May act as a negative regulator of cyclin-D1 (CCND1) and cyclin-E (CCNE1) in the cell cycle (312 aa)

**Q9NQG5**	RPR1B	Regulation of nuclear pre-mRNA domain-containing protein 1B OS=Homo sapiens OX=9606 GN=RPRD1B PE=1 SV=1	Interacts with phosphorylated C-terminal heptapeptide repeat domain (CTD) of the largest RNA polymerase II subunit POLR2A, and participates in dephosphorylation of the CTD by RPAP2. Transcriptional regulator which enhances expression of CCND1. Promotes binding of RNA polymerase II to the CCDN1 promoter and to the termination region before the poly-A site but decreases its binding after the poly-A site. Prevents RNA polymerase II from reading through the 3' end termination site and may allow it to be recruited back to the prom [...] (326 aa)
**P78347**	GTF2I	General transcription factor II-I OS=Homo sapiens OX=9606 GN=GTF2I PE=1 SV=2	Interacts with the basal transcription machinery by coordinating the formation of a multiprotein complex at the C-FOS promoter, and linking specific signal responsive activator complexes. Promotes the formation of stable high-order complexes of SRF and PHOX1 and interacts cooperatively with PHOX1 to promote serum-inducible transcription of a reporter gene deriven by the C- FOS serum response element (SRE). Acts as a coregulator for USF1 by binding independently two promoter elements, a pyrimidine-rich initiator (Inr) and an upstream E-box. Required fo [...] (998 aa)
**Q9H0S4**	DDX47	Probable ATP-dependent RNA helicase DDX47 OS=Homo sapiens OX=9606 GN=DDX47 PE=1 SV=1	Involved in apoptosis. May have a role in rRNA processing and mRNA splicing. Associates with pre-rRNA precursors
**Q8IWX8**	CHERP	Calcium homeostasis endoplasmic reticulum protein OS=Homo sapiens OX=9606 GN=CHERP PE=1 SV=3	Involved in calcium homeostasis, growth and proliferation
**P35659**	DEK	Protein DEK OS=Homo sapiens OX=9606 GN=DEK PE=1 SV=1	Involved in chromatin organization (375 aa)
**Q14683**	SMC1A	Structural maintenance of chromosomes protein 1A OS=Homo sapiens OX=9606 GN=SMC1A PE=1 SV=2	Involved in chromosome cohesion during cell cycle and in DNA repair. Central component of cohesin complex. The cohesin complex is required for the cohesion of sister chromatids after DNA replication. The cohesin complex apparently forms a large proteinaceous ring within which sister chromatids can be trapped. At anaphase, the complex is cleaved and dissociates from chromatin, allowing sister chromatids to segregate. The cohesin complex may also play a role in spindle pole assembly during mitosis. Involved in DNA repair via its interacti [...] (1233 aa)

**Q15051**	IQCB1	IQ calmodulin-binding motif-containing protein 1 OS=Homo sapiens OX=9606 GN=IQCB1 PE=1 SV=1	Involved in ciliogenesis. The function in an early step in cilia formation depends on its association with CEP290/NPHP6. Involved in regulation of the BBSome complex integrity, specifically for presence of BBS2 and BBS5 in the complex, and in ciliary targeting of selected BBSome cargos. May play a role in controlling entry of the BBSome complex to cilia possibly implicating CEP290/NPHP6
**P51610**	HCFC1	Host cell factor 1 OS=Homo sapiens OX=9606 GN=HCFC1 PE=1 SV=2	Involved in control of the cell cycle. Also antagonizes transactivation by ZBTB17 and GABP2; represses ZBTB17 activation of the p15(INK4b) promoter and inhibits its ability to recruit p300. Coactivator for EGR2 and GABP2. Tethers the chromatin modifying Set1/Ash2 histone H3 'Lys-4' methyltransferase (H3K4me) and Sin3 histone deacetylase (HDAC) complexes (involved in the activation and repression of transcription, respectively) together. Component of a THAP1/THAP3-HCFC1-OGT complex that is required for the regulation of the transcriptional activity of RRM1. As part o [...] (2035 aa)
**P06748**	NPM	Nucleophosmin OS=Homo sapiens OX=9606 GN=NPM1 PE=1 SV=2	Involved in diverse cellular processes such as ribosome biogenesis, centrosome duplication, protein chaperoning, histone assembly, cell proliferation, and regulation of tumor suppressors p53/TP53 and ARF. Binds ribosome presumably to drive ribosome nuclear export. Associated with nucleolar ribonucleoprotein structures and bind single-stranded nucleic acids. Acts as a chaperonin for the core histones H3, H2B and H4. Stimulates APEX1 endonuclease activity on apurinic/apyrimidinic (AP) double- stranded DNA but inhibits APEX1 endonuclease activity on AP single-stranded RNA. [...] (294 aa)
**Q9Y620**	RA54B	DNA repair and recombination protein RAD54B OS=Homo sapiens OX=9606 GN=RAD54B PE=1 SV=1	Involved in DNA repair and mitotic recombination. May play an active role in recombination processes in concert with other members of the RAD52 epistasis group

**O60870**	KIN17	DNA/RNA-binding protein KIN17 OS=Homo sapiens OX=9606 GN=KIN PE=1 SV=2	Involved in DNA replication and the cellular response to DNA damage. May participate in DNA replication factories and create a bridge between DNA replication and repair mediated by high molecular weight complexes. May play a role in illegitimate recombination and regulation of gene expression. May participate in mRNA processing. Binds, in vitro, to double-stranded DNA. Also shown to bind preferentially to curved DNA in vitro and in vivo (By similarity). Binds via its C-terminal domain to RNA in vitro
**P18887**	XRCC1	DNA repair protein XRCC1 OS=Homo sapiens OX=9606 GN=XRCC1 PE=1 SV=2	Involved in DNA single-strand break repair by mediating the assembly of DNA break repair protein complexes. Probably during DNA repair, negatively regulates ADP-ribose levels by modulating ADP-ribosyltransferase PARP1 activity (633 aa)
**P51587**	BRCA2	Breast cancer type 2 susceptibility protein OS=Homo sapiens OX=9606 GN=BRCA2 PE=1 SV=3	Involved in double-strand break repair and/or homologous recombination. Binds RAD51 and potentiates recombinational DNA repair by promoting assembly of RAD51 onto single-stranded DNA (ssDNA). Acts by targeting RAD51 to ssDNA over double-stranded DNA, enabling RAD51 to displace replication protein-A (RPA) from ssDNA and stabilizing RAD51-ssDNA filaments by blocking ATP hydrolysis. Part of a PALB2-scaffolded HR complex containing RAD51C and which is thought to play a role in DNA repair by HR. May participate in S phase checkpoint activation. B [...] (3418 aa)
**Q9Y5X1**	SNX9	Sorting nexin-9 OS=Homo sapiens OX=9606 GN=SNX9 PE=1 SV=1	Involved in endocytosis and intracellular vesicle trafficking, both during interphase and at the end of mitosis. Required for efficient progress through mitosis and cytokinesis. Required for normal formation of the cleavage furrow at the end of mitosis. Plays a role in endocytosis via clathrin-coated pits, but also clathrin-independent, actin-dependent fluid-phase endocytosis. Plays a role in macropinocytosis. Promotes internalization of TNFR. Promotes degradation of EGFR after EGF signaling. Stimulates the GTPase activity of DNM1. Promotes DNM1 oligomerization. Promot [...] (595 aa)

**Q01831**	XPC	DNA repair protein complementing XP-C cells OS=Homo sapiens OX=9606 GN=XPC PE=1 SV=4	Involved in global genome nucleotide excision repair (GG-NER) by acting as damage sensing and DNA-binding factor component of the XPC complex. Has only a low DNA repair activity by itself which is stimulated by RAD23B and RAD23A. Has a preference to bind DNA containing a short single-stranded segment but not to damaged oligonucleotides. This feature is proposed to be related to a dynamic sensor function- XPC can rapidly screen duplex DNA for non-hydrogen-bonded bases by forming a transient nucleoprotein intermediate complex which matures int [...] (940 aa)
**Q9UN37**	VPS4A	Vacuolar protein sorting-associated protein 4A OS=Homo sapiens OX=9606 GN=VPS4A PE=1 SV=1	Involved in late steps of the endosomal multivesicular bodies (MVB) pathway. Recognizes membrane-associated ESCRT-III assemblies and catalyzes their disassembly, possibly in combination with membrane fission. Redistributes the ESCRT-III components to the cytoplasm for further rounds of MVB sorting. MVBs contain intraluminal vesicles (ILVs) that are generated by invagination and scission from the limiting membrane of the endosome and mostly are delivered to lysosomes enabling degradation of membrane proteins, such as stimulated growth fact [...] (437 aa)
**Q66PJ3**	AR6P4	ADP-ribosylation factor-like protein 6-interacting protein 4 OS=Homo sapiens OX=9606 GN=ARL6IP4 PE=1 SV=2	Involved in modulating alternative pre-mRNA splicing with either 5' distal site activation or preferential use of 3' proximal site. In case of infection by Herpes simplex virus (HSVI), may act as a splicing inhibitor of HSVI pre-mRNA (421 aa)
**Q9BZI7**	REN3B	Regulator of nonsense transcripts 3B OS=Homo sapiens OX=9606 GN=UPF3B PE=1 SV=1	Involved in nonsense-mediated decay (NMD) of mRNAs containing premature stop codons by associating with the nuclear exon junction complex (EJC) and serving as link between the EJC core and NMD machinery. Recruits UPF2 at the cytoplasmic side of the nuclear envelope and the subsequent formation of an UPF1-UPF2- UPF3 surveillance complex (including UPF1 bound to release factors at the stalled ribosome) is believed to activate NMD. In cooperation with UPF2 stimulates both ATPase and RNA helicase activities of UPF1. Binds spliced mRNA upstream of exon- [...] (483 aa)
**Q96KR1**	ZFR	Zinc finger RNA-binding protein OS=Homo sapiens OX=9606 GN=ZFR PE=1 SV=2	Involved in postimplantation and gastrulation stages of development. Involved in the nucleocytoplasmic shuttling of STAU2. Binds to DNA and RNA (By similarity) (1074 aa)

**O75533**	SF3B1	Splicing factor 3B subunit 1 OS=Homo sapiens OX=9606 GN=SF3B1 PE=1 SV=3	Involved in pre-mRNA splicing as a component of the splicing factor SF3B complex. SF3B complex is required for 'A' complex assembly formed by the stable binding of U2 snRNP to the branchpoint sequence (BPS) in pre-mRNA. Sequence independent binding of SF3A/SF3B complex upstream of the branch site is essential, it may anchor U2 snRNP to the pre-mRNA. May also be involved in the assembly of the 'E' complex. Belongs also to the minor U12-dependent spliceosome, which is involved in the splicing of rare class of nuclear pre-mRNA intron
**Q13435**	SF3B2	Splicing factor 3B subunit 2 OS=Homo sapiens OX=9606 GN=SF3B2 PE=1 SV=2	Involved in pre-mRNA splicing as a component of the splicing factor SF3B complex. SF3B complex is required for 'A' complex assembly formed by the stable binding of U2 snRNP to the branchpoint sequence (BPS) in pre-mRNA. Sequence independent binding of SF3A/SF3B complex upstream of the branch site is essential, it may anchor U2 snRNP to the pre-mRNA. May also be involved in the assembly of the 'E' complex. Belongs also to the minor U12-dependent spliceosome, which is involved in the splicing of rare class of nuclear pre-mRNA intron
**Q15393**	SF3B3	Splicing factor 3B subunit 3 OS=Homo sapiens OX=9606 GN=SF3B3 PE=1 SV=4	Involved in pre-mRNA splicing as a component of the splicing factor SF3B complex. SF3B complex is required for 'A' complex assembly formed by the stable binding of U2 snRNP to the branchpoint sequence (BPS) in pre-mRNA. Sequence independent binding of SF3A/SF3B complex upstream of the branch site is essential, it may anchor U2 snRNP to the pre-mRNA. May also be involved in the assembly of the 'E' complex. Belongs also to the minor U12-dependent spliceosome, which is involved in the splicing of rare class of nuclear pre-mRNA intron (1217 aa)
**O95400**	CD2B2	CD2 antigen cytoplasmic tail-binding protein 2 OS=Homo sapiens OX=9606 GN=CD2BP2 PE=1 SV=1	Involved in pre-mRNA splicing as component of the U5 snRNP complex that is involved in spliceosome assembly
**Q9NQT8**	KI13B	Kinesin-like protein KIF13B OS=Homo sapiens OX=9606 GN=KIF13B PE=1 SV=2	Involved in reorganization of the cortical cytoskeleton. Regulates axon formation by promoting the formation of extra axons. May be functionally important for the intracellular trafficking of MAGUKs and associated protein complexes
**Q15050**	RRS1	Ribosome biogenesis regulatory protein homolog OS=Homo sapiens OX=9606 GN=RRS1 PE=1 SV=2	Involved in ribosomal large subunit assembly. May regulate the localization of the 5S RNP/5S ribonucleoprotein particle to the nucleolus

**Q9H7B2**	RPF2	Ribosome production factor 2 homolog OS=Homo sapiens OX=9606 GN=RPF2 PE=1 SV=2	Involved in ribosomal large subunit assembly. May regulate the localization of the 5S RNP/5S ribonucleoprotein particle to the nucleolus (306 aa)
**P46087**	NOP2	Probable 28S rRNA (cytosine(4447)-C(5))-methyltransferase OS=Homo sapiens OX=9606 GN=NOP2 PE=1 SV=2	Involved in ribosomal large subunit assembly. S-adenosyl-L-methionine-dependent methyltransferase that specifically methylates the C(5) position of cytosine 4447 in 28S rRNA (Probable). May play a role in the regulation of the cell cycle and the increased nucleolar activity that is associated with the cell proliferation (Probable)
**Q9Y5Q8**	TF3C5	General transcription factor 3C polypeptide 5 OS=Homo sapiens OX=9606 GN=GTF3C5 PE=1 SV=2	Involved in RNA polymerase III-mediated transcription. Integral, tightly associated component of the DNA-binding TFIIIC2 subcomplex that directly binds tRNA and virus-associated RNA promoters
**O00512**	BCL9	B-cell CLL/lymphoma 9 protein OS=Homo sapiens OX=9606 GN=BCL9 PE=1 SV=4	Involved in signal transduction through the Wnt pathway. Promotes beta-catenin's transcriptional activity (By similarity)
**P17844**	DDX5	Probable ATP-dependent RNA helicase DDX5 OS=Homo sapiens OX=9606 GN=DDX5 PE=1 SV=1	Involved in the alternative regulation of pre-mRNA splicing; its RNA helicase activity is necessary for increasing tau exon 10 inclusion and occurs in a RBM4-dependent manner. Binds to the tau pre-mRNA in the stem-loop region downstream of exon 10. The rate of ATP hydrolysis is highly stimulated by single-stranded RNA. Involved in transcriptional regulation; the function is independent of the RNA helicase activity. Transcriptional coactivator for androgen receptor AR but probably not ESR1. Synergizes with DDX17 and SRA1 RNA to activate MYOD1 tr [...] (614 aa)
**P09874**	PARP1	Poly [ADP-ribose] polymerase 1 OS=Homo sapiens OX=9606 GN=PARP1 PE=1 SV=4	Involved in the base excision repair (BER) pathway, by catalyzing the poly(ADP-ribosyl)ation of a limited number of acceptor proteins involved in chromatin architecture and in DNA metabolism. This modification follows DNA damages and appears as an obligatory step in a detection/signaling pathway leading to the reparation of DNA strand breaks. Mediates the poly(ADP-ribosyl)ation of APLF and CHFR. Positively regulates the transcription of MTUS1 and negatively regulates the transcription of MTUS2/TIP150. With EEF1A1 and TXK, forms a complex that acts as a T [...] (1014 aa)
**Q9BZE4**	NOG1	Nucleolar GTP-binding protein 1 OS=Homo sapiens OX=9606 GN=GTPBP4 PE=1 SV=3	Involved in the biogenesis of the 60S ribosomal subunit

**O00567**	NOP56	Nucleolar protein 56 OS=Homo sapiens OX=9606 GN=NOP56 PE=1 SV=4	Involved in the early to middle stages of 60S ribosomal subunit biogenesis. Core component of box C/D small nucleolar ribonucleoprotein (snoRNP) particles. Required for the biogenesis of box C/D snoRNAs such U3, U8 and U14 snoRNAs
**Q9Y230**	RUVB2	RuvB-like 2 OS=Homo sapiens OX=9606 GN=RUVBL2 PE=1 SV=3	Involved in the endoplasmic reticulum (ER)-associated degradation (ERAD) pathway where it negatively regulates expression of ER stress response genes
**Q9NP79**	VTA1	Vacuolar protein sorting-associated protein VTA1 homolog OS=Homo sapiens OX=9606 GN=VTA1 PE=1 SV=1	Involved in the endosomal multivesicular bodies (MVB) pathway. MVBs contain intraluminal vesicles (ILVs) that are generated by invagination and scission from the limiting membrane of the endosome and mostly are delivered to lysosomes enabling degradation of membrane proteins, such as stimulated growth factor receptors, lysosomal enzymes and lipids. Thought to be a cofactor of VPS4A/B, which catalyzes disassembles membrane-associated ESCRT-III assemblies. Involved in the sorting and down-regulation of EGFR (By similarity). Involv [...] (307 aa)
**Q86VV8**	RTTN	Rotatin OS=Homo sapiens OX=9606 GN=RTTN PE=1 SV=3	Involved in the genetic cascade that governs left-right specification. Plays a role in the maintenance of a normal ciliary structure. Required for correct asymmetric expression of NODAL, LEFTY and PITX2
**P09651**	ROA1	Heterogeneous nuclear ribonucleoprotein A1 OS=Homo sapiens OX=9606 GN=HNRNPA1 PE=1 SV=5	Involved in the packaging of pre-mRNA into hnRNP particles, transport of poly(A) mRNA from the nucleus to the cytoplasm and may modulate splice site selection. May bind to specific miRNA hairpins
**Q8WWM7**	ATX2L	Ataxin-2-like protein OS=Homo sapiens OX=9606 GN=ATXN2L PE=1 SV=2	Involved in the regulation of stress granule and P-body formation
**P16949**	STMN1	Stathmin OS=Homo sapiens OX=9606 GN=STMN1 PE=1 SV=3	Involved in the regulation of the microtubule (MT) filament system by destabilizing microtubules. Prevents assembly and promotes disassembly of microtubules. Phosphorylation at Ser- 16 may be required for axon formation during neurogenesis. Involved in the control of the learned and innate fear (By similarity)
**P31942**	HNRH3	Heterogeneous nuclear ribonucleoprotein H3 OS=Homo sapiens OX=9606 GN=HNRNPH3 PE=1 SV=2	Involved in the splicing process and participates in early heat shock-induced splicing arrest. Due to their great structural variations the different isoforms may possess different functions in the splicing reaction

**Q13217**	DNJC3	DnaJ homolog subfamily C member 3 OS=Homo sapiens OX=9606 GN=DNAJC3 PE=1 SV=1	Involved in the unfolded protein response (UPR) during endoplasmic reticulum (ER) stress. Acts as a negative regulator of the EIF2AK4/GCN2 kinase activity by preventing the phosphorylation of eIF-2-alpha at 'Ser-52' and hence attenuating general protein synthesis under ER stress, hypothermic and amino acid starving stress conditions (By similarity). Co-chaperone of HSPA8/HSC70, it stimulates its ATPase activity. May inhibit both the autophosphorylation of EIF2AK2/PKR and the ability of EIF2AK2 to catalyze phosphorylation of the EIF2A. May inhibit EIF2 [...] (504 aa)
**O14497**	ARI1A	AT-rich interactive domain-containing protein 1A OS=Homo sapiens OX=9606 GN=ARID1A PE=1 SV=3	Involved in transcriptional activation and repression of select genes by chromatin remodeling (alteration of DNA-nucleosome topology). Component of SWI/SNF chromatin remodeling complexes that carry out key enzymatic activities, changing chromatin structure by altering DNA-histone contacts within a nucleosome in an ATP-dependent manner. Binds DNA non-specifically. Belongs to the neural progenitors-specific chromatin remodeling complex (npBAF complex) and the neuron-specific chromatin remodeling complex (nBAF complex). During neural devel [...] (2285 aa)
**P51532**	SMCA4	Transcription activator BRG1 OS=Homo sapiens OX=9606 GN=SMARCA4 PE=1 SV=2	Involved in transcriptional activation and repression of select genes by chromatin remodeling (alteration of DNA-nucleosome topology). Component of SWI/SNF chromatin remodeling complexes that carry out key enzymatic activities, changing chromatin structure by altering DNA-histone contacts within a nucleosome in an ATP-dependent manner. Component of the CREST-BRG1 complex, a multiprotein complex that regulates promoter activation by orchestrating a calcium-dependent release of a repressor complex and a recruitment of an activator complex. In resting neurons [...] (1647 aa)

**Q8TAQ2**	SMRC2	SWI/SNF complex subunit SMARCC2 OS=Homo sapiens OX=9606 GN=SMARCC2 PE=1 SV=1	Involved in transcriptional activation and repression of select genes by chromatin remodeling (alteration of DNA-nucleosome topology). Component of SWI/SNF chromatin remodeling complexes that carry out key enzymatic activities, changing chromatin structure by altering DNA-histone contacts within a nucleosome in an ATP-dependent manner. Can stimulate the ATPase activity of the catalytic subunit of these complexes. May be required for CoREST dependent repression of neuronal specific gene promoters in non-neuronal cells. Belongs to the neural progenitors-s [...] (1214 aa)
**Q969G3**	SMCE1	SWI/SNF-related matrix-associated actin-dependent regulator of chromatin subfamily E member 1 OS=Homo sapiens OX=9606 GN=SMARCE1 PE=1 SV=2	Involved in transcriptional activation and repression of select genes by chromatin remodeling (alteration of DNA-nucleosome topology). Component of SWI/SNF chromatin remodeling complexes that carry out key enzymatic activities, changing chromatin structure by altering DNA-histone contacts within a nucleosome in an ATP-dependent manner. Belongs to the neural progenitors- specific chromatin remodeling complex (npBAF complex) and the neuron-specific chromatin remodeling complex (nBAF complex). D [...] (411 aa)
**Q86U86**	PB1	Protein polybromo-1 OS=Homo sapiens OX=9606 GN=PBRM1 PE=1 SV=1	Involved in transcriptional activation and repression of select genes by chromatin remodeling (alteration of DNA-nucleosome topology). Required for the stability of the SWI/SNF chromatin remodeling complex SWI/SNF-B (PBAF). Acts as a negative regulator of cell proliferation
**P23396**	RS3	40S ribosomal protein S3 OS=Homo sapiens OX=9606 GN=RPS3 PE=1 SV=2	Involved in translation as a component of the 40S small ribosomal subunit. Has endonuclease activity and plays a role in repair of damaged DNA. Cleaves phosphodiester bonds of DNAs containing altered bases with broad specificity and cleaves supercoiled DNA more efficiently than relaxed DNA. Displays high binding affinity for 7,8-dihydro-8-oxoguanine (8-oxoG), a common DNA lesion caused by reactive oxygen species (ROS). Has also been shown to bind with similar affinity to intact and damaged DNA. Stimulates the N-glycosylase activity of the base excision protein [...] (259 aa)

**Q07021**	C1QBP	Complement component 1 Q subcomponent-binding protein, mitochondrial OS=Homo sapiens OX=9606 GN=C1QBP PE=1 SV=1	Is believed to be a multifunctional and multicompartmental protein involved in inflammation and infection processes, ribosome biogenesis, regulation of apoptosis, transcriptional regulation and pre-mRNA splicing. At the cell surface is thought to act as an endothelial receptor for plasma proteins of the complement and kallikrein-kinin cascades. Putative receptor for C1q
**Q8NE71**	ABCF1	ATP-binding cassette sub-family F member 1 OS=Homo sapiens OX=9606 GN=ABCF1 PE=1 SV=2	Isoform 2 is required for efficient Cap- and IRES-mediated mRNA translation initiation. Isoform 2 is not involved in the ribosome biogenesis
**P49916**	DNLI3	DNA ligase 3 OS=Homo sapiens OX=9606 GN=LIG3 PE=1 SV=2	Isoform 3 functions as heterodimer with DNA-repair protein XRCC1 in the nucleus and can correct defective DNA strand- break repair and sister chromatid exchange following treatment with ionizing radiation and alkylating agents. Isoform 1 is targeted to mitochondria, where it functions as DNA ligase in mitochondrial base-excision DNA repair (1009 aa)
**P13645**	K1C10	Keratin, type I cytoskeletal 10 OS=Homo sapiens OX=9606 GN=KRT10 PE=1 SV=6	Keratins, type I (584 aa); Plays a role in the establishment of the epidermal barrier on plantar skin.<br><br>(Microbial infection) Acts as a mediator of S.aureus adherence to desquamated nasal epithelial cells via clfB, and hence may play a role in nasal colonization.
**Q7Z794**	K2C1B	Keratin, type II cytoskeletal 1b OS=Homo sapiens OX=9606 GN=KRT77 PE=2 SV=3	Keratins, type II; Keratins are intermediate filament proteins responsible for the structural integrity of epithelial cells and are subdivided into epithelial keratins and hair keratins. This gene encodes an epithelial keratin that is expressed in the skin and eccrine sweat glands. The type II keratins are clustered in a region of chromosome 12q13.
**Q8N1N4**	K2C78	Keratin, type II cytoskeletal 78 OS=Homo sapiens OX=9606 GN=KRT78 PE=1 SV=2	Keratins, type II (520 aa); This gene is a member of the type II keratin gene family and encodes a protein with an intermediate filament domain. Keratins are the major structural proteins in epithelial cells, forming a cytoplasmic network of 10 to 12 nm wide intermediate filaments and creating a scaffold that gives cells the ability to withstand mechanical and non-mechanical stresses. The genes of the type II keratin family are located as a gene cluster at 12p13.13. Four pseudogenes of this gene family have been identified

**P13647**	K2C5	Keratin, type II cytoskeletal 5 OS=Homo sapiens OX=9606 GN=KRT5 PE=1 SV=3	Keratins, type II (590 aa); The protein encoded by this gene is a member of the keratin gene family. The type II cytokeratins consist of basic or neutral proteins which are arranged in pairs of heterotypic keratin chains coexpressed during differentiation of simple and stratified epithelial tissues. This type II cytokeratin is specifically expressed in the basal layer of the epidermis with family member KRT14. Mutations in these genes have been associated with a complex of diseases termed epidermolysis bullosa simplex. The type II cytokeratins are clustered in a region of chromosome 12q12-q13
**Q6Y7W6**	GGYF2	GRB10-interacting GYF protein 2 OS=Homo sapiens OX=9606 GN=GIGYF2 PE=1 SV=1	Key component of the 4EHP-GYF2 complex, a multiprotein complex that acts as a repressor of translation initiation. In 4EHP-GYF2 the complex, acts as a factor that bridges EIF4E2 to ZFP36/TTP, linking translation repression with mRNA decay (By similarity). May act cooperatively with GRB10 to regulate tyrosine kinase receptor signaling, including IGF1 and insulin receptors
**Q5T5Y3**	CAMP1	Calmodulin-regulated spectrin-associated protein 1 OS=Homo sapiens OX=9606 GN=CAMSAP1 PE=1 SV=2	Key microtubule-organizing protein that specifically binds the minus-end of non-centrosomal microtubules and regulates their dynamics and organization. Specifically recognizes growing microtubule minus-ends and stabilizes microtubules. Acts on free microtubule minus-ends that are not capped by microtubule- nucleating proteins or other factors and protects microtubule minus-ends from depolymerization. In contrast to CAMSAP2 and CAMSAP3, tracks along the growing tips of minus-end microtubules without significantly affecting the polymeri [...] (1602 aa)
**Q5UIP0**	RIF1	Telomere-associated protein RIF1 OS=Homo sapiens OX=9606 GN=RIF1 PE=1 SV=2	Key regulator of TP53BP1 that plays a key role in the repair of double-strand DNA breaks (DSBs) in response to DNA damage- acts by promoting non-homologous end joining (NHEJ)- mediated repair of DSBs. In response to DNA damage, interacts with ATM-phosphorylated TP53BP1. Interaction with TP53BP1 leads to dissociate the interaction between NUDT16L1/TIRR and TP53BP1, thereby unmasking the tandem Tudor-like domain of TP53BP1 and allowing recruitment to DNA DSBs. Once recruited to DSBs, RIF1 and TP53BP1 act by promoting NHEJ-mediated repair of DSBs. In the [...] (2472 aa)

**Q9ULG3**	K1257	Uncharacterized protein KIAA1257 OS=Homo sapiens OX=9606 GN=KIAA1257 PE=2 SV=2	KIAA1257 (KIAA1257) is a Protein Coding gene
**O43670**	ZN207	BUB3-interacting and GLEBS motif-containing protein ZNF207 OS=Homo sapiens OX=9606 GN=ZNF207 PE=1 SV=1	Kinetochore- and microtubule-binding protein that plays a key role in spindle assembly. ZNF207/BuGZ is mainly composed of disordered low-complexity regions and undergoes phase transition or coacervation to form temperature-dependent liquid droplets. Coacervation promotes microtubule bundling and concentrates tubulin, promoting microtubule polymerization and assembly of spindle and spindle matrix by concentrating its building blocks. Also acts as a regulator of mitotic chromosome alignment by mediating the stability and kinetoc [...] (494 aa)
**Q8N1G4**	LRC47	Leucine-rich repeat-containing protein 47 OS=Homo sapiens OX=9606 GN=LRRC47 PE=1 SV=1	LRRC47 (Leucine Rich Repeat Containing 47) is a Protein Coding gene. Gene Ontology (GO) annotations related to this gene include phenylalanine-tRNA ligase activity. An important paralog of this gene is FARSB.
**Q9UNF0**	PACN2	Protein kinase C and casein kinase substrate in neurons protein 2 OS=Homo sapiens OX=9606 GN=PACSIN2 PE=1 SV=2	Lipid-binding protein that is able to promote the tubulation of the phosphatidic acid-containing membranes it preferentially binds. Plays a role in intracellular vesicle- mediated transport. Involved in the endocytosis of cell-surface receptors like the EGF receptor, contributing to its internalization in the absence of EGF stimulus. May also play a role in the formation of caveolae at the cell membrane. Recruits DNM2 to caveolae, and thereby plays a role in caveola-mediated endocytosis
**P62269**	RS18	40S ribosomal protein S18 OS=Homo sapiens OX=9606 GN=RPS18 PE=1 SV=3	Located at the top of the head of the 40S subunit, it contacts several helices of the 18S rRNA
**Q96GA3**	LTV1	Protein LTV1 homolog OS=Homo sapiens OX=9606 GN=LTV1 PE=1 SV=1	LTV1 (LTV1 Ribosome Biogenesis Factor) is a Protein Coding gene. Diseases associated with LTV1 include Spinal Cord Disease. Among its related pathways are rRNA processing in the nucleus and cytosol and Gene Expression. An important paralog of this gene is ENSG00000280148.
**P15924**	DESP	Desmoplakin OS=Homo sapiens OX=9606 GN=DSP PE=1 SV=3	Major high molecular weight protein of desmosomes. Involved in the organization of the desmosomal cadherin- plakoglobin complexes into discrete plasma membrane domains and in the anchoring of intermediate filaments to the desmosomes

**P40926**	MDHM	Malate dehydrogenase, mitochondrial OS=Homo sapiens OX=9606 GN=MDH2 PE=1 SV=3	Malate dehydrogenase catalyzes the reversible oxidation of malate to oxaloacetate, utilizing the NAD/NADH cofactor system in the citric acid cycle. The protein encoded by this gene is localized to the mitochondria and may play pivotal roles in the malate-aspartate shuttle that operates in the metabolic coordination between cytosol and mitochondria. Several transcript variants encoding different isoforms have been found for this gene.
**Q06033**	ITIH3	Inter-alpha-trypsin inhibitor heavy chain H3 OS=Homo sapiens OX=9606 GN=ITIH3 PE=1 SV=2	May act as a carrier of hyaluronan in serum or as a binding protein between hyaluronan and other matrix protein, including those on cell surfaces in tissues to regulate the localization, synthesis and degradation of hyaluronan which are essential to cells undergoing biological processes (890 aa)
**Q14692**	BMS1	Ribosome biogenesis protein BMS1 homolog OS=Homo sapiens OX=9606 GN=BMS1 PE=1 SV=1	May act as a molecular switch during maturation of the 40S ribosomal subunit in the nucleolus (1282 aa)
**Q9Y6V0**	PCLO	Protein piccolo OS=Homo sapiens OX=9606 GN=PCLO PE=1 SV=5	May act as a scaffolding protein involved in the organization of synaptic active zones and in synaptic vesicle trafficking
**O95104**	SFR15	Splicing factor, arginine/serine-rich 15 OS=Homo sapiens OX=9606 GN=SCAF4 PE=1 SV=3	May act to physically and functionally link transcription and pre-mRNA processing
**Q9BQG0**	MBB1A	Myb-binding protein 1A OS=Homo sapiens OX=9606 GN=MYBBP1A PE=1 SV=2	May activate or repress transcription via interactions with sequence specific DNA-binding proteins. Repression may be mediated at least in part by histone deacetylase activity (HDAC activity). Acts as a corepressor and in concert with CRY1, represses the transcription of the core circadian clock component PER2. Preferentially binds to dimethylated histone H3 'Lys-9' (H3K9me2) on the PER2 promoter
**P08133**	ANXA6	Annexin A6 OS=Homo sapiens OX=9606 GN=ANXA6 PE=1 SV=3	May associate with CD21. May regulate the release of Ca(2+) from intracellular stores
**Q14320**	FA50A	Protein FAM50A OS=Homo sapiens OX=9606 GN=FAM50A PE=1 SV=2	May be a DNA-binding protein or transcriptional factor
**Q53T59**	H1BP3	HCLS1-binding protein 3 OS=Homo sapiens OX=9606 GN=HS1BP3 PE=1 SV=1	May be a modulator of IL-2 signaling (392 aa)
**Q6PL18**	ATAD2	ATPase family AAA domain-containing protein 2 OS=Homo sapiens OX=9606 GN=ATAD2 PE=1 SV=1	May be a transcriptional coactivator of the nuclear receptor ESR1 required to induce the expression of a subset of estradiol target genes, such as CCND1, MYC and E2F1. May play a role in the recruitment or occupancy of CREBBP at some ESR1 target gene promoters. May be required for histone hyperacetylation. Involved in the estrogen-induced cell proliferation and cell cycle progression of breast cancer cells

**Q9Y265**	RUVB1	RuvB-like 1 OS=Homo sapiens OX=9606 GN=RUVBL1 PE=1 SV=1	May be able to bind plasminogen at cell surface and enhance plasminogen activation
**Q9HB71**	CYBP	Calcyclin-binding protein OS=Homo sapiens OX=9606 GN=CACYBP PE=1 SV=2	May be involved in calcium-dependent ubiquitination and subsequent proteasomal degradation of target proteins. Probably serves as a molecular bridge in ubiquitin E3 complexes. Participates in the ubiquitin-mediated degradation of beta-catenin (CTNNB1) (228 aa)
**Q9UJS0**	CMC2	Calcium-binding mitochondrial carrier protein Aralar2 OS=Homo sapiens OX=9606 GN=SLC25A13 PE=1 SV=2	May be involved in cytochrome c oxidase biogenesis (79 aa)
**Q9ULV4**	COR1C	Coronin-1C OS=Homo sapiens OX=9606 GN=CORO1C PE=1 SV=1	May be involved in cytokinesis, motility, and signal transduction
**P40222**	TXLNA	Alpha-taxilin OS=Homo sapiens OX=9606 GN=TXLNA PE=1 SV=3	May be involved in intracellular vesicle traffic and potentially in calcium-dependent exocytosis in neuroendocrine cells
**P55209**	NP1L1	Nucleosome assembly protein 1-like 1 OS=Homo sapiens OX=9606 GN=NAP1L1 PE=1 SV=1	May be involved in modulating chromatin formation and contribute to regulation of cell proliferation
**P42166**	LAP2A	Lamina-associated polypeptide 2, isoform alpha OS=Homo sapiens OX=9606 GN=TMPO PE=1 SV=2	May be involved in the structural organization of the nucleus and in the post-mitotic nuclear assembly. Plays an important role, together with LMNA, in the nuclear anchorage of RB1
**O43583**	DENR	Density-regulated protein OS=Homo sapiens OX=9606 GN=DENR PE=1 SV=2	May be involved in the translation of target mRNAs by scanning and recognition of the initiation codon. Involved in translation initiation; promotes recruitmnet of aminoacetyled initiator tRNA to P site of 40S ribosomes. Can promote release of deacylated tRNA and mRNA from recycled 40S subunits following ABCE1-mediated dissociation of post-termination ribosomal complexes into subunits. Plays a role in the modulation of the translational profile of a subset of cancer-related mRNAs when recruited to the translational initiation complex by the oncogene MCTS1 (198 aa)
**Q5HYK9**	ZN667	Zinc finger protein 667 OS=Homo sapiens OX=9606 GN=ZNF667 PE=2 SV=2	May be involved in transcriptional regulation
**Q96ME7**	ZN512	Zinc finger protein 512 OS=Homo sapiens OX=9606 GN=ZNF512 PE=1 SV=2	May be involved in transcriptional regulation
**Q7Z3U7**	MON2	Protein MON2 homolog OS=Homo sapiens OX=9606 GN=MON2 PE=1 SV=3	May be required for traffic between late Golgi and early endosomes
**Q9BVP2**	GNL3	Guanine nucleotide-binding protein-like 3 OS=Homo sapiens OX=9606 GN=GNL3 PE=1 SV=2	May be required to maintain the proliferative capacity of stem cells. Stabilizes MDM2 by preventing its ubiquitination, and hence proteasomal degradation (By similarity)
**Q969S3**	ZN622	Zinc finger protein 622 OS=Homo sapiens OX=9606 GN=ZNF622 PE=1 SV=1	May behave as an activator of the bound transcription factor, MYBL2, and be involved in embryonic development (477 aa)

**Q9UGY1**	NOL12	Nucleolar protein 12 OS=Homo sapiens OX=9606 GN=NOL12 PE=1 SV=1	May bind to 28S rRNA
**Q9Y383**	LC7L2	Putative RNA-binding protein Luc7-like 2 OS=Homo sapiens OX=9606 GN=LUC7L2 PE=1 SV=2	May bind to RNA via its Arg/Ser-rich domain (458 aa)
**Q9H078**	CLPB	Caseinolytic peptidase B protein homolog OS=Homo sapiens OX=9606 GN=CLPB PE=1 SV=1	May function as a regulatory ATPase and be related to secretion/protein trafficking process
**Q9H7N4**	SFR19	Splicing factor, arginine/serine-rich 19 OS=Homo sapiens OX=9606 GN=SCAF1 PE=1 SV=3	May function in pre-mRNA splicing (1312 aa)
**Q9HC35**	EMAL4	Echinoderm microtubule-associated protein-like 4 OS=Homo sapiens OX=9606 GN=EML4 PE=1 SV=3	May modify the assembly dynamics of microtubules, such that microtubules are slightly longer, but more dynamic
**Q8WXX5**	DNJC9	DnaJ homolog subfamily C member 9 OS=Homo sapiens OX=9606 GN=DNAJC9 PE=1 SV=1	May play a role as co-chaperone of the Hsp70 family proteins HSPA1A, HSPA1B and HSPA8
**Q96DR8**	MUCL1	Mucin-like protein 1 OS=Homo sapiens OX=9606 GN=MUCL1 PE=1 SV=1	May play a role as marker for the diagnosis of metastatic breast cancer (90 aa)
**P61247**	RS3A	40S ribosomal protein S3a OS=Homo sapiens OX=9606 GN=RPS3A PE=1 SV=2	May play a role during erythropoiesis through regulation of transcription factor DDIT3
**Q9UQ80**	PA2G4	Proliferation-associated protein 2G4 OS=Homo sapiens OX=9606 GN=PA2G4 PE=1 SV=3	May play a role in a ERBB3-regulated signal transduction pathway. Seems be involved in growth regulation. Acts a corepressor of the androgen receptor (AR) and is regulated by the ERBB3 ligand neuregulin-1/heregulin (HRG). Inhibits transcription of some E2F1-regulated promoters, probably by recruiting histone acetylase (HAT) activity. Binds RNA. Associates with 28S, 18S and 5.8S mature rRNAs, several rRNA precursors and probably U3 small nucleolar RNA. May be involved in regulation of intermediate and late steps of rRNA processing. May be involved i [...] (394 aa)
**Q9BX40**	LS14B	Protein LSM14 homolog B OS=Homo sapiens OX=9606 GN=LSM14B PE=1 SV=1	May play a role in control of mRNA translation
**Q9NTM9**	CUTC	Copper homeostasis protein cutC homolog OS=Homo sapiens OX=9606 GN=CUTC PE=1 SV=1	May play a role in copper homeostasis. Can bind one Cu(1+) per subunit (273 aa)
**Q0VAQ4**	SMAGP	Small cell adhesion glycoprotein OS=Homo sapiens OX=9606 GN=SMAGP PE=1 SV=1	May play a role in epithelial cell-cell contacts. May play a role in tumor invasiveness and metastasis formation (97 aa)
**Q7Z2T5**	TRM1L	TRMT1-like protein OS=Homo sapiens OX=9606 GN=TRMT1L PE=1 SV=2	May play a role in motor coordination and exploratory behavior
**Q9ULX3**	NOB1	RNA-binding protein NOB1 OS=Homo sapiens OX=9606 GN=NOB1 PE=1 SV=1	May play a role in mRNA degradation
**Q9ULR0**	ISY1	Pre-mRNA-splicing factor ISY1 homolog OS=Homo sapiens OX=9606 GN=ISY1 PE=1 SV=3	May play a role in pre-mRNA splicing

**Q16625**	OCLN	Occludin OS=Homo sapiens OX=9606 GN=OCLN PE=1 SV=1	May play a role in the formation and regulation of the tight junction (TJ) paracellular permeability barrier. It is able to induce adhesion when expressed in cells lacking tight junctions
**Q8NC51**	PAIRB	Plasminogen activator inhibitor 1 RNA-binding protein OS=Homo sapiens OX=9606 GN=SERBP1 PE=1 SV=2	May play a role in the regulation of mRNA stability. Binds to the 3'-most 134 nt of the SERPINE1/PAI1 mRNA, a region which confers cyclic nucleotide regulation of message decay (408 aa)
**P48634**	PRC2A	Protein PRRC2A OS=Homo sapiens OX=9606 GN=PRRC2A PE=1 SV=3	May play a role in the regulation of pre-mRNA splicing (2157 aa)
**Q9Y4K4**	M4K5	Mitogen-activated protein kinase kinase kinase kinase 5 OS=Homo sapiens OX=9606 GN=MAP4K5 PE=1 SV=2	May play a role in the response to environmental stress. Appears to act upstream of the JUN N-terminal pathway
**Q92997**	DVL3	Segment polarity protein dishevelled homolog DVL-3 OS=Homo sapiens OX=9606 GN=DVL3 PE=1 SV=2	May play a role in the signal transduction pathway mediated by multiple Wnt genes
**P43243**	MATR3	Matrin-3 OS=Homo sapiens OX=9606 GN=MATR3 PE=1 SV=2	May play a role in transcription or may interact with other nuclear matrix proteins to form the internal fibrogranular network. In association with the SFPQ-NONO heteromer may play a role in nuclear retention of defective RNAs. Plays a role in the regulation of DNA virus-mediated innate immune response by assembling into the HDP-RNP complex, a complex that serves as a platform for IRF3 phosphorylation and subsequent innate immune response activation through the cGAS-STING pathway. May bind to specific miRNA hairpins (847 aa)
**Q13610**	PWP1	Periodic tryptophan protein 1 homolog OS=Homo sapiens OX=9606 GN=PWP1 PE=1 SV=1	May play an important role in cell growth and/or transcription
**P62753**	RS6	40S ribosomal protein S6 OS=Homo sapiens OX=9606 GN=RPS6 PE=1 SV=1	May play an important role in controlling cell growth and proliferation through the selective translation of particular classes of mRNA
**P04264**	K2C1	Keratin, type II cytoskeletal 1 OS=Homo sapiens OX=9606 GN=KRT1 PE=1 SV=6	May regulate the activity of kinases such as PKC and SRC via binding to integrin beta-1 (ITB1) and the receptor of activated protein C kinase 1 (RACK1). In complex with C1QBP is a high affinity receptor for kininogen-1/HMWK
**P35527**	K1C9	Keratin, type I cytoskeletal 9 OS=Homo sapiens OX=9606 GN=KRT9 PE=1 SV=3	May serve an important special function either in the mature palmar and plantar skin tissue or in the morphogenetic program of the formation of these tissues. Plays a role in keratin filament assembly

**P67809**	YBOX1	Nuclease-sensitive element-binding protein 1 OS=Homo sapiens OX=9606 GN=YBX1 PE=1 SV=3	Mediates pre-mRNA alternative splicing regulation. Binds to splice sites in pre-mRNA and regulates splice site selection. Binds and stabilizes cytoplasmic mRNA. Contributes to the regulation of translation by modulating the interaction between the mRNA and eukaryotic initiation factors (By similarity). Regulates the transcription of numerous genes. Its transcriptional activity on the multidrug resistance gene MDR1 is enhanced in presence of the APEX1 acetylated form at 'Lys-6' and 'Lys-7'. Binds to promoters that contain a Y-box (5'-CTGATTG [...] (324 aa)
**Q9C0E2**	XPO4	Exportin-4 OS=Homo sapiens OX=9606 GN=XPO4 PE=1 SV=2	Mediates the nuclear export of proteins (cargos) with broad substrate specificity. In the nucleus binds cooperatively to its cargo and to the GTPase Ran in its active GTP-bound form. Docking of this trimeric complex to the nuclear pore complex (NPC) is mediated through binding to nucleoporins. Upon transit of a nuclear export complex into the cytoplasm, disassembling of the complex and hydrolysis of Ran-GTP to Ran-GDP (induced by RANBP1 and RANGAP1, respectively) cause release of the cargo from the export receptor. XPO4 then return to the nuclear compartment and mediate ano [...] (1151 aa)
**P26358**	DNMT1	DNA (cytosine-5)-methyltransferase 1 OS=Homo sapiens OX=9606 GN=DNMT1 PE=1 SV=2	Methylates CpG residues. Preferentially methylates hemimethylated DNA. Associates with DNA replication sites in S phase maintaining the methylation pattern in the newly synthesized strand, that is essential for epigenetic inheritance. Associates with chromatin during G2 and M phases to maintain DNA methylation independently of replication. It is responsible for maintaining methylation patterns established in development. DNA methylation is coordinated with methylation of histones. Mediates transcriptional repression by direct binding to HDAC2. In a [...] (1632 aa)

**Q14980**	NUMA1	Nuclear mitotic apparatus protein 1 OS=Homo sapiens OX=9606 GN=NUMA1 PE=1 SV=2	Microtubule (MT)-binding protein that plays a role in the formation and maintenance of the spindle poles and the alignement and the segregation of chromosomes during mitotic cell division. Functions to tether the minus ends of MTs at the spindle poles, which is critical for the establishment and maintenance of the spindle poles. Plays a role in the establishment of the mitotic spindle orientation during metaphase and elongation during anaphase in a dynein-dynactin-dependent manner. In metaphase, part of a ternary complex composed of GPSM2 and G(i) a [...] (2115 aa)
**Q96BK5**	PINX1	PIN2/TERF1-interacting telomerase inhibitor 1 OS=Homo sapiens OX=9606 GN=PINX1 PE=1 SV=2	Microtubule-binding protein essential for faithful chromosome segregation. Mediates TRF1 and TERT accumulation in nucleolus and enhances TRF1 binding to telomeres. Inhibits telomerase activity. May inhibit cell proliferation and act as tumor suppressor
**Q01844**	EWS	RNA-binding protein EWS OS=Homo sapiens OX=9606 GN=EWSR1 PE=1 SV=1	Might normally function as a transcriptional repressor. EWS-fusion-proteins (EFPS) may play a role in the tumorigenic process. They may disturb gene expression by mimicking, or interfering with the normal function of CTD-POLII within the transcription initiation complex. They may also contribute to an aberrant activation of the fusion protein target genes
**O60220**	TIM8A	Mitochondrial import inner membrane translocase subunit Tim8 A OS=Homo sapiens OX=9606 GN=TIMM8A PE=1 SV=1	Mitochondrial intermembrane chaperone that participates in the import and insertion of some multi-pass transmembrane proteins into the mitochondrial inner membrane. Also required for the transfer of beta-barrel precursors from the TOM complex to the sorting and assembly machinery (SAM complex) of the outer membrane. Acts as a chaperone-like protein that protects the hydrophobic precursors from aggregation and guide them through the mitochondrial intermembrane space. The TIMM8-TIMM13 complex mediates the import of proteins [...] (97 aa)

**P11142**	HSP7C	Heat shock cognate 71 kDa protein OS=Homo sapiens OX=9606 GN=HSPA8 PE=1 SV=1	Molecular chaperone implicated in a wide variety of cellular processes, including protection of the proteome from stress, folding and transport of newly synthesized polypeptides, activation of proteolysis of misfolded proteins and the formation and dissociation of protein complexes. Plays a pivotal role in the protein quality control system, ensuring the correct folding of proteins, the re-folding of misfolded proteins and controlling the targeting of proteins for subsequent degradation. This is achieved through cycles of ATP binding, ATP hydrolysis a [...] (646 aa)
**P14625**	ENPL	Endoplasmin OS=Homo sapiens OX=9606 GN=HSP90B1 PE=1 SV=1	Molecular chaperone that functions in the processing and transport of secreted proteins. When associated with CNPY3, required for proper folding of Toll-like receptors (By similarity). Functions in endoplasmic reticulum associated degradation (ERAD). Has ATPase activity
**P08238**	HS90B	Heat shock protein HSP 90-beta OS=Homo sapiens OX=9606 GN=HSP90AB1 PE=1 SV=4	Molecular chaperone that promotes the maturation, structural maintenance and proper regulation of specific target proteins involved for instance in cell cycle control and signal transduction. Undergoes a functional cycle that is linked to its ATPase activity. This cycle probably induces conformational changes in the client proteins, thereby causing their activation. Interacts dynamically with various co-chaperones that modulate its substrate recognition, ATPase cycle and chaperone function. Engages with a range of client protein classes via its interacti [...] (724 aa)
**P50990**	TCPQ	T-complex protein 1 subunit theta OS=Homo sapiens OX=9606 GN=CCT8 PE=1 SV=4	Component of the chaperonin-containing T-complex (TRiC), a molecular chaperone complex that assists the folding of proteins upon ATP hydrolysis (PubMed:25467444). The TRiC complex mediates the folding of WRAP53/TCAB1, thereby regulating telomere maintenance (PubMed:25467444). As part of the TRiC complex may play a role in the assembly of BBSome, a complex involved in ciliogenesis regulating transports vesicles to the cilia (PubMed:20080638). The TRiC complex plays a role in the folding of actin and tubulin

**O15371**	EIF3D	Eukaryotic translation initiation factor 3 subunit D OS=Homo sapiens OX=9606 GN=EIF3D PE=1 SV=1	mRNA cap-binding component of the eukaryotic translation initiation factor 3 (eIF-3) complex, a complex required for several steps in the initiation of protein synthesis of a specialized repertoire of mRNAs. The eIF-3 complex associates with the 40S ribosome and facilitates the recruitment of eIF-1, eIF-1A, eIF-2-GTP-methionyl-tRNAi and eIF-5 to form the 43S pre-initiation complex (43S PIC). The eIF-3 complex stimulates mRNA recruitment to the 43S PIC and scanning of the mRNA for AUG recognition. The eIF-3 complex is also required f [...] (548 aa)
**P63241**	IF5A1	Eukaryotic translation initiation factor 5A-1 OS=Homo sapiens OX=9606 GN=EIF5A PE=1 SV=2	mRNA-binding protein involved in translation elongation. Has an important function at the level of mRNA turnover, probably acting downstream of decapping. Involved in actin dynamics and cell cycle progression, mRNA decay and probably in a pathway involved in stress response and maintenance of cell wall integrity. With syntenin SDCBP, functions as a regulator of p53/TP53 and p53/TP53-dependent apoptosis. Regulates also TNF- alpha-mediated apoptosis. Mediates effects of polyamines on neuronal process extension and survival. May play an impor [...] (184 aa)
**Q6IS14**	IF5AL	Eukaryotic translation initiation factor 5A-1-like OS=Homo sapiens OX=9606 GN=EIF5AL1 PE=2 SV=2	mRNA-binding protein involved in translation elongation. Has an important function at the level of mRNA turnover, probably acting downstream of decapping. Involved in actin dynamics and cell cycle progression, mRNA decay and probably in a pathway involved in stress response and maintenance of cell wall integrity. Functions as a regulator of apoptosis. Mediates effects of polyamines on neuronal process extension and survival. May play an important role in brain development and function, and in skeletal muscle stem cell differentiation [...] (154 aa)

**Q96T88**	UHRF1	E3 ubiquitin-protein ligase UHRF1 OS=Homo sapiens OX=9606 GN=UHRF1 PE=1 SV=1	Multidomain protein that acts as a key epigenetic regulator by bridging DNA methylation and chromatin modification. Specifically recognizes and binds hemimethylated DNA at replication forks via its YDG domain and recruits DNMT1 methyltransferase to ensure faithful propagation of the DNA methylation patterns through DNA replication. In addition to its role in maintenance of DNA methylation, also plays a key role in chromatin modification- through its tudor-like regions and PHD- type zinc fingers, specifically recognizes and binds histone H3 trimethylat [...] (806 aa)
**Q08211**	DHX9	ATP-dependent RNA helicase A OS=Homo sapiens OX=9606 GN=DHX9 PE=1 SV=4	Multifunctional ATP-dependent nucleic acid helicase that unwinds DNA and RNA in a 3’ to 5’ direction and that plays important roles in many processes, such as DNA replication, transcriptional activation, post-transcriptional RNA regulation, mRNA translation and RNA-mediated gene silencing. Requires a 3’-single-stranded tail as entry site for acid nuclei unwinding activities as well as the binding and hydrolyzing of any of the four ribo- or deoxyribo- nucleotide triphosphates (NTPs). Unwinds numerous nucleic acid substrates such as double-stranded (ds) DNA [...] (1270 aa)
**P06733**	ENOA	Alpha-enolase OS=Homo sapiens OX=9606 GN=ENO1 PE=1 SV=2	Multifunctional enzyme that, as well as its role in glycolysis, plays a part in various processes such as growth control, hypoxia tolerance and allergic responses. May also function in the intravascular and pericellular fibrinolytic system due to its ability to serve as a receptor and activator of plasminogen on the cell surface of several cell-types such as leukocytes and neurons. Stimulates immunoglobulin production
**Q06787**	FMR1	Synaptic functional regulator FMR1 OS=Homo sapiens OX=9606 GN=FMR1 PE=1 SV=1	Multifunctional polyribosome-associated RNA-binding protein that plays a central role in neuronal development and synaptic plasticity through the regulation of alternative mRNA splicing, mRNA stability, mRNA dendritic transport and postsynaptic local protein synthesis of a subset of mRNAs. Plays a role in the alternative splicing of its own mRNA. Plays a role in mRNA nuclear export (By similarity). Together with export factor NXF2, is involved in the regulation of the NXF1 mRNA stability in neurons (By similarity). Stabilizes the scaffolding postsyna [...] (632 aa)

**P27695**	APEX1	DNA-(apurinic or apyrimidinic site) lyase OS=Homo sapiens OX=9606 GN=APEX1 PE=1 SV=2	Multifunctional protein that plays a central role in the cellular response to oxidative stress. The two major activities of APEX1 in DNA repair and redox regulation of transcriptional factors. Functions as a apurinic/apyrimidinic (AP) endodeoxyribonuclease in the DNA base excision repair (BER) pathway of DNA lesions induced by oxidative and alkylating agents. Initiates repair of AP sites in DNA by catalyzing hydrolytic incision of the phosphodiester backbone immediately adjacent to the damage, generating a single-strand break with 5’-deoxyribo [...] (318 aa)
**O15347**	HMGB3	High mobility group protein B3 OS=Homo sapiens OX=9606 GN=HMGB3 PE=1 SV=4	Multifunctional protein with various roles in different cellular compartments. May act in a redox sensitive manner. Associates with chromatin and binds DNA with a preference to non- canonical DNA structures such as single-stranded DNA. Can bent DNA and enhance DNA flexibility by looping thus providing a mechanism to promote activities on various gene promoters (By similarity). Proposed to be involved in the innate immune response to nucleic acids by acting as a cytoplasmic promiscuous immunogenic DNA/RNA sensor (By similarity). Negatively regulates B-cel [...] (200 aa)
**P26583**	HMGB2	High mobility group protein B2 OS=Homo sapiens OX=9606 GN=HMGB2 PE=1 SV=2	Multifunctional protein with various roles in different cellular compartments. May act in a redox sensitive manner. In the nucleus is an abundant chromatin-associated non-histone protein involved in transcription, chromatin remodeling and V(D)J recombination and probably other processes. Binds DNA with a preference to non-canonical DNA structures such as single-stranded DNA. Can bent DNA and enhance DNA flexibility by looping thus providing a mechanism to promote activities on various gene promoters by enhancing transcription factor binding and/or bringi [...] (209 aa)

**P09429**	HMGB1	High mobility group protein B1 OS=Homo sapiens OX=9606 GN=HMGB1 PE=1 SV=3	Multifunctional redox sensitive protein with various roles in different cellular compartments. In the nucleus is one of the major chromatin-associated non-histone proteins and acts as a DNA chaperone involved in replication, transcription, chromatin remodeling, V(D)J recombination, DNA repair and genome stability. Proposed to be an universal biosensor for nucleic acids. Promotes host inflammatory response to sterile and infectious signals and is involved in the coordination and integration of innate and adaptive immune responses. In the cytoplasm functio [...] (215 aa)
**P25490**	TYY1	Transcriptional repressor protein YY1 OS=Homo sapiens OX=9606 GN=YY1 PE=1 SV=2	Multifunctional transcription factor that exhibits positive and negative control on a large number of cellular and viral genes by binding to sites overlapping the transcription start site. Binds to the consensus sequence 5’-CCGCCATNTT-3’; some genes have been shown to contain a longer binding motif allowing enhanced binding; the initial CG dinucleotide can be methylated greatly reducing the binding affinity. The effect on transcription regulation is depending upon the context in which it binds and diverse mechanisms of action include direct activa [...] (414 aa)
**Q01105**	SET	Protein SET OS=Homo sapiens OX=9606 GN=SET PE=1 SV=3	Multitasking protein, involved in apoptosis, transcription, nucleosome assembly and histone chaperoning. Isoform 2 anti-apoptotic activity is mediated by inhibition of the GZMA-activated DNase, NME1. In the course of cytotoxic T- lymphocyte (CTL)-induced apoptosis, GZMA cleaves SET, disrupting its binding to NME1 and releasing NME1 inhibition. Isoform 1 and isoform 2 are potent inhibitors of protein phosphatase 2A. Isoform 1 and isoform 2 inhibit EP300/CREBBP and PCAF-mediated acetylation of histones (HAT) and nucleosomes, most probably by masking the accessibility of lysi [...] (290 aa)
**P54727**	RD23B	UV excision repair protein RAD23 homolog B OS=Homo sapiens OX=9606 GN=RAD23B PE=1 SV=1	Multiubiquitin chain receptor involved in modulation of proteasomal degradation. Binds to polyubiquitin chains. Proposed to be capable to bind simultaneously to the 26S proteasome and to polyubiquitinated substrates and to deliver ubiquitinated proteins to the proteasome. May play a role in endoplasmic reticulum- associated degradation (ERAD) of misfolded glycoproteins by association with PNGase and delivering deglycosylated proteins to the proteasome

**P13535**	MYH8	Myosin-8 OS=Homo sapiens OX=9606 GN=MYH8 PE=1 SV=3	Muscle contraction; Myosins are a large family of motor proteins that share the common features of ATP hydrolysis (ATPase enzyme activity), actin binding and potential for kinetic energy transduction. Originally isolated from muscle cells, almost all eukaryotic cells are known to contain myosins.
**P19105**	ML12A	Myosin regulatory light chain 12A OS=Homo sapiens OX=9606 GN=MYL12A PE=1 SV=2	Myosin regulatory subunit that plays an important role in regulation of both smooth muscle and nonmuscle cell contractile activity via its phosphorylation. Implicated in cytokinesis, receptor capping, and cell locomotion (By similarity)
**Q86UY6**	NAA40	N-alpha-acetyltransferase 40 OS=Homo sapiens OX=9606 GN=NAA40 PE=1 SV=1	N-alpha-acetyltransferase that specifically mediates the acetylation of the N-terminal residues of histones H4 and H2A. In contrast to other N-alpha-acetyltransferase, has a very specific selectivity for histones H4 and H2A N-terminus and specifically recognizes the 'Ser-Gly-Arg- Gly sequence'. Acts as a negative regulator of apoptosis. May play a role in hepatic lipid metabolism (By similarity)
**P23193**	TCEA1	Transcription elongation factor A protein 1 OS=Homo sapiens OX=9606 GN=TCEA1 PE=1 SV=2	Necessary for efficient RNA polymerase II transcription elongation past template-encoded arresting sites. The arresting sites in DNA have the property of trapping a certain fraction of elongating RNA polymerases that pass through, resulting in locked ternary complexes. Cleavage of the nascent transcript by S-II allows the resumption of elongation from the new 3'-terminus
**P41567**	EIF1	Eukaryotic translation initiation factor 1 OS=Homo sapiens OX=9606 GN=EIF1 PE=1 SV=1	Necessary for scanning and involved in initiation site selection. Promotes the assembly of 48S ribosomal complexes at the authentic initiation codon of a conventional capped mRNA (113 aa)
**Q15637**	SF01	Splicing factor 1 OS=Homo sapiens OX=9606 GN=SF1 PE=1 SV=4	Necessary for the ATP-dependent first step of spliceosome assembly. Binds to the intron branch point sequence (BPS) 5'-UACUAAC-3' of the pre-mRNA. May act as transcription repressor

**P26368**	U2AF2	Splicing factor U2AF 65 kDa subunit OS=Homo sapiens OX=9606 GN=U2AF2 PE=1 SV=4	Necessary for the splicing of pre-mRNA. By recruiting PRPF19 and the PRP19C/Prp19 complex/NTC/Nineteen complex to the RNA polymerase II C-terminal domain (CTD), and thereby pre-mRNA, may couple transcription to splicing. Induces cardiac troponin-T (TNNT2) pre-mRNA exon inclusion in muscle. Regulates the TNNT2 exon 5 inclusion through competition with MBNL1. Binds preferentially to a single-stranded structure within the polypyrimidine tract of TNNT2 intron 4 during spliceosome assembly. Required for the export of mRNA out of the nucleus, even if the [...] (475 aa)
**Q01130**	SRSF2	Serine/arginine-rich splicing factor 2 OS=Homo sapiens OX=9606 GN=SRSF2 PE=1 SV=4	Necessary for the splicing of pre-mRNA. It is required for formation of the earliest ATP-dependent splicing complex and interacts with spliceosomal components bound to both the 5'- and 3'-splice sites during spliceosome assembly. It also is required for ATP-dependent interactions of both U1 and U2 snRNPs with pre- mRNA. Interacts with other spliceosomal components, via the RS domains, to form a bridge between the 5'- and 3'-splice site binding components, U1 snRNP and U2AF. Binds to purine-rich RNA sequences, either 5'-AGSAGAGTA-3' (S=C or G) or [...] (221 aa)
**Q9UBB4**	ATX10	Ataxin-10 OS=Homo sapiens OX=9606 GN=ATXN10 PE=1 SV=1	Necessary for the survival of cerebellar neurons. Induces neuritogenesis by activating the Ras-MAP kinase pathway. May play a role in the maintenance of a critical intracellular glycosylation level and homeostasis
**Q4G0J3**	LARP7	La-related protein 7 OS=Homo sapiens OX=9606 GN=LARP7 PE=1 SV=1	Negative transcriptional regulator of polymerase II genes, acting by means of the 7SK RNP system. Within the 7SK RNP complex, the positive transcription elongation factor b (P-TEFb) is sequestered in an inactive form, preventing RNA polymerase II phosphorylation and subsequent transcriptional elongation
**Q7Z5L7**	PODN	Podocan OS=Homo sapiens OX=9606 GN=PODN PE=1 SV=2	Negatively regulates cell proliferation and cell migration
**Q5TBB1**	RNH2B	Ribonuclease H2 subunit B OS=Homo sapiens OX=9606 GN=RNASEH2B PE=1 SV=1	Non catalytic subunit of RNase H2, an endonuclease that specifically degrades the RNA of RNA-DNA hybrids. Participates in DNA replication, possibly by mediating the removal of lagging- strand Okazaki fragment RNA primers during DNA replication. Mediates the excision of single ribonucleotides from DNA-RNA duplexes (312 aa)

**Q12904**	AIMP1	Aminoacyl tRNA synthase complex-interacting multifunctional protein 1 OS=Homo sapiens OX=9606 GN=AIMP1 PE=1 SV=2	Non-catalytic component of the multisynthase complex. Stimulates the catalytic activity of cytoplasmic arginyl-tRNA synthase. Binds tRNA. Possesses inflammatory cytokine activity. Negatively regulates TGF-beta signaling through stabilization of SMURF2 by binding to SMURF2 and inhibiting its SMAD7-mediated degradation. Involved in glucose homeostasis through induction of glucagon secretion at low glucose levels. Promotes dermal fibroblast proliferation and wound repair. Regulates KDELR1-mediated retention of HSP90B1 [...] (336 aa)
**Q06265**	EXOS9	Exosome complex component RRP45 OS=Homo sapiens OX=9606 GN=EXOSC9 PE=1 SV=3	Non-catalytic component of the RNA exosome complex which has 3’->5’ exoribonuclease activity and participates in a multitude of cellular RNA processing and degradation events. In the nucleus, the RNA exosome complex is involved in proper maturation of stable RNA species such as rRNA, snRNA and snoRNA, in the elimination of RNA processing by-products and non-coding ’pervasive’ transcripts, such as antisense RNA species and promoter-upstream transcripts (PROMPTs), and of mRNAs with processing defects, thereby limiting or excluding their export to the cyto [...] (456 aa)
**Q5RKV6**	EXOS6	Exosome complex component MTR3 OS=Homo sapiens OX=9606 GN=EXOSC6 PE=1 SV=1	Non-catalytic component of the RNA exosome complex which has 3’->5’ exoribonuclease activity and participates in a multitude of cellular RNA processing and degradation events. In the nucleus, the RNA exosome complex is involved in proper maturation of stable RNA species such as rRNA, snRNA and snoRNA, in the elimination of RNA processing by-products and non-coding ’pervasive’ transcripts, such as antisense RNA species and promoter-upstream transcripts (PROMPTs), and of mRNAs with processing defects, thereby limiting or excluding their export to the cytop [...] (272 aa)
**P27816**	MAP4	Microtubule-associated protein 4 OS=Homo sapiens OX=9606 GN=MAP4 PE=1 SV=3	Non-neuronal microtubule-associated protein. Promotes microtubule assembly (1152 aa)

**Q15046**	SYK	Lysine--tRNA ligase OS=Homo sapiens OX=9606 GN=KARS PE=1 SV=3	Non-receptor tyrosine kinase which mediates signal transduction downstream of a variety of transmembrane receptors including classical immunoreceptors like the B-cell receptor (BCR). Regulates several biological processes including innate and adaptive immunity, cell adhesion, osteoclast maturation, platelet activation and vascular development. Assembles into signaling complexes with activated receptors at the plasma membrane via interaction between its SH2 domains and the receptor tyrosine- phosphorylated ITAM domains. The association with the receptor can [...] (635 aa)
**Q13263**	TIF1B	Transcription intermediary factor 1-beta OS=Homo sapiens OX=9606 GN=TRIM28 PE=1 SV=5	Nuclear corepressor for KRAB domain-containing zinc finger proteins (KRAB-ZFPs). Mediates gene silencing by recruiting CHD3, a subunit of the nucleosome remodeling and deacetylation (NuRD) complex, and SETDB1 (which specifically methylates histone H3 at 'Lys-9' (H3K9me)) to the promoter regions of KRAB target genes. Enhances transcriptional repression by coordinating the increase in H3K9me, the decrease in histone H3 'Lys-9 and 'Lys-14' acetylation (H3K9ac and H3K14ac, respectively) and the disposition of HP1 proteins to silence gene expression [...] (835 aa)
**Q9NW13**	RBM28	RNA-binding protein 28 OS=Homo sapiens OX=9606 GN=RBM28 PE=1 SV=3	Nucleolar component of the spliceosomal ribonucleoprotein complexes
**Q13428**	TCOF	Treacle protein OS=Homo sapiens OX=9606 GN=TCOF1 PE=1 SV=3	Nucleolar protein that acts as a regulator of RNA polymerase I by connecting RNA polymerase I with enzymes responsible for ribosomal processing and modification. Required for neural crest specification-following monoubiquitination by the BCR(KBTBD8) complex, associates with NOLC1 and acts as a platform to connect RNA polymerase I with enzymes responsible for ribosomal processing and modification, leading to remodel the translational program of differentiating cells in favor of neural crest specification (1488 aa)

**P19338**	NUCL	Nucleolin OS=Homo sapiens OX=9606 GN=NCL PE=1 SV=3	Nucleolin is the major nucleolar protein of growing eukaryotic cells. It is found associated with intranucleolar chromatin and pre-ribosomal particles. It induces chromatin decondensation by binding to histone H1. It is thought to play a role in pre-rRNA transcription and ribosome assembly. May play a role in the process of transcriptional elongation. Binds RNA oligonucleotides with 5’-UUAGGG-3’ repeats more tightly than the telomeric single-stranded DNA 5’-TTAGGG-3’ repeats (710 aa)
**Q8NH59**	O51Q1	Olfactory receptor 51Q1 OS=Homo sapiens OX=9606 GN=OR51Q1 PE=2 SV=2	Odorant receptor; Olfactory receptors interact with odorant molecules in the nose, to initiate a neuronal response that triggers the perception of a smell. The olfactory receptor proteins are members of a large family of G-protein-coupled receptors (GPCR) arising from single coding-exon genes. Olfactory receptors share a 7-transmembrane domain structure with many neurotransmitter and hormone receptors and are responsible for the recognition and G protein-mediated transduction of odorant signals. The olfactory receptor gene family is the largest in the genome. The nomenclature assigned to the olfactory receptor genes and proteins for this organism is independent of other organisms
**P61978**	HNRPK	Heterogeneous nuclear ribonucleoprotein K OS=Homo sapiens OX=9606 GN=HNRNPK PE=1 SV=1	One of the major pre-mRNA-binding proteins. Binds tenaciously to poly(C) sequences. Likely to play a role in the nuclear metabolism of hnRNAs, particularly for pre-mRNAs that contain cytidine-rich sequences. Can also bind poly(C) single- stranded DNA. Plays an important role in p53/TP53 response to DNA damage, acting at the level of both transcription activation and repression. When sumoylated, acts as a transcriptional coactivator of p53/TP53, playing a role in p21/CDKN1A and 14-3-3 sigma/SFN induction (By similarity). As far as transcription [...] (464 aa)
**Q9ULD0**	OGDHL	2-oxoglutarate dehydrogenase-like, mitochondrial OS=Homo sapiens OX=9606 GN=OGDHL PE=1 SV=3	The protein encoded by this gene is similar to oxoglutarate dehydrogenase (OGDH) of the OGDH complex, which degrades glucose and glutamate. This gene encodes several isoforms, including some that appear to localize to mitochondria. The encoded protein down-regulates the AKT signaling cascade and can suppress the growth of cervical cancer cells.

**Q15287**	RNPS1	RNA-binding protein with serine-rich domain 1 OS=Homo sapiens OX=9606 GN=RNPS1 PE=1 SV=1	Part of pre- and post-splicing multiprotein mRNP complexes. Auxiliary component of the splicing-dependent multiprotein exon junction complex (EJC) deposited at splice junction on mRNAs. The EJC is a dynamic structure consisting of core proteins and several peripheral nuclear and cytoplasmic associated factors that join the complex only transiently either during EJC assembly or during subsequent mRNA metabolism. Component of the ASAP and PSAP complexes which bind RNA in a sequence-independent manner and are proposed to be recruited to the E [...] (305 aa)
**Q8IYB3**	SRRM1	Serine/arginine repetitive matrix protein 1 OS=Homo sapiens OX=9606 GN=SRRM1 PE=1 SV=2	Part of pre- and post-splicing multiprotein mRNP complexes. Involved in numerous pre-mRNA processing events. Promotes constitutive and exonic splicing enhancer (ESE)-dependent splicing activation by bridging together sequence-specific (SR family proteins, SFRS4, SFRS5 and TRA2B/SFRS10) and basal snRNP (SNRP70 and SNRPA1) factors of the spliceosome. Stimulates mRNA 3’-end cleavage independently of the formation of an exon junction complex. Binds both pre-mRNA and spliced mRNA 20-25 nt upstream of exon-exon junctions. Binds RNA and DNA with lo [...] (904 aa)
**O14617**	AP3D1	AP-3 complex subunit delta-1 OS=Homo sapiens OX=9606 GN=AP3D1 PE=1 SV=1	Part of the AP-3 complex, an adaptor-related complex which is not clathrin-associated. The complex is associated with the Golgi region as well as more peripheral structures. It facilitates the budding of vesicles from the Golgi membrane and may be directly involved in trafficking to lysosomes. Involved in process of CD8+ T-cell and NK cell degranulation. In concert with the BLOC-1 complex, AP-3 is required to target cargos into vesicles assembled at cell bodies for delivery into neurites and nerve terminals (By similarity)
**O43172**	PRP4	U4/U6 small nuclear ribonucleoprotein Prp4 OS=Homo sapiens OX=9606 GN=PRPF4 PE=1 SV=2	Participates in pre-mRNA splicing. Part of the U4/U5/U6 tri-snRNP complex, one of the building blocks of the spliceosome
**O43395**	PRPF3	U4/U6 small nuclear ribonucleoprotein Prp3 OS=Homo sapiens OX=9606 GN=PRPF3 PE=1 SV=2	Participates in pre-mRNA splicing. Part of the U4/U5/U6 tri-snRNP complex, one of the building blocks of the spliceosome
**Q96LT9**	RNPC3	RNA-binding region-containing protein 3 OS=Homo sapiens OX=9606 GN=RNPC3 PE=1 SV=1	Participates in pre-mRNA U12-dependent splicing, performed by the minor spliceosome which removes U12-type introns. U12-type introns comprises less than 1% of all non-coding sequences. Binds to the 3’-stem-loop of m(7)G-capped U12 snRNA

**O15381**	NVL	Nuclear valosin-containing protein-like OS=Homo sapiens OX=9606 GN=NVL PE=1 SV=1	Participates in the assembly of the telomerase holoenzyme and effecting of telomerase activity via its interaction with TERT. May play a role in 60S ribosomal subunit biogenesis
**P10599**	THIO	Thioredoxin OS=Homo sapiens OX=9606 GN=TXN PE=1 SV=3	Participates in various redox reactions through the reversible oxidation of its active center dithiol to a disulfide and catalyzes dithiol-disulfide exchange reactions. Plays a role in the reversible S-nitrosylation of cysteine residues in target proteins, and thereby contributes to the response to intracellular nitric oxide. Nitrosylates the active site Cys of CASP3 in response to nitric oxide (NO), and thereby inhibits caspase-3 activity. Induces the FOS/JUN AP-1 DNA-binding activity in ionizing radiation (IR) cells through its oxidation/reduction status and stimulates A [...] (105 aa)
**P67870**	CSK2B	Casein kinase II subunit beta OS=Homo sapiens OX=9606 GN=CSNK2B PE=1 SV=1	Participates in Wnt signaling (By similarity). Plays a complex role in regulating the basal catalytic activity of the alpha subunit
**Q9UMZ3**	PTPRQ	Phosphatidylinositol phosphatase PTPRQ OS=Homo sapiens OX=9606 GN=PTPRQ PE=1 SV=2	Phosphatidylinositol phosphatase required for auditory function. May act by regulating the level of phosphatidylinositol 4,5-bisphosphate (PIP2) level in the basal region of hair bundles. Can dephosphorylate a broad range of phosphatidylinositol phosphates, including phosphatidylinositol 3,4,5-trisphosphate and most phosphatidylinositol monophosphates and diphosphates. Phosphate can be hydrolyzed from the D3 and D5 positions in the inositol ring. Has low tyrosine-protein phosphatase activity
**Q9UBS0**	KS6B2	Ribosomal protein S6 kinase beta-2 OS=Homo sapiens OX=9606 GN=RPS6KB2 PE=1 SV=2	Phosphorylates specifically ribosomal protein S6 (482 aa)
**Q9NZH5**	PTTG2	Securin-2 OS=Homo sapiens OX=9606 GN=PTTG2 PE=2 SV=2	PTTG2 (Pituitary Tumor-Transforming 2) is a Protein Coding gene. Among its related pathways are Oocyte meiosis and Human T-cell leukemia virus 1 infection. Gene Ontology (GO) annotations related to this gene include SH3 domain binding. An important paralog of this gene is PTTG1.

**P00747**	PLMN	Plasminogen OS=Homo sapiens OX=9606 GN=PLG PE=1 SV=2	Plasmin dissolves the fibrin of blood clots and acts as a proteolytic factor in a variety of other processes including embryonic development, tissue remodeling, tumor invasion, and inflammation. In ovulation, weakens the walls of the Graafian follicle. It activates the urokinase-type plasminogen activator, collagenases and several complement zymogens, such as C1 and C5. Cleavage of fibronectin and laminin leads to cell detachment and apoptosis. Also cleaves fibrin, thrombospondin and von Willebrand factor. Its role in tissue remodeling and tumor invasion may be modulated b [...] (810 aa)
**P56182**	RRP1	Ribosomal RNA processing protein 1 homolog A OS=Homo sapiens OX=9606 GN=RRP1 PE=1 SV=1	Plays a critical role in the generation of 28S rRNA
**Q96T60**	PNKP	Bifunctional polynucleotide phosphatase/kinase OS=Homo sapiens OX=9606 GN=PNKP PE=1 SV=1	Plays a key role in the repair of DNA damage, functioning as part of both the non-homologous end-joining (NHEJ) and base excision repair (BER) pathways. Through its two catalytic activities, PNK ensures that DNA termini are compatible with extension and ligation by either removing 3’-phosphates from, or by phosphorylating 5’-hydroxyl groups on, the ribose sugar of the DNA backbone
**Q13242**	SRSF9	Serine/arginine-rich splicing factor 9 OS=Homo sapiens OX=9606 GN=SRSF9 PE=1 SV=1	Plays a role in constitutive splicing and can modulate the selection of alternative splice sites. Represses the splicing of MAPT/Tau exon 10
**P51991**	ROA3	Heterogeneous nuclear ribonucleoprotein A3 OS=Homo sapiens OX=9606 GN=HNRNPA3 PE=1 SV=2	Plays a role in cytoplasmic trafficking of RNA. Binds to the cis-acting response element, A2RE. May be involved in pre-mRNA splicing
**P11021**	BIP	Endoplasmic reticulum chaperone BiP OS=Homo sapiens OX=9606 GN=HSPA5 PE=1 SV=2	Plays a role in facilitating the assembly of multimeric protein complexes inside the endoplasmic reticulum. Involved in the correct folding of proteins and degradation of misfolded proteins via its interaction with DNAJC10, probably to facilitate the release of DNAJC10 from its substrate (By similarity)
**Q16555**	DPYL2	Dihydropyrimidinase-related protein 2 OS=Homo sapiens OX=9606 GN=DPYSL2 PE=1 SV=1	Plays a role in neuronal development and polarity, as well as in axon growth and guidance, neuronal growth cone collapse and cell migration. Necessary for signaling by class 3 semaphorins and subsequent remodeling of the cytoskeleton. May play a role in endocytosis

**P26599**	PTBP1	Polypyrimidine tract-binding protein 1 OS=Homo sapiens OX=9606 GN=PTBP1 PE=1 SV=1	Plays a role in pre-mRNA splicing and in the regulation of alternative splicing events. Activates exon skipping of its own pre-mRNA during muscle cell differentiation. Binds to the polypyrimidine tract of introns. May promote RNA looping when bound to two separate polypyrimidine tracts in the same pre-mRNA. May promote the binding of U2 snRNP to pre-mRNA. Cooperates with RAVER1 to modulate switching between mutually exclusive exons during maturation of the TPM1 pre-mRNA. Represses the splicing of MAPT/Tau exon 10. In case of infection by picornav [...] (557 aa)
**Q53GS9**	SNUT2	U4/U6.U5 tri-snRNP-associated protein 2 OS=Homo sapiens OX=9606 GN=USP39 PE=1 SV=2	Plays a role in pre-mRNA splicing as a component of the U4/U6-U5 tri-snRNP, one of the building blocks of the spliceosome. Regulates AURKB mRNA levels, and thereby plays a role in cytokinesis and in the spindle checkpoint. Does not have ubiquitin-specific peptidase activity, but could be a competitor of ubiquitin C-terminal hydrolases (UCHs) (565 aa)
**Q07955**	SRSF1	Serine/arginine-rich splicing factor 1 OS=Homo sapiens OX=9606 GN=SRSF1 PE=1 SV=2	Plays a role in preventing exon skipping, ensuring the accuracy of splicing and regulating alternative splicing. Interacts with other spliceosomal components, via the RS domains, to form a bridge between the 5’- and 3’-splice site binding components, U1 snRNP and U2AF. Can stimulate binding of U1 snRNP to a 5’-splice site-containing pre-mRNA. Binds to purine-rich RNA sequences, either the octamer, 5’-RGAAGAAC-3’ (r=A or G) or the decamers, AGGACAGAGC/AGGACGAAGC. Binds preferentially to the 5’- CGAGGCG-3’ motif in vitro. Three copies of the octame [...] (248 aa)
**Q9H8Y5**	ANKZ1	Ankyrin repeat and zinc finger domain-containing protein 1 OS=Homo sapiens OX=9606 GN=ANKZF1 PE=1 SV=1	Plays a role in the cellular response to hydrogen peroxide and in the maintenance of mitochondrial integrity under conditions of cellular stress. Involved in the endoplasmic reticulum (ER)-associated degradation (ERAD) pathway (By similarity)
**Q15785**	TOM34	Mitochondrial import receptor subunit TOM34 OS=Homo sapiens OX=9606 GN=TOMM34 PE=1 SV=2	Plays a role in the import of cytosolically synthesized preproteins into mitochondria. Binds the mature portion of precursor proteins. Interacts with cellular components, and possesses weak ATPase activity. May be a chaperone-like protein that helps to keep newly synthesized precursors in an unfolded import compatible state

**Q8NHQ1**	CEP70	Centrosomal protein of 70 kDa OS=Homo sapiens OX=9606 GN=CEP70 PE=1 SV=2	Plays a role in the organization of both preexisting and nascent microtubules in interphase cells. During mitosis, required for the organization and orientation of the mitotic spindle (597 aa)
**Q14202**	ZMYM3	Zinc finger MYM-type protein 3 OS=Homo sapiens OX=9606 GN=ZMYM3 PE=1 SV=2	Plays a role in the regulation of cell morphology and cytoskeletal organization
**O60841**	IF2P	Eukaryotic translation initiation factor 5B OS=Homo sapiens OX=9606 GN=EIF5B PE=1 SV=4	Plays a role in translation initiation. Translational GTPase that catalyzes the joining of the 40S and 60S subunits to form the 80S initiation complex with the initiator methionine-tRNA in the P-site base paired to the start codon. GTP binding and hydrolysis induces conformational changes in the enzyme that renders it active for productive interactions with the ribosome. The release of the enzyme after formation of the initiation complex is a prerequisite to form elongation-competent ribosomes
**Q9Y4Y9**	LSM5	U6 snRNA-associated Sm-like protein LSm5 OS=Homo sapiens OX=9606 GN=LSM5 PE=1 SV=3	Plays a role in U6 snRNP assembly and function. Binds to the 3' end of U6 snRNA, thereby facilitating formation of the spliceosomal U4/U6 duplex formation in vitro
**P09884**	DPOLA	DNA polymerase alpha catalytic subunit OS=Homo sapiens OX=9606 GN=POLA1 PE=1 SV=2	Plays an essential role in the initiation of DNA replication. During the S phase of the cell cycle, the DNA polymerase alpha complex (composed of a catalytic subunit POLA1/p180, a regulatory subunit POLA2/p70 and two primase subunits PRIM1/p49 and PRIM2/p58) is recruited to DNA at the replicative forks via direct interactions with MCM10 and WDHD1. The primase subunit of the polymerase alpha complex initiates DNA synthesis by oligomerising short RNA primers on both leading and lagging strands. These primers are initially extended by the polymerase [...] (1462 aa)
**Q14157**	UBP2L	Ubiquitin-associated protein 2-like OS=Homo sapiens OX=9606 GN=UBAP2L PE=1 SV=2	Plays an important role in the activity of long-term repopulating hematopoietic stem cells (LT-HSCs) (1087 aa)
**Q7L2E3**	DHX30	ATP-dependent RNA helicase DHX30 OS=Homo sapiens OX=9606 GN=DHX30 PE=1 SV=1	Plays an important role in the assembly of the mitochondrial large ribosomal subunit. Required for optimal function of the zinc-finger antiviral protein ZC3HAV1 (By similarity). Associates with mitochondrial DNA
**Q9UHL4**	DPP2	Dipeptidyl peptidase 2 OS=Homo sapiens OX=9606 GN=DPP7 PE=1 SV=3	Plays an important role in the degradation of some oligopeptides
**P05386**	RLA1	60S acidic ribosomal protein P1 OS=Homo sapiens OX=9606 GN=RPLP1 PE=1 SV=1	Plays an important role in the elongation step of protein synthesis
**P05387**	RLA2	60S acidic ribosomal protein P2 OS=Homo sapiens OX=9606 GN=RPLP2 PE=1 SV=1	Plays an important role in the elongation step of protein synthesis

**O75531**	BAF	Barrier-to-autointegration factor OS=Homo sapiens OX=9606 GN=BANF1 PE=1 SV=1	Plays fundamental roles in nuclear assembly, chromatin organization, gene expression and gonad development. May potently compress chromatin structure and be involved in membrane recruitment and chromatin decondensation during nuclear assembly. Contains 2 non-specific dsDNA-binding sites which may promote DNA cross-bridging
**O00139**	KIF2A	Kinesin-like protein KIF2A OS=Homo sapiens OX=9606 GN=KIF2A PE=1 SV=3	Plus end-directed microtubule-dependent motor required for normal brain development. May regulate microtubule dynamics during axonal growth. Required for normal progression through mitosis. Required for normal congress of chromosomes at the metaphase plate. Required for normal spindle dynamics during mitosis. Promotes spindle turnover. Implicated in formation of bipolar mitotic spindles. Has microtubule depolymerization activity
**Q15691**	MARE1	Microtubule-associated protein RP/EB family member 1 OS=Homo sapiens OX=9606 GN=MAPRE1 PE=1 SV=3	Plus-end tracking protein (+TIP) that binds to the plus-end of microtubules and regulates the dynamics of the microtubule cytoskeleton. Promotes cytoplasmic microtubule nucleation and elongation. May be involved in spindle function by stabilizing microtubules and anchoring them at centrosomes. Also acts as a regulator of minus-end microtubule organization- interacts with the complex formed by AKAP9 and PDE4DIP, leading to recruit CAMSAP2 to the Golgi apparatus, thereby tethering non-centrosomal minus-end microtubules to the Golgi, [...] (268 aa)
**Q4VXU2**	PAP1L	Polyadenylate-binding protein 1-like OS=Homo sapiens OX=9606 GN=PABPC1L PE=2 SV=1	poly(A) binding protein cytoplasmic 1 like
**Q15910**	EZH2	Histone-lysine N-methyltransferase EZH2 OS=Homo sapiens OX=9606 GN=EZH2 PE=1 SV=2	Polycomb group (PcG) protein. Catalytic subunit of the PRC2/EED-EZH2 complex, which methylates ’Lys-9’ (H3K9me) and ’Lys- 27’ (H3K27me) of histone H3, leading to transcriptional repression of the affected target gene. Able to mono-, di- and trimethylate ’Lys-27’ of histone H3 to form H3K27me1, H3K27me2 and H3K27me3, respectively. Displays a preference for substrates with less methylation, loses activity when progressively more methyl groups are incorporated into H3K27, H3K27me0 > H3K27me1 > H3K27me2. Compared to EZH1-containing complexes, it is [...] (751 aa)

**Q15022**	SUZ12	Polycomb protein SUZ12 OS=Homo sapiens OX=9606 GN=SUZ12 PE=1 SV=3	Polycomb group (PcG) protein. Component of the PRC2/EED- EZH2 complex, which methylates 'Lys-9' (H3K9me) and 'Lys-27' (H3K27me) of histone H3, leading to transcriptional repression of the affected target gene. The PRC2/EED-EZH2 complex may also serve as a recruiting platform for DNA methyltransferases, thereby linking two epigenetic repression systems. Genes repressed by the PRC2/EED-EZH2 complex include HOXC8, HOXA9, MYT1 and CDKN2A (739 aa)
**Q14684**	RRP1B	Ribosomal RNA processing protein 1 homolog B OS=Homo sapiens OX=9606 GN=RRP1B PE=1 SV=3	Positively regulates DNA damage-induced apoptosis by acting as a transcriptional coactivator of proapoptotic target genes of the transcriptional activator E2F1. Likely to play a role in ribosome biogenesis by targeting serine/threonine protein phosphatase PP1 to the nucleolus. Involved in regulation of mRNA splicing (By similarity). Inhibits SIPA1 GTPase activity (By similarity). Involved in regulating expression of extracellular matrix genes (By similarity). Associates with chromatin and may play a role in modulating chromatin structure
**Q52LJ0**	FA98B	Protein FAM98B OS=Homo sapiens OX=9606 GN=FAM98B PE=1 SV=1	Positively stimulates PRMT1-induced protein arginine dimethylated arginine methylation. Promotes colorectal cancer cell malignancy
**Q8NCA5**	FA98A	Protein FAM98A OS=Homo sapiens OX=9606 GN=FAM98A PE=1 SV=1	Positively stimulates PRMT1-induced protein arginine methylation. Involved in skeletal homeostasis (By similarity). Positively regulates lysosome peripheral distribution and ruffled border formation in osteoclasts (By similarity). Promotes colorectal cancer cell malignancy
**Q9H0D6**	XRN2	5'-3' exoribonuclease 2 OS=Homo sapiens OX=9606 GN=XRN2 PE=1 SV=1	Possesses 5'->3' exoribonuclease activity (By similarity). May promote the termination of transcription by RNA polymerase II. During transcription termination, cleavage at the polyadenylation site liberates a 5' fragment which is subsequently processed to form the mature mRNA and a 3' fragment which remains attached to the elongating polymerase. The processive degradation of this 3' fragment by this protein may promote termination of transcription. Binds to RNA polymerase II (RNAp II) transcription termination R-loops formed by G-rich pause sites (950 aa)
**Q8WWK9**	CKAP2	Cytoskeleton-associated protein 2 OS=Homo sapiens OX=9606 GN=CKAP2 PE=1 SV=1	Possesses microtubule stabilizing properties. Involved in regulating aneuploidy, cell cycling, and cell death in a p53/TP53-dependent manner (By similarity) (683 aa)

**Q9P2V4**	LRIT1	Leucine-rich repeat, immunoglobulin-like domain and transmembrane domain-containing protein 1 OS=Homo sapiens OX=9606 GN=LRIT1 PE=2 SV=1	Possible role in phototransduction
**Q9Y5G5**	PCDG8	Protocadherin gamma-A8 OS=Homo sapiens OX=9606 GN=PCDHGA8 PE=2 SV=1	Potential calcium-dependent cell-adhesion protein. May be involved in the establishment and maintenance of specific neuronal connections in the brain
**Q15398**	DLGP5	Disks large-associated protein 5 OS=Homo sapiens OX=9606 GN=DLGAP5 PE=1 SV=2	Potential cell cycle regulator that may play a role in carcinogenesis of cancer cells. Mitotic phosphoprotein regulated by the ubiquitin-proteasome pathway. Key regulator of adherens junction integrity and differentiation that may be involved in CDH1-mediated adhesion and signaling in epithelial cells (846 aa)
**Q96A73**	P33MX	Putative monooxygenase p33MONOX OS=Homo sapiens OX=9606 GN=KIAA1191 PE=1 SV=1	Potential NADPH-dependent oxidoreductase. May be involved in the regulation of neuronal survival, differentiation and axonal outgrowth
**Q15404**	RSU1	Ras suppressor protein 1 OS=Homo sapiens OX=9606 GN=RSU1 PE=1 SV=3	Potentially plays a role in the Ras signal transduction pathway. Capable of suppressing v-Ras transformation in vitro (277 aa)
**P67775**	PP2AA	Serine/threonine-protein phosphatase 2A catalytic subunit alpha isoform OS=Homo sapiens OX=9606 GN=PPP2CA PE=1 SV=1	PP2A is the major phosphatase for microtubule-associated proteins (MAPs). PP2A can modulate the activity of phosphorylase B kinase casein kinase 2, mitogen-stimulated S6 kinase, and MAP-2 kinase. Cooperates with SGO2 to protect centromeric cohesin from separase-mediated cleavage in oocytes specifically during meiosis I (By similarity). Can dephosphorylate SV40 large T antigen and p53/TP53. Activates RAF1 by dephosphorylating it at 'Ser-259'
**P23284**	PPIB	Peptidyl-prolyl cis-trans isomerase B OS=Homo sapiens OX=9606 GN=PPIB PE=1 SV=2	PPIases accelerate the folding of proteins. It catalyzes the cis-trans isomerization of proline imidic peptide bonds in oligopeptides
**P62937**	PPIA	Peptidyl-prolyl cis-trans isomerase A OS=Homo sapiens OX=9606 GN=PPIA PE=1 SV=2	PPIases accelerate the folding of proteins. It catalyzes the cis-trans isomerization of proline imidic peptide bonds in oligopeptides
**Q9H2H8**	PPIL3	Peptidyl-prolyl cis-trans isomerase-like 3 OS=Homo sapiens OX=9606 GN=PPIL3 PE=1 SV=1	PPIases accelerate the folding of proteins. It catalyzes the cis-trans isomerization of proline imidic peptide bonds in oligopeptides

**P52272**	HNRPM	Heterogeneous nuclear ribonucleoprotein M OS=Homo sapiens OX=9606 GN=HNRNPM PE=1 SV=3	Pre-mRNA binding protein in vivo, binds avidly to poly(G) and poly(U) RNA homopolymers in vitro. Involved in splicing. Acts as a receptor for carcinoembryonic antigen in Kupffer cells, may initiate a series of signaling events leading to tyrosine phosphorylation of proteins and induction of IL-1 alpha, IL-6, IL-10 and tumor necrosis factor alpha cytokines (730 aa)
**O43143**	DHX15	Pre-mRNA-splicing factor ATP-dependent RNA helicase DHX15 OS=Homo sapiens OX=9606 GN=DHX15 PE=1 SV=2	Pre-mRNA processing factor involved in disassembly of spliceosomes after the release of mature mRNA. In cooperation with TFIP11 seem to be involved in the transition of the U2, U5 and U6 snRNP-containing IL complex to the snRNP-free IS complex leading to efficient debranching and turnover of excised introns
**Q96IZ0**	PAWR	PRKC apoptosis WT1 regulator protein OS=Homo sapiens OX=9606 GN=PAWR PE=1 SV=1	Pro-apoptopic protein capable of selectively inducing apoptosis in cancer cells, sensitizing the cells to diverse apoptotic stimuli and causing regression of tumors in animal models. Induces apoptosis in certain cancer cells by activation of the Fas prodeath pathway and coparallel inhibition of NF-kappa-B transcriptional activity. Inhibits the transcriptional activation and augments the transcriptional repression mediated by WT1. Down- regulates the anti-apoptotic protein BCL2 via its interaction with WT1. Seems also to be a transcriptional repress [...] (340 aa)
**Q6P158**	DHX57	Putative ATP-dependent RNA helicase DHX57 OS=Homo sapiens OX=9606 GN=DHX57 PE=1 SV=2	Probable ATP-binding RNA helicase
**Q9UQN3**	CHM2B	Charged multivesicular body protein 2b OS=Homo sapiens OX=9606 GN=CHMP2B PE=1 SV=1	Probable core component of the endosomal sorting required for transport complex III (ESCRT-III) which is involved in multivesicular bodies (MVBs) formation and sorting of endosomal cargo proteins into MVBs. MVBs contain intraluminal vesicles (ILVs) that are generated by invagination and scission from the limiting membrane of the endosome and mostly are delivered to lysosomes enabling degradation of membrane proteins, such as stimulated growth factor receptors, lysosomal enzymes and lipids. The MVB pathway appears to require the sequential functio [...] (213 aa)
**Q8IY81**	SPB1	pre-rRNA processing protein FTSJ3 OS=Homo sapiens OX=9606 GN=FTSJ3 PE=1 SV=2	Probable methyltransferase involved in the processing of the 34S pre-rRNA to 18S rRNA and in 40S ribosomal subunit formation

**Q8WWQ0**	PHIP	PH-interacting protein OS=Homo sapiens OX=9606 GN=PHIP PE=1 SV=2	Probable regulator of the insulin and insulin-like growth factor signaling pathways. Stimulates cell proliferation through regulation of cyclin transcription and has an anti- apoptotic activity through AKT1 phosphorylation and activation. Plays a role in the regulation of cell morphology and cytoskeletal organization
**Q9HCE1**	MOV10	Helicase MOV-10 OS=Homo sapiens OX=9606 GN=MOV10 PE=1 SV=2	Probable RNA helicase. Required for miRNA-mediated gene silencing by the RNA-induced silencing complex (RISC). Required for both miRNA-mediated translational repression and miRNA- mediated cleavage of complementary mRNAs by RISC
**Q9NVP1**	DDX18	ATP-dependent RNA helicase DDX18 OS=Homo sapiens OX=9606 GN=DDX18 PE=1 SV=2	Probable RNA-dependent helicase
**P35908**	K22E	Keratin, type II cytoskeletal 2 epidermal OS=Homo sapiens OX=9606 GN=KRT2 PE=1 SV=2	Probably contributes to terminal cornification. Associated with keratinocyte activation, proliferation and keratinization
**O60739**	EIF1B	Eukaryotic translation initiation factor 1b OS=Homo sapiens OX=9606 GN=EIF1B PE=1 SV=2	Probably involved in translation (113 aa)
**Q9Y520**	PRC2C	Protein PRRC2C OS=Homo sapiens OX=9606 GN=PRRC2C PE=1 SV=4	Proline rich coiled-coil 2C (2817 aa); PRRC2C (Proline Rich Coiled-Coil 2C) is a Protein Coding gene. Gene Ontology (GO) annotations related to this gene include protein C-terminus binding. An important paralog of this gene is PRRC2B.
**O00178**	GTPB1	GTP-binding protein 1 OS=Homo sapiens OX=9606 GN=GTPBP1 PE=1 SV=3	Promotes degradation of target mRNA species. Plays a role in the regulation of circadian mRNA stability. Binds GTP and has GTPase activity (By similarity) (669 aa)
**P02765**	FETUA	Alpha-2-HS-glycoprotein OS=Homo sapiens OX=9606 GN=AHSG PE=1 SV=2	Promotes endocytosis, possesses opsonic properties and influences the mineral phase of bone. Shows affinity for calcium and barium ions
**Q07889**	SOS1	Son of sevenless homolog 1 OS=Homo sapiens OX=9606 GN=SOS1 PE=1 SV=1	Promotes the exchange of Ras-bound GDP by GTP. Probably by promoting Ras activation, regulates phosphorylation of MAP kinase MAPK3 in response to EGF. Catalytic component of a trimeric complex that participates in transduction of signals from Ras to Rac by promoting the Rac-specific guanine nucleotide exchange factor (GEF) activity (By similarity)
**Q8WU90**	ZC3HF	Zinc finger CCCH domain-containing protein 15 OS=Homo sapiens OX=9606 GN=ZC3H15 PE=1 SV=1	Protects DRG1 from proteolytic degradation

**P30101**	PDIA3	Protein disulfide-isomerase A3 OS=Homo sapiens OX=9606 GN=PDIA3 PE=1 SV=4	Protein disulfide isomerase family A member 3; This gene encodes a protein of the endoplasmic reticulum that interacts with lectin chaperones calreticulin and calnexin to modulate folding of newly synthesized glycoproteins. The protein was once thought to be a phospholipase; however, it has been demonstrated that the protein actually has protein disulfide isomerase activity. It is thought that complexes of lectins and this protein mediate protein folding by promoting formation of disulfide bonds in their glycoprotein substrates. This protein also functions as a molecular chaperone that prevents the formation of protein aggregates.
**Q14554**	PDIA5	Protein disulfide-isomerase A5 OS=Homo sapiens OX=9606 GN=PDIA5 PE=1 SV=1	Protein disulfide isomerase family A member 5 (519 aa); This gene encodes a member of the disulfide isomerase (PDI) family of endoplasmic reticulum (ER) proteins that catalyze protein folding and thiol-disulfide interchange reactions. The encoded protein has an N-terminal ER-signal sequence, three catalytically active thioredoxin (TRX) domains, a TRX-like domain, and a C-terminal ER-retention sequence. The N-terminal TRX-like domain is the primary binding site for the major ER chaperone calreticulin and possibly other proteins and substrates as well. Alternative splicing results in multiple protein- and non-protein-coding transcript variants
**P36873**	PP1G	Serine/threonine-protein phosphatase PP1-gamma catalytic subunit OS=Homo sapiens OX=9606 GN=PPP1CC PE=1 SV=1	Protein phosphatase that associates with over 200 regulatory proteins to form highly specific holoenzymes which dephosphorylate hundreds of biological targets. Protein phosphatase 1 (PP1) is essential for cell division, and participates in the regulation of glycogen metabolism, muscle contractility and protein synthesis. Dephosphorylates RPS6KB1. Involved in regulation of ionic conductances and long-term synaptic plasticity. May play an important role in dephosphorylating substrates such as the postsynaptic density- asso [...] (337 aa)
**P18433**	PTPRA	Receptor-type tyrosine-protein phosphatase alpha OS=Homo sapiens OX=9606 GN=PTPRA PE=1 SV=3	Protein tyrosine phosphatase, receptor type A
**Q13206**	DDX10	Probable ATP-dependent RNA helicase DDX10 OS=Homo sapiens OX=9606 GN=DDX10 PE=1 SV=2	Putative ATP-dependent RNA helicase

**Q01780**	EXOSX	Exosome component 10 OS=Homo sapiens OX=9606 GN=EXOSC10 PE=1 SV=2	Putative catalytic component of the RNA exosome complex which has 3'->5' exoribonuclease activity and participates in a multitude of cellular RNA processing and degradation events. In the nucleus, the RNA exosome complex is involved in proper maturation of stable RNA species such as rRNA, snRNA and snoRNA, in the elimination of RNA processing by-products and non-coding 'pervasive' transcripts, such as antisense RNA species and promoter-upstream transcripts (PROMPTs), and of mRNAs with processing defects, thereby limiting or excluding their export to the cytoplasm. [...] (885 aa)
**Q49A26**	GLYR1	Putative oxidoreductase GLYR1 OS=Homo sapiens OX=9606 GN=GLYR1 PE=1 SV=4	Putative oxidoreductase that is recruited on chromatin and promotes KDM1B demethylase activity. Recognizes and binds trimethylated 'Lys-36' of histone H3 (H3K36me3). Regulates p38 MAP kinase activity by mediating stress activation of p38alpha/MAPK14 and specifically regulating MAPK14 signaling. Indirectly promotes phosphorylation of MAPK14 and activation of ATF2. The phosphorylation of MAPK14 requires upstream activity of MAP2K4 and MAP2K6
**O75044**	SRGP2	SLIT-ROBO Rho GTPase-activating protein 2 OS=Homo sapiens OX=9606 GN=SRGAP2 PE=1 SV=3	RAC1 GTPase activating protein (GAP) that binds and deforms membranes, and regulates actin dynamics to regulate cell migration and differentiation. Plays an important role in different aspects of neuronal morphogenesis and migration mainly during development of the cerebral cortex. This includes the biogenesis of neurites, where it is required for both axons and dendrites outgrowth, and the maturation of the dendritic spines. Also stimulates the branching of the leading process and negatively regulates neuron radial migration in the cerebral c [...] (1071 aa)
**Q13418**	ILK	Integrin-linked protein kinase OS=Homo sapiens OX=9606 GN=ILK PE=1 SV=2	Receptor-proximal protein kinase regulating integrin-mediated signal transduction. May act as a mediator of inside-out integrin signaling. Focal adhesion protein part of the complex ILK-PINCH. This complex is considered to be one of the convergence points of integrin- and growth factor-signaling pathway. Could be implicated in mediating cell architecture, adhesion to integrin substrates and anchorage- dependent growth in epithelial cells. Phosphorylates beta-1 and beta-3 integrin subunit on serine and threonine residues, but also AKT1 and GSK3B (452 aa)

**P08651**	NFIC	Nuclear factor 1 C-type OS=Homo sapiens OX=9606 GN=NFIC PE=1 SV=2	Recognizes and binds the palindromic sequence 5'-TTGGCNNNNNGCCAA-3' present in viral and cellular promoters and in the origin of replication of adenovirus type 2. These proteins are individually capable of activating transcription and replication
**Q12857**	NFIA	Nuclear factor 1 A-type OS=Homo sapiens OX=9606 GN=NFIA PE=1 SV=2	Recognizes and binds the palindromic sequence 5'-TTGGCNNNNNGCCAA-3' present in viral and cellular promoters and in the origin of replication of adenovirus type 2. These proteins are individually capable of activating transcription and replication (554 aa)
**P17480**	UBF1	Nucleolar transcription factor 1 OS=Homo sapiens OX=9606 GN=UBTF PE=1 SV=1	Recognizes the ribosomal RNA gene promoter and activates transcription mediated by RNA polymerase I through cooperative interactions with the transcription factor SL1/TIF-IB complex. It binds specifically to the upstream control element (764 aa)
**Q07666**	KHDR1	KH domain-containing, RNA-binding, signal transduction-associated protein 1 OS=Homo sapiens OX=9606 GN=KHDRBS1 PE=1 SV=1	Recruited and tyrosine phosphorylated by several receptor systems, for example the T-cell, leptin and insulin receptors. Once phosphorylated, functions as an adapter protein in signal transduction cascades by binding to SH2 and SH3 domain- containing proteins. Role in G2-M progression in the cell cycle. Represses CBP-dependent transcriptional activation apparently by competing with other nuclear factors for binding to CBP. Also acts as a putative regulator of mRNA stability and/or translation rates and mediate [...] (443 aa)
**O76021**	RL1D1	Ribosomal L1 domain-containing protein 1 OS=Homo sapiens OX=9606 GN=RSL1D1 PE=1 SV=3	Regulates cellular senescence through inhibition of PTEN translation. Acts as a pro-apoptotic regulator in response to DNA damage
**O95747**	OXSR1	Serine/threonine-protein kinase OSR1 OS=Homo sapiens OX=9606 GN=OXSR1 PE=1 SV=1	Regulates downstream kinases in response to environmental stress. May also have a function in regulating the actin cytoskeleton
**Q96AE4**	FUBP1	Far upstream element-binding protein 1 OS=Homo sapiens OX=9606 GN=FUBP1 PE=1 SV=3	Regulates MYC expression by binding to a single-stranded far-upstream element (FUSE) upstream of the MYC promoter. May act both as activator and repressor of transcription (644 aa)
**Q9BRS8**	LARP6	La-related protein 6 OS=Homo sapiens OX=9606 GN=LARP6 PE=1 SV=1	Regulates the coordinated translation of type I collagen alpha-1 and alpha-2 mRNAs, CO1A1 and CO1A2. Stabilizes mRNAs through high-affinity binding of a stem-loop structure in their 5' UTR. This regulation requires VIM and MYH10 filaments, and the helicase DHX9

**Q8WXF1**	PSPC1	Paraspeckle component 1 OS=Homo sapiens OX=9606 GN=PSPC1 PE=1 SV=1	Regulates, cooperatively with NONO and SFPQ, androgen receptor-mediated gene transcription activity in Sertoli cell line (By similarity). Binds to poly(A), poly(G) and poly(U) RNA homopolymers. Regulates the circadian clock by repressing the transcriptional activator activity of the CLOCK-ARNTL/BMAL1 heterodimer (By similarity). Together with NONO, required for the formation of nuclear paraspeckles. Plays a role in the regulation of DNA virus-mediated innate immune response by assembling into the HDP-RNP complex, a complex that serves as a platform for IRF3 pho [...] (523 aa)
**Q69YH5**	CDCA2	Cell division cycle-associated protein 2 OS=Homo sapiens OX=9606 GN=CDCA2 PE=1 SV=2	Regulator of chromosome structure during mitosis required for condensin-depleted chromosomes to retain their compact architecture through anaphase. Acts by mediating the recruitment of phopsphatase PP1-gamma subunit (PPP1CC) to chromatin at anaphase and into the following interphase. At anaphase onset, its association with chromatin targets a pool of PPP1CC to dephosphorylate substrates
**P60660**	MYL6	Myosin light polypeptide 6 OS=Homo sapiens OX=9606 GN=MYL6 PE=1 SV=2	Regulatory light chain of myosin. Does not bind calcium
**Q15021**	CND1	Condensin complex subunit 1 OS=Homo sapiens OX=9606 GN=NCAPD2 PE=1 SV=3	Regulatory subunit of the condensin complex, a complex required for conversion of interphase chromatin into mitotic-like condense chromosomes. The condensin complex probably introduces positive supercoils into relaxed DNA in the presence of type I topoisomerases and converts nicked DNA into positive knotted forms in the presence of type II topoisomerases. May target the condensin complex to DNA via its C-terminal domain
**P11387**	TOP1	DNA topoisomerase 1 OS=Homo sapiens OX=9606 GN=TOP1 PE=1 SV=2	Releases the supercoiling and torsional tension of DNA introduced during the DNA replication and transcription by transiently cleaving and rejoining one strand of the DNA duplex. Introduces a single-strand break via transesterification at a target site in duplex DNA. The scissile phosphodiester is attacked by the catalytic tyrosine of the enzyme, resulting in the formation of a DNA-(3’-phosphotyrosyl)-enzyme intermediate and the expulsion of a 5’-OH DNA strand. The free DNA strand then rotates around the intact phosphodiester bond on the opposing strand, thus remov [...] (765 aa)
**Q2NL82**	TSR1	Pre-rRNA-processing protein TSR1 homolog OS=Homo sapiens OX=9606 GN=TSR1 PE=1 SV=1	Required during maturation of the 40S ribosomal subunit in the nucleolus (804 aa)

**Q13601**	KRR1	KRR1 small subunit processome component homolog OS=Homo sapiens OX=9606 GN=KRR1 PE=1 SV=4	Required for 40S ribosome biogenesis. Involved in nucleolar processing of pre-18S ribosomal RNA and ribosome assembly (By similarity) (381 aa)
**Q9Y2X3**	NOP58	Nucleolar protein 58 OS=Homo sapiens OX=9606 GN=NOP58 PE=1 SV=1	Required for 60S ribosomal subunit biogenesis (By similarity). Core component of box C/D small nucleolar ribonucleoprotein (snoRNP) particles. Required for the biogenesis of box C/D snoRNAs such as U3, U8 and U14 snoRNAs
**Q96T23**	RSF1	Remodeling and spacing factor 1 OS=Homo sapiens OX=9606 GN=RSF1 PE=1 SV=2	Required for assembly of regular nucleosome arrays by the RSF chromatin-remodeling complex. Facilitates transcription of hepatitis B virus (HBV) genes by the pX transcription activator. In case of infection by HBV, together with pX, it represses TNF-alpha induced NF-kappa-B transcription activation. Represses transcription when artificially recruited to chromatin by fusion to a heterogeneous DNA binding domain
**Q8TDN6**	BRX1	Ribosome biogenesis protein BRX1 homolog OS=Homo sapiens OX=9606 GN=BRIX1 PE=1 SV=2	Required for biogenesis of the 60S ribosomal subunit (353 aa)
**Q14676**	MDC1	Mediator of DNA damage checkpoint protein 1 OS=Homo sapiens OX=9606 GN=MDC1 PE=1 SV=3	Required for checkpoint mediated cell cycle arrest in response to DNA damage within both the S phase and G2/M phases of the cell cycle. May serve as a scaffold for the recruitment of DNA repair and signal transduction proteins to discrete foci of DNA damage marked by 'Ser-139' phosphorylation of histone H2AFX. Also required for downstream events subsequent to the recruitment of these proteins. These include phosphorylation and activation of the ATM, CHEK1 and CHEK2 kinases, and stabilization of TP53 and apoptosis. ATM and CHEK2 may also be a [...] (2089 aa)
**Q9P258**	RCC2	Protein RCC2 OS=Homo sapiens OX=9606 GN=RCC2 PE=1 SV=2	Required for completion of mitosis and cytokinesis. May function as a guanine nucleotide exchange factor for the small GTPase RAC1 (522 aa)
**Q7Z7K6**	CENPV	Centromere protein V OS=Homo sapiens OX=9606 GN=CENPV PE=1 SV=1	Required for distribution of pericentromeric heterochromatin in interphase nuclei and for centromere formation and organization, chromosome alignment and cytokinesis (272 aa)

**Q16531**	DDB1	DNA damage-binding protein 1 OS=Homo sapiens OX=9606 GN=DDB1 PE=1 SV=1	Required for DNA repair. Binds to DDB2 to form the UV- damaged DNA-binding protein complex (the UV-DDB complex). The UV- DDB complex may recognize UV-induced DNA damage and recruit proteins of the nucleotide excision repair pathway (the NER pathway) to initiate DNA repair. The UV-DDB complex preferentially binds to cyclobutane pyrimidine dimers (CPD), 6-4 photoproducts (6-4 PP), apurinic sites and short mismatches. Also appears to function as a component of numerous distinct DCX (DDB1-CUL4-X-box) E3 ubiquitin-protein ligase complexes which mediate the ubiq [...] (1140 aa)
**Q9H902**	REEP1	Receptor expression-enhancing protein 1 OS=Homo sapiens OX=9606 GN=REEP1 PE=1 SV=1	Required for endoplasmic reticulum (ER) network formation, shaping and remodeling
**Q9Y6K1**	DNM3A	DNA (cytosine-5)-methyltransferase 3A OS=Homo sapiens OX=9606 GN=DNMT3A PE=1 SV=4	Required for genome-wide de novo methylation and is essential for the establishment of DNA methylation patterns during development. DNA methylation is coordinated with methylation of histones. It modifies DNA in a non-processive manner and also methylates non-CpG sites. May preferentially methylate DNA linker between 2 nucleosomal cores and is inhibited by histone H1. Plays a role in paternal and maternal imprinting. Required for methylation of most imprinted loci in germ cells. Acts as a transcriptional corepressor for ZBTB18. Recruited to trimet [...] (912 aa)
**Q96AG4**	LRC59	Leucine-rich repeat-containing protein 59 OS=Homo sapiens OX=9606 GN=LRRC59 PE=1 SV=1	Required for nuclear import of FGF1, but not that of FGF2. Might regulate nuclear import of exogenous FGF1 by facilitating interaction with the nuclear import machinery and by transporting cytosolic FGF1 to, and possibly through, the nuclear pores (307 aa)
**P39019**	RS19	40S ribosomal protein S19 OS=Homo sapiens OX=9606 GN=RPS19 PE=1 SV=2	Required for pre-rRNA processing and maturation of 40S ribosomal subunits
**P62847**	RS24	40S ribosomal protein S24 OS=Homo sapiens OX=9606 GN=RPS24 PE=1 SV=1	Required for processing of pre-rRNA and maturation of 40S ribosomal subunits
**Q9BWT6**	MND1	Meiotic nuclear division protein 1 homolog OS=Homo sapiens OX=9606 GN=MND1 PE=1 SV=1	Required for proper homologous chromosome pairing and efficient cross-over and intragenic recombination during meiosis (By similarity). Stimulates both DMC1- and RAD51-mediated homologous strand assimilation, which is required for the resolution of meiotic double-strand breaks (205 aa)
**Q96CT7**	CC124	Coiled-coil domain-containing protein 124 OS=Homo sapiens OX=9606 GN=CCDC124 PE=1 SV=1	Required for proper progression of late cytokinetic stages
**P62081**	RS7	40S ribosomal protein S7 OS=Homo sapiens OX=9606 GN=RPS7 PE=1 SV=1	Required for rRNA maturation

**P08865**	RSSA	40S ribosomal protein SA OS=Homo sapiens OX=9606 GN=RPSA PE=1 SV=4	Required for the assembly and/or stability of the 40S ribosomal subunit. Required for the processing of the 20S rRNA- precursor to mature 18S rRNA in a late step of the maturation of 40S ribosomal subunits. Also functions as a cell surface receptor for laminin. Plays a role in cell adhesion to the basement membrane and in the consequent activation of signaling transduction pathways. May play a role in cell fate determination and tissue morphogenesis. Acts as a PPP1R16B-dependent substrate of PPP1CA (295 aa)
**Q9Y3A5**	SBDS	Ribosome maturation protein SBDS OS=Homo sapiens OX=9606 GN=SBDS PE=1 SV=4	Required for the assembly of mature ribosomes and ribosome biogenesis. Together with EFL1, triggers the GTP- dependent release of EIF6 from 60S pre-ribosomes in the cytoplasm, thereby activating ribosomes for translation competence by allowing 80S ribosome assembly and facilitating EIF6 recycling to the nucleus, where it is required for 60S rRNA processing and nuclear export. Required for normal levels of protein synthesis. May play a role in cellular stress resistance. May play a role in cellular response to DNA damage. May play a role in cell prolife [...] (250 aa)
**P23588**	IF4B	Eukaryotic translation initiation factor 4B OS=Homo sapiens OX=9606 GN=EIF4B PE=1 SV=2	Required for the binding of mRNA to ribosomes. Functions in close association with EIF4-F and EIF4-A. Binds near the 5'- terminal cap of mRNA in presence of EIF-4F and ATP. Promotes the ATPase activity and the ATP-dependent RNA unwinding activity of both EIF4-A and EIF4-F (616 aa)
**Q9NWB6**	ARGL1	Arginine and glutamate-rich protein 1 OS=Homo sapiens OX=9606 GN=ARGLU1 PE=1 SV=1	Required for the estrogen-dependent expression of ESR1 target genes. Can act in cooperation with MED1 (273 aa)
**Q99848**	EBP2	Probable rRNA-processing protein EBP2 OS=Homo sapiens OX=9606 GN=EBNA1BP2 PE=1 SV=2	Required for the processing of the 27S pre-rRNA
**P18077**	RL35A	60S ribosomal protein L35a OS=Homo sapiens OX=9606 GN=RPL35A PE=1 SV=2	Required for the proliferation and viability of hematopoietic cells. Plays a role in 60S ribosomal subunit formation. The protein was found to bind to both initiator and elongator tRNAs and consequently was assigned to the P site or P and A site (110 aa)

**Q15642**	CIP4	Cdc42-interacting protein 4 OS=Homo sapiens OX=9606 GN=TRIP10 PE=1 SV=3	Required for translocation of GLUT4 to the plasma membrane in response to insulin signaling (By similarity). Required to coordinate membrane tubulation with reorganization of the actin cytoskeleton during endocytosis. Binds to lipids such as phosphatidylinositol 4,5-bisphosphate and phosphatidylserine and promotes membrane invagination and the formation of tubules. Also promotes CDC42-induced actin polymerization by recruiting WASL/N- WASP which in turn activates the Arp2/3 complex. Actin polymerization may promote the fission of membrane tubules to form en [...] (601 aa)
**P46013**	KI67	Proliferation marker protein Ki-67 OS=Homo sapiens OX=9606 GN=MKI67 PE=1 SV=2	Required to maintain individual mitotic chromosomes dispersed in the cytoplasm following nuclear envelope disassembly. Associates with the surface of the mitotic chromosome, the perichromosomal layer, and covers a substantial fraction of the chromosome surface. Prevents chromosomes from collapsing into a single chromatin mass by forming a steric and electrostatic charge barrier- the protein has a high net electrical charge and acts as a surfactant, dispersing chromosomes and enabling independent chromosome motility. Binds DNA, with a preference for s [...] (3256 aa)
**Q92769**	HDAC2	Histone deacetylase 2 OS=Homo sapiens OX=9606 GN=HDAC2 PE=1 SV=2	Responsible for the deacetylation of lysine residues on the N-terminal part of the core histones (H2A, H2B, H3 and H4). Histone deacetylation gives a tag for epigenetic repression and plays an important role in transcriptional regulation, cell cycle progression and developmental events. Histone deacetylases act via the formation of large multiprotein complexes. Forms transcriptional repressor complexes by associating with MAD, SIN3, YY1 and N-COR. Interacts in the late S-phase of DNA-replication with DNMT1 in the other transcriptional repressor complex composed o [...] (488 aa)
**P12277**	KCRB	Creatine kinase B-type OS=Homo sapiens OX=9606 GN=CKB PE=1 SV=1	Reversibly catalyzes the transfer of phosphate between ATP and various phosphogens (e.g. creatine phosphate). Creatine kinase isoenzymes play a central role in energy transduction in tissues with large, fluctuating energy demands, such as skeletal muscle, heart, brain and spermatozoa (381 aa)

**P12532**	KCRU	Creatine kinase U-type, mitochondrial OS=Homo sapiens OX=9606 GN=CKMT1A PE=1 SV=1	Reversibly catalyzes the transfer of phosphate between ATP and various phosphogens (e.g. creatine phosphate). Creatine kinase isoenzymes play a central role in energy transduction in tissues with large, fluctuating energy demands, such as skeletal muscle, heart, brain and spermatozoa (417 aa)
**P05388**	RLA0	60S acidic ribosomal protein P0 OS=Homo sapiens OX=9606 GN=RPLP0 PE=1 SV=1	Ribosomal protein P0 is the functional equivalent of E.coli protein L10 (317 aa)
**P42696**	RBM34	RNA-binding protein 34 OS=Homo sapiens OX=9606 GN=RBM34 PE=1 SV=2	RNA binding motif containing; This gene encodes a member of the RNA-binding motif family of RNA recognition motif proteins. The encoded protein contains an RNA-binding domain made up of two RNA recognition motif subdomains referred to as RNA recognition motif-1 and RNA recognition motif-2. Alternative splicing results in multiple transcript variants.
**Q9BYG3**	MK67I	MKI67 FHA domain-interacting nucleolar phosphoprotein OS=Homo sapiens OX=9606 GN=NIFK PE=1 SV=1	RNA binding motif containing (293 aa); This gene encodes a protein that interacts with the forkhead-associated domain of the Ki-67 antigen. The encoded protein may bind RNA and may play a role in mitosis and cell cycle progression. Multiple pseudogenes exist on chromosomes 5, 10, 12, 15, and 19
**Q9NTZ6**	RBM12	RNA-binding protein 12 OS=Homo sapiens OX=9606 GN=RBM12 PE=1 SV=1	RNA binding motif containing (932 aa); This gene encodes a protein that contains several RNA-binding motifs, potential transmembrane domains, and proline-rich regions. This gene and the gene for copine I overlap at map location 20q11.21. Alternative splicing in the 5' UTR results in four transcript variants. All variants encode the same protein.
**Q9H0A0**	NAT10	RNA cytidine acetyltransferase OS=Homo sapiens OX=9606 GN=NAT10 PE=1 SV=2	RNA cytidine acetyltransferase with specificity toward both 18S rRNA and tRNAs. Catalyzes the formation of N(4)-acetylcytidine (ac4C) at positions 1337 and 1842 in 18S rRNA (By similarity). Required for early nucleolar cleavages of precursor rRNA at sites A0, A1 and A2 during 18S rRNA synthesis. Catalyzes the formation of ac4C in serine and leucine tRNAs (By similarity). Requires the tRNA-binding adapter protein THUMBD1 for full tRNA acetyltransferase activity but not for 18S rRNA acetylation. Can acetylate both histones and microtubules. Histone acetyla [...] (1025 aa)

**Q9NR30**	DDX21	Nucleolar RNA helicase 2 OS=Homo sapiens OX=9606 GN=DDX21 PE=1 SV=5	RNA helicase that acts as a sensor of the transcriptional status of both RNA polymerase (Pol) I and II- promotes ribosomal RNA (rRNA) processing and transcription from polymerase II (Pol II). Binds various RNAs, such as rRNAs, snoRNAs, 7SK and, at lower extent, mRNAs. In the nucleolus, localizes to rDNA locus, where it directly binds rRNAs and snoRNAs, and promotes rRNA transcription, processing and modification. Required for rRNA 2'- O-methylation, possibly by promoting the recruitment of late- acting snoRNAs SNORD56 and SNORD58 with pre-ribosomal complexes. [...] (783 aa)
**O75643**	U520	U5 small nuclear ribonucleoprotein 200 kDa helicase OS=Homo sapiens OX=9606 GN=SNRNP200 PE=1 SV=2	RNA helicase that plays an essential role in pre-mRNA splicing as component of the U5 snRNP and U4/U6-U5 tri-snRNP complexes. Involved in spliceosome assembly, activation and disassembly. Mediates changes in the dynamic network of RNA-RNA interactions in the spliceosome. Catalyzes the ATP-dependent unwinding of U4/U6 RNA duplices, an essential step in the assembly of a catalytically active spliceosome (2136 aa)
**Q08J23**	NSUN2	tRNA (cytosine(34)-C(5))-methyltransferase OS=Homo sapiens OX=9606 GN=NSUN2 PE=1 SV=2	RNA methyltransferase that methylates tRNAs, and possibly RNA polymerase III transcripts. Methylates cytosine to 5- methylcytosine (m5C) at positions 34 and 48 of intron-containing tRNA(Leu)(CAA) precursors, and at positions 48, 49 and 50 of tRNA(Gly)(GCC) precursors. May act downstream of Myc to regulate epidermal cell growth and proliferation. Required for proper spindle assembly and chromosome segregation, independently of its methyltransferase activity (767 aa)
**Q14152**	EIF3A	Eukaryotic translation initiation factor 3 subunit A OS=Homo sapiens OX=9606 GN=EIF3A PE=1 SV=1	RNA-binding component of the eukaryotic translation initiation factor 3 (eIF-3) complex, which is required for several steps in the initiation of protein synthesis. The eIF-3 complex associates with the 40S ribosome and facilitates the recruitment of eIF-1, eIF-1A, eIF- 2-GTP-methionyl-tRNAi and eIF-5 to form the 43S pre-initiation complex (43S PIC). The eIF-3 complex stimulates mRNA recruitment to the 43S PIC and scanning of the mRNA for AUG recognition. The eIF-3 complex is also required for disassembly and recycling of post-termi [...] (1382 aa)

**O00425**	IF2B3	Insulin-like growth factor 2 mRNA-binding protein 3 OS=Homo sapiens OX=9606 GN=IGF2BP3 PE=1 SV=2	RNA-binding factor that may recruit target transcripts to cytoplasmic protein-RNA complexes (mRNPs). This transcript 'caging' into mRNPs allows mRNA transport and transient storage. It also modulates the rate and location at which target transcripts encounter the translational apparatus and shields them from endonuclease attacks or microRNA-mediated degradation. Binds to the 3'-UTR of CD44 mRNA and stabilizes it, hence promotes cell adhesion and invadopodia formation in cancer cells. Binds to beta-actin/ACTB and MYC transcripts. Bin [...] (579 aa)
**Q9NZI8**	IF2B1	Insulin-like growth factor 2 mRNA-binding protein 1 OS=Homo sapiens OX=9606 GN=IGF2BP1 PE=1 SV=2	RNA-binding factor that recruits target transcripts to cytoplasmic protein-RNA complexes (mRNPs). This transcript 'caging' into mRNPs allows mRNA transport and transient storage. It also modulates the rate and location at which target transcripts encounter the translational apparatus and shields them from endonuclease attacks or microRNA-mediated degradation. Plays a direct role in the transport and translation of transcripts required for axonal regeneration in adult sensory neurons (By similarity). Regulates localized beta-actin/ACT [...] (577 aa)
**Q9Y6M1**	IF2B2	Insulin-like growth factor 2 mRNA-binding protein 2 OS=Homo sapiens OX=9606 GN=IGF2BP2 PE=1 SV=2	RNA-binding factor that recruits target transcripts to cytoplasmic protein-RNA complexes (mRNPs). This transcript 'caging' into mRNPs allows mRNA transport and transient storage. It also modulates the rate and location at which target transcripts encounter the translational apparatus and shields them from endonuclease attacks or microRNA-mediated degradation (By similarity). Binds to the 5'-UTR of the insulin-like growth factor 2 (IGF2) mRNAs. Binding is isoform-specific. Binds to beta- actin/ACTB and MYC transcripts
**P51116**	FXR2	Fragile X mental retardation syndrome-related protein 2 OS=Homo sapiens OX=9606 GN=FXR2 PE=1 SV=2	RNA-binding protein (673 aa); The protein encoded by this gene is a RNA binding protein containing two KH domains and one RCG box, which is similar to FMRP and FXR1. It associates with polyribosomes, predominantly with 60S large ribosomal subunits. This encoded protein may self-associate or interact with FMRP and FXR1. It may have a role in the development of fragile X cognitive disability syndrome.

**P51114**	FXR1	Fragile X mental retardation syndrome-related protein 1 OS=Homo sapiens OX=9606 GN=FXR1 PE=1 SV=3	RNA-binding protein required for embryonic and postnatal development of muscle tissue. May regulate intracellular transport and local translation of certain mRNAs (By similarity)
**Q9NUL3**	STAU2	Double-stranded RNA-binding protein Staufen homolog 2 OS=Homo sapiens OX=9606 GN=STAU2 PE=1 SV=2	RNA-binding protein required for the microtubule-dependent transport of neuronal RNA from the cell body to the dendrite. As protein synthesis occurs within the dendrite, the localization of specific mRNAs to dendrites may be a prerequisite for neurite outgrowth and plasticity at sites distant from the cell body (By similarity) (570 aa)
**Q15717**	ELAV1	ELAV-like protein 1 OS=Homo sapiens OX=9606 GN=ELAVL1 PE=1 SV=2	RNA-binding protein that binds to the 3'-UTR region of mRNAs and increases their stability. Involved in embryonic stem cells (ESCs) differentiation-preferentially binds mRNAs that are not methylated by N6-methyladenosine (m6A), stabilizing them, promoting ESCs differentiation (By similarity). Binds to poly-U elements and AU-rich elements (AREs) in the 3'-UTR of target mRNAs. Binds avidly to the AU-rich element in FOS and IL3/interleukin-3 mRNAs. In the case of the FOS AU-rich element, binds to a core element of 27 nucleotides that contain AUUUA, AUUUUA, and AUUUUU [...] (326 aa)
**Q12906**	ILF3	Interleukin enhancer-binding factor 3 OS=Homo sapiens OX=9606 GN=ILF3 PE=1 SV=3	RNA-binding protein that plays an essential role in the biogenesis of circular RNAs (circRNAs) which are produced by back- splicing circularization of pre-mRNAs. Within the nucleus, promotes circRNAs processing by stabilizing the regulatory elements residing in the flanking introns of the circularized exons. Plays thereby a role in the back-splicing of a subset of circRNAs. As a consequence, participates in a wide range of transcriptional and post-transcriptional processes. Upon viral infection, ILF3 accumulates in the cytoplasm and participates i [...] (898 aa)
**P38159**	RBMX	RNA-binding motif protein, X chromosome OS=Homo sapiens OX=9606 GN=RBMX PE=1 SV=3	RNA-binding protein that plays several role in the regulation of pre- and post-transcriptional processes. Implicated in tissue-specific regulation of gene transcription and alternative splicing of several pre-mRNAs. Binds to and stimulates transcription from the tumor suppressor TXNIP gene promoter; may thus be involved in tumor suppression. When associated with SAFB, binds to and stimulates transcription from the SREBF1 promoter. Associates with nascent mRNAs transcribed by RNA polymerase II. Component of the supraspliceosome complex that regul [...] (391 aa)

**Q6PKG0**	LARP1	La-related protein 1 OS=Homo sapiens OX=9606 GN=LARP1 PE=1 SV=2	RNA-binding protein that promotes translation of specific classes of mRNAs downstream of the mTORC1 complex. Associates with the mRNA 5'cap in an MTOR-dependent manner and associates with mRNAs containing a 5' terminal oligopyrimidine (5'TOP) motif, which is present in mRNAs encoding for ribosomal proteins and several components of the translation machinery. Associates with actively translating ribosomes via interaction with PABPC1/PABP and stimulates translation of mRNAs containing a 5'TOP, thereby regulating cell growth and proliferation. Positively regulates th [...] (1019 aa)
**Q96E39**	RMXL1	RNA binding motif protein, X-linked-like-1 OS=Homo sapiens OX=9606 GN=RBMXL1 PE=1 SV=1	RNA-binding protein which may be involved in pre-mRNA splicing (390 aa)
**Q96EP5**	DAZP1	DAZ-associated protein 1 OS=Homo sapiens OX=9606 GN=DAZAP1 PE=1 SV=1	RNA-binding protein, which may be required during spermatogenesis (407 aa)
**Q92900**	RENT1	Regulator of nonsense transcripts 1 OS=Homo sapiens OX=9606 GN=UPF1 PE=1 SV=2	RNA-dependent helicase and ATPase required for nonsense- mediated decay (NMD) of mRNAs containing premature stop codons. Is recruited to mRNAs upon translation termination and undergoes a cycle of phosphorylation and dephosphorylation; its phosphorylation appears to be a key step in NMD. Recruited by release factors to stalled ribosomes together with the SMG1C protein kinase complex to form the transient SURF (SMG1-UPF1-eRF1- eRF3) complex. In EJC-dependent NMD, the SURF complex associates with the exon junction complex (EJC) (located 50-55 or more [...] (1129 aa)
**Q9BWS9**	CHID1	Chitinase domain-containing protein 1 OS=Homo sapiens OX=9606 GN=CHID1 PE=1 SV=1	Saccharide- and LPS-binding protein with possible roles in pathogen sensing and endotoxin neutralization. Ligand-binding specificity relates to the length of the oligosaccharides, with preference for chitotetraose (in vitro)
**Q8WVM0**	TFB1M	Dimethyladenosine transferase 1, mitochondrial OS=Homo sapiens OX=9606 GN=TFB1M PE=1 SV=1	S-adenosyl-L-methionine-dependent methyltransferase which specifically dimethylates mitochondrial 12S rRNA at the conserved stem loop. Also required for basal transcription of mitochondrial DNA, probably via its interaction with POLRMT and TFAM. Stimulates transcription independently of the methyltransferase activity
**P02808**	STAT	Statherin OS=Homo sapiens OX=9606 GN=STATH PE=1 SV=2	Salivary protein that stabilizes saliva supersaturated with calcium salts by inhibiting the precipitation of calcium phosphate salts. It also modulates hydroxyapatite crystal formation on the tooth surface

**Q99996**	AKAP9	A-kinase anchor protein 9 OS=Homo sapiens OX=9606 GN=AKAP9 PE=1 SV=4	Scaffolding protein that assembles several protein kinases and phosphatases on the centrosome and Golgi apparatus. Required to maintain the integrity of the Golgi apparatus. Recruited to the Golgi apparatus by GM130/GOLGA2 and is required for microtubule nucleation at the cis-side of the Golgi apparatus. Required for association of the centrosomes with the poles of the bipolar mitotic spindle during metaphase. Forms a complex with PDE4DIP- the complex recruits CAMSAP2 to the Golgi apparatus, leading to tether non-centrosomal minus-end microtubules to the Golg [...] (3907 aa)
**Q9Y5K6**	CD2AP	CD2-associated protein OS=Homo sapiens OX=9606 GN=CD2AP PE=1 SV=1	Seems to act as an adapter protein between membrane proteins and the actin cytoskeleton. In collaboration with CBLC, modulates the rate of RET turnover and may act as regulatory checkpoint that limits the potency of GDNF on neuronal survival. Controls CBLC function, converting it from an inhibitor to a promoter of RET degradation (By similarity). May play a role in receptor clustering and cytoskeletal polarity in the junction between T-cell and antigen-presenting cell (By similarity). May anchor the podocyte slit diaphragm to the actin cytoskeleton in renal glom [...] (639 aa)
**Q13185**	CBX3	Chromobox protein homolog 3 OS=Homo sapiens OX=9606 GN=CBX3 PE=1 SV=4	Seems to be involved in transcriptional silencing in heterochromatin-like complexes. Recognizes and binds histone H3 tails methylated at ’Lys-9’, leading to epigenetic repression. May contribute to the association of the heterochromatin with the inner nuclear membrane through its interaction with lamin B receptor (LBR). Involved in the formation of functional kinetochore through interaction with MIS12 complex proteins. Contributes to the conversion of local chromatin to a heterochromatin-like repressive state through H3 ’Lys-9’ trimethylation, mediates the [...] (183 aa)
**P55145**	MANF	Mesencephalic astrocyte-derived neurotrophic factor OS=Homo sapiens OX=9606 GN=MANF PE=1 SV=3	Selectively promotes the survival of dopaminergic neurons of the ventral mid-brain. Modulates GABAergic transmission to the dopaminergic neurons of the substantia nigra. Enhances spontaneous, as well as evoked, GABAergic inhibitory postsynaptic currents in dopaminergic neurons (By similarity). Inhibits cell proliferation and endoplasmic reticulum (ER) stress-induced cell death (182 aa)

**Q92754**	AP2C	Transcription factor AP-2 gamma OS=Homo sapiens OX=9606 GN=TFAP2C PE=1 SV=1	Sequence-specific DNA-binding protein that interacts with inducible viral and cellular enhancer elements to regulate transcription of selected genes. AP-2 factors bind to the consensus sequence 5'-GCCNNNGGC-3' and activate genes involved in a large spectrum of important biological functions including proper eye, face, body wall, limb and neural tube development. They also suppress a number of genes including MCAM/MUC18, C/EBP alpha and MYC. Involved in the MTA1-mediated epigenetic regulation of ESR1 expression in breast cancer (450 aa)
**P78362**	SRPK2	SRSF protein kinase 2 OS=Homo sapiens OX=9606 GN=SRPK2 PE=1 SV=3	Serine/arginine-rich protein-specific kinase which specifically phosphorylates its substrates at serine residues located in regions rich in arginine/serine dipeptides, known as RS domains and is involved in the phosphorylation of SR splicing factors and the regulation of splicing. Promotes neuronal apoptosis by up-regulating cyclin-D1 (CCND1) expression. This is done by the phosphorylation of SRSF2, leading to the suppression of p53/TP53 phosphorylation thereby relieving the repressive effect of p53/TP53 on cyclin-D1 (CCND1) expression. Phosphorylates ACIN1, and [...] (699 aa)
**Q96SB4**	SRPK1	SRSF protein kinase 1 OS=Homo sapiens OX=9606 GN=SRPK1 PE=1 SV=2	Serine/arginine-rich protein-specific kinase which specifically phosphorylates its substrates at serine residues located in regions rich in arginine/serine dipeptides, known as RS domains and is involved in the phosphorylation of SR splicing factors and the regulation of splicing. Plays a central role in the regulatory network for splicing, controlling the intranuclear distribution of splicing factors in interphase cells and the reorganization of nuclear speckles during mitosis. Can influence additional steps of mRNA maturation, as well as other cellular activiti [...] (655 aa)
**Q99986**	VRK1	Serine/threonine-protein kinase VRK1 OS=Homo sapiens OX=9606 GN=VRK1 PE=1 SV=1	Serine/threonine kinase involved in Golgi disassembly during the cell cycle- following phosphorylation by PLK3 during mitosis, required to induce Golgi fragmentation. Acts by mediating phosphorylation of downstream target protein. Phosphorylates 'Thr- 18' of p53/TP53 and may thereby prevent the interaction between p53/TP53 and MDM2. Phosphorylates casein and histone H3. Phosphorylates BANF1- disrupts its ability to bind DNA, reduces its binding to LEM domain-containing proteins and causes its relocalization from the nucleus to the cytoplasm. Phosph [...] (396 aa)

**Q7L7X3**	TAOK1	Serine/threonine-protein kinase TAO1 OS=Homo sapiens OX=9606 GN=TAOK1 PE=1 SV=1	Serine/threonine-protein kinase involved in various processes such as p38/MAPK14 stress-activated MAPK cascade, DNA damage response and regulation of cytoskeleton stability. Phosphorylates MAP2K3, MAP2K6 and MARK2. Acts as an activator of the p38/MAPK14 stress-activated MAPK cascade by mediating phosphorylation and subsequent activation of the upstream MAP2K3 and MAP2K6 kinases. Involved in G-protein coupled receptor signaling to p38/MAPK14. In response to DNA damage, involved in the G2/M transition DNA damage checkpoint by activating the p38/MAPK1 [...] (1001 aa)
**Q9UBS4**	DJB11	DnaJ homolog subfamily B member 11 OS=Homo sapiens OX=9606 GN=DNAJB11 PE=1 SV=1	Serves as a co-chaperone for HSPA5. Binds directly to both unfolded proteins that are substrates for ERAD and nascent unfolded peptide chains, but dissociates from the HSPA5-unfolded protein complex before folding is completed. May help recruiting HSPA5 and other chaperones to the substrate. Stimulates HSPA5 ATPase activity
**Q12929**	EPS8	Epidermal growth factor receptor kinase substrate 8 OS=Homo sapiens OX=9606 GN=EPS8 PE=1 SV=1	Signaling adapter that controls various cellular protrusions by regulating actin cytoskeleton dynamics and architecture. Depending on its association with other signal transducers, can regulate different processes. Together with SOS1 and ABI1, forms a trimeric complex that participates in transduction of signals from Ras to Rac by activating the Rac- specific guanine nucleotide exchange factor (GEF) activity. Acts as a direct regulator of actin dynamics by binding actin filaments and has both barbed-end actin filament capping and act [...] (822 aa)
**O76094**	SRP72	Signal recognition particle subunit SRP72 OS=Homo sapiens OX=9606 GN=SRP72 PE=1 SV=3	Signal-recognition-particle assembly has a crucial role in targeting secretory proteins to the rough endoplasmic reticulum membrane. Binds the 7S RNA only in presence of SRP68. This ribonucleoprotein complex might interact directly with the docking protein in the ER membrane and possibly participate in the elongation arrest function
**P37108**	SRP14	Signal recognition particle 14 kDa protein OS=Homo sapiens OX=9606 GN=SRP14 PE=1 SV=2	Signal-recognition-particle assembly has a crucial role in targeting secretory proteins to the rough endoplasmic reticulum membrane. SRP9 together with SRP14 and the Alu portion of the SRP RNA, constitutes the elongation arrest domain of SRP. The complex of SRP9 and SRP14 is required for SRP RNA binding (136 aa)

**P29558**	RBMS1	RNA-binding motif, single-stranded-interacting protein 1 OS=Homo sapiens OX=9606 GN=RBMS1 PE=1 SV=3	Single-stranded DNA binding protein that interacts with the region upstream of the MYC gene. Binds specifically to the DNA sequence motif 5'-[AT]CT[AT][AT]T-3'. Probably has a role in DNA replication (406 aa)
**Q8TDG4**	HELQ	Helicase POLQ-like OS=Homo sapiens OX=9606 GN=HELQ PE=1 SV=2	Single-stranded DNA-dependent ATPase and 5' to 3' DNA helicase. Involved in the repair of DNA cross-links and double-strand break (DSB) resistance. Participates in FANCD2-mediated repair. Forms a complex with POLN polymerase that participates in homologous recombination (HR) repair and is essential for cellular protection against DNA cross-links
**P12956**	XRCC6	X-ray repair cross-complementing protein 6 OS=Homo sapiens OX=9606 GN=XRCC6 PE=1 SV=2	Single-stranded DNA-dependent ATP-dependent helicase. Has a role in chromosome translocation. The DNA helicase II complex binds preferentially to fork-like ends of double-stranded DNA in a cell cycle-dependent manner. It works in the 3'-5' direction. Binding to DNA may be mediated by XRCC6. Involved in DNA non-homologous end joining (NHEJ) required for double-strand break repair and V(D)J recombination. The XRCC5/6 dimer acts as regulatory subunit of the DNA-dependent protein kinase complex DNA-PK by increasing the affinity of the catalytic s [...] (609 aa)
**P13010**	XRCC5	X-ray repair cross-complementing protein 5 OS=Homo sapiens OX=9606 GN=XRCC5 PE=1 SV=3	Single-stranded DNA-dependent ATP-dependent helicase. Has a role in chromosome translocation. The DNA helicase II complex binds preferentially to fork-like ends of double-stranded DNA in a cell cycle-dependent manner. It works in the 3'-5' direction. Binding to DNA may be mediated by XRCC6. Involved in DNA non-homologous end joining (NHEJ) required for double-strand break repair and V(D)J recombination. The XRCC5/6 dimer acts as regulatory subunit of the DNA-dependent protein kinase complex DNA-PK by increasing the affinity of the catalytic s [...] (732 aa)
**P57721**	PCBP3	Poly(rC)-binding protein 3 OS=Homo sapiens OX=9606 GN=PCBP3 PE=2 SV=2	Single-stranded nucleic acid binding protein that binds preferentially to oligo dC (371 aa)
**Q15366**	PCBP2	Poly(rC)-binding protein 2 OS=Homo sapiens OX=9606 GN=PCBP2 PE=1 SV=1	Single-stranded nucleic acid binding protein that binds preferentially to oligo dC. Major cellular poly(rC)-binding protein. Binds also poly(rU). Negatively regulates cellular antiviral responses mediated by MAVS signaling. It acts as an adapter between MAVS and the E3 ubiquitin ligase ITCH, therefore triggering MAVS ubiquitination and degradation (366 aa)

**Q9H967**	WDR76	WD repeat-containing protein 76 OS=Homo sapiens OX=9606 GN=WDR76 PE=1 SV=2	Specifically binds 5-hydroxymethylcytosine (5hmC), suggesting that it acts as a specific reader of 5hmC
**Q9UNQ2**	DIM1	Probable dimethyladenosine transferase OS=Homo sapiens OX=9606 GN=DIMT1 PE=1 SV=1	Specifically dimethylates two adjacent adenosines in the loop of a conserved hairpin near the 3’-end of 18S rRNA in the 40S particle. Involved in the pre-rRNA processing steps leading to small-subunit rRNA production independently of its RNA-modifying catalytic activity
**Q9H6S0**	YTDC2	3'-5' RNA helicase YTHDC2 OS=Homo sapiens OX=9606 GN=YTHDC2 PE=1 SV=2	Specifically recognizes and binds N6-methyladenosine (m6A)-containing RNAs affecting the translation efficiency and mRNA abundance of its targets. Is required for proper spermatocyte development (By similarity). M6A is a modification present at internal sites of mRNAs and some non-coding RNAs and plays a role in the efficiency of mRNA splicing, processing and stability. When associated with MEIOC, binds transcripts that regulate the mitotic cell cycle inhibiting progression into metaphase, thereby allowing meiotic prophase to proceed normall [...] (1430 aa)
**Q7Z739**	YTHD3	YTH domain-containing family protein 3 OS=Homo sapiens OX=9606 GN=YTHDF3 PE=1 SV=1	Specifically recognizes and binds N6-methyladenosine (m6A)-containing RNAs and promotes RNA translation efficiency. M6A is a modification present at internal sites of mRNAs and some non- coding RNAs and plays a role in the efficiency of mRNA splicing, processing and stability. Shares m6A- containing mRNAs targets with YTHDF1 and YTHDF2, and regulates different processes depending on the context. Facilitates the translation of targeted mRNAs in cooperation with YTHDF1 by binding to m6A-containing mRNAs and interacting with 40S and 60S ribosome sub [...] (588 aa)
**Q9Y5A9**	YTHD2	YTH domain-containing family protein 2 OS=Homo sapiens OX=9606 GN=YTHDF2 PE=1 SV=2	Specifically recognizes and binds N6-methyladenosine (m6A)-containing RNAs, and regulates mRNA stability. M6A is a modification present at internal sites of mRNAs and some non-coding RNAs and plays a role in mRNA stability and processing. Acts as a regulator of mRNA stability-binding to m6A-containing mRNAs results in the localization to mRNA decay sites, such as processing bodies (P-bodies), leading to mRNA degradation. Required maternally to regulate oocyte maturation-probably acts by binding to m6A-containing mRNAs, thereby regulating mater [...] (579 aa)

**Q86XZ4**	SPAS2	Spermatogenesis-associated serine-rich protein 2 OS=Homo sapiens OX=9606 GN=SPATS2 PE=1 SV=1	Spermatogenesis associated serine rich 2 (545 aa); SPATS2 (Spermatogenesis Associated Serine Rich 2) is a Protein Coding gene. An important paralog of this gene is SPATS2L.
**Q9NUQ6**	SPS2L	SPATS2-like protein OS=Homo sapiens OX=9606 GN=SPATS2L PE=1 SV=2	Spermatogenesis associated serine rich 2 like; SPATS2L (Spermatogenesis Associated Serine Rich 2 Like) is a Protein Coding gene. An important paralog of this gene is SPATS2.
**Q9ULW0**	TPX2	Targeting protein for Xklp2 OS=Homo sapiens OX=9606 GN=TPX2 PE=1 SV=2	Spindle assembly factor required for normal assembly of mitotic spindles. Required for normal assembly of microtubules during apoptosis. Required for chromatin and/or kinetochore dependent microtubule nucleation. Mediates AURKA localization to spindle microtubules. Activates AURKA by promoting its autophosphorylation at 'Thr-288' and protects this residue against dephosphorylation. TPX2 is inactivated upon binding to importin- alpha. At the onset of mitosis, GOLGA2 interacts with importin-alpha, liberating TPX2 from importin-alpha, allowing TPX2 to activate [...] (747 aa)
**Q96I25**	SPF45	Splicing factor 45 OS=Homo sapiens OX=9606 GN=RBM17 PE=1 SV=1	Splice factor that binds to the single-stranded 3'AG at the exon/intron border and promotes its utilization in the second catalytic step. Involved in the regulation of alternative splicing and the utilization of cryptic splice sites. Promotes the utilization of a cryptic splice site created by the beta-110 mutation in the HBB gene. The resulting frameshift leads to sickle cell anemia
**P41223**	BUD31	Protein BUD31 homolog OS=Homo sapiens OX=9606 GN=BUD31 PE=1 SV=2	Involved in the pre-mRNA splicing process (PubMed:28502770, PubMed:28076346). May play a role as regulator of AR transcriptional activity; may increase AR transcriptional activity (PubMed:25091737).
**P14866**	HNRPL	Heterogeneous nuclear ribonucleoprotein L OS=Homo sapiens OX=9606 GN=HNRNPL PE=1 SV=2	Splicing factor binding to exonic or intronic sites and acting as either an activator or repressor of exon inclusion. Exhibits a binding preference for CA-rich elements. Component of the heterogeneous nuclear ribonucleoprotein (hnRNP) complexes and associated with most nascent transcripts. Associates, together with APEX1, to the negative calcium responsive element (nCaRE) B2 of the APEX2 promoter

**P84103**	SRSF3	Serine/arginine-rich splicing factor 3 OS=Homo sapiens OX=9606 GN=SRSF3 PE=1 SV=1	Splicing factor that specifically promotes exon-inclusion during alternative splicing. Interaction with YTHDC1, a RNA-binding protein that recognizes and binds N6-methyladenosine (m6A)-containing RNAs, promotes recruitment of SRSF3 to its mRNA-binding elements adjacent to m6A sites, leading to exon-inclusion during alternative splicing. Also functions as export adapter involved in mRNA nuclear export such as of histone H2A. Binds mRNA which is thought to be transferred to the NXF1-NXT1 heterodimer for export (TAP/NXF1 pathway)
**Q15056**	IF4H	Eukaryotic translation initiation factor 4H OS=Homo sapiens OX=9606 GN=EIF4H PE=1 SV=5	Stimulates the RNA helicase activity of EIF4A in the translation initiation complex. Binds weakly mRNA
**P39748**	FEN1	Flap endonuclease 1 OS=Homo sapiens OX=9606 GN=FEN1 PE=1 SV=1	Structure-specific nuclease with 5’-flap endonuclease and 5’-3’ exonuclease activities involved in DNA replication and repair. During DNA replication, cleaves the 5’-overhanging flap structure that is generated by displacement synthesis when DNA polymerase encounters the 5’-end of a downstream Okazaki fragment. It enters the flap from the 5’-end and then tracks to cleave the flap base, leaving a nick for ligation. Also involved in the long patch base excision repair (LP-BER) pathway, by cleaving within the apurinic/apyrimidinic (AP) site-terminated flap. Acts as a [...] (380 aa)
**Q8NEZ5**	FBX22	F-box only protein 22 OS=Homo sapiens OX=9606 GN=FBXO22 PE=1 SV=1	Substrate-recognition component of the SCF (SKP1-CUL1-F- box protein)-type E3 ubiquitin ligase complex. Promotes the proteasome-dependent degradation of key sarcomeric proteins, such as alpha-actinin (ACTN2) and filamin-C (FLNC), essential for maintenance of normal contractile function
**Q12874**	SF3A3	Splicing factor 3A subunit 3 OS=Homo sapiens OX=9606 GN=SF3A3 PE=1 SV=1	Subunit of the splicing factor SF3A required for ’A’ complex assembly formed by the stable binding of U2 snRNP to the branchpoint sequence (BPS) in pre-mRNA. Sequence independent binding of SF3A/SF3B complex upstream of the branch site is essential, it may anchor U2 snRNP to the pre-mRNA. May also be involved in the assembly of the ’E’ complex
**Q15428**	SF3A2	Splicing factor 3A subunit 2 OS=Homo sapiens OX=9606 GN=SF3A2 PE=1 SV=2	Subunit of the splicing factor SF3A required for ’A’ complex assembly formed by the stable binding of U2 snRNP to the branchpoint sequence (BPS) in pre-mRNA. Sequence independent binding of SF3A/SF3B complex upstream of the branch site is essential, it may anchor U2 snRNP to the pre-mRNA. May also be involved in the assembly of the ’E’ complex

**Q15459**	SF3A1	Splicing factor 3A subunit 1 OS=Homo sapiens OX=9606 GN=SF3A1 PE=1 SV=1	Subunit of the splicing factor SF3A required for 'A' complex assembly formed by the stable binding of U2 snRNP to the branchpoint sequence (BPS) in pre-mRNA. Sequence independent binding of SF3A/SF3B complex upstream of the branch site is essential, it may anchor U2 snRNP to the pre-mRNA. May also be involved in the assembly of the 'E' complex
**Q99748**	NRTN	Neurturin OS=Homo sapiens OX=9606 GN=NRTN PE=1 SV=1	Supports the survival of sympathetic neurons in culture. May regulate the development and maintenance of the CNS. Might control the size of non-neuronal cell population such as haemopoietic cells
**O75937**	DNJC8	DnaJ homolog subfamily C member 8 OS=Homo sapiens OX=9606 GN=DNAJC8 PE=1 SV=2	Suppresses polyglutamine (polyQ) aggregation of ATXN3 in neuronal cells
**Q01658**	NC2B	Protein Dr1 OS=Homo sapiens OX=9606 GN=DR1 PE=1 SV=1	The association of the DR1/DRAP1 heterodimer with TBP results in a functional repression of both activated and basal transcription of class II genes. This interaction precludes the formation of a transcription-competent complex by inhibiting the association of TFIIA and/or TFIIB with TBP. Can bind to DNA on its own. Component of the ATAC complex, a complex with histone acetyltransferase activity on histones H3 and H4 (176 aa)
**Q14919**	NC2A	Dr1-associated corepressor OS=Homo sapiens OX=9606 GN=DRAP1 PE=1 SV=3	The association of the DR1/DRAP1 heterodimer with TBP results in a functional repression of both activated and basal transcription of class II genes. This interaction precludes the formation of a transcription-competent complex by inhibiting the association of TFIIA and/or TFIIB with TBP. Can bind to DNA on its own (205 aa)
**P11182**	ODB2	Lipoamide acyltransferase component of branched-chain alpha-keto acid dehydrogenase complex, mitochondrial OS=Homo sapiens OX=9606 GN=DBT PE=1 SV=3	The branched-chain alpha-keto dehydrogenase complex catalyzes the overall conversion of alpha-keto acids to acyl-CoA and CO(2). It contains multiple copies of three enzymatic components- branched-chain alpha-keto acid decarboxylase (E1), lipoamide acyltransferase (E2) and lipoamide dehydrogenase (E3). Within this complex, the catalytic function of this enzyme is to accept, and to transfer to coenzyme A, acyl groups that are generated by the branched-chain alpha-keto acid decarboxylase component (482 aa)

**P53621**	COPA	Coatomer subunit alpha OS=Homo sapiens OX=9606 GN=COPA PE=1 SV=2	The coatomer is a cytosolic protein complex that binds to dilysine motifs and reversibly associates with Golgi non- clathrin-coated vesicles, which further mediate biosynthetic protein transport from the ER, via the Golgi up to the trans Golgi network. Coatomer complex is required for budding from Golgi membranes, and is essential for the retrograde Golgi-to-ER transport of dilysine-tagged proteins. In mammals, the coatomer can only be recruited by membranes associated to ADP-ribosylation factors (ARFs), which are small GTP-binding proteins
**P35249**	RFC4	Replication factor C subunit 4 OS=Homo sapiens OX=9606 GN=RFC4 PE=1 SV=2	The elongation of primed DNA templates by DNA polymerase delta and epsilon requires the action of the accessory proteins proliferating cell nuclear antigen (PCNA) and activator 1. This subunit may be involved in the elongation of the multiprimed DNA template (363 aa)
**P50579**	MAP2	Methionine aminopeptidase 2 OS=Homo sapiens OX=9606 GN=METAP2 PE=1 SV=1	The exact function of MAP2 is unknown but MAPs may stabilize the microtubules against depolymerization. They also seem to have a stiffening effect on microtubules
**P39023**	RL3	60S ribosomal protein L3 OS=Homo sapiens OX=9606 GN=RPL3 PE=1 SV=2	The L3 protein is a component of the large subunit of cytoplasmic ribosomes
**P02533**	K1C14	Keratin, type I cytoskeletal 14 OS=Homo sapiens OX=9606 GN=KRT14 PE=1 SV=4	The nonhelical tail domain is involved in promoting KRT5-KRT14 filaments to self-organize into large bundles and enhances the mechanical properties involved in resilience of keratin intermediate filaments in vitro (472 aa)
**Q8TAX9**	GSDMB	Gasdermin-B OS=Homo sapiens OX=9606 GN=GSDMB PE=1 SV=2	The N-terminal moiety promotes pyroptosis. May be acting by homooligomerizing within the membrane and forming pores. The physiological relevance of this observation is unknown (Probable)
**Q9UHI6**	DDX20	Probable ATP-dependent RNA helicase DDX20 OS=Homo sapiens OX=9606 GN=DDX20 PE=1 SV=2	The SMN complex plays a catalyst role in the assembly of small nuclear ribonucleoproteins (snRNPs), the building blocks of the spliceosome. Thereby, plays an important role in the splicing of cellular pre-mRNAs. Most spliceosomal snRNPs contain a common set of Sm proteins SNRPB, SNRPD1, SNRPD2, SNRPD3, SNRPE, SNRPF and SNRPG that assemble in a heptameric protein ring on the Sm site of the small nuclear RNA to form the core snRNP. In the cytosol, the Sm proteins SNRPD1, SNRPD2, SNRPE, SNRPF and SNRPG are trapped in an inactive 6S pICln-Sm compl [...] (824 aa)

**Q9Y3F4**	STRAP	Serine-threonine kinase receptor-associated protein OS=Homo sapiens OX=9606 GN=STRAP PE=1 SV=1	The SMN complex plays a catalyst role in the assembly of small nuclear ribonucleoproteins (snRNPs), the building blocks of the spliceosome. Thereby, plays an important role in the splicing of cellular pre-mRNAs. Most spliceosomal snRNPs contain a common set of Sm proteins SNRPB, SNRPD1, SNRPD2, SNRPD3, SNRPE, SNRPF and SNRPG that assemble in a heptameric protein ring on the Sm site of the small nuclear RNA to form the core snRNP. In the cytosol, the Sm proteins SNRPD1, SNRPD2, SNRPE, SNRPF and SNRPG are trapped in an inactive 6S pICl [...] (350 aa)
**Q8TAT6**	NPL4	Nuclear protein localization protein 4 homolog OS=Homo sapiens OX=9606 GN=NPLOC4 PE=1 SV=3	The ternary complex containing UFD1, VCP and NPLOC4 binds ubiquitinated proteins and is necessary for the export of misfolded proteins from the ER to the cytoplasm, where they are degraded by the proteasome. The NPLOC4-UFD1-VCP complex regulates spindle disassembly at the end of mitosis and is necessary for the formation of a closed nuclear envelope (By similarity). Acts as a negative regulator of type I interferon production via the complex formed with VCP and UFD1, which binds to DDX58/RIG-I and recruits RNF125 to promote ubiquitination [...] (608 aa)
**P51911**	CNN1	Calponin-1 OS=Homo sapiens OX=9606 GN=CNN1 PE=1 SV=2	Thin filament-associated protein that is implicated in the regulation and modulation of smooth muscle contraction. It is capable of binding to actin, calmodulin, troponin C and tropomyosin. The interaction of calponin with actin inhibits the actomyosin Mg-ATPase activity (By similarity) (297 aa)
**P30041**	PRDX6	Peroxiredoxin-6 OS=Homo sapiens OX=9606 GN=PRDX6 PE=1 SV=3	Thiol-specific peroxidase that catalyzes the reduction of hydrogen peroxide and organic hydroperoxides to water and alcohols, respectively. Can reduce H(2)O(2) and short chain organic, fatty acid, and phospholipid hydroperoxides. Also has phospholipase activity, and can therefore either reduce the oxidized sn-2 fatty acyl grup of phospholipids (peroxidase activity) or hydrolyze the sn-2 ester bond of phospholipids (phospholipase activity). These activities are dependent on binding to phospholipids at acidic pH and to oxidized phospholipds at cytosolic pH. Plays a role [...] (224 aa)

**P32119**	PRDX2	Peroxiredoxin-2 OS=Homo sapiens OX=9606 GN=PRDX2 PE=1 SV=5	Thiol-specific peroxidase that catalyzes the reduction of hydrogen peroxide and organic hydroperoxides to water and alcohols, respectively. Plays a role in cell protection against oxidative stress by detoxifying peroxides and as sensor of hydrogen peroxide-mediated signaling events. Might participate in the signaling cascades of growth factors and tumor necrosis factor-alpha by regulating the intracellular concentrations of H(2)O(2)
**Q06830**	PRDX1	Peroxiredoxin-1 OS=Homo sapiens OX=9606 GN=PRDX1 PE=1 SV=1	Thiol-specific peroxidase that catalyzes the reduction of hydrogen peroxide and organic hydroperoxides to water and alcohols, respectively. Plays a role in cell protection against oxidative stress by detoxifying peroxides and as sensor of hydrogen peroxide-mediated signaling events. Might participate in the signaling cascades of growth factors and tumor necrosis factor-alpha by regulating the intracellular concentrations of H(2)O(2). Reduces an intramolecular disulfide bond in GDPD5 that gates the ability to GDPD5 to drive postmitotic motor neuron differentiation (By s [...] (199 aa)
**P62750**	RL23A	60S ribosomal protein L23a OS=Homo sapiens OX=9606 GN=RPL23A PE=1 SV=1	Component of the ribosome, a large ribonucleoprotein complex responsible for the synthesis of proteins in the cell. Binds a specific region on the 26S rRNA. May promote p53/TP53 degradation possibly through the stimulation of MDM2-mediated TP53 polyubiquitination (PubMed:26203195).
**P55795**	HNRH2	Heterogeneous nuclear ribonucleoprotein H2 OS=Homo sapiens OX=9606 GN=HNRNPH2 PE=1 SV=1	This protein is a component of the heterogeneous nuclear ribonucleoprotein (hnRNP) complexes which provide the substrate for the processing events that pre-mRNAs undergo before becoming functional, translatable mRNAs in the cytoplasm. Binds poly(RG)
**P31943**	HNRH1	Heterogeneous nuclear ribonucleoprotein H OS=Homo sapiens OX=9606 GN=HNRNPH1 PE=1 SV=4	This protein is a component of the heterogeneous nuclear ribonucleoprotein (hnRNP) complexes which provide the substrate for the processing events that pre-mRNAs undergo before becoming functional, translatable mRNAs in the cytoplasm. Mediates pre-mRNA alternative splicing regulation. Inhibits, together with CUGBP1, insulin receptor (IR) pre-mRNA exon 11 inclusion in myoblast. Binds to the IR RNA. Binds poly(RG)
**P09661**	RU2A	U2 small nuclear ribonucleoprotein A' OS=Homo sapiens OX=9606 GN=SNRPA1 PE=1 SV=2	This protein is associated with sn-RNP U2. It helps the A’ protein to bind stem loop IV of U2 snRNA
**P49411**	EFTU	Elongation factor Tu, mitochondrial OS=Homo sapiens OX=9606 GN=TUFM PE=1 SV=2	This protein promotes the GTP-dependent binding of aminoacyl-tRNA to the A-site of ribosomes during protein biosynthesis

**Q05639**	EF1A2	Elongation factor 1-alpha 2 OS=Homo sapiens OX=9606 GN=EEF1A2 PE=1 SV=1	This protein promotes the GTP-dependent binding of aminoacyl-tRNA to the A-site of ribosomes during protein biosynthesis
**P68104**	EF1A1	Elongation factor 1-alpha 1 OS=Homo sapiens OX=9606 GN=EEF1A1 PE=1 SV=1	This protein promotes the GTP-dependent binding of aminoacyl-tRNA to the A-site of ribosomes during protein biosynthesis. With PARP1 and TXK, forms a complex that acts as a T helper 1 (Th1) cell-specific transcription factor and binds the promoter of IFN-gamma to directly regulate its transcription, and is thus involved importantly in Th1 cytokine production
**Q9Y210**	TRPC6	Short transient receptor potential channel 6 OS=Homo sapiens OX=9606 GN=TRPC6 PE=1 SV=1	Thought to form a receptor-activated non-selective calcium permeant cation channel. Probably is operated by a phosphatidylinositol second messenger system activated by receptor tyrosine kinases or G-protein coupled receptors. Activated by diacylglycerol (DAG) in a membrane-delimited fashion, independently of protein kinase C. Seems not to be activated by intracellular calcium store depletion
**P05787**	K2C8	Keratin, type II cytoskeletal 8 OS=Homo sapiens OX=9606 GN=KRT8 PE=1 SV=7	Together with KRT19, helps to link the contractile apparatus to dystrophin at the costameres of striated muscle
**Q5VWG9**	TAF3	Transcription initiation factor TFIID subunit 3 OS=Homo sapiens OX=9606 GN=TAF3 PE=1 SV=1	Transcription factor TFIID is one of the general factors required for accurate and regulated initiation by RNA polymerase II. TFIID is a multimeric protein complex that plays a central role in mediating promoter responses to various activators and repressors. Required in complex with TBPL2 for the differentiation of myoblasts into myocytes. The complex replaces TFIID at specific promoters at an early stage in the differentiation process
**P08047**	SP1	Transcription factor Sp1 OS=Homo sapiens OX=9606 GN=SP1 PE=1 SV=3	Transcription factor that can activate or repress transcription in response to physiological and pathological stimuli. Binds with high affinity to GC-rich motifs and regulates the expression of a large number of genes involved in a variety of processes such as cell growth, apoptosis, differentiation and immune responses. Highly regulated by post-translational modifications (phosphorylations, sumoylation, proteolytic cleavage, glycosylation and acetylation). Binds also the PDGFR- alpha G-box promoter. May have a role in modulating the cellular response to DNA d [...] (785 aa)
**Q9Y6H1**	CHCH2	Coiled-coil-helix-coiled-coil-helix domain-containing protein 2 OS=Homo sapiens OX=9606 GN=CHCHD2 PE=1 SV=1	Transcription factor. Binds to the oxygen responsive element of COX4I2 and activates its transcription under hypoxia conditions (4% oxygen), as well as normoxia conditions (20% oxygen)

**Q9H9S0**	NANOG	Homeobox protein NANOG OS=Homo sapiens OX=9606 GN=NANOG PE=1 SV=2	Transcription regulator involved in inner cell mass and embryonic stem (ES) cells proliferation and self-renewal. Imposes pluripotency on ES cells and prevents their differentiation towards extraembryonic endoderm and trophectoderm lineages. Blocks bone morphogenetic protein-induced mesoderm differentiation of ES cells by physically interacting with SMAD1 and interfering with the recruitment of coactivators to the active SMAD transcriptional complexes. Acts as a transcriptional activator or repressor. Binds optimally to the DNA consensus sequence 5'-TAAT[GT][GT] [...] (305 aa)
**Q14498**	RBM39	RNA-binding protein 39 OS=Homo sapiens OX=9606 GN=RBM39 PE=1 SV=2	Transcriptional coactivator for steroid nuclear receptors ESR1/ER-alpha and ESR2/ER-beta, and JUN/AP-1 (By similarity). May be involved in pre-mRNA splicing process
**O75475**	PSIP1	PC4 and SFRS1-interacting protein OS=Homo sapiens OX=9606 GN=PSIP1 PE=1 SV=1	Transcriptional coactivator involved in neuroepithelial stem cell differentiation and neurogenesis. Involved in particular in lens epithelial cell gene regulation and stress responses. May play an important role in lens epithelial to fiber cell terminal differentiation. May play a protective role during stress-induced apoptosis. Isoform 2 is a more general and stronger transcriptional coactivator. Isoform 2 may also act as an adapter to coordinate pre-mRNA splicing. Cellular cofactor for lentiviral integration
**O60869**	EDF1	Endothelial differentiation-related factor 1 OS=Homo sapiens OX=9606 GN=EDF1 PE=1 SV=1	Transcriptional coactivator stimulating NR5A1 and ligand-dependent NR1H3/LXRA and PPARG transcriptional activities. Enhances the DNA-binding activity of ATF1, ATF2, CREB1 and NR5A1. Regulates nitric oxid synthase activity probably by sequestering calmodulin in the cytoplasm. May function in endothelial cells differentiation, hormone-induced cardiomyocytes hypertrophy and lipid metabolism
**P54259**	ATN1	Atrophin-1 OS=Homo sapiens OX=9606 GN=ATN1 PE=1 SV=3	Transcriptional corepressor. Recruits NR2E1 to repress transcription. Promotes vascular smooth cell (VSMC) migration and orientation (By similarity). Corepressor of MTG8 transcriptional repression. Has some intrinsic repression activity which is independent of the number of poly-Asn (polyQ) repeats (1190 aa)
**Q8IWS0**	PHF6	PHD finger protein 6 OS=Homo sapiens OX=9606 GN=PHF6 PE=1 SV=1	Transcriptional regulator that associates with ribosomal RNA promoters and suppresses ribosomal RNA (rRNA) transcription

**Q9UIS9**	MBD1	Methyl-CpG-binding domain protein 1 OS=Homo sapiens OX=9606 GN=MBD1 PE=1 SV=2	Transcriptional repressor that binds CpG islands in promoters where the DNA is methylated at position 5 of cytosine within CpG dinucleotides. Binding is abolished by the presence of 7-mG that is produced by DNA damage by methylmethanesulfonate (MMS). Acts as transcriptional repressor and plays a role in gene silencing by recruiting AFT7IP, which in turn recruits factors such as the histone methyltransferase SETDB1. Probably forms a complex with SETDB1 and ATF7IP that represses transcription and couples DNA methylation and histone 'Lys-9' trimethylat [...] (655 aa)
**P09493**	TPM1	Tropomyosin alpha-1 chain OS=Homo sapiens OX=9606 GN=TPM1 PE=1 SV=2	Binds to actin filaments in muscle and non-muscle cells (PubMed:23170982). Plays a central role, in association with the troponin complex, in the calcium dependent regulation of vertebrate striated muscle contraction (PubMed:23170982). Smooth muscle contraction is regulated by interaction with caldesmon. In non-muscle cells is implicated in stabilizing cytoskeleton actin filaments.
**P67936**	TPM4	Tropomyosin alpha-4 chain OS=Homo sapiens OX=9606 GN=TPM4 PE=1 SV=3	Binds to actin filaments in muscle and non-muscle cells. Plays a central role, in association with the troponin complex, in the calcium dependent regulation of vertebrate striated muscle contraction. Smooth muscle contraction is regulated by interaction with caldesmon. In non-muscle cells is implicated in stabilizing cytoskeleton actin filaments (By similarity). Binds calcium (PubMed:1836432).
**P07437**	TBB5	Tubulin beta chain OS=Homo sapiens OX=9606 GN=TUBB PE=1 SV=2	Tubulin is the major constituent of microtubules. It binds two moles of GTP, one at an exchangeable site on the beta chain and one at a non-exchangeable site on the alpha chain
**P68363**	TBA1B	Tubulin alpha-1B chain OS=Homo sapiens OX=9606 GN=TUBA1B PE=1 SV=1	Tubulin is the major constituent of microtubules. It binds two moles of GTP, one at an exchangeable site on the beta chain and one at a non-exchangeable site on the alpha chain
**P68371**	TBB4B	Tubulin beta-4B chain OS=Homo sapiens OX=9606 GN=TUBB4B PE=1 SV=1	Tubulin is the major constituent of microtubules. It binds two moles of GTP, one at an exchangeable site on the beta chain and one at a non-exchangeable site on the alpha chain
**Q9BQE3**	TBA1C	Tubulin alpha-1C chain OS=Homo sapiens OX=9606 GN=TUBA1C PE=1 SV=1	Tubulin is the major constituent of microtubules. It binds two moles of GTP, one at an exchangeable site on the beta chain and one at a non-exchangeable site on the alpha chain

**Q9H4B7**	TBB1	Tubulin beta-1 chain OS=Homo sapiens OX=9606 GN=TUBB1 PE=1 SV=1	Tubulin is the major constituent of microtubules. It binds two moles of GTP, one at an exchangeable site on the beta chain and one at a non-exchangeable site on the alpha chain (By similarity)
**Q9BVA1**	TBB2B	Tubulin beta-2B chain OS=Homo sapiens OX=9606 GN=TUBB2B PE=1 SV=1	Tubulin is the major constituent of microtubules. It binds two moles of GTP, one at an exchangeable site on the beta chain and one at a non-exchangeable site on the alpha chain (By similarity). Plays a critical role in proper axon guidance in both central and peripheral axon tracts. Implicated in neuronal migration
**Q04695**	K1C17	Keratin, type I cytoskeletal 17 OS=Homo sapiens OX=9606 GN=KRT17 PE=1 SV=2	Type I keratin involved in the formation and maintenance of various skin appendages, specifically in determining shape and orientation of hair (By similarity). Required for the correct growth of hair follicles, in particular for the persistence of the anagen (growth) state (By similarity). Modulates the function of TNF-alpha in the specific context of hair cycling. Regulates protein synthesis and epithelial cell growth through binding to the adapter protein SFN and by stimulating Akt/mTOR pathway (By similarity). Involved in tissue repair. May be a mark [...] (432 aa)
**O15042**	SR140	U2 snRNP-associated SURP motif-containing protein OS=Homo sapiens OX=9606 GN=U2SURP PE=1 SV=2	U2 snRNP associated SURP domain containing; U2SURP (U2 SnRNP Associated SURP Domain Containing) is a Protein Coding gene. Among its related pathways are mRNA Splicing - Major Pathway and Gene Expression. Gene Ontology (GO) annotations related to this gene include nucleic acid binding and RNA binding.
**P62979**	RS27A	Ubiquitin-40S ribosomal protein S27a OS=Homo sapiens OX=9606 GN=RPS27A PE=1 SV=2	Ubiquitin- Exists either covalently attached to another protein, or free (unanchored). When covalently bound, it is conjugated to target proteins via an isopeptide bond either as a monomer (monoubiquitin), a polymer linked via different Lys residues of the ubiquitin (polyubiquitin chains) or a linear polymer linked via the initiator Met of the ubiquitin (linear polyubiquitin chains). Polyubiquitin chains, when attached to a target protein, have different functions depending on the Lys residue of the ubiquitin that is linked- Lys-6-linked may be inv [...] (156 aa)

**P62987**	RL40	Ubiquitin-60S ribosomal protein L40 OS=Homo sapiens OX=9606 GN=UBA52 PE=1 SV=2	Ubiquitin- Exists either covalently attached to another protein, or free (unanchored). When covalently bound, it is conjugated to target proteins via an isopeptide bond either as a monomer (monoubiquitin), a polymer linked via different Lys residues of the ubiquitin (polyubiquitin chains) or a linear polymer linked via the initiator Met of the ubiquitin (linear polyubiquitin chains). Polyubiquitin chains, when attached to a target protein, have different functions depending on the Lys residue of the ubiquitin that is linked- Lys-6-linked may be invo [...] (128 aa)
**P68036**	UB2L3	Ubiquitin-conjugating enzyme E2 L3 OS=Homo sapiens OX=9606 GN=UBE2L3 PE=1 SV=1	Ubiquitin-conjugating enzyme E2 that specifically acts with HECT-type and RBR family E3 ubiquitin-protein ligases. Does not function with most RING-containing E3 ubiquitin-protein ligases because it lacks intrinsic E3-independent reactivity with lysine- in contrast, it has activity with the RBR family E3 enzymes, such as PRKN and ARIH1, that function like function like RING-HECT hybrids. Accepts ubiquitin from the E1 complex and catalyzes its covalent attachment to other proteins. In vitro catalyzes 'Lys-11'-linked polyubiquitination. Involved in the [...] (212 aa)
**Q9GZQ8**	MLP3B	Microtubule-associated proteins 1A/1B light chain 3B OS=Homo sapiens OX=9606 GN=MAP1LC3B PE=1 SV=3	Ubiquitin-like modifier involved in formation of autophagosomal vacuoles (autophagosomes). Plays a role in mitophagy which contributes to regulate mitochondrial quantity and quality by eliminating the mitochondria to a basal level to fulfill cellular energy requirements and preventing excess ROS production. Whereas LC3s are involved in elongation of the phagophore membrane, the GABARAP/GATE-16 subfamily is essential for a later stage in autophagosome maturation. Promotes primary ciliogenesis by removing OFD1 from centriolar satellit [...] (125 aa)

**P61956**	SUMO2	Small ubiquitin-related modifier 2 OS=Homo sapiens OX=9606 GN=SUMO2 PE=1 SV=3	Ubiquitin-like protein that can be covalently attached to proteins as a monomer or as a lysine-linked polymer. Covalent attachment via an isopeptide bond to its substrates requires prior activation by the E1 complex SAE1-SAE2 and linkage to the E2 enzyme UBE2I, and can be promoted by an E3 ligase such as PIAS1-4, RANBP2, CBX4 or ZNF451. This post-translational modification on lysine residues of proteins plays a crucial role in a number of cellular processes such as nuclear transport, DNA replication and repair, mitosis and signal transduction. Polyme [...] (95 aa)
**Q9UMS4**	PRP19	Pre-mRNA-processing factor 19 OS=Homo sapiens OX=9606 GN=PRPF19 PE=1 SV=1	Ubiquitin-protein ligase which is a core component of several complexes mainly involved pre-mRNA splicing and DNA repair. Core component of the PRP19C/Prp19 complex/NTC/Nineteen complex which is part of the spliceosome and participates in its assembly, its remodeling and is required for its activity. During assembly of the spliceosome, mediates 'Lys-63'-linked polyubiquitination of the U4 spliceosomal protein PRPF3. Ubiquitination of PRPF3 allows its recognition by the U5 component PRPF8 and stabilizes the U4/U5/U6 tri-snRNP spliceosomal complex. Recruite [...] (504 aa)
**O75367**	H2AY	Core histone macro-H2A.1 OS=Homo sapiens OX=9606 GN=H2AFY PE=1 SV=4	Variant histone H2A which replaces conventional H2A in a subset of nucleosomes where it represses transcription. Nucleosomes wrap and compact DNA into chromatin, limiting DNA accessibility to the cellular machineries which require DNA as a template. Histones thereby play a central role in transcription regulation, DNA repair, DNA replication and chromosomal stability. DNA accessibility is regulated via a complex set of post-translational modifications of histones, also called histone code, and nucleosome remodeling. Involved in stable X chromosome inactivation [...] (372 aa)

**Q9P0M6**	H2AW	Core histone macro-H2A.2 OS=Homo sapiens OX=9606 GN=H2AFY2 PE=1 SV=3	Variant histone H2A which replaces conventional H2A in a subset of nucleosomes where it represses transcription. Nucleosomes wrap and compact DNA into chromatin, limiting DNA accessibility to the cellular machineries which require DNA as a template. Histones thereby play a central role in transcription regulation, DNA repair, DNA replication and chromosomal stability. DNA accessibility is regulated via a complex set of post- translational modifications of histones, also called histone code, and nucleosome remodeling. May be involved in stable X chromosome inac [...] (372 aa)
**P16104**	H2AX	Histone H2AX OS=Homo sapiens OX=9606 GN=H2AFX PE=1 SV=2	Variant histone H2A which replaces conventional H2A in a subset of nucleosomes. Nucleosomes wrap and compact DNA into chromatin, limiting DNA accessibility to the cellular machineries which require DNA as a template. Histones thereby play a central role in transcription regulation, DNA repair, DNA replication and chromosomal stability. DNA accessibility is regulated via a complex set of post-translational modifications of histones, also called histone code, and nucleosome remodeling. Required for checkpoint-mediated arrest of cell cycle progression in response to low dose [...] (143 aa)
**Q71UI9**	H2AV	Histone H2A.V OS=Homo sapiens OX=9606 GN=H2AFV PE=1 SV=3	Variant histone H2A which replaces conventional H2A in a subset of nucleosomes. Nucleosomes wrap and compact DNA into chromatin, limiting DNA accessibility to the cellular machineries which require DNA as a template. Histones thereby play a central role in transcription regulation, DNA repair, DNA replication and chromosomal stability. DNA accessibility is regulated via a complex set of post-translational modifications of histones, also called histone code, and nucleosome remodeling. May be involved in the formation of constitutive heterochromatin. May be required for ch [...] (128 aa)

**P84243**	H33	Histone H3.3 OS=Homo sapiens OX=9606 GN=H3F3A PE=1 SV=2	Variant histone H3 which replaces conventional H3 in a wide range of nucleosomes in active genes. Constitutes the predominant form of histone H3 in non-dividing cells and is incorporated into chromatin independently of DNA synthesis. Deposited at sites of nucleosomal displacement throughout transcribed genes, suggesting that it represents an epigenetic imprint of transcriptionally active chromatin. Nucleosomes wrap and compact DNA into chromatin, limiting DNA accessibility to the cellular machineries which require DNA as a template. Histones thereby play a central role in [...] (136 aa)
**P08670**	VIME	Vimentin OS=Homo sapiens OX=9606 GN=VIM PE=1 SV=4	Vimentins are class-III intermediate filaments found in various non-epithelial cells, especially mesenchymal cells. Vimentin is attached to the nucleus, endoplasmic reticulum, and mitochondria, either laterally or terminally (466 aa)
**O60504**	VINEX	Vinexin OS=Homo sapiens OX=9606 GN=SORBS3 PE=1 SV=2	Vinexin alpha isoform promotes up-regulation of actin stress fiber formation. Vinexin beta isoform plays a role in cell spreading and enhances the activation of JNK/SAPK in response to EGF stimulation by using its third SH3 domain (671 aa)
**P20290**	BTF3	Transcription factor BTF3 OS=Homo sapiens OX=9606 GN=BTF3 PE=1 SV=1	When associated with NACA, prevents inappropriate targeting of non-secretory polypeptides to the endoplasmic reticulum (ER). Binds to nascent polypeptide chains as they emerge from the ribosome and blocks their interaction with the signal recognition particle (SRP), which normally targets nascent secretory peptides to the ER. BTF3 is also a general transcription factor that can form a stable complex with RNA polymerase II. Required for the initiation of transcription (206 aa)

**Q9NX58**	LYAR	Cell growth-regulating nucleolar protein OS=Homo sapiens OX=9606 GN=LYAR PE=1 SV=2	Zinc fingers C2HC-type (379 aa); Plays a role in the maintenance of the appropriate processing of 47S/45S pre-rRNA to 32S/30S pre-rRNAs and their subsequent processing to produce 18S and 28S rRNAs (PubMed:24495227). Also acts at the level of transcription regulation. Along with PRMT5, binds the gamma-globin (HBG1/HBG2) promoter and represses its expression (PubMed:25092918). In neuroblastoma cells, may also repress the expression of oxidative stress genes, including CHAC1, HMOX1, SLC7A11, ULBP1 and SNORD41 that encodes a small nucleolar RNA (PubMed:28686580). Preferentially binds to a DNA motif containing 5'-GGTTAT-3' (PubMed:25092918). Stimulates phagocytosis of photoreceptor outer segments by retinal pigment epithelial cells (By similarity). Prevents nucleolin/NCL self-cleavage, maintaining a normal steady-state level of NCL protein in undifferentiated embryonic stem cells (ESCs), which in turn is essential for ESC self-renewal
**Q5T1V6**	DDX59	Probable ATP-dependent RNA helicase DDX59 OS=Homo sapiens OX=9606 GN=DDX59 PE=1 SV=1	Zinc fingers HIT-type; DDX59 (DEAD-Box Helicase 59) is a Protein Coding gene. Diseases associated with DDX59 include Orofaciodigital Syndrome V and Orofaciodigital Syndrome. Gene Ontology (GO) annotations related to this gene include nucleic acid binding and helicase activity.
**P68133**	ACTS	Actin, alpha skeletal muscle OS=Homo sapiens OX=9606 GN=ACTA1 PE=1 SV=1	Actins are highly conserved proteins that are involved in various types of cell motility and are ubiquitously expressed in all eukaryotic cells.

**P25705**	ATPA	ATP synthase subunit alpha, mitochondrial OS=Homo sapiens OX=9606 GN=ATP5F1A PE=1 SV=1	Mitochondrial membrane ATP synthase (F(1)F(0) ATP synthase or Complex V) produces ATP from ADP in the presence of a proton gradient across the membrane which is generated by electron transport complexes of the respiratory chain. F-type ATPases consist of two structural domains, F(1) - containing the extramembraneous catalytic core, and F(0) - containing the membrane proton channel, linked together by a central stalk and a peripheral stalk. During catalysis, ATP synthesis in the catalytic domain of F(1) is coupled via a rotary mechanism of the central stalk subunits to proton translocation. Subunits alpha and beta form the catalytic core in F(1). Rotation of the central stalk against the surrounding alpha(3)beta(3) subunits leads to hydrolysis of ATP in three separate catalytic sites on the beta subunits. Subunit alpha does not bear the catalytic high-affinity ATP-binding sites
**A6NMY6**	AXA2L	Putative annexin A2-like protein OS=Homo sapiens OX=9606 GN=ANXA2P2 PE=5 SV=2	Calcium-regulated membrane-binding protein whose affinity for calcium is greatly enhanced by anionic phospholipids. It binds two calcium ions with high affinity. May be involved in heat-stress response.
**P0CW27**	CC166	Coiled-coil domain-containing protein 166 OS=Homo sapiens OX=9606 GN=CCDC166 PE=4 SV=1	CCDC166 (Coiled-Coil Domain Containing 166) is a Protein Coding gene. An important paralog of this gene is BBOF1.
**Q9UHI8**	ATS1	A disintegrin and metalloproteinase with thrombospondin motifs 1 OS=Homo sapiens OX=9606 GN=ADAMTS1 PE=1 SV=4	Cleaves aggrecan, a cartilage proteoglycan, at the '1938-Glu-|-Leu-1939' site (within the chondroitin sulfate attachment domain), and may be involved in its turnover (By similarity). Has angiogenic inhibitor activity. Active metalloprotease, which may be associated with various inflammatory processes as well as development of cancer cachexia. May play a critical role in follicular rupture.

**P60896**	SEM1	26S proteasome complex subunit SEM1 OS=Homo sapiens OX=9606 GN=SEM1 PE=1 SV=1	Component of the 26S proteasome, a multiprotein complex involved in the ATP-dependent degradation of ubiquitinated proteins. This complex plays a key role in the maintenance of protein homeostasis by removing misfolded or damaged proteins, which could impair cellular functions, and by removing proteins whose functions are no longer required. Therefore, the proteasome participates in numerous cellular processes, including cell cycle progression, apoptosis, or DNA damage repair (PubMed:15117943). Component of the TREX-2 complex (transcription and export complex 2), composed of at least ENY2, GANP, PCID2, SEM1, and either centrin CETN2 or CETN3 (PubMed:22307388). The TREX-2 complex functions in docking export-competent ribonucleoprotein particles (mRNPs) to the nuclear entrance of the nuclear pore complex (nuclear basket). TREX-2 participates in mRNA export and accurate chromatin positioning in the nucleus by tethering genes to the nuclear periphery. Binds and stabilizes BRCA2 and is thus involved in the control of R-loop-associated DNA damage and thus transcription-associated genomic instability. R-loop accumulation increases in SEM1-depleted cells.
**Q9UJX3**	APC7	Anaphase-promoting complex subunit 7 OS=Homo sapiens OX=9606 GN=ANAPC7 PE=1 SV=4	Component of the anaphase promoting complex/cyclosome (APC/C), a cell cycle-regulated E3 ubiquitin ligase that controls progression through mitosis and the G1 phase of the cell cycle. The APC/C complex acts by mediating ubiquitination and subsequent degradation of target proteins- it mainly mediates the formation of 'Lys-11'-linked polyubiquitin chains and, to a lower extent, the formation of 'Lys-48'- and 'Lys-63'-linked polyubiquitin chains; Belongs to the APC7 family (599 aa)

**P62424**	RL7A	60S ribosomal protein L7a OS=Homo sapiens OX=9606 GN=RPL7A PE=1 SV=2	Cytoplasmic ribosomes, organelles that catalyze protein synthesis, consist of a small 40S subunit and a large 60S subunit. Together these subunits are composed of 4 RNA species and approximately 80 structurally distinct proteins. This gene encodes a ribosomal protein that is a component of the 60S subunit. The protein belongs to the L7AE family of ribosomal proteins. It can interact with a subclass of nuclear hormone receptors, including thyroid hormone receptor, and inhibit their ability to transactivate by preventing their binding to their DNA response elements. This gene is included in the surfeit gene cluster, a group of very tightly linked genes that do not share sequence similarity. It is co-transcribed with the U24, U36a, U36b, and U36c small nucleolar RNA genes, which are located in its second, fifth, fourth, and sixth introns, respectively. This gene rearranges with the trk proto-oncogene to form the chimeric oncogene trk-2h, which encodes an oncoprotein consisting of the N terminus of ribosomal protein L7a fused to the receptor tyrosine kinase domain of trk. As is typical for genes encoding ribosomal proteins, there are multiple processed pseudogenes of this gene dispersed through the genome
**Q969Q0**	RL36L	60S ribosomal protein L36a-like OS=Homo sapiens OX=9606 GN=RPL36AL PE=1 SV=3	Cytoplasmic ribosomes, organelles that catalyze protein synthesis, consist of a small 40S subunit and a large 60S subunit. Together these subunits are composed of 4 RNA species and approximately 80 structurally distinct proteins. This gene encodes a ribosomal protein that is a component of the 60S subunit. The protein, which shares sequence similarity with yeast ribosomal protein L44, belongs to the L44E (L36AE) family of ribosomal proteins. This gene and the human gene officially named ribosomal protein L36a (RPL36A) encode nearly identical proteins; however, they are distinct genes. Although the name of this gene has been referred to as ribosomal protein L36a (RPL36A), its official name is ribosomal protein L36a-like (RPL36AL). As is typical for genes encoding ribosomal proteins, there are multiple processed pseudogenes of this gene dispersed through the genome.
**Q9NWV4**	CZIB	CXXC motif containing zinc binding protein OS=Homo sapiens OX=9606 GN=CZIB PE=1 SV=1	CZIB (CXXC Motif Containing Zinc Binding Protein) is a Protein Coding gene.

**Q6P6C2**	ALKB5	RNA demethylase ALKBH5 OS=Homo sapiens OX=9606 GN=ALKBH5 PE=1 SV=2	Dioxygenase that demethylates RNA by oxidative demethylation: specifically demethylates N6-methyladenosine (m6A) RNA, the most prevalent internal modification of messenger RNA (mRNA) in higher eukaryotes (PubMed:23177736, PubMed:24489119, PubMed:24616105, PubMed:24778178). Can also demethylate N6-methyladenosine in single-stranded DNA (in vitro) (PubMed:24616105). Requires molecular oxygen, alpha-ketoglutarate and iron (PubMed:21264265, PubMed:23177736, PubMed:24489119, PubMed:24616105, PubMed:24778178). Demethylation of m6A mRNA affects mRNA processing and export (PubMed:23177736). Required for the late meiotic and haploid phases of spermatogenesis by mediating m6A demethylation in spermatocytes and round spermatids: m6A demethylation of target transcripts is required for correct splicing and the production of longer 3'-UTR mRNAs in male germ cells (By similarity).
**O15523**	DDX3Y	ATP-dependent RNA helicase DDX3Y OS=Homo sapiens OX=9606 GN=DDX3Y PE=1 SV=2	The protein encoded by this gene is a member of the DEAD-box RNA helicase family, characterized by nine conserved motifs, included the conserved Asp-Glu-Ala-Asp (DEAD) motif. These motifs are thought to be involved in ATP binding, hydrolysis, RNA binding, and in the formation of intramolecular interactions. This protein shares high similarity to DDX3X, on the X chromosome, but a deletion of this gene is not complemented by DDX3X. Mutations in this gene result in male infertility, a reduction in germ cell numbers, and can result in Sertoli-cell only sydrome. Pseudogenes sharing similarity to both this gene and the DDX3X paralog are found on chromosome 4 and the X chromosome. Alternative splicing results in multiple transcript variants encoding different isoforms
**Q5VTE0**	EF1A3	Putative elongation factor 1-alpha-like 3 OS=Homo sapiens OX=9606 GN=EEF1A1P5 PE=5 SV=1	EEF1A1P5 (Eukaryotic Translation Elongation Factor 1 Alpha 1 Pseudogene 5) is a Pseudogene. Among its related pathways are Viral mRNA Translation and Gene Expression. Gene Ontology (GO) annotations related to this gene include GTP binding and translation elongation factor activity.
**Q96CP2**	FWCH2	FLYWCH family member 2 OS=Homo sapiens OX=9606 GN=FLYWCH2 PE=1 SV=1	FLYWCH2 (FLYWCH Family Member 2) is a Protein Coding gene. An important paralog of this gene is FLYWCH1.
**O75343**	GCYB2	Guanylate cyclase soluble subunit beta-2 OS=Homo sapiens OX=9606 GN=GUCY1B2 PE=5 SV=2	Guanylyl Cyclases (GC) are a group of enzymes that, in the presence of a metal ion co-factor such as Mg2+ or Mn2+, convert guanosine-5'-triphosphate (GTP) into 3',5'-guanosine monophosphate (cGMP) and pyrophosphate. There are two main groups of guanylyl cyclase.

**O96028**	NSD2	Histone-lysine N-methyltransferase NSD2 OS=Homo sapiens OX=9606 GN=NSD2 PE=1 SV=1	Histone methyltransferase with histone H3 'Lys-27' (H3K27me) methyltransferase activity. Isoform 2 may act as a transcription regulator that binds DNA and suppresses IL5 transcription through HDAC recruitment.
**O96019**	ACL6A	Actin-like protein 6A OS=Homo sapiens OX=9606 GN=ACTL6A PE=1 SV=1	Involved in transcriptional activation and repression of select genes by chromatin remodeling (alteration of DNA-nucleosome topology). Component of SWI/SNF chromatin remodeling complexes that carry out key enzymatic activities, changing chromatin structure by altering DNA-histone contacts within a nucleosome in an ATP-dependent manner. Required for maximal ATPase activity of SMARCA4/BRG1/BAF190A and for association of the SMARCA4/BRG1/BAF190A containing remodeling complex BAF with chromatin/nuclear matrix. Belongs to the neural progenitors-specific chromatin remodeling complex (npBAF complex) and is required for the proliferation of neural progenitors.
**Q8N6M0**	OTU6B	Deubiquitinase OTUD6B OS=Homo sapiens OX=9606 GN=OTUD6B PE=1 SV=1	Isoform 1: Deubiquitinating enzyme that may play a role in the ubiquitin-dependent regulation of protein synthesis, downstream of mTORC1 (PubMed:21267069, PubMed:27864334). May associate with the protein synthesis initiation complex and modify its ubiquitination to repress translation (PubMed:27864334). May also repress DNA synthesis and modify different cellular targets thereby regulating cell growth and proliferation (PubMed:27864334). May also play a role in proteasome assembly and function (PubMed:28343629).<br><br>Isoform 2: Stimulates protein synthesis. Influences the expression of CCND1/cyclin D1 by promoting its translation and regulates MYC/c-Myc protein stability.

**Q7Z7F7**	RM55	39S ribosomal protein L55, mitochondrial OS=Homo sapiens OX=9606 GN=MRPL55 PE=1 SV=1	Mammalian mitochondrial ribosomal proteins are encoded by nuclear genes and help in protein synthesis within the mitochondrion. Mitochondrial ribosomes (mitoribosomes) consist of a small 28S subunit and a large 39S subunit. They have an estimated 75% protein to rRNA composition compared to prokaryotic ribosomes, where this ratio is reversed. Another difference between mammalian mitoribosomes and prokaryotic ribosomes is that the latter contain a 5S rRNA. Among different species, the proteins comprising the mitoribosome differ greatly in sequence, and sometimes in biochemical properties, which prevents easy recognition by sequence homology. This gene encodes a 39S subunit protein. Multiple transcript variants encoding two different isoforms were identified through sequence analysis
**Q96GC5**	RM48	39S ribosomal protein L48, mitochondrial OS=Homo sapiens OX=9606 GN=MRPL48 PE=1 SV=2	Mammalian mitochondrial ribosomal proteins are encoded by nuclear genes and help in protein synthesis within the mitochondrion. Mitochondrial ribosomes (mitoribosomes) consist of a small 28S subunit and a large 39S subunit. They have an estimated 75% protein to rRNA composition compared to prokaryotic ribosomes, where this ratio is reversed. Another difference between mammalian mitoribosomes and prokaryotic ribosomes is that the latter contain a 5S rRNA. Among different species, the proteins comprising the mitoribosome differ greatly in sequence, and sometimes in biochemical properties, which prevents easy recognition by sequence homology. This gene encodes a 39S subunit protein. A pseudogene corresponding to this gene is found on chromosome 6p. Several transcript variants, some protein-coding and some non-protein coding, have been found for this gene.
**P42167**	LAP2B	Lamina-associated polypeptide 2, isoforms beta/gamma OS=Homo sapiens OX=9606 GN=TMPO PE=1 SV=2	May be involved in the structural organization of the nucleus and in the post-mitotic nuclear assembly. Plays an important role, together with LMNA, in the nuclear anchorage of RB1; Belongs to the LEM family (694 aa)
**Q09666**	AHNK	Neuroblast differentiation-associated protein AHNAK OS=Homo sapiens OX=9606 GN=AHNAK PE=1 SV=2	May be required for neuronal cell differentiation; PDZ domain containing (5890 aa)

**Q8IWZ3**	ANKH1	Ankyrin repeat and KH domain-containing protein 1 OS=Homo sapiens OX=9606 GN=ANKHD1 PE=1 SV=1	May play a role as a scaffolding protein that may be associated with the abnormal phenotype of leukemia cells. Isoform 2 may possess an antiapoptotic effect and protect cells during normal cell survival through its regulation of caspases.
**Q6UB98**	ANR12	Ankyrin repeat domain-containing protein 12 OS=Homo sapiens OX=9606 GN=ANKRD12 PE=1 SV=3	May recruit HDACs to the p160 coactivators/nuclear receptor complex to inhibit ligand-dependent transactivation; Ankyrin repeat domain containing (2062 aa)
**O15392**	BIRC5	Baculoviral IAP repeat-containing protein 5 OS=Homo sapiens OX=9606 GN=BIRC5 PE=1 SV=3	Multitasking protein that has dual roles in promoting cell proliferation and preventing apoptosis (PubMed:9859993, PubMed:21364656, PubMed:20627126). Component of a chromosome passage protein complex (CPC) which is essential for chromosome alignment and segregation during mitosis and cytokinesis (PubMed:16322459). Acts as an important regulator of the localization of this complex; directs CPC movement to different locations from the inner centromere during prometaphase to midbody during cytokinesis and participates in the organization of the center spindle by associating with polymerized microtubules (PubMed:20826784). Involved in the recruitment of CPC to centromeres during early mitosis via association with histone H3 phosphorylated at 'Thr-3' (H3pT3) during mitosis (PubMed:20929775). The complex with RAN plays a role in mitotic spindle formation by serving as a physical scaffold to help deliver the RAN effector molecule TPX2 to microtubules (PubMed:18591255). May counteract a default induction of apoptosis in G2/M phase (PubMed:9859993). The acetylated form represses STAT3 transactivation of target gene promoters (PubMed:20826784). May play a role in neoplasia (PubMed:10626797). Inhibitor of CASP3 and CASP7 (PubMed:21536684). Isoform 2 and isoform 3 do not appear to play vital roles in mitosis (PubMed:12773388, PubMed:16291752). Isoform 3 shows a marked reduction in its anti-apoptotic effects when compared with the displayed wild-type isoform (PubMed:10626797).

**Q9BRP8**	PYM1	Partner of Y14 and mago OS=Homo sapiens OX=9606 GN=PYM1 PE=1 SV=1	Key regulator of the exon junction complex (EJC), a multiprotein complex that associates immediately upstream of the exon-exon junction on mRNAs and serves as a positional landmark for the intron exon structure of genes and directs post-transcriptional processes in the cytoplasm such as mRNA export, nonsense-mediated mRNA decay (NMD) or translation. Acts as an EJC disassembly factor, allowing translation-dependent EJC removal and recycling by disrupting mature EJC from spliced mRNAs. Its association with the 40S ribosomal subunit probably prevents a translation-independent disassembly of the EJC from spliced mRNAs, by restricting its activity to mRNAs that have been translated. Interferes with NMD and enhances translation of spliced mRNAs, probably by antagonizing EJC functions. May bind RNA; the relevance of RNA-binding remains unclear in vivo, RNA-binding was detected by PubMed:14968132, while PubMed:19410547 did not detect RNA-binding activity independently of the EJC.
**Q2VIR3**	IF2GL	Eukaryotic translation initiation factor 2 subunit 3B OS=Homo sapiens OX=9606 GN=EIF2S3B PE=2 SV=2	Putative eukaryotic translation initiation factor 2 subunit 3-like protein; As a subunit of eukaryotic initiation factor 2 (eIF2), involved in the early steps of protein synthesis. In the presence of GTP, eIF2 forms a ternary complex with initiator tRNA Met-tRNAi and then recruits the 40S ribosomal complex, a step that determines the rate of protein translation. This step is followed by mRNA binding to form the 43S pre-initiation complex. Junction of the 60S ribosomal subunit to form the 80S initiation complex is preceded by hydrolysis of the GTP bound to eIF2 and release of an eIF2-GD
**Q58FF7**	H90B3	Putative heat shock protein HSP 90-beta-3 OS=Homo sapiens OX=9606 GN=HSP90AB3P PE=5 SV=1	Putative molecular chaperone that may promote the maturation, structural maintenance and proper regulation of specific target proteins.

**P63244**	RACK1	Receptor of activated protein C kinase 1 OS=Homo sapiens OX=9606 GN=RACK1 PE=1 SV=3	Scaffolding protein involved in the recruitment, assembly and/or regulation of a variety of signaling molecules. Interacts with a wide variety of proteins and plays a role in many cellular processes. Component of the 40S ribosomal subunit involved in translational repression (PubMed:23636399). Involved in the initiation of the ribosome quality control (RQC), a pathway that takes place when a ribosome has stalled during translation, by promoting ubiquitination of a subset of 40S ribosomal subunits (PubMed:28132843). Binds to and stabilizes activated protein kinase C (PKC), increasing PKC-mediated phosphorylation. May recruit activated PKC to the ribosome, leading to phosphorylation of EIF6. Inhibits the activity of SRC kinases including SRC, LCK and YES1. Inhibits cell growth by prolonging the G0/G1 phase of the cell cycle. Enhances phosphorylation of BMAL1 by PRKCA and inhibits transcriptional activity of the BMAL1-CLOCK heterodimer. Facilitates ligand-independent nuclear translocation of AR following PKC activation, represses AR transactivation activity and is required for phosphorylation of AR by SRC. Modulates IGF1R-dependent integrin signaling and promotes cell spreading and contact with the extracellular matrix. Involved in PKC-dependent translocation of ADAM12 to the cell membrane. Promotes the ubiquitination and proteasome-mediated degradation of proteins such as CLEC1B and HIF1A. Required for VANGL2 membrane localization, inhibits Wnt signaling, and regulates cellular polarization and oriented cell division during gastrulation. Required for PTK2/FAK1 phosphorylation and dephosphorylation. Regulates [illegible]
**Q9BTL3**	RAMAC	RNA guanine-N7 methyltransferase activating subunit OS=Homo sapiens OX=9606 GN=RAMAC PE=1 SV=1	Regulatory subunit of the mRNA-capping methyltransferase RNMT:RAMAC complex that methylates the N7 position of the added guanosine to the 5'-cap structure of mRNAs (PubMed:22099306, PubMed:27422871). Promotes the recruitment of the methyl donor, S-adenosyl-L-methionine, to RNMT (PubMed:27422871). Regulates RNMT expression by a post-transcriptional stabilizing mechanism (PubMed:22099306). Binds RNA (PubMed:22099306).

**Q02543**	RL18A	60S ribosomal protein L18a OS=Homo sapiens OX=9606 GN=RPL18A PE=1 SV=2	Ribosomes, the organelles that catalyze protein synthesis, consist of a small 40S subunit and a large 60S subunit. Together these subunits are composed of 4 RNA species and approximately 80 structurally distinct proteins. This gene encodes a member of the L18AE family of ribosomal proteins that is a component of the 60S subunit. The encoded protein may play a role in viral replication by interacting with the hepatitis C virus internal ribosome entry site (IRES). This gene is co-transcribed with the U68 snoRNA, located within the third intron. As is typical for genes encoding ribosomal proteins, there are multiple processed pseudogenes of this gene dispersed throughout the genome
**P15880**	RS2	40S ribosomal protein S2 OS=Homo sapiens OX=9606 GN=RPS2 PE=1 SV=2	Ribosomes, the organelles that catalyze protein synthesis, consist of a small 40S subunit and a large 60S subunit. Together these subunits are composed of 4 RNA species and approximately 80 structurally distinct proteins. This gene encodes a ribosomal protein that is a component of the 40S subunit. The protein belongs to the S5P family of ribosomal proteins. It is located in the cytoplasm. This gene shares sequence similarity with mouse LLRep3. It is co-transcribed with the small nucleolar RNA gene U64, which is located in its third intron. As is typical for genes encoding ribosomal proteins, there are multiple processed pseudogenes of this gene dispersed through the genome.
**P25398**	RS12	40S ribosomal protein S12 OS=Homo sapiens OX=9606 GN=RPS12 PE=1 SV=3	Ribosomes, the organelles that catalyze protein synthesis, consist of a small 40S subunit and a large 60S subunit. Together these subunits are composed of 4 RNA species and approximately 80 structurally distinct proteins. This gene encodes a ribosomal protein that is a component of the 40S subunit. The protein belongs to the S12E family of ribosomal proteins. It is located in the cytoplasm. Increased expression of this gene in colorectal cancers compared to matched normal colonic mucosa has been observed. As is typical for genes encoding ribosomal proteins, there are multiple processed pseudogenes of this gene dispersed through the genome

**P26373**	RL13	60S ribosomal protein L13 OS=Homo sapiens OX=9606 GN=RPL13 PE=1 SV=4	Ribosomes, the organelles that catalyze protein synthesis, consist of a small 40S subunit and a large 60S subunit. Together these subunits are composed of 4 RNA species and approximately 80 structurally distinct proteins. This gene encodes a ribosomal protein that is a component of the 60S subunit. The protein belongs to the L13E family of ribosomal proteins. It is located in the cytoplasm. This gene is expressed at significantly higher levels in benign breast lesions than in breast carcinomas. Alternatively spliced transcript variants encoding distinct isoforms have been found for this gene. As is typical for genes encoding ribosomal proteins, there are multiple processed pseudogenes of this gene dispersed through the genome
**P32969**	RL9	60S ribosomal protein L9 OS=Homo sapiens OX=9606 GN=RPL9 PE=1 SV=1	Ribosomes, the organelles that catalyze protein synthesis, consist of a small 40S subunit and a large 60S subunit. Together these subunits are composed of 4 RNA species and approximately 80 structurally distinct proteins. This gene encodes a ribosomal protein that is a component of the 60S subunit. The protein belongs to the L6P family of ribosomal proteins. It is located in the cytoplasm. As is typical for genes encoding ribosomal proteins, there are multiple processed pseudogenes of this gene dispersed through the genome. Alternative splicing results in multiple transcript variants
**P36578**	RL4	60S ribosomal protein L4 OS=Homo sapiens OX=9606 GN=RPL4 PE=1 SV=5	Ribosomes, the organelles that catalyze protein synthesis, consist of a small 40S subunit and a large 60S subunit. Together these subunits are composed of 4 RNA species and approximately 80 structurally distinct proteins. This gene encodes a ribosomal protein that is a component of the 60S subunit. The protein belongs to the L4E family of ribosomal proteins. It is located in the cytoplasm. As is typical for genes encoding ribosomal proteins, there are multiple processed pseudogenes of this gene dispersed through the genome

**P46781**	RS9	40S ribosomal protein S9 OS=Homo sapiens OX=9606 GN=RPS9 PE=1 SV=3	Ribosomes, the organelles that catalyze protein synthesis, consist of a small 40S subunit and a large 60S subunit. Together these subunits are composed of 4 RNA species and approximately 80 structurally distinct proteins. This gene encodes a ribosomal protein that is a component of the 40S subunit. The protein belongs to the S4P family of ribosomal proteins. It is located in the cytoplasm. Variable expression of this gene in colorectal cancers compared to adjacent normal tissues has been observed, although no correlation between the level of expression and the severity of the disease has been found. As is typical for genes encoding ribosomal proteins, multiple processed pseudogenes derived from this gene are dispersed through the genome
**P60866**	RS20	40S ribosomal protein S20 OS=Homo sapiens OX=9606 GN=RPS20 PE=1 SV=1	Ribosomes, the organelles that catalyze protein synthesis, consist of a small 40S subunit and a large 60S subunit. Together these subunits are composed of 4 RNA species and approximately 80 structurally distinct proteins. This gene encodes a ribosomal protein that is a component of the 40S subunit. The protein belongs to the S10P family of ribosomal proteins. It is located in the cytoplasm. This gene is co-transcribed with the small nucleolar RNA gene U54, which is located in its second intron. As is typical for genes encoding ribosomal proteins, there are multiple processed pseudogenes of this gene dispersed through the genome. Two transcript variants encoding different isoforms have been identified for this gene
**P61254**	RL26	60S ribosomal protein L26 OS=Homo sapiens OX=9606 GN=RPL26 PE=1 SV=1	Ribosomes, the organelles that catalyze protein synthesis, consist of a small 40S subunit and a large 60S subunit. Together these subunits are composed of 4 RNA species and approximately 80 structurally distinct proteins. This gene encodes a ribosomal protein that is a component of the 60S subunit. The protein belongs to the L24P family of ribosomal proteins. It is located in the cytoplasm. As is typical for genes encoding ribosomal proteins, there are multiple processed pseudogenes of this gene dispersed through the genome. Mutations in this gene result in Diamond-Blackfan anemia. Alternative splicing results in multiple transcript variants

**P62249**	RS16	40S ribosomal protein S16 OS=Homo sapiens OX=9606 GN=RPS16 PE=1 SV=2	Ribosomes, the organelles that catalyze protein synthesis, consist of a small 40S subunit and a large 60S subunit. Together these subunits are composed of 4 RNA species and approximately 80 structurally distinct proteins. This gene encodes a ribosomal protein that is a component of the 40S subunit. The protein belongs to the S9P family of ribosomal proteins. It is located in the cytoplasm. As is typical for genes encoding ribosomal proteins, there are multiple processed pseudogenes of this gene dispersed through the genome
**P62263**	RS14	40S ribosomal protein S14 OS=Homo sapiens OX=9606 GN=RPS14 PE=1 SV=3	Ribosomes, the organelles that catalyze protein synthesis, consist of a small 40S subunit and a large 60S subunit. Together these subunits are composed of 4 RNA species and approximately 80 structurally distinct proteins. This gene encodes a ribosomal protein that is a component of the 40S subunit. The protein belongs to the S11P family of ribosomal proteins. It is located in the cytoplasm. Transcript variants utilizing alternative transcription initiation sites have been described in the literature. As is typical for genes encoding ribosomal proteins, there are multiple processed pseudogenes of this gene dispersed through the genome. In Chinese hamster ovary cells, mutations in this gene can lead to resistance to emetine, a protein synthesis inhibitor. Multiple alternatively spliced transcript variants encoding the same protein have been found for this gene

**P62273**	RS29	40S ribosomal protein S29 OS=Homo sapiens OX=9606 GN=RPS29 PE=1 SV=2	Ribosomes, the organelles that catalyze protein synthesis, consist of a small 40S subunit and a large 60S subunit. Together these subunits are composed of 4 RNA species and approximately 80 structurally distinct proteins. This gene encodes a ribosomal protein that is a component of the 40S subunit and a member of the S14P family of ribosomal proteins. The protein, which contains a C2-C2 zinc finger-like domain that can bind to zinc, can enhance the tumor suppressor activity of Ras-related protein 1A (KREV1). It is located in the cytoplasm. Variable expression of this gene in colorectal cancers compared to adjacent normal tissues has been observed, although no correlation between the level of expression and the severity of the disease has been found. As is typical for genes encoding ribosomal proteins, there are multiple processed pseudogenes of this gene dispersed through the genome. Alternatively spliced transcript variants encoding different isoforms have been found for this gene
**P62277**	RS13	40S ribosomal protein S13 OS=Homo sapiens OX=9606 GN=RPS13 PE=1 SV=2	Ribosomes, the organelles that catalyze protein synthesis, consist of a small 40S subunit and a large 60S subunit. Together these subunits are composed of 4 RNA species and approximately 80 structurally distinct proteins. This gene encodes a ribosomal protein that is a component of the 40S subunit. The protein belongs to the S15P family of ribosomal proteins. It is located in the cytoplasm. The protein has been shown to bind to the 5.8S rRNA in rat. The gene product of the E. coli ortholog (ribosomal protein S15) functions at early steps in ribosome assembly. This gene is co-transcribed with two U14 small nucleolar RNA genes, which are located in its third and fifth introns. As is typical for genes encoding ribosomal proteins, there are multiple processed pseudogenes of this gene dispersed through the genome

**P62829**	RL23	60S ribosomal protein L23 OS=Homo sapiens OX=9606 GN=RPL23 PE=1 SV=1	Ribosomes, the organelles that catalyze protein synthesis, consist of a small 40S subunit and a large 60S subunit. Together these subunits are composed of 4 RNA species and approximately 80 structurally distinct proteins. This gene encodes a ribosomal protein that is a component of the 60S subunit. The protein belongs to the L14P family of ribosomal proteins. It is located in the cytoplasm. This gene has been referred to as rpL17 because the encoded protein shares amino acid identity with ribosomal protein L17 from Saccharomyces cerevisiae; however, its official symbol is RPL23. As is typical for genes encoding ribosomal proteins, there are multiple processed pseudogenes of this gene dispersed through the genome
**P62841**	RS15	40S ribosomal protein S15 OS=Homo sapiens OX=9606 GN=RPS15 PE=1 SV=2	Ribosomes, the organelles that catalyze protein synthesis, consist of a small 40S subunit and a large 60S subunit. Together these subunits are composed of 4 RNA species and approximately 80 structurally distinct proteins. This gene encodes a ribosomal protein that is a component of the 40S subunit. The protein belongs to the S19P family of ribosomal proteins. It is located in the cytoplasm. This gene has been found to be activated in various tumors, such as insulinomas, esophageal cancers, and colon cancers. As is typical for genes encoding ribosomal proteins, there are multiple processed pseudogenes of this gene dispersed through the genome. Alternative splicing results in multiple transcript variants
**P62851**	RS25	40S ribosomal protein S25 OS=Homo sapiens OX=9606 GN=RPS25 PE=1 SV=1	Ribosomes, the organelles that catalyze protein synthesis, consist of a small 40S subunit and a large 60S subunit. Together these subunits are composed of 4 RNA species and approximately 80 structurally distinct proteins. This gene encodes a ribosomal protein that is a component of the 40S subunit. The protein belongs to the S25E family of ribosomal proteins. It is located in the cytoplasm. As is typical for genes encoding ribosomal proteins, there are multiple processed pseudogenes of this gene dispersed through the genome

**P62899**	RL31	60S ribosomal protein L31 OS=Homo sapiens OX=9606 GN=RPL31 PE=1 SV=1	Ribosomes, the organelles that catalyze protein synthesis, consist of a small 40S subunit and a large 60S subunit. Together these subunits are composed of 4 RNA species and approximately 80 structurally distinct proteins. This gene encodes a ribosomal protein that is a component of the 60S subunit. The protein belongs to the L31E family of ribosomal proteins. It is located in the cytoplasm. Higher levels of expression of this gene in familial adenomatous polyps compared to matched normal tissues have been observed. As is typical for genes encoding ribosomal proteins, there are multiple processed pseudogenes of this gene dispersed through the genome. Alternatively spliced transcript variants encoding distinct isoforms have been found for this gene
**P61313**	RL15	60S ribosomal protein L15 OS=Homo sapiens OX=9606 GN=RPL15 PE=1 SV=2	Ribosomes, the organelles that catalyze protein synthesis, consist of a small 40S subunit and a large 60S subunit. Together these subunits are composed of four RNA species and approximately 80 structurally distinct proteins. This gene encodes a member of the L15E family of ribosomal proteins and a component of the 60S subunit. This gene shares sequence similarity with the yeast ribosomal protein YL10 gene. Elevated expression of this gene has been observed in esophageal tumors and gastric cancer tissues, and deletion of this gene has been observed in a Diamond-Blackfan anemia (DBA) patient. As is typical for genes encoding ribosomal proteins, there are multiple processed pseudogenes of this gene dispersed through the genome
**Q9Y224**	RTRAF	RNA transcription, translation and transport factor protein OS=Homo sapiens OX=9606 GN=RTRAF PE=1 SV=1	RNA-binding protein involved in modulation of mRNA transcription by Polymerase II (PubMed:16950395). Component of the tRNA-splicing ligase complex and is required for tRNA ligation (PubMed:24870230). May be required for RNA transport (PubMed:24608264).<br><br>(Microbial infection) In case of infection by influenza virus A (IVA), is involved in viral replication (PubMed:21900157).
**Q59GN2**	R39L5	Putative 60S ribosomal protein L39-like 5 OS=Homo sapiens OX=9606 GN=RPL39P5 PE=5 SV=2	RPL39P5 (Ribosomal Protein L39 Pseudogene 5) is a Pseudogene. Gene Ontology (GO) annotations related to this gene include structural constituent of ribosome
**Q6DKI1**	RL7L	60S ribosomal protein L7-like 1 OS=Homo sapiens OX=9606 GN=RPL7L1 PE=1 SV=2	RPL7L1 (Ribosomal Protein L7 Like 1) is a Protein Coding gene. An important paralog of this gene is RPL7.

**Q8NHW5**	RLA0L	60S acidic ribosomal protein P0-like OS=Homo sapiens OX=9606 GN=RPLP0P6 PE=5 SV=1	RPLP0P6 (Ribosomal Protein Lateral Stalk Subunit P0 Pseudogene 6) is a Pseudogene.
**Q9NQ39**	RS10L	Putative 40S ribosomal protein S10-like OS=Homo sapiens OX=9606 GN=RPS10P5 PE=5 SV=1	RPS10P5 (Ribosomal Protein S10 Pseudogene 5) is a Pseudogene.
**P08708**	RS17	40S ribosomal protein S17 OS=Homo sapiens OX=9606 GN=RPS17 PE=1 SV=2	Ribosomes, the organelles that catalyze protein synthesis, consist of a small 40S subunit and a large 60S subunit. Together these subunits are composed of four RNA species and approximately 80 structurally distinct proteins. This gene encodes a ribosomal protein that is a component of the 40S subunit. The protein belongs to the S17E family of ribosomal proteins and is located in the cytoplasm. Mutations in this gene cause Diamond-Blackfan anemia 4. Alternative splicing of this gene results in multiple transcript variants. As is typical for genes encoding ribosomal proteins, there are multiple processed pseudogenes of this gene dispersed through the genome
**Q5JNZ5**	RS26L	Putative 40S ribosomal protein S26-like 1 OS=Homo sapiens OX=9606 GN=RPS26P11 PE=5 SV=1	RPS26P11 (Ribosomal Protein S26 Pseudogene 11) is a Pseudogene.
**P02768**	ALBU	Serum albumin OS=Homo sapiens OX=9606 GN=ALB PE=1 SV=2	Serum albumin, the main protein of plasma, has a good binding capacity for water, Ca2+, Na+, K+, fatty acids, hormones, bilirubin and drugs. Its main function is the regulation of the colloidal osmotic pressure of blood. Major zinc transporter in plasma, typically binds about 80% of all plasma zinc

**Q5FBB7**	SGO1	Shugoshin 1 OS=Homo sapiens OX=9606 GN=SGO1 PE=1 SV=1	Plays a central role in chromosome cohesion during mitosis by preventing premature dissociation of cohesin complex from centromeres after prophase, when most of cohesin complex dissociates from chromosomes arms. May act by preventing phosphorylation of the STAG2 subunit of cohesin complex at the centromere, ensuring cohesin persistence at centromere until cohesin cleavage by ESPL1/separase at anaphase. Essential for proper chromosome segregation during mitosis and this function requires interaction with PPP2R1A. Its phosphorylated form is necessary for chromosome congression and for the proper attachment of spindle microtubule to the kinetochore. Necessary for kinetochore localization of PLK1 and CENPF. May play a role in the tension sensing mechanism of the spindle-assembly checkpoint by regulating PLK1 kinetochore affinity. Isoform 3 plays a role in maintaining centriole cohesion involved in controlling spindle pole integrity. Involved in centromeric enrichment of AUKRB in prometaphase.
**Q9BWG4**	SSBP4	Single-stranded DNA-binding protein 4 OS=Homo sapiens OX=9606 GN=SSBP4 PE=1 SV=1	SSBP4 (Single Stranded DNA Binding Protein 4) is a Protein Coding gene. Gene Ontology (GO) annotations related to this gene include single-stranded DNA binding. An important paralog of this gene is SSBP3.
**P42285**	MTREX	Exosome RNA helicase MTR4 OS=Homo sapiens OX=9606 GN=MTREX PE=1 SV=3	Component of exosome targeting complexes. Subunit of the trimeric nuclear exosome targeting (NEXT) complex, a complex that directs a subset of non-coding short-lived RNAs for exosomal degradation. Subunit of the trimeric poly(A) tail exosome targeting (PAXT) complex, a complex that directs a subset of long and polyadenylated poly(A) RNAs for exosomal degradation. The RNA exosome is fundamental for the degradation of RNA in eukaryotic nuclei. Substrate targeting is facilitated by its cofactor MTREX, which links to RNA-binding protein adapters (PubMed:27871484). Associated with the RNA exosome complex and involved in the 3'-processing of the 7S pre-RNA to the mature 5.8S rRNA (PubMed:17412707, PubMed:29107693). May be involved in pre-mRNA splicing.
**Q9BRI3**	ZNT2	Zinc transporter 2 OS=Homo sapiens OX=9606 GN=SLC30A2 PE=1 SV=1	The protein encoded by this gene is a zinc transporter that acts as a homodimer. The encoded protein plays a role in secreting zinc into breast milk. Two transcript variants encoding different isoforms have been found for this gene

**Q9UG63**	ABCF2	ATP-binding cassette sub-family F member 2 OS=Homo sapiens OX=9606 GN=ABCF2 PE=1 SV=2	This gene encodes a member of the ATP-binding cassette (ABC) transporter superfamily. ATP-binding cassette proteins transport various molecules across extra- and intracellular membranes. Alterations in this gene may be involved in cancer progression. Alternative splicing results in multiple transcript variants. Related pseudogenes have been identified on chromosomes 3 and 7.
**E9PRG8**	CK098	Uncharacterized protein C11orf98 OS=Homo sapiens OX=9606 GN=C11orf98 PE=4 SV=1	This gene shares three exons in common with another gene, LBH domain containing 1 (GeneID:79081), but the encoded protein uses a reading frame that is different from that of the LBH domain containing 1 gene
**P0DMV9**	HS71B	Heat shock 70 kDa protein 1B OS=Homo sapiens OX=9606 GN=HSPA1B PE=1 SV=1	This intronless gene encodes a 70kDa heat shock protein which is a member of the heat shock protein 70 family. In conjuction with other heat shock proteins, this protein stabilizes existing proteins against aggregation and mediates the folding of newly translated proteins in the cytosol and in organelles. It is also involved in the ubiquitin-proteasome pathway through interaction with the AU-rich element RNA-binding protein 1. The gene is located in the major histocompatibility complex class III region, in a cluster with two closely related genes which encode similar proteins
**Q96EY4**	TMA16	Translation machinery-associated protein 16 OS=Homo sapiens OX=9606 GN=TMA16 PE=1 SV=2	TMA16 (Translation Machinery Associated 16 Homolog) is a Protein Coding gene.
**P06576**	ATPB	ATP synthase subunit beta, mitochondrial OS=Homo sapiens OX=9606 GN=ATP5F1B PE=1 SV=3	Mitochondrial membrane ATP synthase (F(1)F(0) ATP synthase or Complex V) produces ATP from ADP in the presence of a proton gradient across the membrane which is generated by electron transport complexes of the respiratory chain. F-type ATPases consist of two structural domains, F(1) - containing the extramembraneous catalytic core, and F(0) - containing the membrane proton channel, linked together by a central stalk and a peripheral stalk. During catalysis, ATP synthesis in the catalytic domain of F(1) is coupled via a rotary mechanism of the central stalk subunits to proton translocation. Subunits alpha and beta form the catalytic core in F(1). Rotation of the central stalk against the surrounding alpha(3)beta(3) subunits leads to hydrolysis of ATP in three separate catalytic sites on the beta subunits.

**Q01081**	U2AF1	Splicing factor U2AF 35 kDa subunit OS=Homo sapiens OX=9606 GN=U2AF1 PE=1 SV=3	Plays a critical role in both constitutive and enhancer-dependent splicing by mediating protein-protein interactions and protein-RNA interactions required for accurate 3'-splice site selection. Recruits U2 snRNP to the branch point. Directly mediates interactions between U2AF2 and proteins bound to the enhancers and thus may function as a bridge between U2AF2 and the enhancer complex to recruit it to the adjacent intron.
**Q92890**	UFD1	Ubiquitin recognition factor in ER-associated degradation protein 1 OS=Homo sapiens OX=9606 GN=UFD1 PE=1 SV=3	Essential component of the ubiquitin-dependent proteolytic pathway which degrades ubiquitin fusion proteins. The ternary complex containing UFD1, VCP and NPLOC4 binds ubiquitinated proteins and is necessary for the export of misfolded proteins from the ER to the cytoplasm, where they are degraded by the proteasome. The NPLOC4-UFD1-VCP complex regulates spindle disassembly at the end of mitosis and is necessary for the formation of a closed nuclear envelope. It may be involved in the development of some ectoderm-derived structures (By similarity). Acts as a negative regulator of type I interferon production via the complex formed with VCP and NPLOC4, which binds to DDX58/RIG-I and recruits RNF125 to promote ubiquitination and degradation of DDX58/RIG-I (PubMed:26471729).
**Q9P035**	HACD3	Very-long-chain (3R)-3-hydroxyacyl-CoA dehydratase 3 OS=Homo sapiens OX=9606 GN=HACD3 PE=1 SV=2	Catalyzes the third of the four reactions of the long-chain fatty acids elongation cycle. This endoplasmic reticulum-bound enzymatic process, allows the addition of two carbons to the chain of long- and very long-chain fatty acids/VLCFAs per cycle. This enzyme catalyzes the dehydration of the 3-hydroxyacyl-CoA intermediate into trans-2,3-enoyl-CoA, within each cycle of fatty acid elongation. Thereby, it participates in the production of VLCFAs of different chain lengths that are involved in multiple biological processes as precursors of membrane lipids and lipid mediators. May be involved in Rac1-signaling pathways leading to the modulation of gene expression. Promotes insulin receptor/INSR autophosphorylation and is involved in INSR internalization (PubMed:25687571).
**Q6ZTR5**	CFA47	Cilia- and flagella-associated protein 47 OS=Homo sapiens OX=9606 GN=CFAP47 PE=2 SV=5	While this gene is well-supported by transcript data, no functional information on its protein product is currently available.

**Q9NUD5**	ZCHC3	Zinc finger CCHC domain-containing protein 3 OS=Homo sapiens OX=9606 GN=ZCCHC3 PE=1 SV=2	ZCCHC3 (Zinc Finger CCHC-Type Containing 3) is a Protein Coding gene. Gene Ontology (GO) annotations related to this gene include nucleic acid binding.
**P20848**	A1ATR	Putative alpha-1-antitrypsin-related protein OS=Homo sapiens OX=9606 GN=SERPINA2 PE=1 SV=1	Putative serine protease inhibitor; This gene encodes a member of the serpin family of proteins, a group of proteins that inhibit serine proteases. The encoded intracellular glycoprotein is localized at the endoplasmic reticulum. This gene is a polymorphic pseudogene, with the non-functional allele being predominant in some populations.
**P82675**	RT05	28S ribosomal protein S5, mitochondrial OS=Homo sapiens OX=9606 GN=MRPS5 PE=1 SV=2	Mammalian mitochondrial ribosomal proteins are encoded by nuclear genes and help in protein synthesis within the mitochondrion. Mitochondrial ribosomes (mitoribosomes) consist of a small 28S subunit and a large 39S subunit. They have an estimated 75% protein to rRNA composition compared to prokaryotic ribosomes, where this ratio is reversed. Another difference between mammalian mitoribosomes and prokaryotic ribosomes is that the latter contain a 5S rRNA. Among different species, the proteins comprising the mitoribosome differ greatly in sequence, and sometimes in biochemical properties, which prevents easy recognition by sequence homology. This gene encodes a 28S subunit protein that belongs to the ribosomal protein S5P family.
**Q5TF21**	SOGA3	Protein SOGA3 OS=Homo sapiens OX=9606 GN=SOGA3 PE=3 SV=1	SOGA3 (SOGA Family Member 3) is a Protein Coding gene. Diseases associated with SOGA3 include Heart Conduction Disease.
**Q69YL0**	NCAS2	Uncharacterized protein NCBP2-AS2 OS=Homo sapiens OX=9606 GN=NCBP2-AS2 PE=1 SV=1	NCBP2-AS2 (NCBP2 Antisense RNA 2 (Head To Head)) is a Protein Coding gene, and is affiliated with the lncRNA class.
**Q8NCX0**	CC150	Coiled-coil domain-containing protein 150 OS=Homo sapiens OX=9606 GN=CCDC150 PE=1 SV=2	CCDC150 (Coiled-Coil Domain Containing 150) is a Protein Coding gene.
**Q9BSJ6**	PIMRE	Protein PIMREG OS=Homo sapiens OX=9606 GN=PIMREG PE=1 SV=1	During mitosis, may play a role in the control of metaphase-to-anaphase transition.
